# Supplementary material for: Florida Keys Cassiopea host benthos-like external microbiomes and a gut dominated by Vibrio, Endozoicomonas and Mycoplasma
Source: PLoS One. 2025 Aug 12;20(8):e0330180. doi: 10.1371/journal.pone.0330180 (PMC12342295; doi:10.1371/journal.pone.0330180)
Supplement: S2 File — Sfig1) Rarefaction curves of all samples. Sfig2) Principal component analysis of Bray-Curtis distance between microbiomes of Cassiopea species. Sfig 3) Upset plot of overlap between taxa found in bell, GVC, water and substrate samples. Sfig4) Heatmap of core microbiome prevalence in GVC and bell. Sfig5) Aldex2 plot of effect size of all ASVs between bell and GVC samples. Sfig6) Aldex2 plot of effect size of all ASVs between bell and substrate samples. Sfig7) Correlation plot (corrplot) of collected environmental and medusa factors across samples. Sfig8) Redundancy analysis plot of GVC. Sfig9) Redundancy analysis plot of bell samples [96,97]. (DOCX) [file pone.0330180.s002.docx]

**Supplementary figures and methodological statements**

Supplement A1: Full length 16S rRNA gene identities

Three samples were sent to CD Genomics for full length 16S on PacBio Sequel using universal 16S primers (F: AGRGTTTGATYNTGGCTCAG; R:TASGGHTACCTTGTTASGACTT). Samples sent for full length 16S rRNA sequencing included gastrovascular cavity swab extractions from medusae Marathon Key GVC 30 and Key West GVC 96 and GVC 120. As the usable reads returned from this were low (597-12520 per sample), these full-length identities are not discussed in the main text. For further details, please refer to the appendix (A1).

Full length 16S rRNA gene sequences (BioProject: PRJNA1020446) were run on mothur v1.48.0 with deviation from the MiSeq SOP on trim length. ASV cutoff was set to five differences, and ASV representative sequences were compared against the NCBI GenBank (Sept 24, 2023) database using BLAST to find nearest matches by e-value.

In order to identify core microbial groups to a lower taxonomic level, three internal medusa gastrovascular swabs (medusae Kmuffet_96, Kmuffet_120 and Kmuffet_30) from Marathon Key (MK) and Key West (GB) were sequence for full length 16S rRNA gene (see S1 for location of samples). As the primers were not chloroplast exclusionary, the Key West sites produced few useable sequences (Kmuffet_96: 597 and Kmuffet_120: 1097). The Marathon Key medusa (Kmuffet_30), likely bleached at time of collection, returned 12,520 non-chloroplast sequences. The Mycoplasma-like full length 16S rRNA gene sequence (Genbank Acc. No. OR592270) from Marathon Key and Key West has no closely related sequences currently identified (greatest identity 88.43% to sequence HQ393440). The *Endozoicomonas* strain from both Marathon Key and Key West (Genbank Acc. No. OR592271) has 99.93% identity to *Endozoicomonas atrinae* strain WP70(T) collected from the intestine of South Korean *Atrina pectinata* (Accession NR_134024)(96) and 99.80% identity (<5 nucleotide differences) with isolates collected from *Gorgonia ventalina* (GenBank Acc. No. GU118345) in Bocas del Toro, Panama (97). *Endozoicomanas* and *Mycoplasma-*like isolates were the only two groups to have ASVs with over 100 sequences.


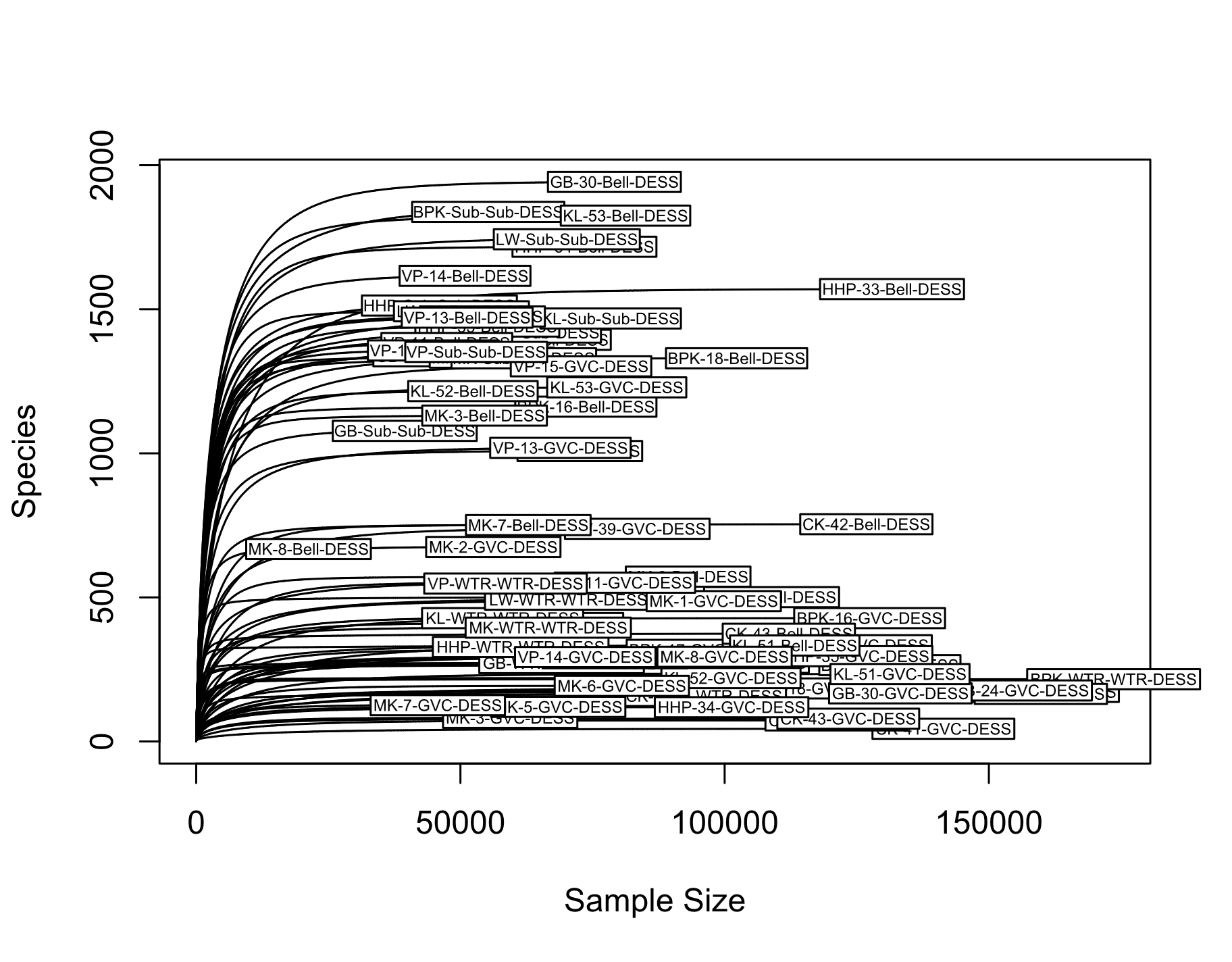


**Supplementary Fig 1. Rarefaction curves of all samples**


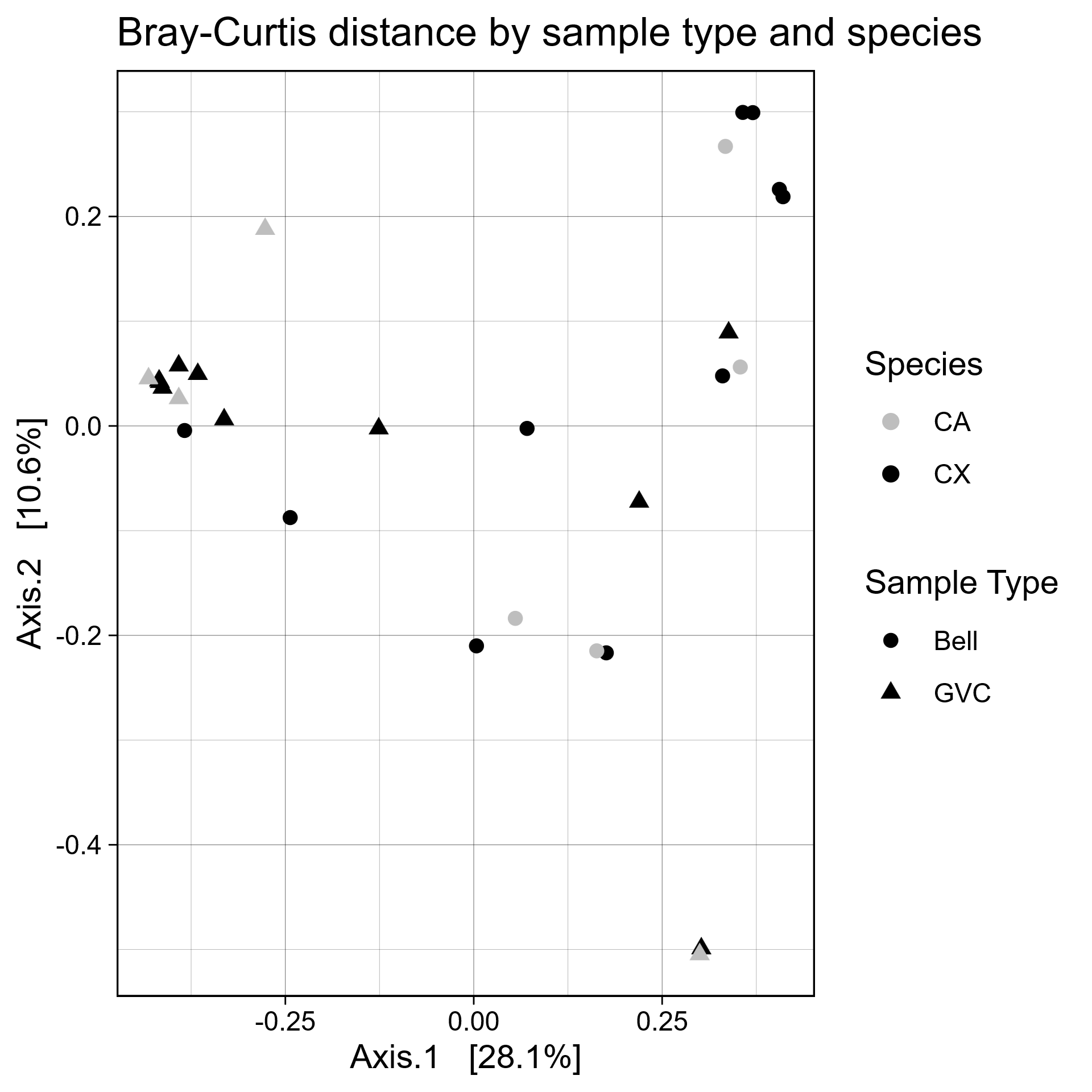


**Supplementary Fig 2. Principal component analysis of Bray-Curtis distance between microbiomes of *Cassiopea* species.** *Cassiopea andromeda* (CA: grey) and *Cassiopea xamachana* (CX: black) samples at the sites were they cooccurred (Cudjoe Key, Garrison Bight and Key Largo). The mitochondrial haplotype of the non-native Cassiopea, Cassiopea andromeda, a cryptic sister species to C. xamachana, was found in four of the 34 medusae sampled. The four C. andromeda mitotype individuals included were collected from the Key Largo (1), Cudjoe Key (2), and Key West (1) sites.


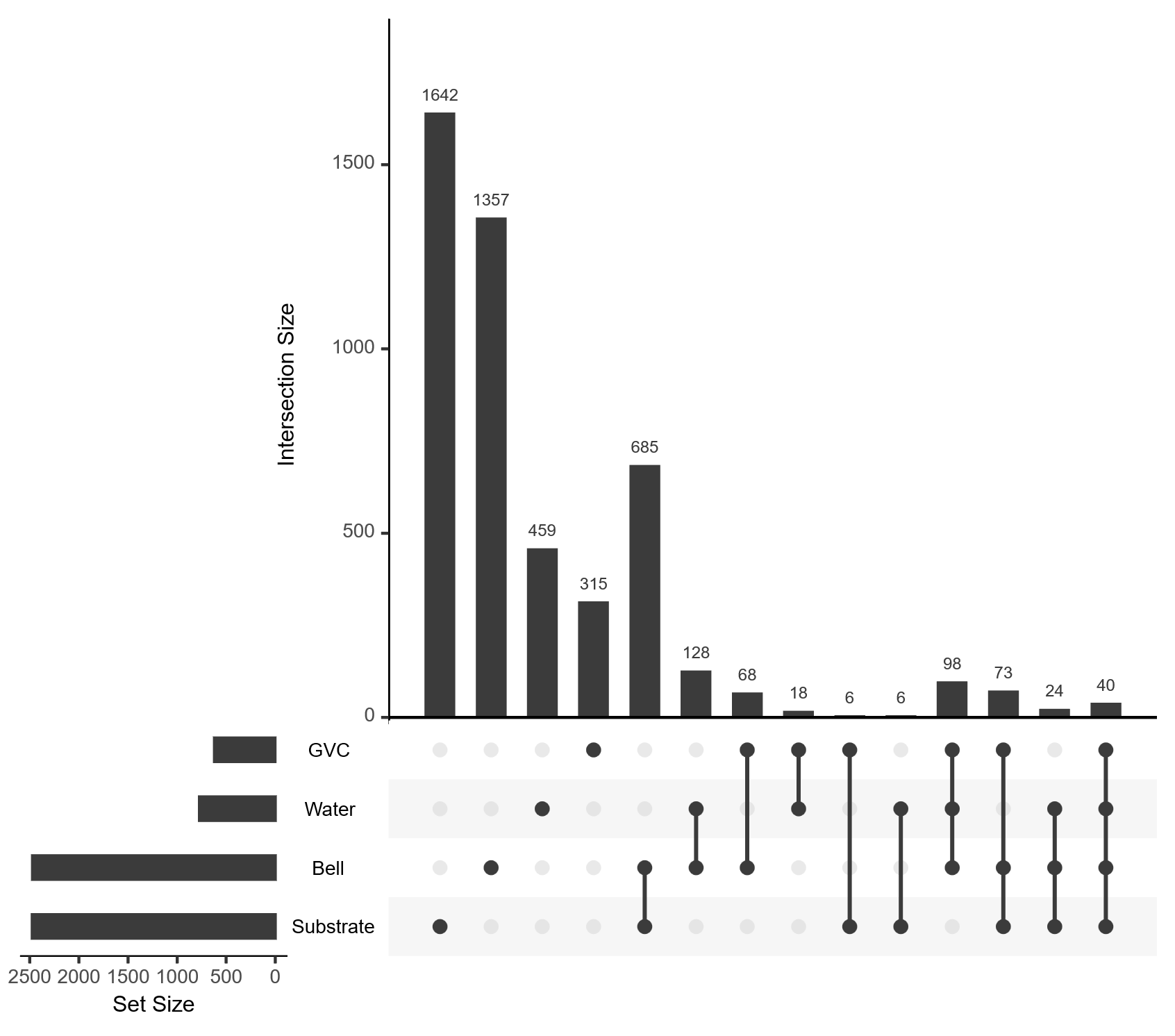


**Supplementary Fig 3. Upset plot of overlap between taxa found in bell, GVC, water and substrate samples.** Taxa were restricted to those with an average of 5 reads per sample of a given type (>40 reads in water or substrate, >150 reads in GVC or bell). Lefthand shows total number of amplicon sequence variants found in each sample type group, top displays number of overlapping ASVs for each intersecting sample type set.


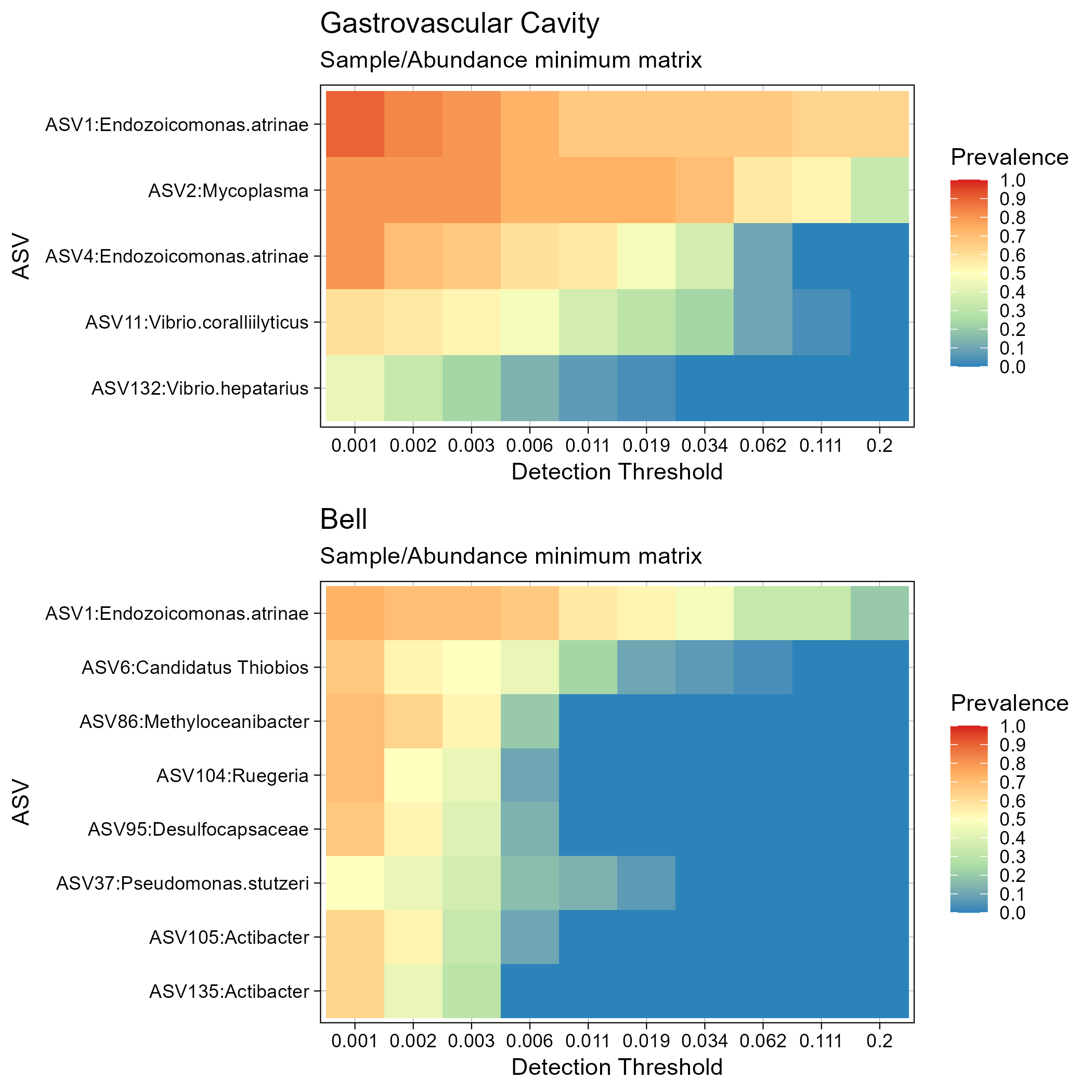
**Supplementary Figure 4. Core microbiome prevalence in GVC and bell.** Heatmap of gastrovascular cavity (top) and bell (bottom) shaded by prevalence (0-1 scale) vs detection threshold (proportions of reads in samples) across the ASVs identified as core in each group.


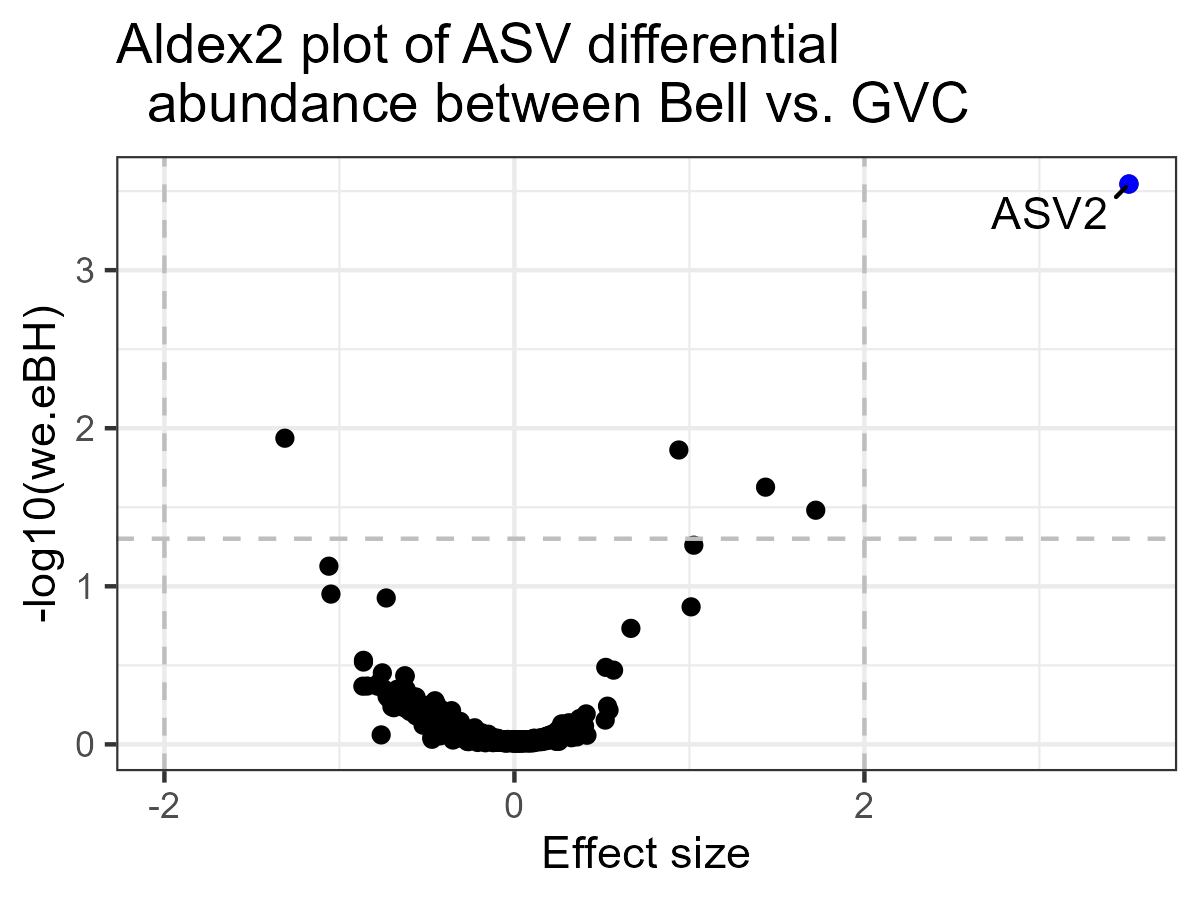


**Supplementary Figure 5. Aldex2 plot of effect size of all ASVs between bell and GVC samples.** Only ASV2 has an effect >[± 2].


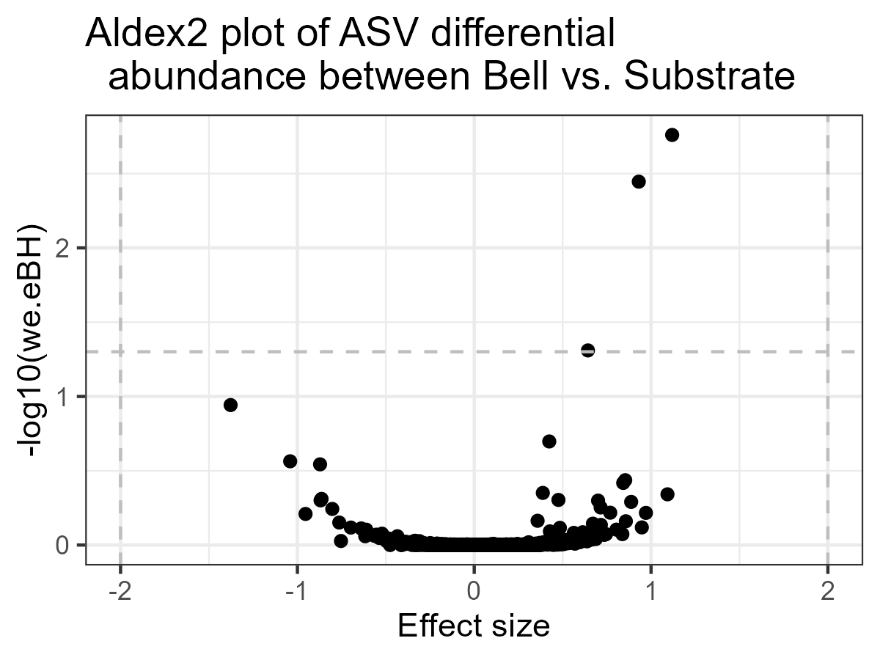


**Supplementary Figure 6. Aldex2 plot of effect size of all ASVs between bell and substrate samples.** No ASVs are significantly different.

**
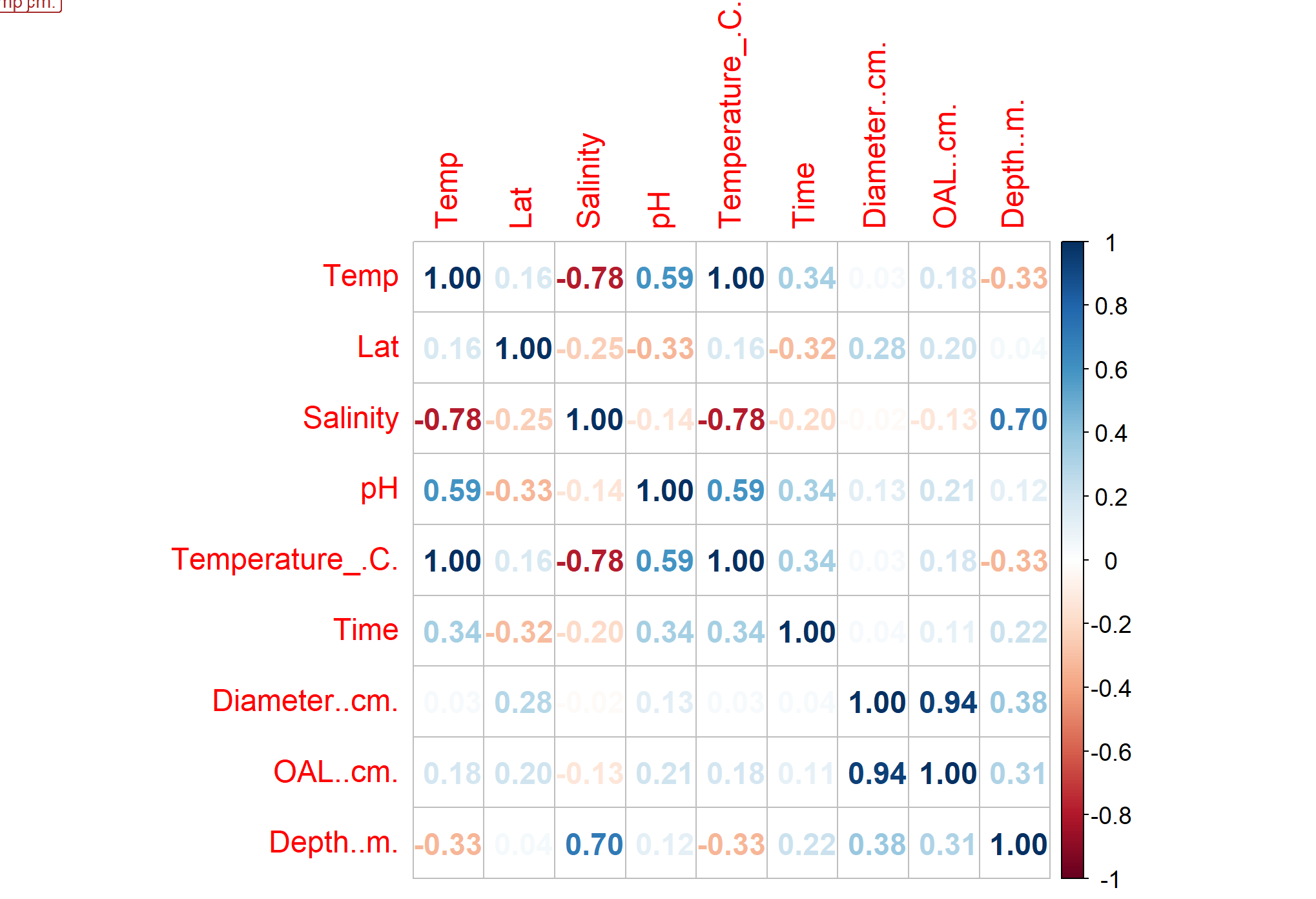
**

**Supplementary Figure 7. Correlation plot (corrplot) of collected environmental and medusa factors across samples.** Color is based on strength of the correlation.


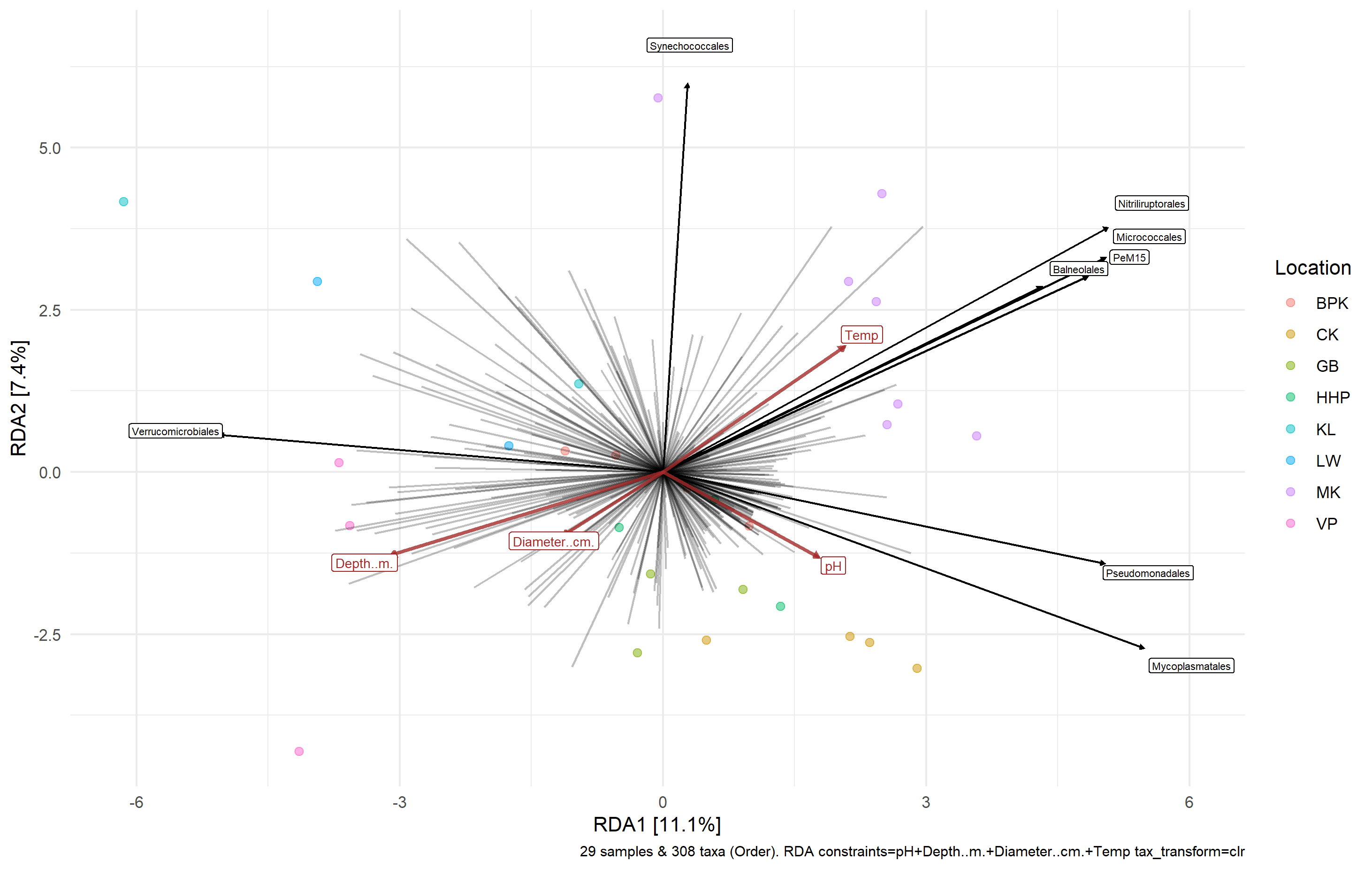


**Supplementary Figure 8. Redundancy analysis plot of GVC.** The relative value of depth, temperature, diameter and pH on RDA axes 1 and 2, as well as the eight most impactful bacterial orders. Points are colored by site of origin.

**
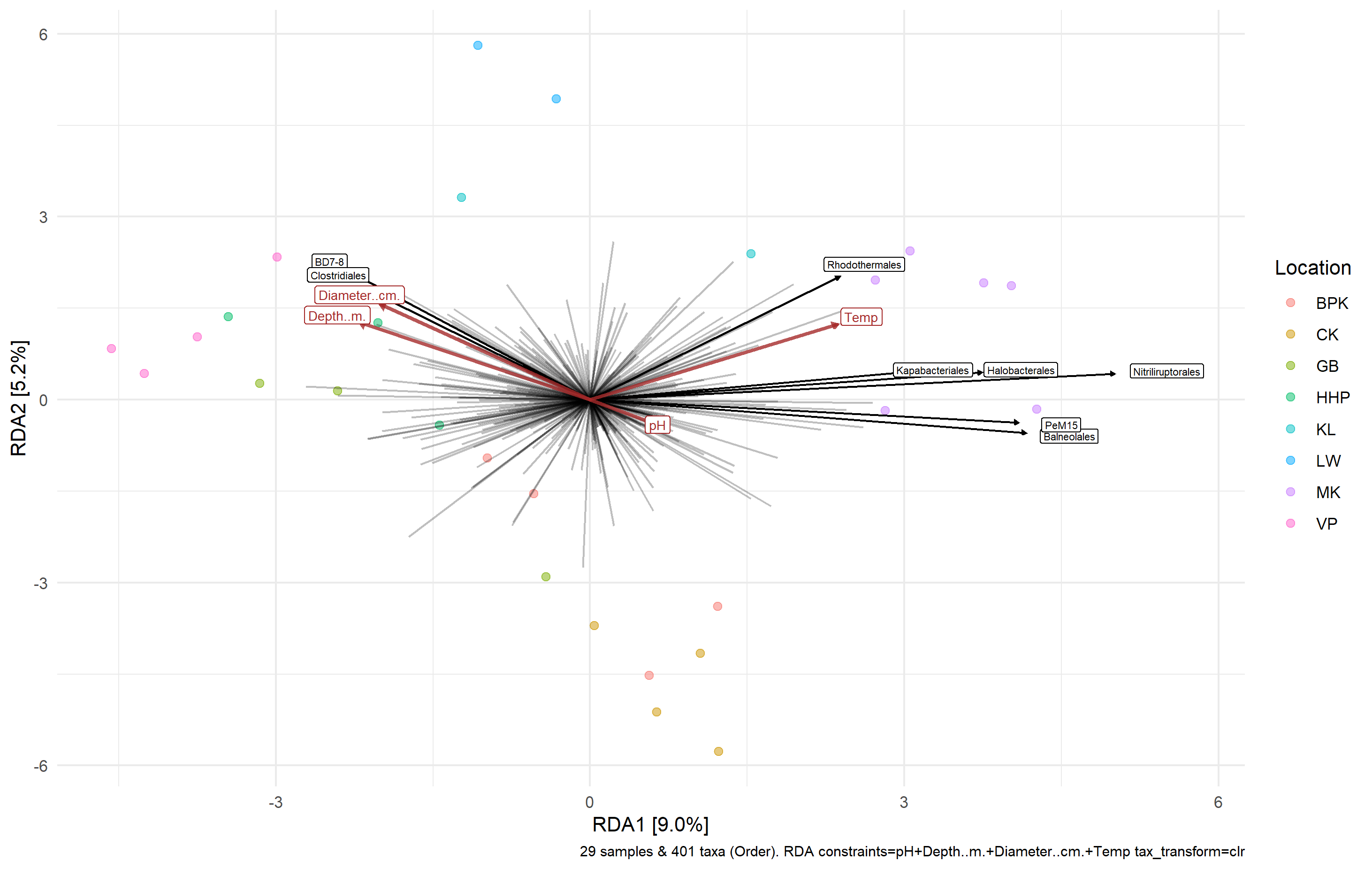
**

**Supplementary Figure 9. Redundancy analysis plot of bell.** The relative value of depth, temperature, diameter and pH on RDA axes 1 and 2, as well as the eight most impactful bacterial orders. Points are colored by site of origin.
