# Supplementary material for: Florida Keys Cassiopea host benthos-like external microbiomes and a gut dominated by Vibrio, Endozoicomonas and Mycoplasma
Source: PLoS One. 2025 Aug 12;20(8):e0330180. doi: 10.1371/journal.pone.0330180 (PMC12342295; doi:10.1371/journal.pone.0330180)
Supplement: Attachment — Submitted filename: PONE-D-24-22287_R1_reviewer.pdf [file pone.0330180.s004.pdf]

PLOS ONE

Gastric microbiomes of Cassiopea in the Florida Keys are low diversity and Endozoicomonas-, Vibrio- and Mycoplasma-dominated

--Manuscript Draft--

**Manuscript Number:**	PONE-D-24-22287R1
**Article Type:**	Research Article
**Full Title:**	Gastric microbiomes of Cassiopea in the Florida Keys are low diversity and Endozoicomonas-, Vibrio- and Mycoplasma-dominated
**Short Title:**	Microbiome of Cassiopea in the Florida Keys
**Corresponding Author:**	Kaden Muffett<br>University of California Merced<br>Galveston, Texas UNITED STATES OF AMERICA
**Keywords:**	Cnidaria; microbiome; symbiosis; Cassiopea; Scyphozoa
**Abstract:**	Interactions with microbial communities fundamentally shape metazoans' physiology, development, and health across marine ecosystems. This is especially true in zooxanthellate (symbiotic algae-containing) cnidarians. In photosymbiotic anthozoans (e.g. shallow water anemones and corals), the key members of the associated microbiota are increasingly well studied, however there is limited data on photosymbiotic scyphozoans (true jellyfish). Using 16S rRNA barcoding, we sampled the internal and external mucus of the zooxanthellate Upside-Down Jellyfish, Cassiopea xamachana throughout eight sites covering the full length of the Florida Keys. We find that across sites, these medusae to have low-diversity internal microbial communities distinct from the communities of their external surfaces and their environment. These internal communities are dominated by only three taxa: Endozoicomonas cf. atrinae, an uncultured novel Mycoplasma, and Vibrio cf. coralliilyticus. Cassiopea bell mucosal samples conform largely to the communities of surrounding sediment with the addition of Endozoicomonas cf. atrinae. The microbial taxa we identify associated with wild Florida Keys Cassiopea bear a strong resemblance to those found within photosymbiotic anthozoans, increasing the known links in ecological position between these groups.
**Order of Authors:**	Kaden Muffett
	Jessica Labonté
	Maria Pia Miglietta
	Corresponding
**Response to Reviewers:**	Response to editor and reviewers<br><br>Dear editor and reviewers,<br>After careful review of your suggestions and criticisms, the methodological and statistical analyses in this manuscript have been revisited, redone, and in the process greatly improved. Many of your broad concerns in these areas are addressed with a significantly expanded methods section.<br>Sincerely,<br>KMM<br><br>Response to editor:<br>Thank you for your considered feedback.<br>In addition to the improved manuscript, please note these specific changes:<br>1.Inclusion of the funder role statement along with the manuscript<br>2.Corrected Funding statement in cover letter<br>3.Fixed formatting in manuscript<br>4.Added ethics statement<br><br>Comments to reviewer 1:<br>Thank you for your careful feedback. After substantial revision to the backbone of this work, we hope you will find all of your comments duly taken into consideration. Please

Powered by Editorial Manager® and ProduXion Manager® from Aries Systems Corporation

	see specific corrections in addition to the broader overhaul in the response below.<br><br>1.Abstract needs to mention consistent results from across sampling sites.<br>a.Thank you for your feedback. This has been corrected.<br>2.Clarify inclusion of C. andromeda in analysis.<br>a.In the text (ln 146) it now clarifies that C. xamachana only are used for downstream analyses with the exception explicitly comparing the two mitotypes (ln 298).<br>3.Standardize “benthic” sample name<br>a.Thank you for your suggestion. All substrate samples are now referred to as substrate as opposed to benthic.<br>4.Figure 2<br>a.Figure two has been replaced with an improved version that includes your suggestions.<br>5.Figure 3<br>a.Figure 3 has been replaced and now is a chart of Shannon and observed diversity<br>6.Citation for Dirichlet multinomial analysis<br>a.The lack of reference to Holme’s original work has been rectified.<br>7.Line 227: claim about clustering distinction<br>a.Thank you. This claim has been removed and has been replaced with Faith’s D and more appropriate accompanying statistical tests.<br>8.Core microbiome information incomplete<br>a.Thank you for the feedback. This section was indeed incomplete and confusing. It has been replaced with analyses from the “microbiome” r package that are more appropriate.<br>9.Lack discussion of external microbiome<br>a.Thank you for this comment. There is now a section discussing this within the paper.<br>10. Seasonality and size comments unnecessary<br>a.Thank you for your feedback, this section has been removed.<br>Specific comments:<br>•Line 33: “These communities” please specify you are referring to “microbial communities”<br>oThis issue has been noted and corrected.<br>•Line 38: replace “clade” with “Family”<br>oThis issue has been noted and corrected.<br>•Line 47: the statement about the tolerance of Cassiopea to intense temperature fluctuations needs a reference<br>oThis issue has been noted and corrected.<br>•Line 63: I do not understand the purpose of the statement: “Pathogenesis is often a focus…”<br>oThis statement has been clarified and corrected.<br>•Line 150-151: Why were you not able to use the Siva 138 database?<br>oThis work now uses the most up-to-date silva database.<br>•Line 165: “benthic OR water” should not be “water AND sediment”?<br>oThis issue has been noted and corrected.<br>•Line 216: Please revise “Family richness was significantly lower within medusae”<br>oThis line has been corrected.<br>•Line 227: after referring to your results, it would be good to add a figure number<br>oFigure numbers are now added in correct positions.<br>•Lines 280-282: this statement has no support. It could work the other way, going from the exterior but remaining an important component in the internal cavity. Please revise.<br>oThis error has been noted and replaced.<br>•Lines 299-300: I do not understand why you mention this statement, since in your results no identification of “obligate” taxa was made for the GVC. Please revise.<br>oThis statement has been noted and revised.<br>•Line 314: a space is needed before the parenthesis<br>oThis issue has been corrected.<br>•Lines 317-319: is not clear what point are you trying to make.<br>oThank you for the feedback, this statement has been removed and replaced with one of greater clarity.<br>•Line 326: use “Family” rather than “clade”<br>oCorrected.<br>•Line 327: orphan phrase? “…appear to harbor the microbiome, …”<br>oThank you for the feedback, this statement has been clarified and reworded.<br>•Line 381: I suggest changing “lacks a conservatively defined core microbiome” for “in

our samples, no core microbiome could be demonstrated”
oThis statement has been removed and replaced after reanalysis.

Comments to reviewer 2:

The authors thank reviewer 2 for their supportive feedback. The poor explanation of statistical tests and underanalysis of the dataset at hand presented key failings that have been carefully corrected in the updated version. We hope that these revisions make this work more meaningful and improve readability.

Reviewer #2: The authors study the microbiome composition of two upside-down jellyfish species in the Florida Keys using 16S rRNA barcoding. The paper describes the main bacterial taxa in different locations and describes the microbiome composition focusing on an understudied group. They briefly compare these findings with the existing literature for other anthozoan groups.
I found the study interesting, but the results seem overstated, and some important information is missing. For example, the title does not accurately reflect the study's findings. It should better represent the study’s scope and avoid claiming general patterns that the data does not appear to support. My major concern is the lack of explanation in the statistical analyses. I think improving this part will help to better understand the analyses and evaluate the content of the manuscript.

Responses to specific comments:

•Overall, there is a lack of information on sample sizes throughout the study. This omission makes it difficult to assess the balance of the study design or to understand how many samples are being compared in each analysis. Sample size details should be included in the figures, methods, and results sections.
oThank you for your comment. The sample size information is now added to the first table and where relevant in other sections.
•The authors use two different preservatives—ethanol and DESS—that appear to be combined without distinction. I recommend including a statement in the methods on how this might affect microbiome composition. If the authors tested for potential effects, the results should be included, or they should reference studies clarifying whether these preservatives could influence the results.
oEthanol was not used for any downstream analyses, a statement that has now been added to the methods section to clarify.
•Table S1: Please provide descriptions for abbreviations used to aid comprehension.
oClarification has now been added to the supplementary information.
•Table 1: Please add the sample sizes per site.
oCorrected
•L129-135: If PacBio sequences were not used in the study, I recommend removing this from the methods section.
oCorrected.
•L157: Please describe the statistical analyses clearly enough to allow reproducibility. This section lacks essential details.
oCorrected.
•Figure legends need explanations of the content. They are currently limited to single-line titles, which restricts comprehension. I recommend revising the figure design, e.g. Figure 1.
oCorrected
•L226: "significantly different".
oThis statement has been replaced and clarified.
•Figure 4: The stress level is high. How balanced is this comparison? Although the figure appears to show a comparison analysis, the text refers to a pairwise comparison. Could the authors clarify the type of analysis used here?
oThank you for your comment. This analysis has been replaced with a more appropriate MDS of Euclidean distance from the corrected data.
•The results section presents many analyses and tests that were not introduced in the methods section. Additionally, the results refer to supplementary tables without legends, making it difficult to assess the accuracy of these tests. I recommend a more detailed explanation of the analyses conducted.

	oThis consistent issue has been corrected and all analyses are now listed in the revised version of the manuscript.<br>•Figure 2: The y-axis label is missing.<br>oThis figure has been replaced with a more appropriate figure.
Additional Information:	
Question	Response
**Financial Disclosure**<br><br>Enter a financial disclosure statement that describes the sources of funding for the work included in this submission. Review the submission guidelines for detailed requirements. View published research articles from *PLOS ONE* for specific examples.<br><br>This statement is required for submission and **will appear in the published article** if the submission is accepted. Please make sure it is accurate.<br><br>**Funded studies**<br>Enter a statement with the following details:<br>• Initials of the authors who received each award<br>• Grant numbers awarded to each author<br>• The full name of each funder<br>• URL of each funder website<br>• Did the sponsors or funders play any role in the study design, data collection and analysis, decision to publish, or preparation of the manuscript?<br><br>Did you receive funding for this work?	Yes
Please add funding details.<br>as follow-up to "**Financial Disclosure**<br><br>Enter a financial disclosure statement that describes the sources of funding for the work included in this submission. Review the submission guidelines for detailed requirements. View published research articles from *PLOS ONE* for specific examples.<br><br>This statement is required for submission and **will appear in the published article** if	This work was funded by Texas Sea Grant, NSF EAGER award # 1936565, and Texas A&M University Galveston. The funders had no role in study design, data collection and analysis, decision to publish, or preparation of the manuscript.

the submission is accepted. Please make sure it is accurate.<br><br>**Funded studies**<br>Enter a statement with the following details:<br>• Initials of the authors who received each award<br>• Grant numbers awarded to each author<br>• The full name of each funder<br>• URL of each funder website<br>• Did the sponsors or funders play any role in the study design, data collection and analysis, decision to publish, or preparation of the manuscript?<br><br>Did you receive funding for this work?"	
Please select the country of your main research funder (please select carefully as in some cases this is used in fee calculation).<br>as follow-up to "**Financial Disclosure**<br><br>Enter a financial disclosure statement that describes the sources of funding for the work included in this submission. Review the submission guidelines for detailed requirements. View published research articles from *PLOS ONE* for specific examples.<br><br>This statement is required for submission and **will appear in the published article** if the submission is accepted. Please make sure it is accurate.<br><br>**Funded studies**<br>Enter a statement with the following details:<br>• Initials of the authors who received each award<br>• Grant numbers awarded to each author<br>• The full name of each funder<br>• URL of each funder website<br>• Did the sponsors or funders play any role in the study design, data collection and analysis, decision to publish, or preparation of the manuscript?	UNITED STATES - US

Powered by Editorial Manager® and ProduXion Manager® from Aries Systems Corporation

Did you receive funding for this work?"	
**Competing Interests**<br><br>Use the instructions below to enter a competing interest statement for this submission. On behalf of all authors, disclose any competing interests that could be perceived to bias this work—acknowledging all financial support and any other relevant financial or non-financial competing interests.<br><br>This statement is required for submission and **will appear in the published article** if the submission is accepted. Please make sure it is accurate and that any funding sources listed in your Funding Information later in the submission form are also declared in your Financial Disclosure statement.<br><br>View published research articles from *PLOS ONE* for specific examples.<br><br>**NO authors have competing interests**<br><br>Enter: *The authors have declared that no competing interests exist.*<br><br>**Authors with competing interests**<br><br>Enter competing interest details beginning with this statement:<br><br>*I have read the journal's policy and the authors of this manuscript have the following competing interests: [insert competing interests here]*<br><br>* typeset	The authors have declared that no competing interests exist.
**Ethics Statement**<br><br>Enter an ethics statement for this submission. This statement is required if the study involved:<br><br>• Human participants<br>• Human specimens or tissue	Special use permitting for these samples was waived by Florida Fish and Wildlife.

• Vertebrate animals or cephalopods<br>• Vertebrate embryos or tissues<br>• Field research<br><br>Write "N/A" if the submission does not require an ethics statement.<br><br>General guidance is provided below. Consult the submission guidelines for detailed instructions. **Make sure that all information entered here is included in the Methods section of the manuscript.**	

Powered by Editorial Manager® and ProduXion Manager® from Aries Systems Corporation

**Format for specific study types**<br><br>**Human Subject Research (involving human participants and/or tissue)**<br>• Give the name of the institutional review board or ethics committee that approved the study<br>• Include the approval number and/or a statement indicating approval of this research<br>• Indicate the form of consent obtained (written/oral) or the reason that consent was not obtained (e.g. the data were analyzed anonymously)<br><br>**Animal Research (involving vertebrate animals, embryos or tissues)**<br>• Provide the name of the Institutional Animal Care and Use Committee (IACUC) or other relevant ethics board that reviewed the study protocol, and indicate whether they approved this research or granted a formal waiver of ethical approval<br>• Include an approval number if one was obtained<br>• If the study involved *non-human primates*, add *additional details* about animal welfare and steps taken to ameliorate suffering<br>• If anesthesia, euthanasia, or any kind of animal sacrifice is part of the study, include briefly which substances and/or methods were applied<br><br>**Field Research**<br><br>Include the following details if this study involves the collection of plant, animal, or other materials from a natural setting:<br>• Field permit number<br>• Name of the institution or relevant body that granted permission	
**Data Availability**<br><br>Authors are required to make all data underlying the findings described fully available, without restriction, and from the time of publication. PLOS allows rare exceptions to address legal and ethical concerns. See the PLOS Data Policy and FAQ for detailed information.	Yes - all data are fully available without restriction

<table>
<tr><td>A Data Availability Statement describing where the data can be found is required at submission. Your answers to this question constitute the Data Availability Statement and will be published in the article, if accepted.

Important: Stating ‘data available on request from the author’ is not sufficient. If your data are only available upon request, select ‘No’ for the first question and explain your exceptional situation in the text box.

Do the authors confirm that all data underlying the findings described in their manuscript are fully available without restriction?</td><td></td></tr>
<tr><td>Describe where the data may be found in full sentences. If you are copying our sample text, replace any instances of XXX with the appropriate details.

• If the data are held or will be held in a public repository, include URLs, accession numbers or DOIs. If this information will only be available after acceptance, indicate this by ticking the box below. For example: All XXX files are available from the XXX database (accession number(s) XXX, XXX.).
• If the data are all contained within the manuscript and/or Supporting Information files, enter the following: All relevant data are within the manuscript and its Supporting Information files.
• If neither of these applies but you are able to provide details of access elsewhere, with or without limitations, please do so. For example:

Data cannot be shared publicly because of [XXX]. Data are available from the XXX Institutional Data Access / Ethics Committee (contact via XXX) for researchers who meet the criteria for access to confidential data.

The data underlying the results presented in the study are available from (include the name of the third party</td><td>All raw sequencing files are available from the SRA database (BioProject accession numbers PRJNA1020446, PRJNA1020388.)</td></tr>
</table>

and contact information or URL).<br>• This text is appropriate if the data are owned by a third party and authors do not have permission to share the data.<br><br>* typeset	
Additional data availability information:	

Powered by Editorial Manager® and ProduXion Manager® from Aries Systems Corporation

Manuscript
Click here to access/download;Manuscript;Clean_Cassiopea_Microbiome_PL

Gastric microbiomes of *Cassiopea* in the Florida Keys are low diversity and *Endozoicomonas*-, *Vibrio*- and *Mycoplasma-dominated*

Kaden M Muffett[1]*, Jessica M Labonté[2], Maria Pia Miglietta[2]

1 University of California Merced, Merced, CA, 95343
2 Texas A&M University at Galveston, Galveston, TX, 77554

*Corresponding email: kmmuffett@gmail.com

Running Page Head: Bacteria of *Cassiopea* in the Florida Keys

Abstract

Interactions with microbial communities fundamentally shape metazoans' physiology, development, and health across marine ecosystems. This is especially true in zooxanthellate (symbiotic algae-containing) cnidarians. In photosymbiotic anthozoans (eg. shallow water anemones and corals), the key members of the associated microbiota are increasingly well studied, however there is limited data on photosymbiotic scyphozoans (true jellyfish). Using 16S rRNA barcoding, we sampled the internal and external mucus of the zooxanthellate Upside-Down Jellyfish, *Cassiopea xamachana* throughout eight sites covering the full length of the Florida Keys. We find that across sites, these medusae to have low-diversity internal microbial communities distinct from the communities of their external surfaces and their environment. These internal communities are dominated by only three taxa: *Endozoicomonas* cf. *atrinae*, an uncultured novel *Mycoplasma*, and *Vibrio* cf. *coralliilyticus*. *Cassiopea* bell mucosal samples conform largely to the communities of surrounding sediment with the addition of *Endozoicomonas* cf. *atrinae*. The microbial taxa we identify associated with wild Florida Keys

Cassiopea bear a strong resemblance to those found within photosymbiotic anthozoans, increasing the known links in ecological position between these groups.

Key words: Cnidaria, microbiome, symbiosis, *Cassiopea*, Scyphozoa

Introduction

Cnidarian-photosymbiont interactions are central to ecosystem function in coral reefs and beyond. Algal photosymbionts, or zooxanthellae, are present in the well-known stony corals, octocorals, and anemones, but also play important roles in a variety of lesser-known jellyfishes, chief among them, jellyfish of the genus *Cassiopea* (1–4). For symbiotic anthozoans, microbial communities play a key role in maintaining health within this photosymbiont partnership, with community members directly conferring disease resistance and heat tolerance on hosts (5–7). These microbial communities are often specialized, with distinct corals demonstrating co-evolution with members of their bacterial communities (8), but retain several prevalent genera, such as *Endozoicomonas*, across many hosts (9). While anthozoan communities are under active
see below or cite microbial
investigation, few analyses have documented the stability and nature of microbiome communities associated with zooxanthellate jellyfishes, a group with divergent life history traits
Family
from Anthozoa but with a comparable obligate symbiosis with dinoflagellates (clade Symbiodiniaceae).

The Upside-Down Jellyfish, *Cassiopea* spp., is a model organism for cnidarian-photosymbiont interactions (10,11). These medusae are residents of tropical and subtropical waters globally and can survive in water that is frequently shallow (as little as 8 cm depth) and hot (up to 33˚C for *C. andromeda*) (12,13). *Cassiopea* obligatorily hosts algal symbionts, losing mass even when well fed while in aposymbiotic conditions (14). As in corals and other scyphozoans, mucus plays a central role for *Cassiopea*, presenting a sticky interface between the host and their benthic environment (15,16). In contrast to other symbiotic cnidarians, such as stony corals, *Cassiopea* can tolerate heat and intense temperature fluctuations(17).

Understanding the *Cassiopea* natural microbial community could provide new potential insights into the function and diversity of cnidarian holobionts.

Past jellyfish (class Scyphozoa) microbial studies have focused on the microbiome of lab-reared specimens (18,19) or limited sample sizes (20–22). While these types of studies may be able to track state changes across life stages (18) and between condition types (symbiotic/aposymbiotic) (19), there is little guarantee that the microbial communities of jellyfish kept under prolonged laboratory conditions ~~translate~~ bear resemblance to *in situ* microbiomes. Recent *in situ* studies have provided insights into microbial taxa associated with some scyphozoans ("true jellyfish") but have not included species of the genus *Cassiopea*. In the moon jellyfish, *Aurelia* spp., individuals from the Chinese coast were *Vibrionaceae*-dominated, while samples from the northwestern Atlantic were *Mycoplasma*-dominated(22,23). Between the Indonesian marine lakes Kakaban, Haji Buang, and Tanah Bamban, *Mastigias* microbial community composition varied significantly by lake, but consistently included *Oceanospirillales* (primarily *Endozoicomonas*-like) (20). In samples across taxa, *Mycoplasma* is a common component of the identified gut and tissue microbiome of jellyfishes, though its function remains unknown (22–27). Vertebrate pathogen research is often a focus of scyphozoan microbiome discovery—*Tenacibaculum* has been identified in several scyphozoan species, e.g. *Cotyllorhiza tuberculata*, *Aurelia aurita*, and *Pelagia noctiluca*, and, as a fish pathogen, which can have important fisheries ramifications (22,23,25,28).

As most studied jellyfish populations occur in coastal waters only sporadically, the relative consistency of *Cassiopea* assemblages provides a system in which location effects can be analyzed at single time points. *Cassiopea* spp. presents an opportunity to act as a comparison group for the microbial communities associated with both other scyphozoans and symbiotic

anthozoans. The ubiquity of *Cassiopea* assemblages within the Florida Keys allows for the examination of differences in the microbial community of one population (Florida Keys *Cassiopea*) at one time point across a broad geographic area.

The aim of this work is to identify the core components of the internal and external mucosal microbiomes of *Cassiopea* and compare the microbial communities of *Cassiopea* across sites of the Florida Keys.

Materials and Methods

Sampling

A total of 55 medusae were collected by wading in eight sites along the length of the Florida Keys in late August 2021 over eight days (see Table 1 for a list of sites). Species identification for each individual is reported in Muffett & Miglietta 2023 and summarized in Supplementary Table S1 (29). At each site, coordinates, temperature, time of day, depth, salinity, and pH were recorded (Table 1). Salinity was measured with a refractometer, and temperature and pH were measured with an Apera A1209. A minimum of two and a maximum of 10 medusae were sampled per site. At each site and in between each medusa, all equipment was cleaned with absolute ethanol and 10% bleach solution. A 1 L water sample was collected from above (>10 cm above the benthos) each medusa aggregation and filtered (0.2 um pore size, Thermo Scientific Cat No.09-740-30G) with a manual sampler as described in the USGS manual
since no good results were obtaeind in ethanol, should eliminate this
water sampling protocol (30), replicate filters were placed in 15 mL of absolute ethanol or dimethyl sulfoxide ethylenediamine tetraacetic acid saturated salt storage solution (1 L pH 7.5: 93.06 g EDTA, 60 mL 20 % NaOH solution, 20 mL 25 % HCl, 40 mL DMSO, 800 mL water,

NaCl to oversaturation; see ref. 30) (commonly referred to as DESS), one filter in each buffer. At each *Cassiopea* medusa aggregation, duplicate scrapings of ~10 g of sediment collections (substrate sample) were placed in either 15 mL of absolute ethanol or DESS storage solution.

Table 1: List of collection sites. Table with latitude, longitude, salinity, pH, surface temperature, average diameter of collected medusae from the site, and date of collection. Number of paired GVC and bell swab samples per collection are for *Cassiopea xamachana* (CX). For collections that included *C. andromeda* mitotyped medusae, the number of *C. andromeda* samples is presented after the dash (CA). These values only include samples collected in DESS.

This table needs work with the headings and column spaces; needs to define : “average col.” and “GVC” meaning

Site Name	Latitude	Longitude	Salinity (ppt)	pH	Temp (C)	Average depth (m)	Average col. medusa diam (cm)	# of GVC Samps	# of Bell Samps	Date (AM/PM)
Key West	24.561526	-81.7881727	36	8.2	30.4	0.56	6.62	CX 3/ CA 1*	CX 3/ CA 1*	15.8.2021 PM
Cudjoe Key	24.6775378	-81.4991819	36	8.4	32.6	0.36	5.91	CX 4/ CA 2*	CX 4/ CA 2*	17.8.2021 AM
Big Pine Key	24.6978731	-81.3572574	35	7.7	29.1	0.15	4.43	CX 4	CX 4	15.8.2021 PM
Bahia Honda Key TP1	24.6825793	-81.2293673	37	8.3	31.8	0.86	12.41	CX 2	CX 2	13.8.2021 PM
Bahia Honda Key TP2	24.6825793	-81.2293673	36	8	29.4	0.51	10.28	CX 2	CX 2	15.8.2021 AM
Marathon Key	24.69396	-81.0980507	31	8.1	35.2	0.08	6.53	CX 7	CX 7	11.8.2021 PM
Lower Matacumbe Key	24.8582157	-80.7267927	35	8.1	32.9	0.89	11.40	CX 2	CX 2	16.8.2021 PM
Tavernier	25.0233783	-80.4940316	31	8.1	33.3	0.35	12.26	CX 3	CX 3	16.8.2021 AM
Key Largo	25.0872104	-80.4415797	35	7.8	32.0	0.56	4.30	CX 3/ CA 1*	CX 3/ CA 1*	18.8.2021 AM

Each *Cassiopea* medusa was collected from shallow waters <1.5 m within a single assemblage at each site. Medusae were gently grasped by the oral arms, lifted from the water, swabbed along the apex of the bell, then placed bell-down onto a pre-prepared dissection plate

(Figure 1). External bell swabs were done in replicate and placed in 3 mL DESS or absolute ethanol. Medusa oral arm base was bisected between oral arm groups, and replicate swabs were

should introduce the term GVC here

inserted and circled the digestive cavity. One internal swab was placed in DESS, the other in ethanol, as with external swabs. A section of bell tissue was then collected and placed in ethanol for mitochondrial species confirmation (see 28). The size of each medusa was measured, and individual medusae were photographed. As sampling was done in a nonsterile environment, blanks for buffer with swab and buffer with filter were also taken.

Figure 1: Sampling strategy for medusae collection onsite. Demonstration of the typical orientation of medusa (right upper) and swab locations (apical bell surface and internal to medusa). GVC access strategy is displayed on the left and relevant environmental samples (water filter, substrate sample) are displayed on the right.

DNA Extraction and Sequencing

Samples in DESS and EtOH were maintained at room temperature for three to ten days and then frozen. DESS can be stored at room temperature for at least thirty days without significantly compromising community integrity (31). DNA from all samples (see Supplementary Table S1) was then extracted using Zymobiomics DNA Kits (PN# D4300T). All samples were pre-tested through the ampliciation of the 16S rRNA gene (variable region V4) amplifications. Only samples that produced visible bands for the V4 region were sent for 16S rRNA V3-V4 preparation and sequencing. While DESS preserved samples yielded good

eliminate from methodology then

amplification results, EtOH-preserved samples largely failed to produce adequate amplification. As such, only DESS-stored samples were analyzed. Sequencing was performed by the University of Texas Medical Branch Sequencing Lab. Library was prepared with Zymo Quick 16S V3-V4 kit (PN #D6400) in 20 uL reactions (10 uL Quick-16S qPCR premix, 4 uL Quick-16S V3-V4

primers (341f: (CCTACGGGDGGCWGCAG, CCTAYGGGGYGCWGCAG) and 806r: (GACTACNVGGGTMTCTAATCC)), 4 uL water and 2 uL sample). qPCR was run using Roche LightCycler 480 Instrument II (10min 95C, [30sec 95C, 30sec 55C, 3min 72C]x20). PCRs were enzymatically cleaned according to Zymo Quick-16S kit protocols, and barcodes were added in a secondary reaction at 20 uL (10 min 95˚C, [30 sec 95˚C, 30 sec 55˚C, 3 min 72˚C]x5). Plates included two replicate microbial community standards (Zymobiomics Cat. No. D6300) and two PCR negative controls. Samples were loaded at 10 pM, with 15% 10 pM PhiX sequencing control, and run on Illumina MiSeq with a 600 cycle v3 kit.

Data preprocessing

Data generated was run through the DADA2 v 1.26 pipeline on R v 4.4 with only limited deviations from the standard protocols for ASV generation(32,33). Briefly, sequences were filtered and trimmed ("filterAndTrim(truncLen=c(285,230), maxN=0, maxEE=c(4,4), truncQ=2,trimLeft = c(17,21)"), errors were error corrected ("learnErrors", "dada"), and sequences were merged ("mergePairs"). Reads between 380 and 458 bp were retained. Chimeric reads were removed ("removeBimeraDenovo"), then taxonomy was assigned using the Silva v 138.1 database ("assignTaxonomy"). Retention metrics were retained and these metrics along with the full code can be found on the github (https://github.com/kadenmuffett/Cassiopea-Code-Microbiome-Repository). Sequence and metadata tables were imported into phyloseq v 1.42, where reads assigned to mitochondria and chloroplast were removed. Contaminants were removed using the decontam package v. 1.18, identifying and removing 47 ASVs based on differential abundance in swab and buffer controls(34,35).

unnecessary

meaning, C. andromeda was discarded?

For all downstream analyses, dataset was limited to those stored in DESS and medusae without a *C. andromeda* mitotype. This includes 60 paired bell and GVC samples (30 of each), 8 water samples and 8 substrate samples with 6.47 mil sequences across 34808 taxa.The complete dataset (NCBI Bioproject Accession number PRJNA1020388) contains 12.78 million reads.

Statistical analyses

Rarefaction

Refraction curves were generated in vegan 2.6.6.1 (“rarecurve”) (SFIG. 1)(36). Shannon diversity was computed with vegan and observed diversity was ploted with phyloseq (“estimate_richness”, “plot_richness”)(34). Faith’s phylogenetic diversity was computed using the MiscMetabar v 0.10.1, phanghorn v 2.12.1, metagMisc v 0.5.0 and PhyloseqMeasures v 2.1 packages by first generating an ML tree from all sequences over 300 reads across the dataset, then computing per sample Faith’s diversity (37–42). Faith’s diversity was displayed with ggstatsplot v 0.13.0 (43). Bray curtis and Euclidean distance were computed in phyloseq with “distance” to “ordinate” to “plot_ordination”. Between group comparisons were completed with the pairwiseAdonis package v 0.4.1 (“ pairwise.adonis2”) on Euclidean distance measurements (44). Statistical comparisons were computed with Kruskal-Wallis using the Kruskal.test and pairwise comparisons using the “kruskal.test” and “dunn.test” commands with holm p adjustment (45).

Data transmutation for starplots and display was assisted by the speedyseq package v 0.5.3.9021 (46). Star plots were made using the stars package v 0.6-7 (47). Differential abundance was calculated with ALDEx2 v 1.38.0 following established pipelines for microbiome data (48,49). Dirichlet Multinomial mixture analysis, as described by Holmes and

implemented in the DirichletMultinomial package v 1.48 (50,51). Core microbiome was computed with the microbiome package v 1.28.0(52).

Ethics Statement

This work was performed on lower level invertebrates not subject to Animal Use Protocols or within the purview of Texas A&M University IRB approval. Permitting for these specimens was waived by Florida Fish and Wildlife Conservation Commision.

Results

Data description

In the sampling section you claimed to have sampled 55 medusae, revise

We analyzed the microbiome associated with 34 medusae from eight sites within the Florida Keys. For each medusa, we analyzed the microbial composition of their gastrovascular cavity (GVC) and external or bell mucus (herein referred to as "bell"), as well as their substrate (Sub) or water environment (WTR). All medusae were identified as *Cassiopea xamachana*, with the exception of four medusae identified as mitochondrial haplotype *C. andromeda* found in Key West, Cudjoe Key, and Key Largo (see Supplementary Table S1). 10.13 and 9.07 million reads

not clear in respect to what?

were retained after filtering ("filterAndTrim") and merging ("mergePairs") respectively (for all retention statistics, see supplementary table S2) . There were 8.91 million sequences after chimera removal. After primary phyloseq filtration, the dataset included 6.47 million sequences retained in 34808 unique ASVs across 76 total samples (30 bell, 30 GVC, 8 water, 8 substrate). Reference sequences for top ASVs are also available in the supplementary material (table S3).

Distribution of sequences in full dataset

The dataset generated was predominantly bacteria with few archael reads. At the phylum level, 57% of sequences were Proteobacteria, 7% were Firmicutes, 13% were Bacteroidota, and 4% were Cyanobacteria (Fig 2). No other phylum had greater than 3% relative abundance in the dataset.

Figure 2. Top ten (top) phyla and (bottom) class relative abundances of sequences in samples aggregated by sample type.

At the class level, Gammaproteobacteria comprised 41% of all sequences. Alphaproteobacteria, the next most abundant, comprised 17%. Bacteriodia (12%), Bacilli (6%), Cyanobacteriia (4%), and Desulfobacteria (3%), were the other classes with a relative abundance above 2%. No Archaean group was prevalent above a relative abundance of 0.25%, with the three most abundant classes being Halobacterota (0.25%), Thermoplasmatota (0.21%) and Nanoarchaeota (0.22%).

Distribution of sequences within gastrovascular cavity (GVC) samples

Within gastrovascular cavity samples, 75% of all sequences belonged to just three orders, Pseudomonadales (39%), Mycoplasmatales (24%), and Enterobacterales (13%). The majority of Pseudomonadales sequences identified within the GVC were dispersed across very few OTUs, with 66% identified as *Endozoicomonas atrinae* ASV1 and 8% as *E. atrinae* ASV4. This was true of the Mycoplasmatales as well, with 77% of associated gut sequences identified as *Mycoplasma* sp. ASV2 or *Mycoplasma* sp. ASV9 (15%).

why did not mention Enterobacterales?

Distribution of sequences within bell mucus samples

At the phylum level, within the bell mucosal swabs, 55% of the sequences were Proteobacteria, 14.5% Bacteroidota, 5.1% Cyanobacteria, and 6% Desulfobacterota.

At the order level, the most abundant groups were Pseudomonadales (23%), Chromatiales (6%), Rhodobacterales (7%), Desulfobacterales (5%), and Flavobacteriales (5%). *Endozoicomonas atrinae* ASV1 was the single most abundant surface mucus ASV at a mean of 12%. The next most abundant were Synechococcus CC9902 ASV15 (1.3%), *Endozoicomonas atrinae* ASV4 (1.1%) and Chromatiales *Candidatus Thiobios* ASV6 (0.8%).

Substrate and water samples

Water samples shared some ASVs with the bell mucus of medusae, the largest contributing ASV was Chromatiales *Candidatus Thiobios* sp. ASV6 (8%). Additional prominent taxa were Rhodobacteraceae *HIMB11* ASV12 (6%), *HIMB11* ASV19 (5%), and SAR11 Clade III ASV 10 (4%). By contrast, substrate samples had no ASVs surpassing a mean of 1% abundance across sites, with the highest two ASVs Sandaracinaceae ASV94 and *Actibacter* ASV105 reaching 0.6% mean abundance across sediment samples (see table 2; extended table is table S4).

Table 2: Top 5 ASVs for each sample type. Table with ASVs ordered from highest to lowest average proportion in samples of each type dispayed as a percentage of composition within that sample type. Includes Silva 138 taxanomic assignment for each ASV.

	1	2	3	4	5
	ASV1 *Endozoicomonas atrinae*	ASV15 *Synechococcus* CC9902	ASV4 *Endozoicomonas atrinae*	ASV6 *Candidatus Thiobios*	ASV10 SAR11 *Clade III*
Bell	11.585 %	1.272 %	1.105 %	0.832 %	0.709 %
	ASV94 Sandaracinaceae	ASV105 *Actibacter*	ASV95 Desulfocapsaceae	ASV169 *Robiginitalea*	ASV358 *Clostridiisalibacter*
Sub	0.625 %	0.605%	0.566%	0.492%	0.474%
	ASV6 *Candidatus Thiobios*	ASV12 Rhodobacteraceae *HIMB11*	ASV19 Rhodobacteraceae *HIMB11*	ASV10 SAR11 *Clade III*	ASV18 *Marinobacterium*

WTR	7.521 %	5.839%	4.725%	4.497%	2.711%
	ASV1 *Endozoicomonas atrinae*	ASV2 *Mycoplasma*	ASV9 *Mycoplasma*	ASV4 *Endozoicomonas atrinae*	ASV11 *Vibrio coralliilyticus*
GVC	34.133%	20.099%	2.823%	2.327%	2.094%

Diversity

Add subtitle: Alfa diversity

Shannon diversity varied by sample type (Kruskal-Wallis: chi-squared = 48.8, df = 3, p-value =1.412e-10). Shannon diversity had a mean of 2.30 in GVCs, 4.01 in water samples, 5.63 in substrate samples and 6.56 in bell samples. Shannon diversity indices for the gastrovascular samples varied from substrate samples and bell samples (Kruskal-Wallis Dunn test with Holm correction: GVC-Bell, p-val=0.00; GVC-Sub, p-val= 0.00; GVC-Water, p-val=0.07; Bell-Sub, p-val=0.07; Bell-Water, p-val=0.06; Water-Sub, p-val=0.01). Shannon diversity was not explained by location (Kruskal-Wallis: chi-squared = 9.7, df = 7, p-value =0.21)(Fig. 3).

Figure 3. Shannon diversity (a) by sample type and (b + c) location. GVC, bell, substrate and water Shannon diversity are shown across both the entire dataset or when segmented by sample type across sites with (b) GVC and (c) bell alpha diversity across sites. All p values of holm-adj Dunn comparison of means tests below a = 0.05 are displayed, with statistical test details displayed above each plot.

Beta diversity

Euclidean distance showed differences between medusa and non-medusa samples (pairwise.adonis, GVC vs bell: p=0.001, bell vs sub: p=0.049, bell vs water: p=0.022, GVC vs sub: p=0.001, GVC vs water: p=0.001, sub v water p=0.001). Variability along the primary axis

was found in both GVC and bell samples, while variability along the secondary axis was found mainly in GVC samples. The location of collection had no clear impact on beta diversity.

Figure 4. PCA of Euclidean distance of samples. PCA is colored by sample collection latitude from low (orange) to high (purple). Sample type is designated by shape, circle (Bell), triangle (GVC), square (Substrate) and cross (Water). The plots show the same PCA faceted by sample type.

Faith's diversity of communities were distinct from each other (Kruskal-Wallis test, chi-squared = 44.1, df = 3, p-value = 1.4e-09). GVC communities were distinct from bell and substrate comparison groups, while bells were distinct from water and gastrovascular cavities (Dunn test with holm correction) (Fig. 5).

Figure 5. Violin plot of Faith's diversity. Samples are colored by sample type. Kruskal-Wallis chi-sq, pvalue and pairwise dunn test with holm correction p-values are displayed. Differences between GVC-water and substrate-bell are not significant.

Core microbiome

I think this section needs to take into consideration that the sampling was done in one month within a single year.

Core microbiome for the purposes of this study was evaluated based on 70% occurrence of an ASV and a relative abundance of > 0.1% of reads in all 34 gastrovascular cavity samples or external bell samples. This is in line with thresholds for other core microbiome work (53). The most abundant ASV, *Endozoicomonas* ASV1, was present in 90% of GVC samples at a relative abundance of > 0.1% of reads, ASV2 and ASV4 were present in 80% . Using less stringent criteria (>70% of samples, any abundance), ASV 11, ASV 132 and ASV 6 become part of the

core microbiome (Fig 6). These ASVs are across only three genera- *Endozoicommonas*, *Mycoplasma* and *Vibrio* (full taxonomic details of ASVs can be found in table S5).

Figure 6. Abundance of Cassiopea core GVC microbiome across dataset. The top five ASVs from the gut core microbiome across all sample types (Bell, GVC, Substrate (Sub) and Water (WTR)) organized from furthest south to furthest north sites. Note the difference in y axis maxima between bell and GVC samples (top) and substrate and water samples (bottom).

In bell samples, ASV1, ASV104, and ASV86 have 73%, 70% and 70% relative abundance in samples at a 0.1% threshold. ASV6, ASV95, ASV37, ASV105, and ASV135 are present at any level in >70% of bell samples. These ASVs cover the genera *Endozoicommonas*, *Candidatus Thiobios*, *Methyloceanibacter*, *Ruegeria* and unknown genera from Desulfocapsaceae and Actibacter (Fig. 7).

Figure 7. Core microbiome prevalence in GVC and bell. Heatmap of gastrovascular cavity (top) and bell (bottom) shaded by prevalence (0-1 scale) vs detection threshold (proportions of reads in samples) across the ASVs identified as core in each group.

In terms of abundance, internal core microbiome components are not just present, but highly dominant in *Cassiopea*'s gut. Some of these taxa are highly restricted to GVC samples (*Mycoplasma*), while others are prevalent across body surfaces (*Endozoicomonas*) (Fig. 7).

The vast majority of ASVs were not differentially abundant between bell and GVC samples. Differential abundance (Aldex2, min effect size absolute value >[±2]) between the gastric and bell surfaces is restricted to the novel *Mycoplasma* group (ASV2), which is

completely lacking in external samples (Fig 8). No features were differentially abundant between substrate and bell samples (Sfig 2).

Figure 8. Aldex2 plot of effect size of all ASVs between bell and GVC samples. Only ASV2 has an effect >[± 2].

Dirichlet multinomial mixture (DMM) analysis identified specific proportions of many of these core microbiome as valuable for category differentiation. Within animal-associated samples, model probability value was optimal for two communities, one GVC and one bell without subcommunities. False assignment rate (mismatch between predicted community and true sample type) was present in 5/60 samples. ASV1 amount was a core group differentiator, with fewer meaningful ASVs defining the GVC community than the bell community (Fig. 9).

Figure 9. Most impactful ASVs for Dirichlet multinomial modeling. Top 0.5% most impactful drivers of body cavity prediction in GVC (top) and bell (bottom) Dirichlet multinomial models ordered from largest to smallest effect size.

Despite DMM identifying all gastric samples as belonging to the same community, relative proportions of those community members differed dramatically, varying substantially in abundance of *Endozoicommonas*, *Mycoplasma* and *Vibrio*. Variation within bell samples primarily occurred with proportion of *Endozoicommonas,* but was not reliably distinguishable from substrate samples by DMM (Fig. 10).

No correlation of physical parameters can be noted for any sample

Figure 10. Star plots of community composition across sites. Star plots for internal (GVC), external (Bell) mucus swabs, water (WTR) and substrate (Sub) sample averages at each site separated by order (three most prominent orders and "other"). Each spoke of the star plot ranges from 0% (center) to 100%. **Internal** group, starting from the 3' position and going counterclockwise: Pseudomonadales, Mycoplasmatales, Enterobacteriales and "Other". **External** group spokes (going counterclockwise): Pseudomonadales, Rhodobacterales, Chromatiales, and "Other". **Water** group spokes (going counterclockwise): Pseudomonadales, Rhodobacterales, Flavobacteriales, and "Other". **Substrate** group spokes (going counterclockwise): Rhodobacterales, Flavobacteriales, Desulfobacterales, and "Other". Each site's measured environmental conditions (salinity, water surface temperature, and pH) are presented below star plots, colored by intensity (low to high represented by pale to dark) . Sites are mapped onto their location within the Florida Keys.

Vibrio

ASVs identical to *Vibrio coralliilyticus* were found across *Cassiopea* gastric samples, with abundance of up to 10% of reads within the sample. This far surpasses rates in the surrounding water and substrate samples (ASV11 averages: Bell: 0.05%, WTR: 0.03%, Sub: 0%, GVC: 2.09%) (Fig. 11).

Figure 11. Percent of reads (0%-10%) assigned to *V. coralliilyticus* ASVs in bell, GVC, substrate and water samples averaged by site. Organized by body cavity**.**

Host medusa species identity

I think this section is not necessary

The mitochondrial haplotype of the non-native *Cassiopea*, *Cassiopea andromeda,* a cryptic sister species to *C. xamachana*, was found in four of the 34 medusae sampled. The four *C. andromeda* mitotype individuals included were from the Key Largo (1), Cudjoe Key (2), and Key West (1) sites. While these were excluded from the primary analysis, *Cassiopea andromeda* showed no divergence from the trends of the *C. xamachana* simultaneously collected (Fig. 12).

I think this figure is not necessary

Figure 12. PCA of Euclidean distance between *Cassiopea* species. *Cassiopea andromeda* (CA: grey) and *Cassiopea xamachana* (CX: black) samples at the sites were they cooccurred (Cudjoe Key, Garrison Bight and Key Largo).

Discussion

Cassiopea medusae show consistent differences in the microbial communities present on their bell and gastric surfaces. Gastrovascular cavity samples have lower diversity than the sediment, external surfaces, and host a novel *Mycoplasma* lineage. This low diversity is consistent with results for multiple other scyphozoans— including *Cotylorhiza tuberculata* (25). *Cassiopea* also appears to have some variation in community composition between sites, a feature consistent with results from other species, such as *Aurelia*, *Mastigias*, and *Tipedalia* (20,24).

A bifurcated outer and inner microbiome

Across all sites, the gastrovascular cavities of *Cassiopea* medusae showed a core microbiome comprised of taxa previously found in photosymbiotic cnidarians. The external microbial community of *Cassiopea* from the Florida Keys adheres fairly closely to the epibenthic microbial community at each site, with the addition of *Endozoicomonas*. ASV1 *Endozoicomonas* cf. *atrinae* may be a primary mucosal component and extend to the exterior of the body through mucus exchange, as it is less prevalent on the bell surface than internal for most individuals. Alternitavely, ASV1 may be a common mucosal resident with no function for the host.

References?

The abundant *Mycoplasma* and *Vibrio* cf. *coralliilyticus* from the gastric cavity of *Cassiopea* were not found in any external mucus samples and found at far lower abundances in external mucosal samples respectively.

The high diversity of the *Cassiopea* bell microbiome found in this study may be unique to *Cassiopea*. Previous work has reported in the low-diversity mucosa, oral arms and umbrella of a previously studied pelagic rhizostome, *Rhizostoma pulmo*, a likely result of the constant contact of *Cassiopea* with the epibenthos (26). The limited overlap between diverse bell communities and low diversity gut communities in *Cassiopea* is notable and consistent with the significant differences in body compartment microbiome of other scyphozoans and anthozoans (21,24,54). While the lack of diversity in scyphozoan microbiomes may seem distinct from many described stony coral microbial communities, these stony coral microbial profiles are often produced using whole body sampling. Lower diversity gastrovascular cavities with distinct community composition may also be a feature of stony corals (55–57).

Microbial communities across sites within this study were not significantly different from each other, as gastrovascular cavity components remained dominated by three taxa throughout the Keys. This microbiome bears limited resemblance to those sampled in common garden experiments and no resemblance to lab-held lines, demonstrating that “core” here should not be misconstrued as obligate (19,58). The lack of obligate bacterial taxa in the *Cassiopea* gastrovascular cavity is also consistent with other microbial hosts, notably the anemone *Exaiptasia diaphana*, another successful model cnidarian (1). While *Cassiopea* require their algal symbiont, bacterial partners may be transient, and bacteria may have far less significance to host health for *Cassiopea* than for specialized obligate hosts (e.g., leafhoppers) (59–62). Without a better understanding of specific metabolic function of different bacterial associates,

microbiome importance cannot be determined. We identified *Endozoicomonas*, *Mycoplasma*, and *Vibrio* as the three most common bacterial genera found within the gastrovascular cavity of *Cassiopea* in the Florida Keys. These are all commonly associated with coral microbial communities (8,9,63). Below, we provide information on these common bacterial components of cnidarian holobionts.

Endozoicomonas functions in microbiomes

The large proportion of *Endozoicomonas* in internal and external samples fits within a broader framework of both *Endozoicomonas* and known cnidarian-associated microbial communities; *Endozoicomonas* strains are present in a variety of invertebrates, including many corals, sponges, sea anemones, scyphozoans and cubozoans (9,63–66). Some hosts house multiple distinct *Endozoicomonas* genotypes, suggesting that different *Endozoicomonas* clades may provide different metabolic services to a host (e.g., Vitamin B12 metabolism) (67). *Endozoicomonas* is more commonly identified in healthy scleractinian corals than bleached and diseased ones (68). For these corals, it may play a role in a variety of fundamental *Symbiodinium*–host interactions, including nutrient transport and symbiont cell breakdown (67). Notably, *Endozoicomonas* is not a consistent marker of microbiome health, its disappearance and reappearance without apparent host health declines has been identified in octocorals (2,66). In some coral, members of *Endozoicomonas* are found in close proximity to *Symbiodinium*, it is unknown whether this feature is shared with *Cassiopea* (67).

Within scyphozoa, *Endozoicomonas* is abundant in the microbial community of the rhizostome scyphozoan *Mastigias* (20). While *Mastigias*, like *Cassiopea*, host zooxanthellae from the family *Symbiodiniaceae*, not all symbiotic rhizostomes harbor the microbe, and not all taxa with the microbe are symbiotic (20,25). *Endozoicomonas* may be common across medusae,

but the difficulty of culturing some strains may have excluded them from the results of past culture-based analyses (66,67). Greater clarity is needed on the function of *Endozoicomonas* for these medusae and other taxa.

Mycoplasmataceae: a poorly understood cnidarian microbiome resident

Mycoplasma, like *Endozoicomonas*, is present in studied coral and scyphozoan microbiomes (9,22,23,25). While intracellular parasitism is common for members of Mycoplasmataceae, those associated with cnidarians may be located extracellularly and exist as opportunistic commensals (2,69). *Mycoplasma* sp. is noted as a potential endosymbiont in lab-raised *Aurelia* polyps, while adults from many jellyfish populations are known to have them (22,24,27). The high divergence between this *Mycoplasma* and others of the genus (closest sequence identity on NCBI at 89%: Accn. No. NR_115937) suggests that this may be a hitherto unknown group. Whether this *Mycoplasma* represents an endosymbiont, a result of prey capture, or a parasite, its exclusivity to the gastrovascular tract in *Cassiopea* sampled within the Keys is notable, as other studies have not demonstrated gastrovascular cavity localization (24,27).

Vibrionaceae

Vibrionaceae is both a core part of coral microbiomes (9,70) and known to facilitate metamorphosis within *Cassiopea* (71,72), however, it is a highly diverse family. While *Vibrio* spp. are found in gut communities of presumed healthy scyphozoans (Daley et al. 2016, Peng et al. 2021) NUMBERS, they can also be associated with senescence and disease (Kramar et al., 2019; Tinta et al., 2012) NUMBERS. For *Cassiopea* specifically, an increase in *Vibrio* has previously been associated with

an aposymbiotic or stressed state (58). Given the variation in Vibrionaceae species and strains, family-wide statements on the beneficial or harmful nature of Vibrionaceae within *Cassiopea*

is unknown

gastrovascular cavities should not be made.

Vibrio coralliilyticus

In this dataset we found ample signal of a 16S V3-V4 with 100% sequence match to multiple *Vibrio* strains, including *V. coralliilyticus* and the oyster pathogen, *V. tubiashii*. As *Vibrio* species are highly varied, the sequence match does not mean assured pathogenicity towards any taxa (75–77). The apparent health of the medusae from whom these samples were collected suggest a either a lack of impact of this specific group on the host or a host-microbe facultative interaction between this *Vibrio* and *Cassiopea*. While there is data on *Cassiopea* microbiome antimicrobial isolates and *Cassiopea* mortality when faced with *Serratia marcescens* infection, we lack adequate details on the *Vibrio* – *Cassiopea* system to determine whether this represents a lowlying widespread infection or a mutualism (78,79).

Drivers of external microbiome

While the external microbiome was not distinct from the sediment around it, it is worth discussing the major groups more common externally than internally. While the core bell

was this written different on purpose???

microbiome represents a substrate-like community, the bell core microbiome includes representatives from families and genera with a variety of potential metabolic capacities. The most plentiful of these ASVs was *Candidatus Thiobios*, known as a sulfur-oxidizing epibiont of the *Zoothamnium niveum* (80,81). As this bacteria can be found in the Florida Keys, their appearance on *Cassiopea* may be circumstantial, but is consistent across medusae (80). Isolates from *Methyloceanibacter* have been demonstrated to be methane oxidizers from marine

environments (82). These may be sediment associated or may recruit to the host to utilize algal products. The potential sulfate reducers within Desulfocapaceae may similarly recruit to the bell and its access to sediment pore waters (83). While *Pseudomonas* cf. *stuzeri* was not as high abundance as the other metabolizers listed above, it may have a role in denitrification or coral health for some species (84–86)

In addition to microbes with known metabolic functions that align with the *Cassiopea* holobiont, the genus *Ruegeria* includes many marine species and has been identified in the microbiomes of photsymbiotic and nonphotosymbiotic cnidarians alike (9,73,87). As Dirichlet modeling for bell microbiomes gave similar weight to many bacteria, the relative impact of all of these bacterial epibionts on the host is unclear.

Laboratory cultures

Cassiopea are primarily used for in-lab studies on symbiosis (12,14,88). Some of these use wild-caught individuals, and others use lab-bred cultures, many of which have been held for years. The lab-raised *Cassiopea* used in Rothig et al. 2021 appear to have a completely different microbiome than the wild individuals caught here (19). This potential discrepancy should be considered when using wild-caught or captive-bred individuals, as captive-bred symbiosis and tolerance levels may differ from medusae in the wild. These discrepancies in microbiome are found in other model cnidarians, such as *Exaiptasia* (1). The information on wild *Cassiopea* microbial communities from the Florida Keys presented here may allow researchers working with lab-raised individuals to better contextualize the taxa that they find in cultured medusae.

Conclusion

The microbiome of *Cassiopea* within the Florida Keys demonstrates strong body compartment discrimination and includes a central group of ASVs overrepresented in the gut and bell mucus. All of these families, Pseudomonadales, Vibrionaceae, and Mycoplasmataceae, are found in other medusae and anthozoans. Despite this overrepresentation of some microbial taxa within the gut, the *Cassiopea* bell is highly diverse, and overlaps with the communities of the water and substrate in and on which it resides. The consistency of *Cassiopea* microbial communities across the Florida Keys demonstrates that specific taxa are strongly associated with *Cassiopea* and that this can extend across many sites with diverse physical conditions. This work provides a greater understanding of the wild microbiome of the common lab organism *Cassiopea*.

It is not clear from your manuscript how was the environemntal parameters info used, if any.

Acknowledgements

The present work was part of KMM's PhD dissertation. Thank you to Dr. David Retchless for input on figure design. We thank the Florida Fish and Wildlife Conservation Commission for their help and guidance.

References

1. Brown T, Otero C, Grajales A, Rodriguez E, Rodriguez-Lanetty M. Worldwide exploration of the microbiome harbored by the cnidarian model, Exaiptasia pallida (Agassiz in Verrill, 1864) indicates a lack of bacterial association specificity at a lower taxonomic rank. PeerJ. 2017;2017(5).

2. Van De Water JAJM, Allemand D, Ferrier-Pagès C. Host-microbe interactions in octocoral holobionts - recent advances and perspectives. Microbiome. 2018;6(1):1–28.

3. Meyer JL, Castellanos-Gell J, Aeby GS, Häse CC, Ushijima B, Paul VJ. Microbial Community Shifts Associated With the Ongoing Stony Coral Tissue Loss Disease Outbreak on the Florida Reef Tract. Front Microbiol. 2019;10(September):1–12.

4. Djeghri N, Pondaven P, Stibor H, Dawson MN. Review of the diversity, traits, and ecology of zooxanthellate jellyfishes. Mar Biol [Internet]. 2019;166(11):1–19. Available from: https://doi.org/10.1007/s00227-019-3581-6

5. Baldassarre L, Ying H, Reitzel AM, Franzenburg S, Fraune S. Microbiota mediated plasticity promotes thermal adaptation in the sea anemone Nematostella vectensis. Nat Commun. 2022;13(1).

6. Ushijima B, Gunasekera SP, Meyer JL, Tittl J, Pitts KA, Thompson S, et al. Chemical and genomic characterization of a potential probiotic treatment for stony coral tissue loss disease. Commun Biol. 2023;6(1).

7. Connelly MT, McRae CJ, Liu PJ, Martin CE, Traylor-Knowles N. Antibiotics Alter Pocillopora Coral-Symbiodiniaceae-Bacteria Interactions and Cause Microbial Dysbiosis During Heat Stress. Front Mar Sci. 2022;8(January):1–18.

8. Pollock FJ, McMinds R, Smith S, Bourne DG, Willis BL, Medina M, et al. Coral-associated bacteria demonstrate phylosymbiosis and cophylogeny. Nat Commun [Internet]. 2018;9(1):1–13. Available from: http://dx.doi.org/10.1038/s41467-018-07275-x

9. Huggett MJ, Apprill A. Coral microbiome database : Integration of sequences reveals high diversity and relatedness of coral- associated microbes. Environ Microbiol Rep. 2019 Jun 1;11(3):372–85.

10. Puntin G, Sweet M, Fraune S, Medina M, Sharp K, Weis VM, et al. Harnessing the Power of Model Organisms To Unravel Microbial Functions in the Coral Holobiont. Microbiol Mol Biol Rev [Internet]. 2022 Oct 26 [cited 2023 Feb 24];86(4). Available from: https://journals.asm.org/doi/10.1128/mmbr.00053-22

11. Ohdera AH, Abrams MJ, Ames CL, Baker DM, Suescún-Bolívar LP, Collins AG, et al. Upside-Down but Headed in the Right Direction: Review of the Highly Versatile Cassiopea xamachana System. Front Ecol Evol. 2018 Apr 9;6:1–15.

12. Mammone M, Ferrier-Pages C, Lavorano S, Rizzo L, Piraino S, Rossi S. High photosynthetic plasticity may reinforce invasiveness of upside-down zooxanthellate jellyfish in Mediterranean coastal waters. PLoS One [Internet]. 2021 Mar 1 [cited 2021 Jun 30];16(3 March):e0248814. Available from: https://doi.org/10.1371/journal.pone.0248814

13. Béziat P, Kunzmann A. Under pressure: Cassiopea andromeda jellyfish exposed to increasing water temperature or lead, cadmium and anthropogenic gadolinium contamination. Mar Biol Res [Internet]. 2022;18(1–2):48–63. Available from: https://doi.org/10.1080/17451000.2022.2066132

14. McGill CJ, Pomory CM. Effects of bleaching and nutrient supplementation on wet weight in the jellyfish Cassiopea xamachana (Bigelow) (Cnidaria: Scyphozoa). Mar Freshw Behav Physiol. 2008;41(3):179–89.

15. Ames CL, Klompen AML, Badhiwala K, Muffett K, Reft AJ, Kumar M, et al. Cassiosomes are stinging-cell structures in the mucus of the upside-down jellyfish Cassiopea xamachana. Commun Biol [Internet]. 2020;3(1):1–15. Available from:

http://dx.doi.org/10.1038/s42003-020-0777-8

16. Bakshani CR, Morales-Garcia AL, Althaus M, Wilcox MD, Pearson JP, Bythell JC, et al. Evolutionary conservation of the antimicrobial function of mucus: A first defence against infection. npj Biofilms Microbiomes [Internet]. 2018;4(1):1–12. Available from: http://dx.doi.org/10.1038/s41522-018-0057-2

17. Klein SG, Pitt KA, Lucas CH, Hung S, Schmidt-roach S, Aranda M, et al. Night-Time Temperature Reprieves Enhance the Thermal Tolerance of a Symbiotic Cnidarian. 2019;6(July):1–16.

18. Lee MD, Kling JD, Araya R, Ceh J. Jellyfish Life Stages Shape Associated Microbial Communities, While a Core Microbiome Is Maintained Across All. Front Microbiol. 2018 Jul 12;0(JUL):1534.

19. Röthig T, Puntin G, Wong JCY, Burian A, McLeod W, Baker DM. Holobiont nitrogen control and its potential for eutrophication resistance in an obligate photosymbiotic jellyfish. Microbiome. 2021;9(1):1–16.

20. Cleary DFR, Becking LE, Polónia ARM, Freitas RM, Gomes NCM. Jellyfish-associated bacterial communities and bacterioplankton in Indonesian Marine lakes. FEMS Microbiol Ecol. 2016;92(5):1–14.

21. Hao W, Gerdts G, Holst S, Wichels A. Bacterial communities associated with scyphomedusae at Helgoland Roads. Mar Biodivers. 2019;49(3):1489–503.

22. Peng S, Hao W, Li Y, Wang L, Sun T, Zhao J, et al. Bacterial Communities Associated With Four Blooming Scyphozoan Jellyfish: Potential Species-Specific Consequences for

Marine Organisms and Humans Health. Front Microbiol. 2021 May 5;12(May):1–16.

23. Daley MC, Urban-Rich J, Moisander PH. Bacterial associations with the hydromedusa Nemopsis bachei and scyphomedusa Aurelia aurita from the North Atlantic Ocean. Mar Biol Res. 2016;12(10):1088–100.

24. Weiland-Bräuer N, Neulinger SC, Pinnow N, Künzel S, Baines JF, Schmitz RA. Composition of bacterial communities associated with Aurelia aurita changes with compartment, life stage, and population. Appl Environ Microbiol. 2015;81(17):6038–52.

25. Viver T, Orellana LH, Hatt JK, Urdiain M, Díaz S, Richter M, et al. The low diverse gastric microbiome of the jellyfish Cotylorhiza tuberculata is dominated by four novel taxa. Environ Microbiol. 2017;19(8):3039–58.

26. Basso L, Rizzo L, Marzano M, Intranuovo M, Fosso B, Pesole G, et al. Jellyfish summer outbreaks as bacterial vectors and potential hazards for marine animals and humans health? The case of Rhizostoma pulmo (Scyphozoa, Cnidaria). Sci Total Environ [Internet]. 2019;692:305–18. Available from: https://doi.org/10.1016/j.scitotenv.2019.07.155

27. Stabili L, Rizzo L, Basso L, Marzano M, Fosso B, Pesole G, et al. The microbial community associated with Rhizostoma pulmo: Ecological significance and potential consequences for marine organisms and human health. Mar Drugs. 2020;18(9).

28. Delannoy CMJ, Houghton JDR, Fleming NEC, Ferguson HW. Mauve Stingers (Pelagia noctiluca) as carriers of the bacterial fish pathogen Tenacibaculum maritimum. Aquaculture [Internet]. 2011;311(1–4):255–7. Available from: http://dx.doi.org/10.1016/j.aquaculture.2010.11.033

29. Muffett K, Miglietta MP. Demystifying Cassiopea species identity in the Florida Keys: Cassiopea xamachana and Cassiopea andromeda coexist in shallow waters. PLoS One [Internet]. 2023;18(3):e0283441. Available from: http://dx.doi.org/10.1371/journal.pone.0283441

30. Laramie MB, Pilliod DS, Goldberg CS, Strickler KM. Environmental DNA Sampling Protocol — Filtering Water to Capture DNA from Aquatic Organisms. In: US Geological Survey Techniques and Methods, book 2. Reston, VA: U.S. Geological Survey; 2015. p. 1–15.

31. Pavlovska M, Prekrasna I, Parnikoza I, Dykyi E. Soil sample preservation strategy affects the microbial community structure. Microbes Environ. 2021;36(1):1–7.

32. Callahan BJ, McMurdie PJ, Rosen MJ, Han AW, Johnson AJA, Holmes SP. DADA2: High-resolution sample inference from Illumina amplicon data. Nat Methods. 2016;13(7):581–3.

33. R-Core-Team (Foundation for Statistical Computing). R: A Language and Environment for Statistical Computing [Internet]. 2022. Available from: https://www.r-project.org/

34. McMurdie PJ, Holmes S. Phyloseq: An R Package for Reproducible Interactive Analysis and Graphics of Microbiome Census Data. PLoS One. 2013;8(4).

35. Davis NM, Proctor DiM, Holmes SP, Relman DA, Callahan BJ. Simple statistical identification and removal of contaminant sequences in marker-gene and metagenomics data. Microbiome. 2018;6(1):1–14.

36. Oksanen J, Simpson G, Blanchet F, Kindt R, Legendre P, Minchin P, et al. _vegan:

Community Ecology Package_. 2024.

37. Paradis E, Schliep K. ape 5 . 0 : an environment for modern phylogenetics and evolutionary analyses in R. 2019;35(July 2018):526–8.

38. Mikryukov V. metagMisc: Miscellaneous functions for metagenomic analysis [Internet]. Available from: https://github.com/vmikk/metagMisc

39. Taudiere A. MiscMetabar: Miscellaneous Functions for Metabarcoding Analysis [Internet]. 2024. Available from: https://cran.r-project.org/package=MiscMetabar

40. Tsirogiannis C, Sandel B. PhyloMeasures: Fast and Exact Algorithms for Computing Phylogenetic Biodiversity Measures. 2017.

41. Schliep KP. phangorn : phylogenetic analysis in R. 2011;27(4):592–3.

42. Battaglia T. btools: A suite of R function for all types of microbial diversity analyses. 2025.

43. Patil I. Visualizations with statistical details: The “ggstatsplot” approach. J Open Source Softw [Internet]. 2021;6(61)(3167). Available from: doi:10.21105/joss.03167

44. Martinez Arbizu P. pairwiseAdonis: Pairwise Multilevel Comparison using Adonis [Internet]. 2017. Available from: https://github.com/pmartinezarbizu/pairwiseAdonis

45. Kassambara A. rstatix: Pipe-Friendly Framework for Basic Statistical Tests [Internet]. 2023. Available from: https://cran.r-project.org/package=rstatix

46. McLaren M. speedyseq: Faster implementations of phyloseq functions [Internet]. 2025. Available from: https://github.com/mikemc/speedyseq

47. Pebesma E, Bivand R. Spatial Data Science: With applications in R [Internet]. London: Chapman and Hall/CRC; 2023. 352 p. Available from: https://r-spatial.org/book/

48. Macvittie S, Doroodian S, Alberto A, Sogin M, Macvittie S, Doroodian S, et al. Exaiptasia diaphana , following antibiotic exposure. 2024;9(6).

49. Fernandes AD, Reid JNS, Macklaim JM, Mcmurrough TA, Edgell DR, Gloor GB. Unifying the analysis of high-throughput sequencing datasets : characterizing RNA-seq , 16S rRNA gene sequencing and selective growth experiments by compositional data analysis. 2014;1–13.

50. Holmes I, Harris K, Quince C. Dirichlet Multinomial Mixtures : Generative Models for Microbial Metagenomics. 2012;7(2).

51. Morgan M. DirichletMultinomial: Dirichlet-Multinomial Mixture Model Machine Learning for Microbiome Data [Internet]. 2024. Available from: https://doi.org/10.18129/B9.bioc.DirichletMultinomial

52. Lahti L, Shetty S. microbiome R package. 2019.

53. Neu AT. Defining and quantifying the core microbiome : Challenges and prospects. 2021;118(51):1–10.

this reference needs correction for authors (Nue, Allen & Roy), and is missing journal name (PNAS)

54. Bonacolta AM, Connelly MT, Rosales SM, del Campo J, Traylor-knowles N. The starlet sea anemone , Nematostella vectensis , possesses body region-specific bacterial associations with spirochetes dominating the capitulum. FEMS Microbiol Lett. 2021;(August 2020):1–9.

55. van Oppen MJH, Blackall LL. Coral microbiome dynamics, functions and design in a

changing world. Nat Rev Microbiol [Internet]. 2019;17(9):557–67. Available from: http://dx.doi.org/10.1038/s41579-019-0223-4

56. Agostini S, Yoshimi S, Higuchi T, Casareto BE. Biological and chemical characteristics of the coral gastric cavity. Coral Reefs. 2011;(March).

57. Bollati E, Hughes DJ, Suggett DJ, Raina J-B, Kühl M. Microscale sampling of the coral gastric cavity reveals a gut-like microbial community. bioRxiv [Internet]. 2024;2024.05.20.594925. Available from: https://www.biorxiv.org/content/10.1101/2024.05.20.594925v1%0Ahttps://www.biorxiv.org/content/10.1101/2024.05.20.594925v1.abstract

58. Carabantes N, Cerqueda-García D, García-Maldonado JQ, Thomé PE. Changes in the Bacterial Community Associated With Experimental Symbiont Loss in the Mucus Layer of Cassiopea xamachana Jellyfish. Front Mar Sci. 2022;9(May):1–13.

59. Wu W, Lei J-N, Mao Q, Tian Y-Z, Shan H-W, Chen J-P. Distribution, Vertical Transmission, and Cooperative Mechanisms of Obligate Symbiotic Bacteria in the Leafhopper Maiestas dorsalis (Hemiptera, Cicadellidea). Insects. 2023;1–13.

60. Sharp KH, Pratte ZA, Kerwin AH, Rotjan RD, Stewart FJ. Season, but not symbiont state, drives microbiome structure in the temperate coral Astrangia poculata. Microbiome. 2017;5(1):120.

61. Hammer TJ, Sanders JG, Fierer N. Not all animals need a microbiome. FEMS Microbiol Lett. 2019;366(10):1–11.

62. Riviere M La, Roumagnac M, Garrabou J, Bally M. Transient Shifts in Bacterial

Communities Associated with the Temperate Gorgonian Paramuricea clavata in the Northwestern Mediterranean Sea. PLoS One. 2013;8(2).

63. Neave MJ, Apprill A, Ferrier-Pagès C, Voolstra CR. Diversity and function of prevalent symbiotic marine bacteria in the genus Endozoicomonas. Appl Microbiol Biotechnol [Internet]. 2016;100(19):8315–24. Available from: http://dx.doi.org/10.1007/s00253-016-7777-0

64. Tinta T, Kogovšek T, Klun K, Malej A, Herndl GJ, Turk V. Jellyfish-associated microbiome in the marine environment: Exploring its biotechnological potential. Mar Drugs. 2019;17(2):1–33.

65. Maher RL, Schmeltzer ER, Meiling S, McMinds R, Ezzat L, Shantz AA, et al. Coral Microbiomes Demonstrate Flexibility and Resilience Through a Reduction in Community Diversity Following a Thermal Stress Event. Front Ecol Evol. 2020;8(October):1–15.

66. Pogoreutz C, Oakley CA, Rädecker N, Cárdenas A, Perna G, Xiang N, et al. Coral holobiont cues prime Endozoicomonas for a symbiotic lifestyle. ISME J. 2022;16(8):1883–95.

67. Neave MJ, Michell CT, Apprill A, Voolstra CR. Endozoicomonas genomes reveal functional adaptation and plasticity in bacterial strains symbiotically associated with diverse marine hosts. Sci Rep. 2017;7(December 2016):1–12.

68. McDevitt-Irwin JM, Baum JK, Garren M, Vega Thurber RL. Responses of coral-associated bacterial communities to local and global stressors. Front Mar Sci. 2017;4(AUG):1–16.

69. Neulinger SC, Gärtner A, Järnegren J, Ludvigsen M, Lochte K, Dullo WC. Tissue-associated "Candidatus mycoplasma corallicola" and filamentous bacteria on the cold-water coral Lophelia pertusa (Scleractinia). Appl Environ Microbiol. 2009;75(5):1437–44.

70. Sweet MJ, Bulling MT. On the importance of the microbiome and pathobiome in coral health and disease. Front Mar Sci. 2017;4(January):1–11.

71. Hofmann DK, Neumann R, Henne K. Strobilation, budding and initiation of scyphistoma morphogenesis in the rhizostome Cassiopea andromeda (Cnidaria: Scyphozoa). Mar Biol. 1978;47(2):161–76.

72. Neumann R. Bacterial Induction of Settlement and Metamorphosis in the Planula Larvae of Cassiopea andromeda (Cnidaria: Scyphozoa, Rhizostomeae). Vol. 1. 1979.

73. Kramar MK, Tinta T, Lučić D, Malej A, Turk V. Bacteria associated with moon jellyfish during bloom and post-bloom periods in the Gulf of Trieste (northern Adriatic). PLoS One. 2019;14(1):1–21.

74. Tinta T, Kogovsek T, Malej A, Turk V. Jellyfish Modulate Bacterial Dynamic and Community Structure. PLoS One. 2012;7(4):1–10.

75. Pollock FJ, Wilson B, Johnson WR, Morris PJ, Willis BL, Bourne DG. Phylogeny of the coral pathogen Vibrio coralliilyticus. Environ Microbiol Rep. 2010;2(1):172–8.

76. Ben-Haim Y, Thompson FL, Thompson CC, Cnockaert MC, Hoste B, Swings J, et al. Vibrio coralliilyticus sp. nov., a temperature-dependent pathogen of the coral Pocillopora damicornis. Int J Syst Evol Microbiol. 2003;53(1):309–15.

77. Richards GP, Watson MA, Needleman DS, Church KM, Häse CC. Mortalities of Eastern

And Pacific oyster larvae caused by the pathogens Vibrio coralliilyticus and Vibrio tubiashii. Appl Environ Microbiol. 2015;81(1):292–7.

78. Emery MA, Beavers KM, Van Buren EW, Batiste R, Dimos B, Pellegrino MW, et al. Trade-off between photosymbiosis and innate immunity influences cnidarian's response to pathogenic bacteria. Proc R Soc B Biol Sci. 2024;291(2032):20240428.

79. Thomé PE, Rivera-Ortega J. Specific antibacterial activity against potential pathogens and re-straining of larvae settlement from a pigmented Pseudoalteromonas strain isolated from the jellyfish Cassiopea xamachana. Int Aquat Res. 2022;14(2):95–105.

80. Bright M, Espada-hinojosa S, Lagkouvardos I, Volland J, Hole W. The giant ciliate Zoothamnium niveum and its thiotrophic epibiont Candidatus Thiobios zoothamnicoli : a model system to study interspecies cooperation. 2014;5(April):1–13.

81. Rinke C, Schmitz-esser S, Stoecker K, Nussbaumer AD, Vanura K, Wagner M, et al. " Candidatus Thiobios zoothamnicoli ," an Ectosymbiotic Bacterium Covering the Giant Marine Ciliate Zoothamnium niveum. 2021;72(3):2014–21.

82. Vekeman B. Methanotrophic microbiomes from North Sea sediment.

83. Song J, Hwang J, Kang I, Cho JC. OPEN A sulfate - reducing bacterial genus , Desulfosediminicola gen . nov ., comprising two novel species cultivated from tidal - flat sediments. Sci Rep [Internet]. 2021;1–13. Available from: https://doi.org/10.1038/s41598-021-99469-5

84. Engelen AH, Aires T, Vermeij MJA, Herndl GJ, Serrão EA, Frade PR. Host differentiation and compartmentalization of microbial communities in the azooxanthellate

cupcorals Tubastrea coccinea and Rhizopsammia goesi in the Caribbean. Front Mar Sci. 2018;9(NOV):1–9.

85. Meirelles LA, Newman DK. Both toxic and beneficial effects of pyocyanin contribute to the lifecycle of Pseudomonas aeruginosa. Mol Microbiol. 2018;110(6):995–1010.

86. Carlson CA, Ingraham JL. Comparison of Denitrification by Pseudomonas stutzeri , Pseudomonas aeruginosa , and Paracoccus denitrificans. 1983;45(4):1247–53.

87. Huo Y, Xu X, Li X, Liu C, Cui H, Wang C, et al. Ruegeria marina sp . nov ., isolated from Marine Sediment. 2011;347–50.

88. Klein SG, Pitt KA, Carroll AR. Reduced salinity increases susceptibility of zooxanthellate jellyfish to herbicide toxicity during a simulated rainfall event. Environ Pollut [Internet]. 2016;209:79–86. Available from: http://dx.doi.org/10.1016/j.envpol.2015.11.012

89. Hyun DW, Shin NR, Kim MS, Oh SJ, Kim PS, Whon TW, et al. Endozoicomonas atrinae sp. nov., isolated from the intestine of a comb pen shell Atrina pectinata. Int J Syst Evol Microbiol. 2014;64(PART 7):2312–8.

90. Sunagawa S, Woodley CM, Medina M. Threatened corals provide underexplored microbial habitats. PLoS One. 2010;5(3):1–7.

Supplementary Information

Supplement A1: Full length 16S rRNA gene identities

Three samples were sent to CD Genomics for full length 16S on PacBio Sequel using universal 16S primers (F: AGRGTTTGATYNTGGCTCAG;

R:TASGGHTACCTTGTTASGACTT). Samples sent for full length 16S rRNA sequencing included gastrovascular cavity swab extractions from medusae Marathon Key GVC 30 and Key West GVC 96 and GVC 120. As the usable reads returned from this were low (597-12520 per sample), these full-length identities are not discussed in the main text. For further details, please refer to the appendix (A1).

Full length 16S rRNA gene sequences (BioProject: PRJNA1020446) were run on mothur v1.48.0 with deviation from the MiSeq SOP on trim length. ASV cutoff was set to five differences, and ASV representative sequences were compared against the NCBI GenBank (Sept 24, 2023) database using BLAST to find nearest matches by e-value.

In order to identify core microbial groups to a lower taxonomic level, three internal medusa gastrovascular swabs (medusae Kmuffet_96, Kmuffet_120 and Kmuffet_30) from Marathon Key (MK) and Key West (GB) were sequence for full length 16S rRNA gene (see S1 for location of samples). As the primers were not chloroplast exclusionary, the Key West sites produced few useable sequences (Kmuffet_96: 597 and Kmuffet_120: 1097). The Marathon Key medusa (Kmuffet_30), likely bleached at time of collection, returned 12,520 non-chloroplast sequences. The Mycoplasma-like full length 16S rRNA gene sequence (Genbank Acc. No. OR592270) from Marathon Key and Key West has no closely related sequences currently identified (greatest identity 88.43% to sequence HQ393440). The *Endozoicomonas* strain from both Marathon Key and Key West (Genbank Acc. No. OR592271) has 99.93% identity to *Endozoicomonas atrinae* strain WP70(T) collected from the intestine of South Korean *Atrina pectinata* (Accession NR_134024)(89) and 99.80% identity (<5 nucleotide differences) with isolates collected from *Gorgonia ventalina* (GenBank Acc. No. GU118345) in Bocas del Toro,

Panama (90). *Endozoicomanas* and *Mycoplasma*-like isolates were the only two groups to have ASVs with over 100 sequences.

S1 Tables: (in Supplementary Excel Workbook)

Supplementary Table S1: Roster of collected medusae

Supplementary Table S2: Read processing tracking table

Supplementary Table S3: Representative sequences of all ASVs from the dataset

Supplementary Table S4: ASVs orderd by abundance in each sample type

Supplementary Table S5: Taxonomy of top 1000 ASVs in dataset

Sfig 1: Rarefaction curves of all samples

Figure 1

Click here to access/download;Figure;KMM_CM_2025_Fig1.jpg

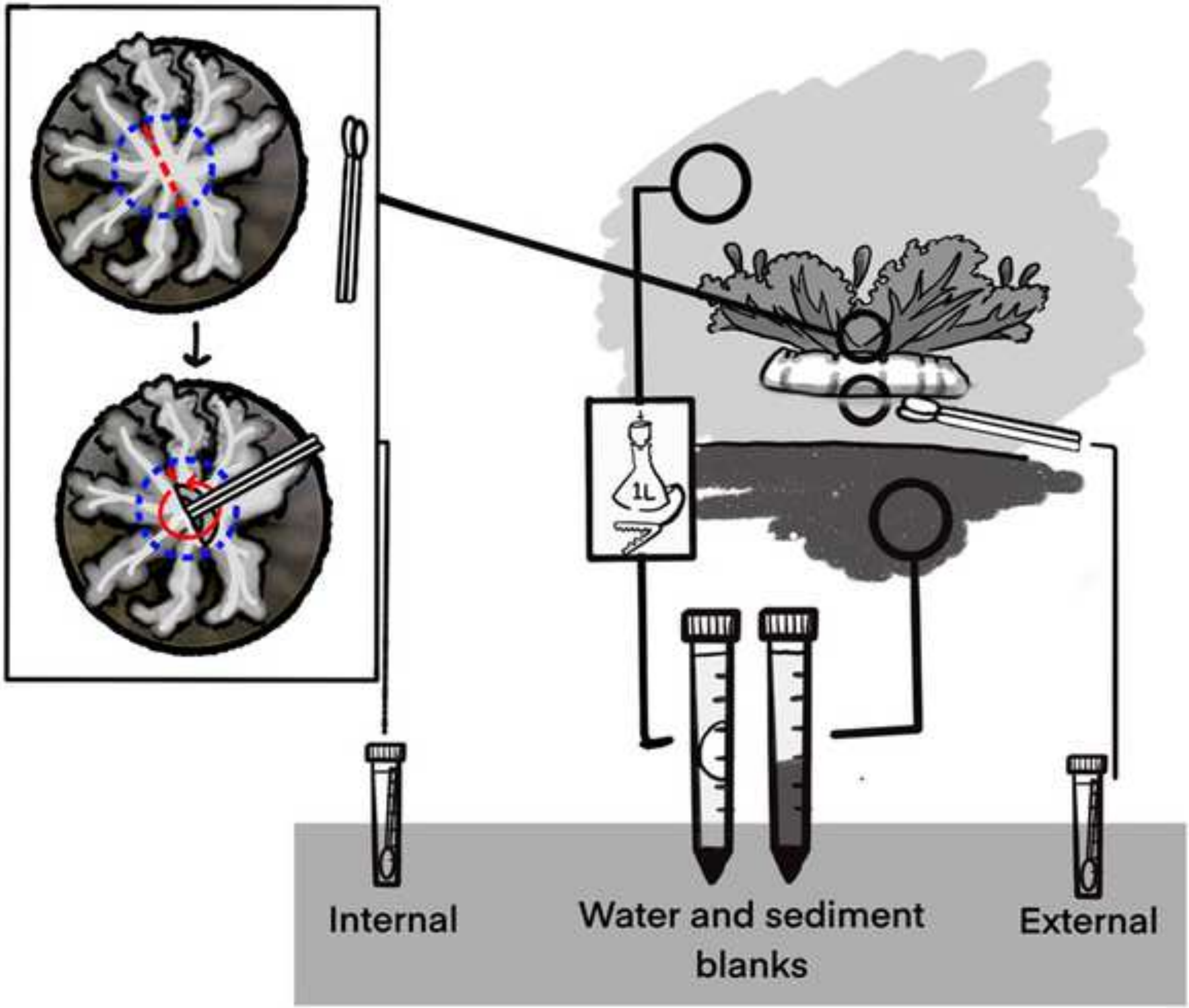

Figure 2

Click here to access/download;Figure;KMM_CM_2025_Fig2.tif

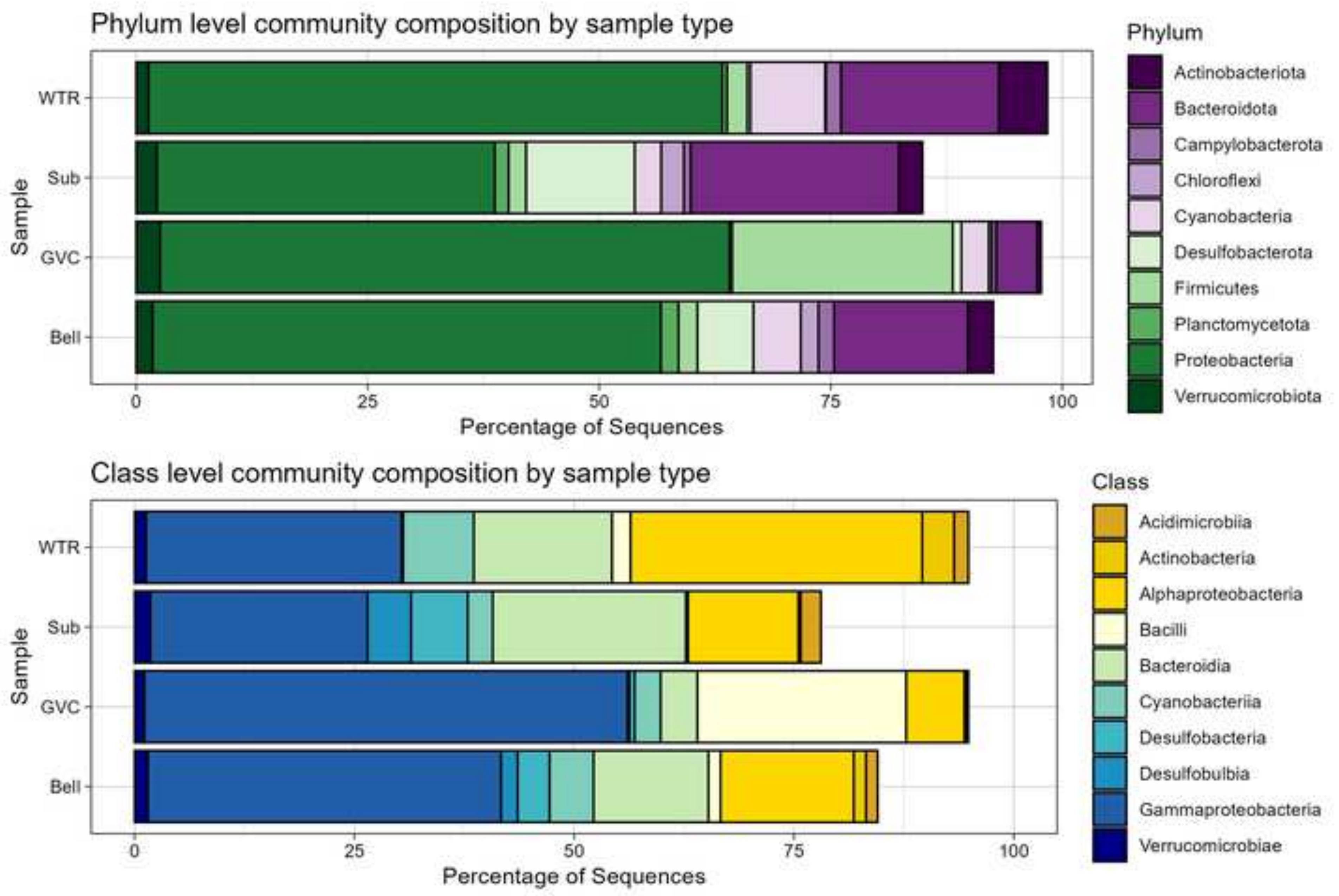

Figure 3

Click here to access/download;Figure;KMM_CM_2025_Fig3.tif

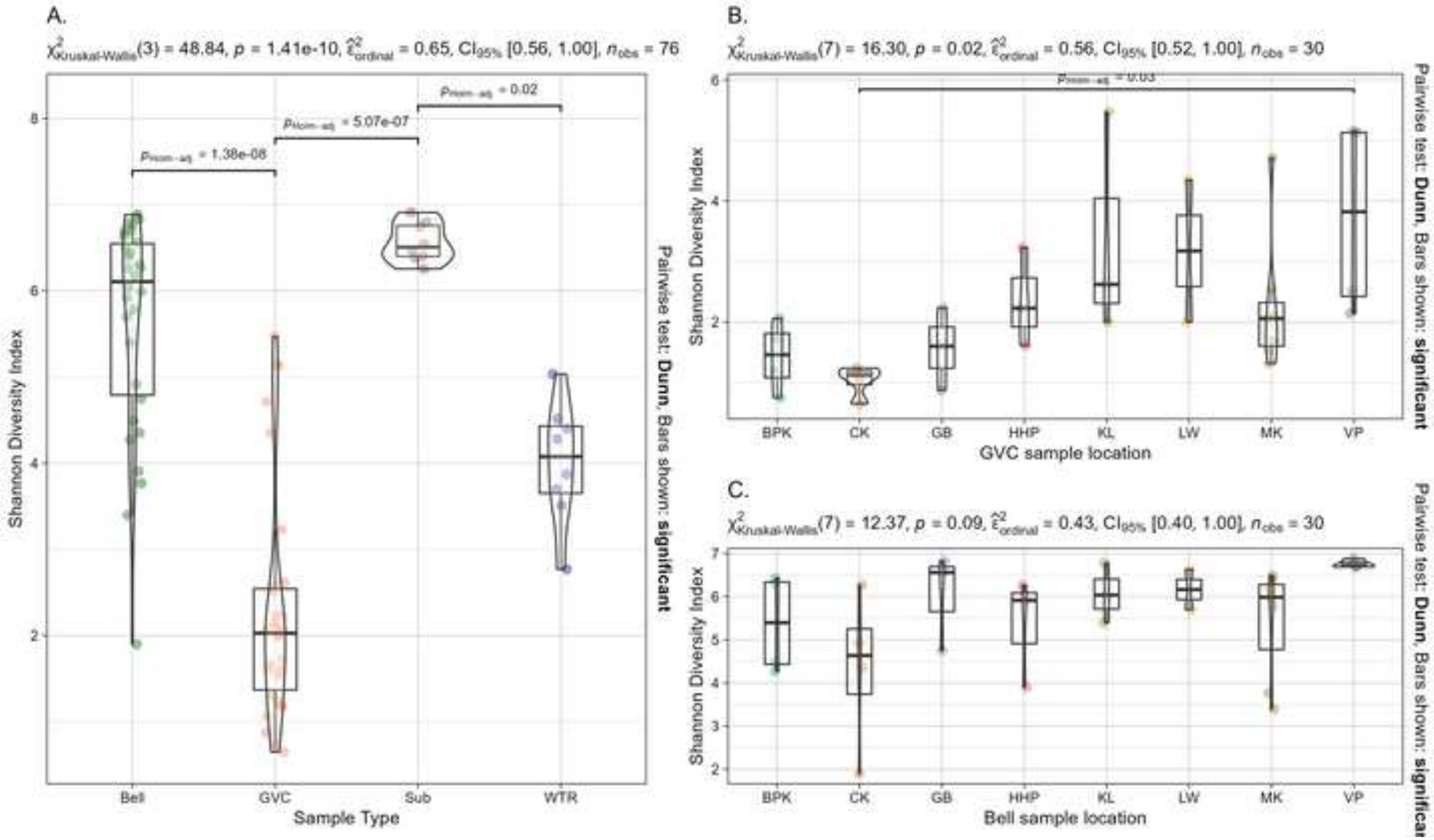

Figure 4

Click here to access/download;Figure;KMM_CM_2025_Fig4.tif

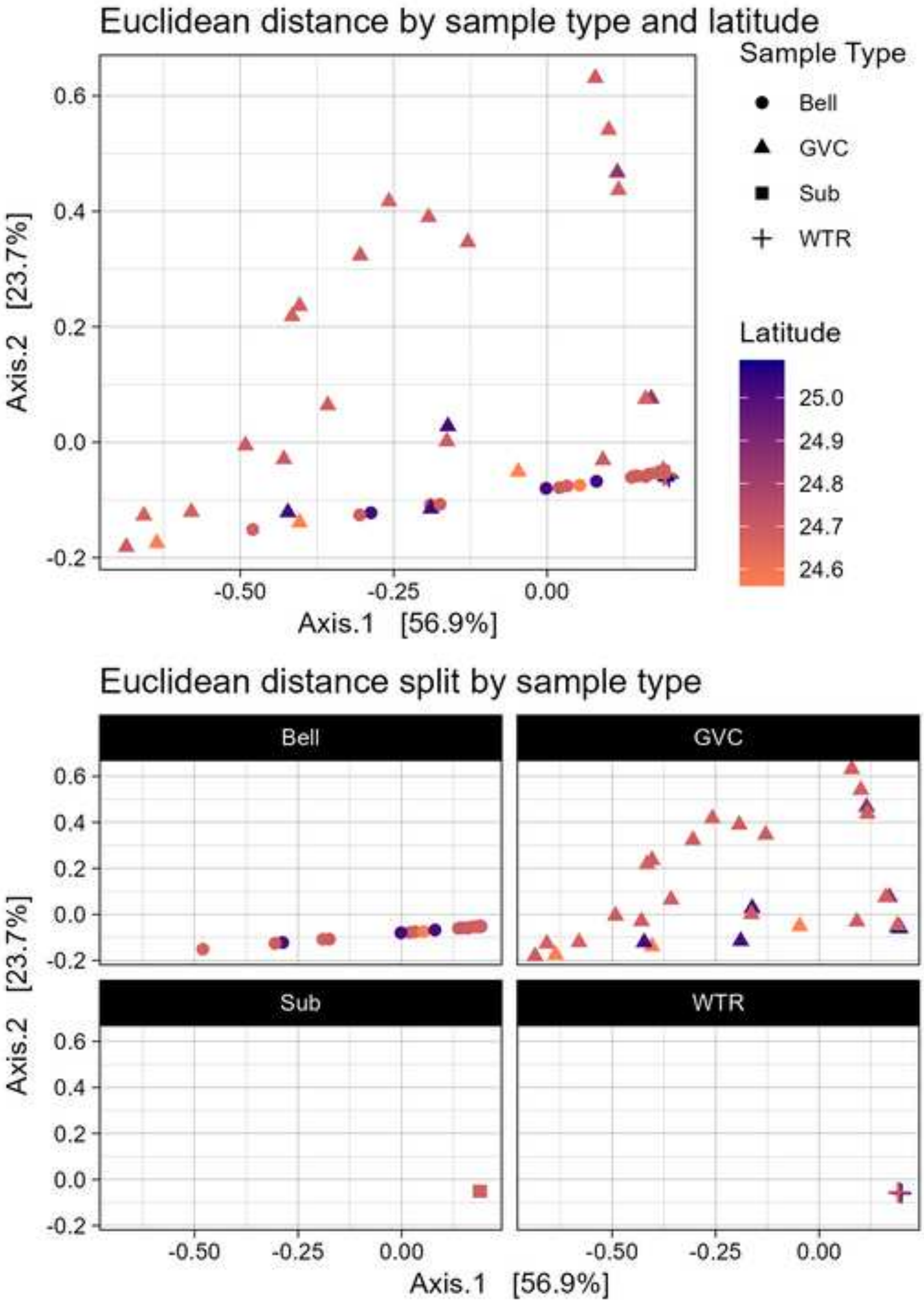

Figure 5

Click here to access/download;Figure;KMM_CM_2025_Fig5.tif

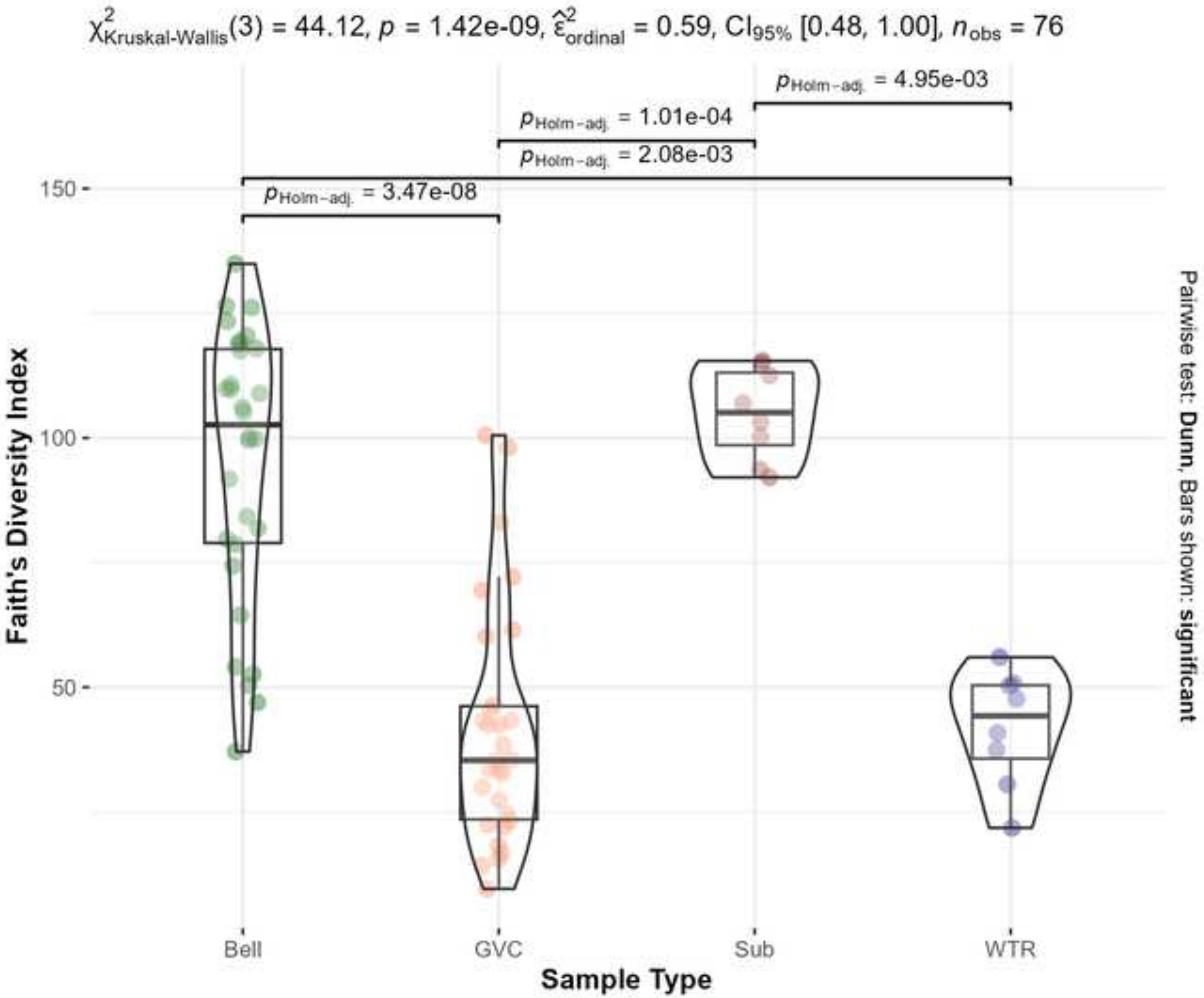

Figure 6

Click here to access/download;Figure;KMM_CM_2025_Fig6.tif

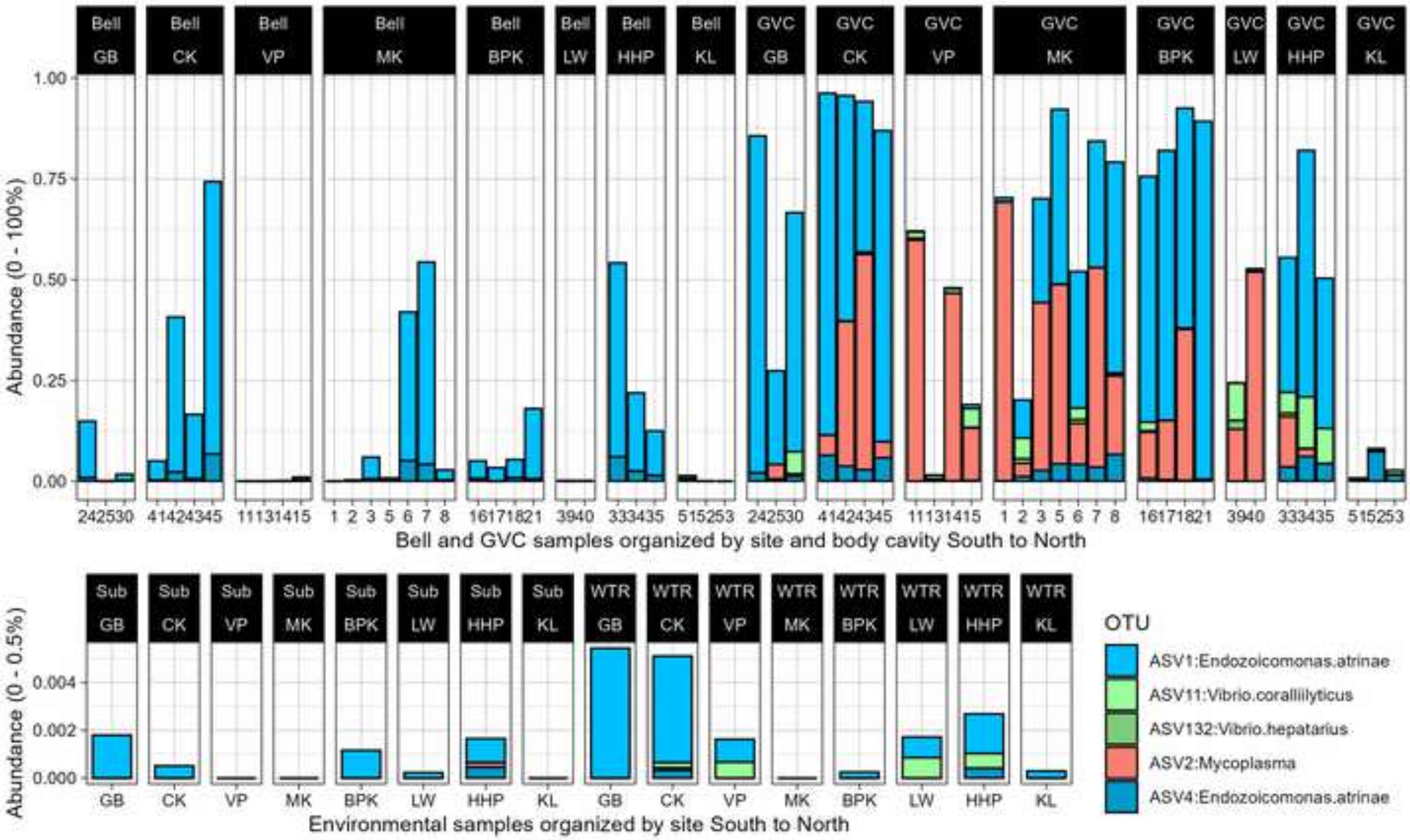

Figure 7

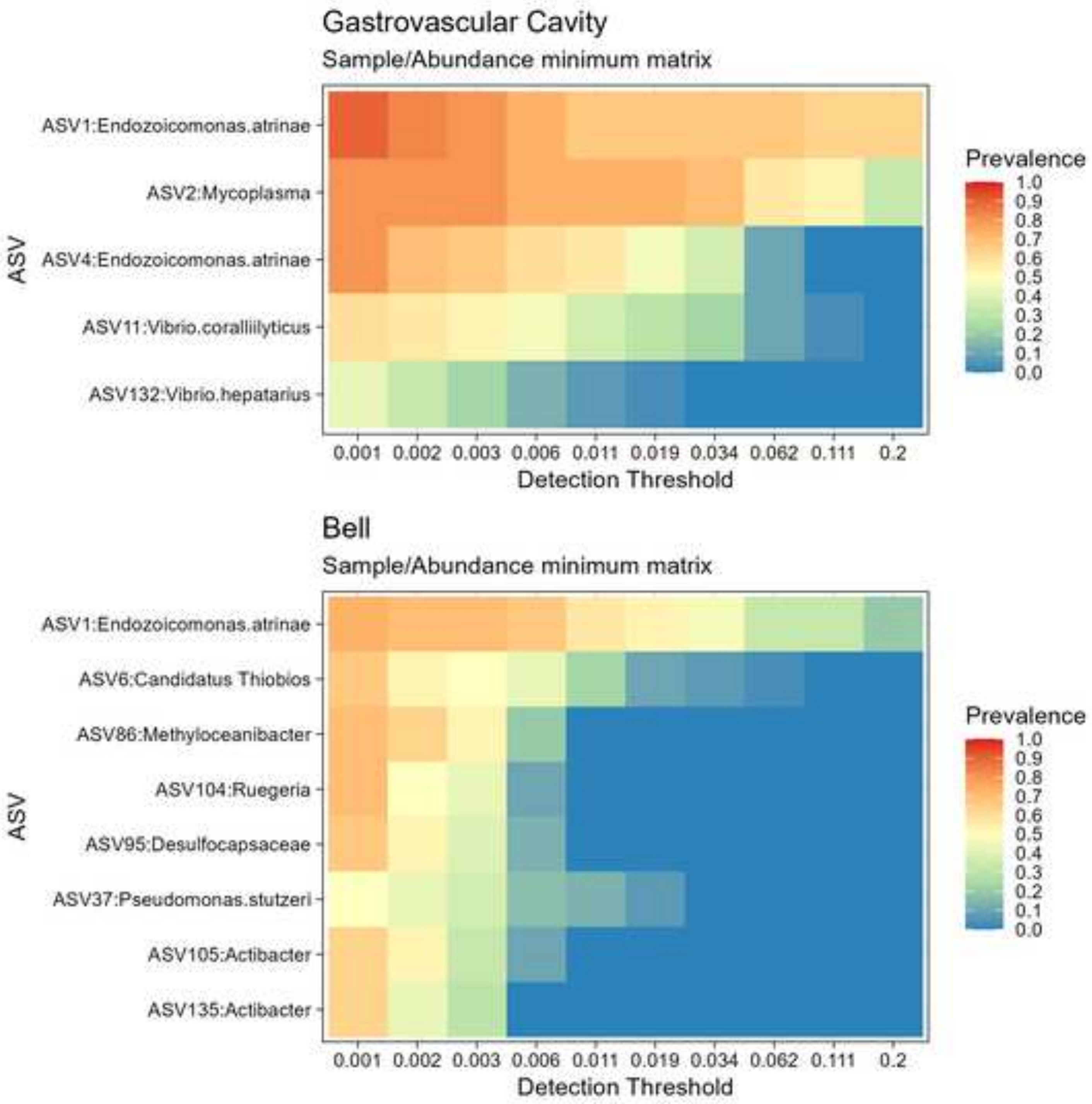
Gastrovascular Cavity
Sample/Abundance minimum matrix
ASV
ASV1:Endozoicomonas.atrinae
ASV2:Mycoplasma
ASV4:Endozoicomonas.atrinae
ASV11:Vibrio.coralliilyticus
ASV132:Vibrio.hepatarius
0.001 0.002 0.003 0.006 0.011 0.019 0.034 0.062 0.111 0.2
Detection Threshold
Prevalence
1.0 0.9 0.8 0.7 0.6 0.5 0.4 0.3 0.2 0.1 0.0
Bell
Sample/Abundance minimum matrix
ASV
ASV1:Endozoicomonas.atrinae
ASV6:Candidatus Thiobios
ASV86:Methyloceanibacter
ASV104:Ruegeria
ASV95:Desulfocapsaceae
ASV37:Pseudomonas.stutzeri
ASV105:Actibacter
ASV135:Actibacter
0.001 0.002 0.003 0.006 0.011 0.019 0.034 0.062 0.111 0.2
Detection Threshold
Prevalence
1.0 0.9 0.8 0.7 0.6 0.5 0.4 0.3 0.2 0.1 0.0

Figure 8

Click here to access/download;Figure;KMM_CM_2025_Fig8.tif

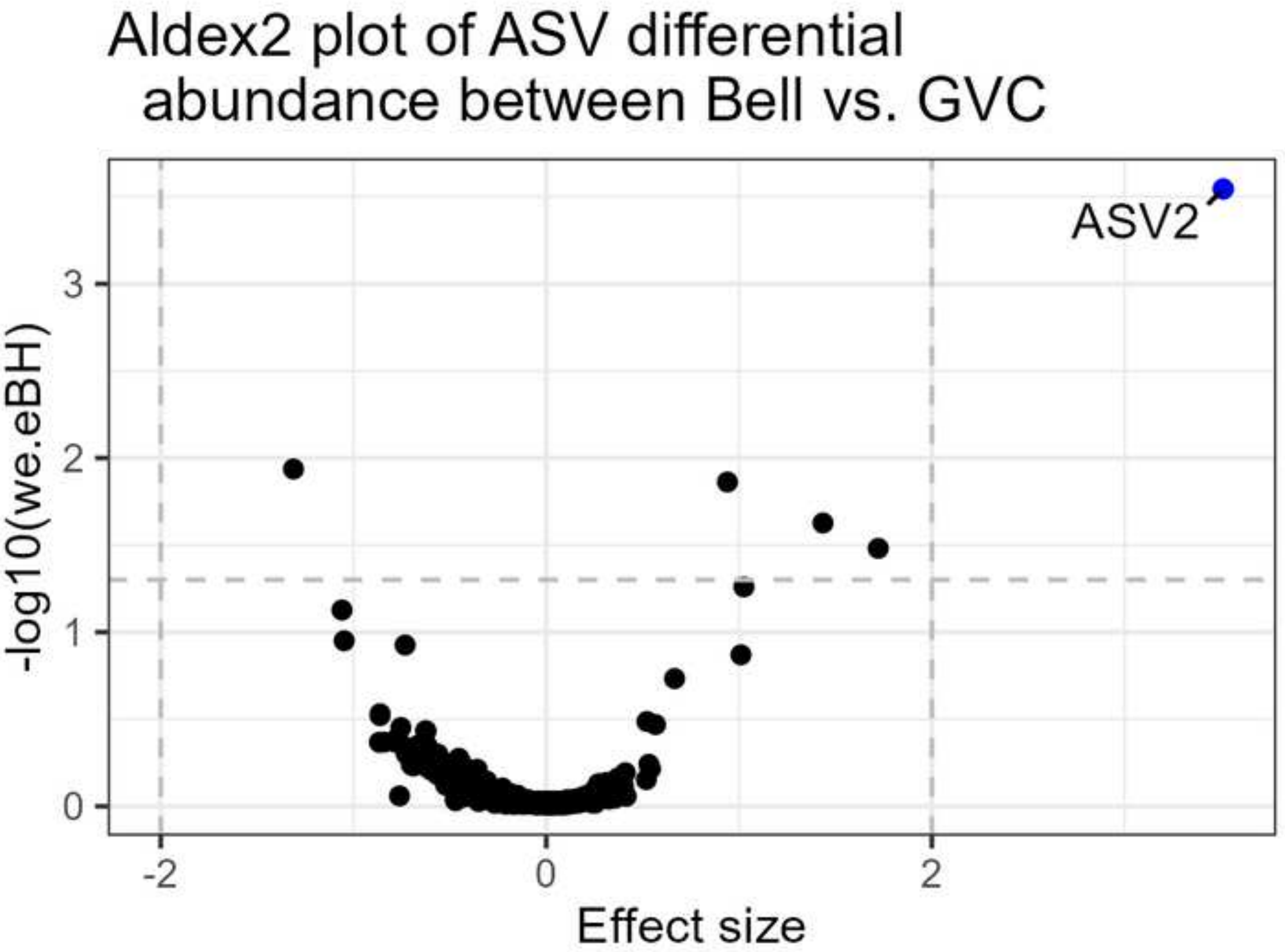

Figure 9

Click here to access/download;Figure;KMM_CM_2025_Fig9.tif

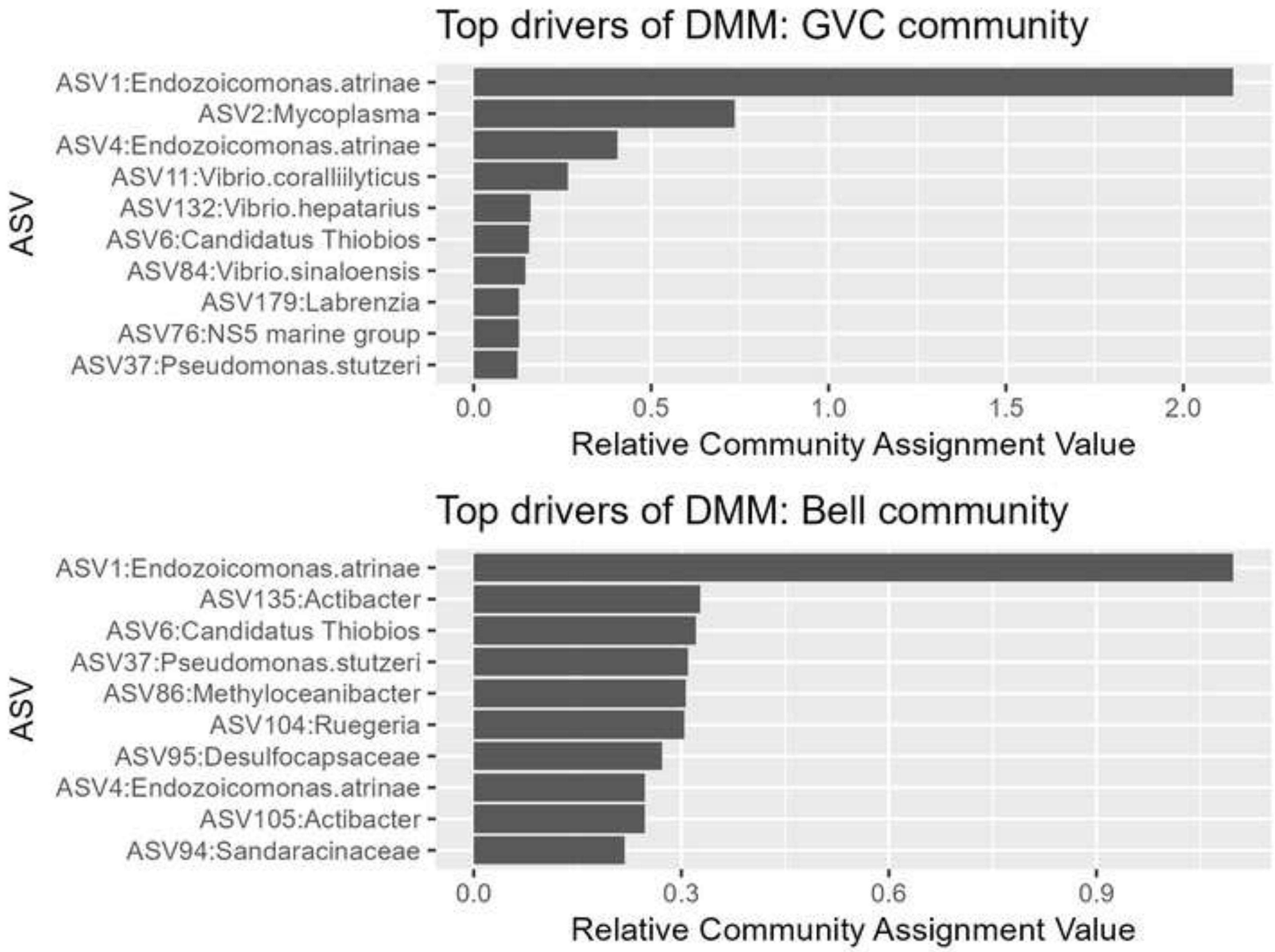

Figure 10 Click here to access/download;Figure;KMM_CM_2025_Fig10.tif

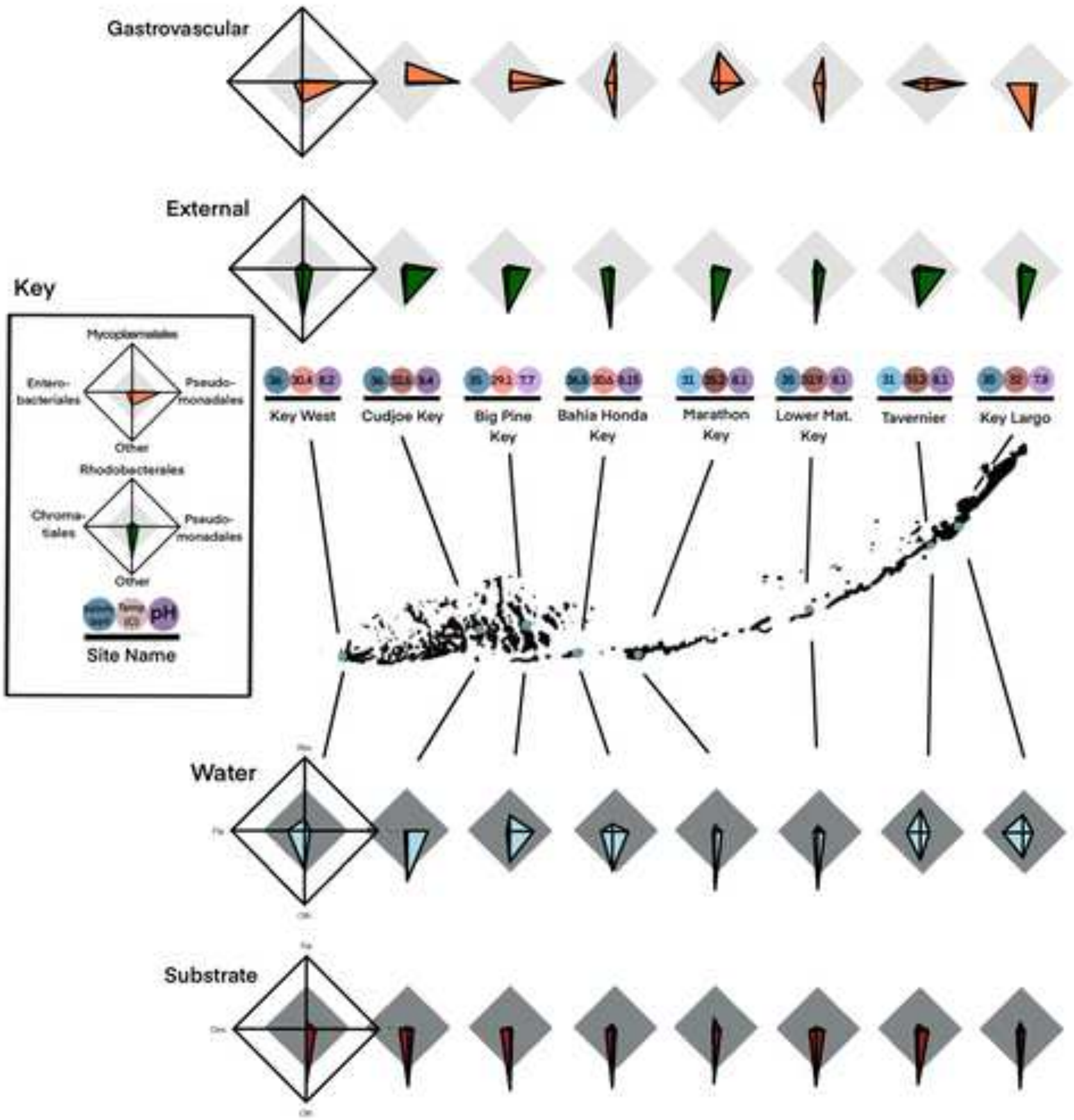

salinity, temp, pH

Figure 11

Click here to access/download;Figure;KMM_CM_2025_Fig11.tif

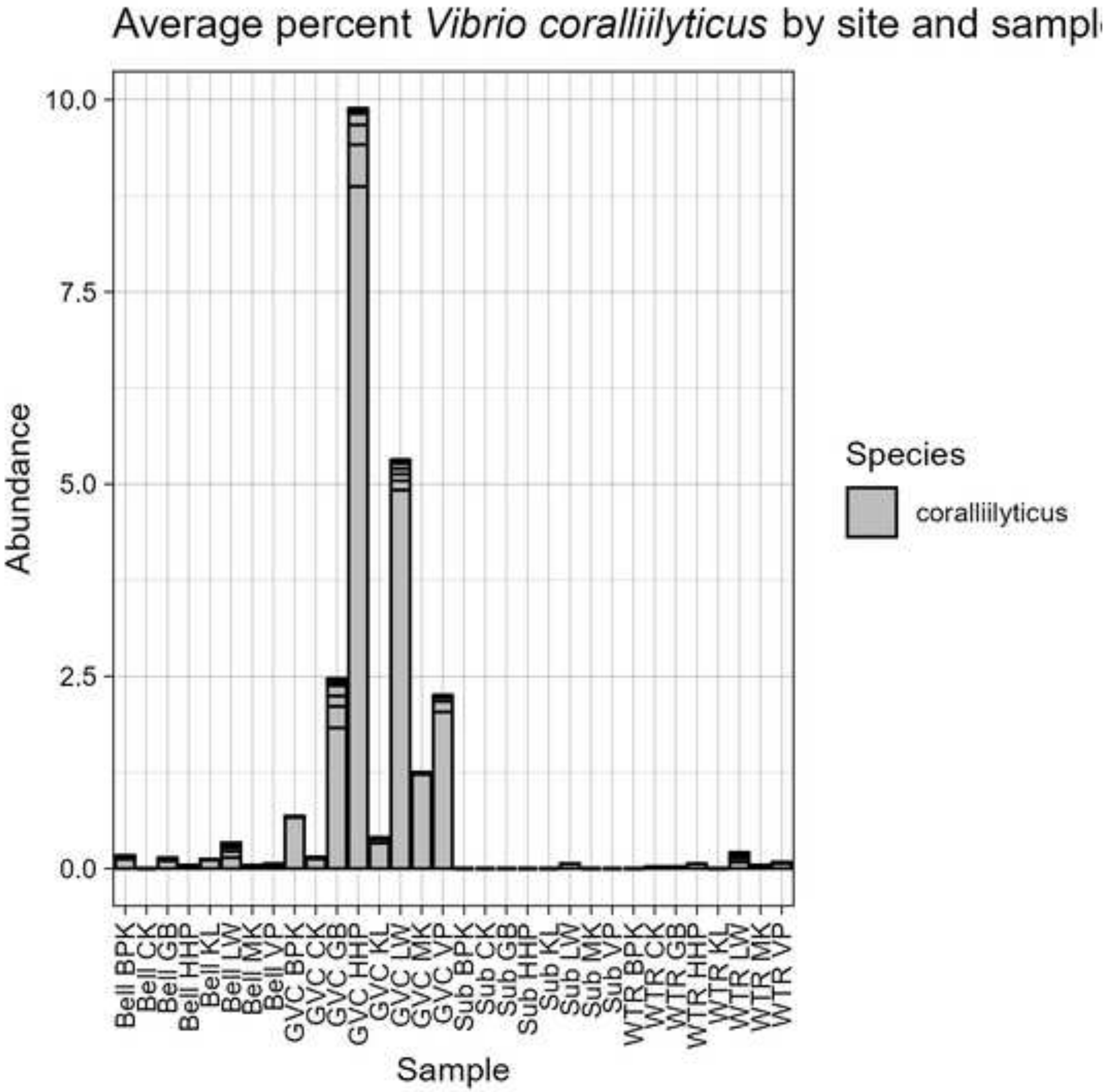

Figure 12

Click here to access/download;Figure;KMM_CM_2025_Fig12.tif

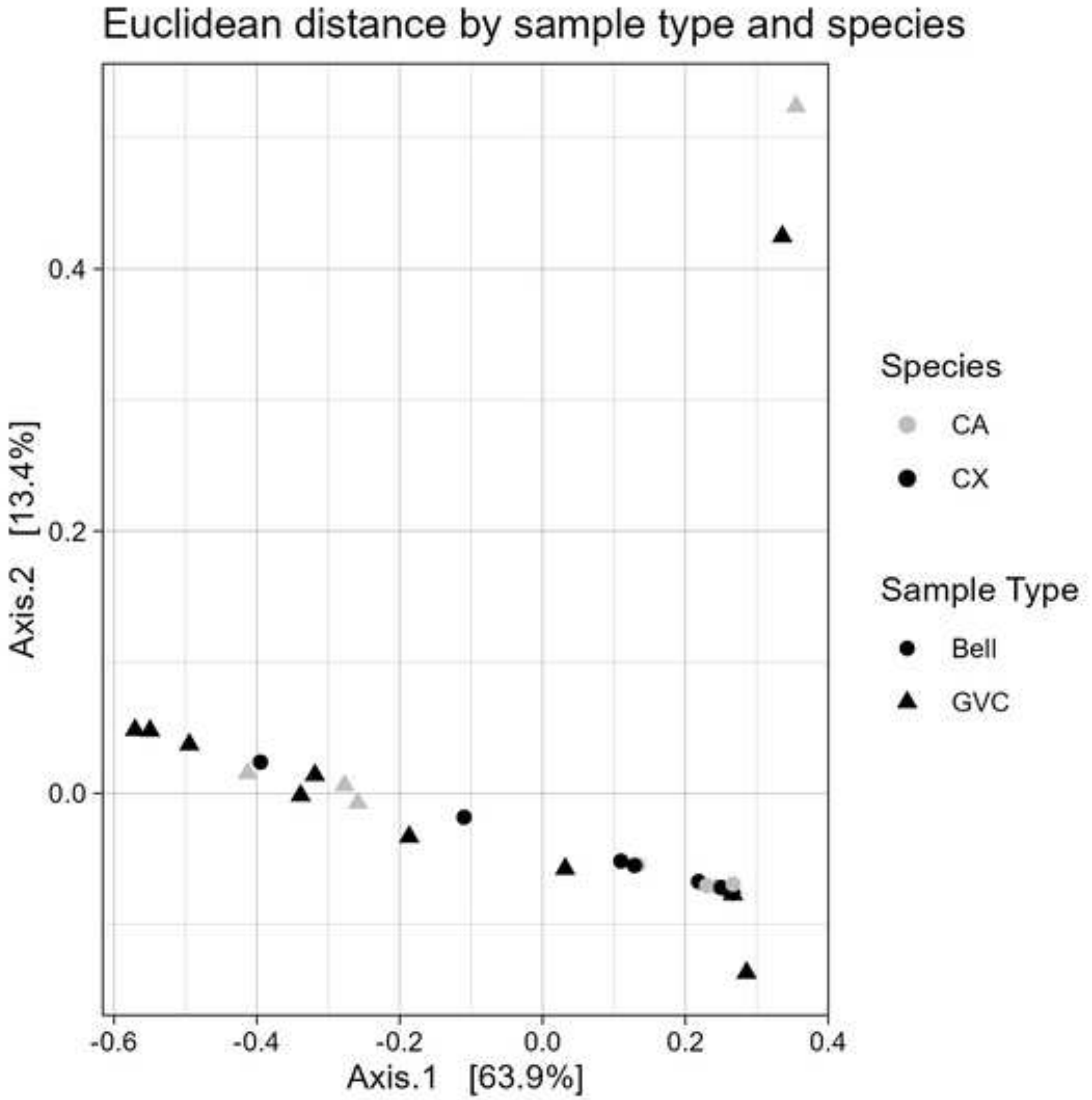

Supporting Information Fig S1

Click here to access/download
Supporting Information
species_rarefaction_plot_S1.png

Supporting Information Fig S2

Click here to access/download
Supporting Information
Bell_sub_aldex_dif_plot.png

Click here to access/download
Supporting Information
Supplementary_Material_Microbial_Community_25.1.19.xlsx

Revised Manuscript with Track Changes

Gastric microbiomes~~Bacterial communities~~ of *Cassiopea* in the Florida Keys are low diversity and ~~share major bacterial taxa with coral microbiomes~~ *Endozoicomonas-~~,~~, Vibrio-* and *Mycoplasma-dominated*

Formatted: Font: Italic
Formatted: Font: Not Italic
Formatted: Font: Italic

Kaden M Muffett[1]*, Jessica M Labontée~~Jessica Labonte~~[2], Maria Pia Miglietta[2]

1 University of California Merced, Merced, CA, 95343

2 Texas A&M University at Galveston, Galveston, TX, 77554

*Corresponding email: kmmuffett@gmail.com

Running Page Head: Bacteria of *Cassiopea* in the Florida Keys

Abstract~~:~~

Formatted: Font: 18 pt, Bold

Interactions with microbial communities fundamentally shape metazoans' physiology, development, and health across marine ecosystems. This is especially true in zooxanthellate (symbiotic algae-containing) cnidarians. In photosymbiotic anthozoans (eg. shallow water anemones and corals), the key members of the associated microbiota are increasingly well studied, ~~but~~ however there is limited data on photosymbiotic scyphozoans (true jellyfish). Using 16S rRNA barcoding, we sampled the internal and external mucus of the zooxanthellate Upside-Down Jellyfish, *Cassiopea xamachana* ~~and *C. andromeda*,~~ throughout eight sites covering the full length of the Florida Keys. We find ~~these~~ that across sites, these medusae to have low-diversity internal microbial communities distinct from the communities of their external surfaces and their environment. These internal communities are dominated by only three taxa: *Endozoicomonas* cf. *atrinae*~~*Endozoicomonadaceae*~~, ~~Mycoplasmataceae~~an uncultured novel *Mycoplasma*, and *Vibrio* cf. *coralliilyticus*~~Vibrionaceae~~. ~~*External*~~ *Cassiopea* bell mucosal

Formatted: Font: Italic
Formatted: Font: Italic
Formatted: Font: Italic
Formatted: Font: Not Italic
Formatted: Font: Italic

samples conform largely to the communities of surrounding sediment with the addition of *Endozoicomonas* cf. *atrinae*~~Endozoicomonadaceae~~. The microbial taxa we identify associated with wild Florida Keys *Cassiopea* bear a strong resemblance to those found within photosymbiotic anthozoans,~~;~~ increasing the known links in ecological position between these groups.

Key words: Cnidaria, microbiome, symbiosis, *Cassiopea*, Scyphozoa

~~1 INTRODUCTION~~Introduction

Formatted: Font: 18 pt, Bold

Cnidarian-photosymbiont interactions are central to ecosystem function in coral reefs and beyond. Algal photosymbionts, or zooxanthellae, are present in the well-known stony corals, octocorals, and anemones, but also play important roles in a variety of lesser-known jellyfishes, chief among them, jellyfish of the genus *Cassiopea* (1–4). For symbiotic anthozoans, microbial communities play a key role in maintaining health within this photosymbiont partnership, with community members directly conferring disease resistance and heat tolerance on hosts (5–7). These microbial communities are often specialized, with distinct corals demonstrating co-evolution with members of their bacterial communities (8), but retain several prevalent ~~clades~~genera, such as *Endozoicomonas~~adaceae~~*, across many hosts (9). While anthozoan communities are under active investigation, few analyses have documented the stability and nature of microbiome communities associated with zooxanthellate jellyfishes, a group with divergent life history traits from Anthozoa but with a ~~similar~~ comparable obligate symbiosis with dinoflagellates (~~s of the~~ clade Symbiodiniaceae).

Formatted: Font: Italic

Formatted: Font: Italic

The Upside-Down Jellyfish, *Cassiopea* spp., is a model organism for cnidarian-photosymbiont interactions (10,11). These medusae are residents of tropical and subtropical waters globally and can survive in water that is frequently shallow (as little as 8 cm depth) and hot (up to 33˚C for *C. andromeda*) (12,13). *Cassiopea* obligatorily hosts algal symbionts, losing mass even when well fed while in aposymbiotic conditions (14). As in corals and other scyphozoans, mucus plays a central role for *Cassiopea*, presenting a sticky interface between the ~~medusae~~ host and their benthic environment (15,16). In contrast to other symbiotic cnidarians, such as stony corals, *Cassiopea* can tolerate heat and intense temperature fluctuations(17).

Understanding the *Cassiopea* natural microbial community could provide new potential insights into the function and diversity of cnidarian holobionts.

Past jellyfish (class Scyphozoa) microbial studies have focused on the microbiome of lab-reared specimens (18,19) or limited sample sizes (20–22). While these types of studies may be able to track state changes across life stages (18) and between condition types (symbiotic/aposymbiotic) (19), there is little guarantee that the microbial communities of jellyfish kept under prolonged laboratory conditions translate ~~to~~ bear resemblance to *in situ* ~~conditions~~microbiomes. Recent *in situ* studies have provided insights into microbial taxa associated with some scyphozoans ("true jellyfish") but have not included species of the genus *Cassiopea*. In the moon jellyfish, *Aurelia* spp., individuals from the Chinese coast were *Vibrionaceae*-dominated, while samples from the northwestern Atlantic were *Mycoplasma*-dominated(22,23). Between the Indonesian marine lakes Kakaban, Haji Buang, and Tanah Bamban, *Mastigias* microbial community composition varied significantly by lake, but consistently included *Oceanospirillales* (primarily *Endozoicomonas*-like) (20). In samples across taxa, *Mycoplasma* is a common component of the identified gut and tissue microbiome of jellyfishes, though its function remains unknown (22–27). ~~Pathogenesis~~ Vertebrate pathogen research is often a focus of scyphozoan microbiome discovery—~~y.~~ *Tenacibaculum* has been identified in several scyphozoan species, e.g. *Cotyllorhiza tuberculata*, *Aurelia aurita*, and *Pelagia noctiluca*, and, as a fish pathogen, which can have important fisheries ramifications (22,23,25,28).

As most studied jellyfish populations occur in coastal waters only sporadically, the relative consistency of *Cassiopea* assemblages provides a system in which location effects can be analyzed at single time points. *Cassiopea* spp. presents an opportunity to act as a comparison

group for the microbial communities associated with both other scyphozoans and symbiotic anthozoans. The ubiquity of *Cassiopea* assemblages within the Florida Keys allows for the examination of differences in the microbial community of one population (Florida Keys *Cassiopea*) at one time point across a broad geographic area.

The aim of this work is to identify the core components of the internal and external mucosal microbiomes of *Cassiopea* and compare the microbial communities of *Cassiopea* across sites of the Florida Keys.

~~2 MATERIALS & METHODS~~Materials and Methods

Formatted: Font: 18 pt, Bold

~~2.1~~ Sampling

Formatted: Font: 16 pt, Bold

A total of 55 medusae were collected by wading in eight sites along the length of the Florida Keys in late August 2021 over eight days (see Table 1 for a list of sites). Species identification for each individual is reported in Muffett & Miglietta 2023 and summarized in Supplementary Table S1 (29). At each site, coordinates, temperature, time of day, depth, salinity, and pH were recorded (Table 1). Salinity was measured with a refractometer, and temperature and pH were measured with an Apera A1209. A minimum of two and a maximum of 10 medusae were sampled per site. At each site and in between each medusa, all equipment was cleaned with absolute ethanol and 10% bleach solution. A 1 L water sample was collected from above (>10 cm above the benthos) each medusa aggregation and filtered (0.2 um pore size, Thermo Scientific Cat No.09-740-30G) with a manual sampler as described in the USGS manual water sampling protocol (30), replicate filters were placed in 15 mL of absolute ethanol or dimethyl sulfoxide ethylenediamine tetraacetic acid saturated salt storage solution (1 L pH 7.5:

93.06 g EDTA, 60 mL 20 % NaOH solution, 20 mL 25 % HCl, 40 mL DMSO, 800 mL water, NaCl to oversaturation; see ref. 30) (commonly referred to as DESS), one filter in each buffer. At each *Cassiopea* medusa aggregation, duplicate scrapings of ~10 g of sediment collections (~~benthic~~ substrate sample) were placed in either 15 mL of absolute ethanol or DESS storage solution.

Table 1: List of collection sites. Table with latitude, longitude, salinity, pH, surface temperature, average diameter of collected medusae from the site, and date of collection. Number of paired GVC and bell swab samples per collection are for *Cassiopea xamachana* (CX). For collections that included *C. andromeda* mitotyped medusae, the number of *C. andromeda* samples is presented after the dash (CA). These values only include samples collected in DESS.

Site Name	Latitude	Longitude	Salinity (ppt)	pH	Temp (C)	Average depth (m)	Average col. medusa diam (cm)	# of GVC Samps	# of Bell Samps	Date (AM/PM)
Key West	24.561526	-81.7881727	36	8.2	30.4	0.56	6.62	CX 3/ CA 1*	CX 3/ CA 1*	15.8.2021 PM
Cudjoe Key	24.6775378	-81.4991819	36	8.4	32.6	0.36	5.91	CX 4/ CA 2*	CX 4/ CA 2*	17.8.2021 AM
Big Pine Key	24.6978731	-81.3572574	35	7.7	29.1	0.15	4.43	CX 4	CX 4	15.8.2021 PM
Bahia Honda Key TP1	24.6825793	-81.2293673	37	8.3	31.8	0.86	12.41	CX 2	CX 2	13.8.2021 PM
Bahia Honda Key TP2	24.6825793	-81.2293673	36	8	29.4	0.51	10.28	CX 2	CX 2	15.8.2021 AM
Marathon Key	24.69396	-81.0980507	31	8.1	35.2	0.08	6.53	CX 7	CX 7	11.8.2021 PM
Lower Matacumbe Key	24.8582157	-80.7267927	35	8.1	32.9	0.89	11.40	CX 2	CX 2	16.8.2021 PM
Tavernier	25.0233783	-80.4940316	31	8.1	33.3	0.35	12.26	CX 3	CX 3	16.8.2021 AM
Key Largo	25.0872104	-80.4415797	35	7.8	32.0	0.56	4.30	CX 3/ CA 1*	CX 3/ CA 1*	18.8.2021 AM

Formatted: Font: Not Bold
Formatted: Font: Not Bold
Formatted: Font: Not Bold, Italic
Formatted: Font: Not Bold
Formatted: Font: Not Bold, Italic
Formatted: Font: Not Bold
Formatted: Font: Not Bold, Italic
Formatted: Font: Not Bold

Each *Cassiopea* medusa was collected from shallow waters <1.5 m within a single assemblage at each site. Medusae were gently grasped by the oral arms, lifted from the water, swabbed along the apex of the bell, then placed bell-down onto a pre-prepared dissection plate (Figure 1). External bell swabs were done in replicate and placed in 3 mL DESS or absolute ethanol. Medusa oral arm base was bisected between oral arm groups, and replicate swabs were inserted and circled the digestive cavity. One internal swab was placed in DESS, the other in ethanol, as with external swabs. A section of bell tissue was then collected and placed in ethanol for mitochondrial species confirmation (see 28). The size of each medusa was ~~taken~~measured, and individual medusae were photographed. As sampling was done in a nonsterile environment, blanks for buffer with swab and buffer with filter were also taken.

Figure 1: Sampling strategy for medusae collection onsite. Demonstration of the typical orientation of medusa (right upper) and swab locations (apical bell surface and internal to medusa). GVC access strategy is displayed on the left and relevant environmental samples (water filter, substrate sample) are displayed on the right.

Formatted: Line spacing: single

DNA ~~2.2~~ Extraction and Sequencing

Formatted: Font: 16 pt, Bold

Formatted: Font: 16 pt, Bold

Samples in DESS and EtOH were maintained at room temperature for three to ten days and then frozen. DESS can be stored at room temperature for at least thirty days without significantly compromising community integrity (31). DNA from all samples (see Supplementary Table S1) was then extracted using Zymobiomics DNA Kits (PN# D4300T). All samples were pre-tested through the ampliciation of the 16S rRNA gene (variable region V4) amplifications. Only samples that produced visible bands for the V4 region were sent for 16S rRNA V3-V4 preparation and sequencing. While DESS preserved samples yielded good amplification results, EtOH-preserved samples largely failed to produce adequate amplification.

As such, only ~~a subset of EtOH-preserved samples were sent for sequencing~~DESS-stored samples were analyzed. Sequencing was performed by the University of Texas Medical Branch Sequencing Lab. Library was prepared with Zymo Quick 16S V3-V4 kit (PN #D6400) in 20 uL reactions (10 uL Quick-16S qPCR premix, 4 uL Quick-16S V3-V4 primers (341f: (CCTACGGGDGGCWGCAG, CCTAYGGGGYGCWGCAG) and 806r: (GACTACNVGGGTMTCTAATCC)), 4 uL water and 2 uL sample). qPCR was run using Roche LightCycler 480 Instrument II (10min 95C, [30sec 95C, 30sec 55C, 3min 72C]x20). PCRs were enzymatically cleaned according to Zymo Quick-16S kit protocols, and barcodes were added in a secondary reaction at 20 uL (10 min 95˚C, [30 sec 95˚C, 30 sec 55˚C, 3 min 72˚C]x5). Plates included two replicate microbial community standards (Zymobiomics Cat. No. D6300) and two PCR negative controls. Samples were loaded at 10 pM, with 15% 10 pM PhiX sequencing control, and run on Illumina MiSeq with a 600 cycle v3 kit.

~~Three samples were sent to CD Genomics for full length 16S on PacBio Sequel using universal 16S primers (F: AGRGTTTGATYNTGGCTCAG; R:TASGGHTACCTTGTTASGACTT). Samples sent for full length 16S rRNA sequencing included gastrovascular cavity swab extractions from medusae Marathon Key GVC 30 and Key West GVC 96 and GVC 120. As the usable reads returned from this were low (597-12520 per sample), these full-length identities are not discussed in the main text. For further details, please refer to the appendix (A1).~~

~~2.3~~ Data ~~analysis~~preprocessing

Formatted: Font: 16 pt, Bold

Data generated was run through the ~~mothur v1.47 (31)~~DADA2 v 1.26 pipeline on R v 4.4 with only limited deviations from the ~~published~~ standard ~~mothur MiSeq~~ protocols for ASV

generation(32,33) ~~(full pipeline~~. Briefly, sequences were filtered and trimmed (“filterAndTrim(truncLen=c(285,230), maxN=0, maxEE=c(4,4), truncQ=2,trimLeft = c(17,21)”), errors were error corrected (“learnErrors”, “dada”), and sequences were merged (“mergePairs”). Reads between 380 and 458 bp were retained. Chimeric reads were removed (“removeBimeraDenovo”), then taxonomy was assigned using the Silva v 138.1 database (“assignTaxonomy”). Retention metrics were retained and these metrics along with the full code can be found on the github (https://github.com/kadenmuffett/Cassiopea-Code-Microbiome-Repository). Sequence and metadata tables were imported into phyloseq v 1.42, where reads assigned to mitochondria and chloroplast were removed. Contaminants were removed using the decontam package v. 1.18, identifying and removing 47 ASVs based on differential abundance in swab and buffer controls(34,35).

For all downstream analyses, dataset was limited to those stored in DESS and medusae without a *C. andromeda* mitotype. This includes 60 paired bell and GVC samples (30 of each), 8 water samples and 8 substrate samples with 6.47 mil sequences across 34808 taxa. ~~can be found in supplementary material S02) (32). Sequences attributable to Chloroplast, Mitochondria, Eukaryota, and unattributable sequences were removed ("remove.seq"). All genera found in the negative controls (4 PCR controls, 5 sampling and extraction controls) at a rate greater than 1% were removed using "remove.lineage". Number of sequences removed at each of these filtering steps can be found in Supplementary Table S03. Based on the sequenced microbial community positive control, ASVs were oversplit, and the amplification was biased towards Gram-positive bacteria. Given this oversplitting, analysis for 16S rRNA gene V3-V4 data was lumped by classification at the family level.~~

Formatted: Indent: First line: 0.5"

Formatted: Font: Italic

The complete dataset (NCBI Bioproject Accession number PRJNA1020388) contains 12.78 million reads. included 13.27 million sequences after merging ("make.contigs"), 7.17 million after screening for homopolymers and length ("screen.seqs": maxlength = 480, maxhomop = 8). Unique sequences were aligned to a 16S rRNA small subunit V3-V4 reference database generated from the Silva v. 132 full database ("pcr.seqs") and filtered again to produce 3.66 million unique sequences that were clustered into ASVs ("pre.cluster": diffs = 4). After chimera removal ("chimera.vsearch"), 2.27 million unique sequences were classified according to the Silva reference library v.132 ("classify.seqs"). ASVs were phylotyped at family level. After quality filtering, 947 families were present. Explorable phylotype datasets can be found in the supplementary materials (S4-S6).

2.4 Statistical analyseis

Formatted: Font: 16 pt, Bold

Formatted: Font: 16 pt, Bold

Refraction curves were generated in vegan 2.6.6.1 ("rarecurve") (SFIG. 1)(36). Shannon diversity was computed with vegan and observed diversity was ploted with phyloseq ("estimate_richness", "plot_richness")(34). Faith's phylogenetic diversity was computed using the MiscMetabar v 0.10.1, phanghorn v 2.12.1, metagMisc v 0.5.0 and PhyloseqMeasures v 2.1 packages by first generating an ML tree from all sequences over 300 reads across the dataset, then computing per sample Faith's diversity (37–42). Faith's diversity was displayed with ggstatsplot v 0.13.0 (43). Bray curtis and Euclidean distance were computed in phyloseq with "distance" to "ordinate" to "plot_ordination". Between group comparisons were completed with the pairwiseAdonis package v 0.4.1 (" pairwise.adonis2") on Euclidean distance measurements (44). Statistical comparisons were computed with Kruskal-Wallis using the Kruskal.test and pairwise comparisons using the "kruskal.test" and "dunn.test" commands with holm p adjustment (45).

Data transmutation for starplots and display was assisted by the speedyseq package v 0.5.3.9021 (46). Star plots were made using the stars package v 0.6-7 (47). Differential abundance was calculated with ALDEx2 v 1.38.0 following established pipelines for microbiome data (48,49). Dirichlet Multinomial mixture analysis, as described by Holmes and implemented in the DirichletMultinomial package v 1.48 (50,51). Core microbiome was computed with the microbiome package v 1.28.0(52).~~, principal component analysis, non-metric multidimensional scaling (NMDS), diversity metrics, Dirichlet multinomial mixture and analysis of molecular variance were computed in mothur (41). NMDS plot was visualized using R 4.0.1 (R Core Team, 2021).~~

Ethics Statement

Formatted: Font: 16 pt, Bold

Formatted: Indent: First line: 0"

This work was performed on lower level invertebrates not subject to Animal Use Protocols or within the purview of Texas A&M University IRB approval. Permitting for these specimens was waived by Florida Fish and Wildlife Conservation Commision.

Formatted: Indent: First line: 0.5"

~~3 RESULTS~~Results

Formatted: Font: 18 pt, Bold

~~3.1~~ Data description

Formatted: Font: 16 pt, Bold

We analyzed the microbiome associated with 34 medusae from eight sites within the Florida Keys. For each medusa, we analyzed the microbial composition of their gastrovascular cavity (GVC) and external or bell mucus (herein referred to as “bell”), as well as their ~~benthic~~ substrate (Sub) or water environment (WTR). All medusae were identified as *Cassiopea xamachana*, with the exception of four medusae identified as mitochondrial haplotype *C. andromeda* found in Key West, Cudjoe Key, and Key Largo (see Supplementary Table S1).

10.13 and 9.07 million reads were retained after filtering (“filterAndTrim”) and merging (“mergePairs”) respectively (for all retention statistics, see supplementary table S2) . There were 8.91 million sequences after chimera removal. After primary phyloseq filtration, the dataset included 6.47 million sequences retained in 34808 unique ASVs across 76 total samples (30 bell, 30 GVC, 8 water, 8 substrate). Reference sequences for top ASVs are also available in the supplementary material (table S3).

~~3.1.1~~ Distribution of sequences in full dataset

Formatted: Font: 14 pt, Bold

~~Almost all of the 5,431,716 final sequences were Bacteria as opposed to Archaea~~The dataset generated was predominantly bacteria with few archael reads. ~~(98.93% to 1.07%).~~ At the phylum level, ~~66.75~~57% of sequences were Proteobacteria, ~~9.50~~7% were ~~Bacteroidetes~~Firmicutes, 13~~8.58~~% were ~~Tenericutes~~Bacteroidota, and 4~~2.45~~% were Cyanobacteria (Fig 2). No other phylum had greater than 3~~2~~% relative abundance in the dataset.~~.~~

Figure 2. Top ten (top~~left~~) ~~phylum level~~phyla and (~~right~~bottom) ~~class~~ class ~~level distribution of~~relative abundances of sequences in samples aggregated by sample type.

At the class level, Gammaproteobacteria comprised 41~~8.90~~% of all sequences. Alphaproteobacteria, the next most abundant, comprised 17~~12.07~~%. Bacteriodia (12~~8.83~~%), Bacilli ~~Mollicutes~~ (6~~8.58~~%), Cyanobacteriia ~~Deltaproteobacteria~~ (4~~5.38~~%), and Desulfobacteria ~~Oxyphotobacteria~~ (3~~2.37~~%), ~~and Verrucomicrobiae (1.27%)~~ were the other classes with a~~n~~ relative abundance above 2~~1~~%. ~~The~~ No Archaean group was prevalent above a relative abundance of 0.25%, with the three most abundant classes being Halobacterota (0.25%),

Thermoplasmatota (0.21%) and Nanoarchaeota (0.22%).majority of Archaea within the dataset were from the classes Halobacteria (40.17%) and Woesearchaeia (33.94%).

3.1.2 Distribution of sequences within gastrovascular cavity (GVC) samples

Within gastrovascular cavity samples, 70.40% of the 2,485,856 total sequences were Proteobacteria, 17.89% Tenericutes, 4.02% Bacteriodetes, 1.75% Cyanobacteria, and 1.19% Chlamydiae. 73.2075% of all sequences from gastrovascular cavities belonged to just three orders, Pseudomonadales Oceanospirillales (3942.01%), Mycoplasmatales (17.8024%), and Enterobacterales Vibrionales (13.38%). The majority of Oceanospirillales Pseudomonadales sequences identified within the GVC were monotypic at the family leveldispersed across very few OTUs, with 98.0766% identified to the familyas *Endozoicomonas atrinae* ASV1 and 8%Endozoicomon as *E. atrinae* ASV4adaceae. This was true of the Mycoplasmatales as well, with 77% of associated gut sequences99.96% were identified as sequences from the family Mycoplasmataceae identified as *Mycoplasma* sp. ASV2 or *Mycoplasma* sp. ASV9 (15%)..

3.1.3 Distribution of sequences within external bell mucus samples

Of the 2,012,513 total sequences fromAt the phylum level, external within the bell mucosal swabs, 65.5755% of the sequences were Proteobacteria, 142.562% BacteroidotaBacteriodea, 3.175.1% Cyanobacteria, and 62.54% DesulfobacterotaPlanctomycetes.

At the order level, the most abundant groups were 19.34% of sequences were Pseudomonadales (23%)Oceanospirillales, 6.65% unclassified Chromatiales (6%)Gammaproteobacteria, 6.33% Rhodobacterales (7%), 4.89% Desulfobacterales (5%), and 4.78% Chromatiales, 4.30% Flavobacteriales (5%). *Endozoicomonas atrinae* ASV1 was the single most abundant surface

Formatted: Font: 14 pt, Bold

Formatted: Font: Italic

Formatted: Font: Italic

Formatted: Font: Italic

Formatted: Font: Italic

Formatted: Font: 14 pt, Bold

mucus ASV at a mean of 12%. The next most abundant were Synechococcus CC9902 ASV15 (1.3%), *Endozoicomonas atrinae* ASV4 (1.1%) and Chromatiales *Candidatus Thiobios* ASV6 (0.8%)., with fourteen additional orders comprising between 1 and 2.5% of abundance each.

Formatted: Font: Italic

Formatted: Indent: First line: 0"

84.40% of the external swab Oceanospirillales sequences were placed within the family Endozoicomonadaceae. The families Rhodobacteraceae (6.33%), Chromatiaceae (4.29%), Flavobacteriaceae (3.22%), and Desulfobacteraceae (3.35%) also had abundances greater than 2%.

3.1.4 Benthic Substrate and water samples

Formatted: Font: 14 pt, Bold

Water samples shared some ASVs with the bell mucus of medusae, the largest contributing ASV was Chromatiales *Candidatus Thiobios* sp. ASV6 (8%). Additional prominent taxa were Rhodobacteraceae *HIMB11* ASV12 (6%), *HIMB11* ASV19 (5%), and SAR11 Clade III ASV 10 (4%). By contrast, substrate samples had no ASVs surpassing a mean of 1% abundance across sites, with the highest two ASVs Sandaracinaceae ASV94 and *Actibacter* ASV105 reaching 0.6% mean abundance across sediment samples (see table 2; extended table is table S4).Benthic samples were primarily dominated by Proteobacteria (51.28%) and Bacteroidetes (19.77%). At the order level, Desulfobacterales (9.67%), unclassified Gammaproteobacteria (7.50%), Rhodobacterales (7.16%), Flavobacteriales (5.96%), and Chromatiales (4.16%) were most common.

Formatted: Font: Italic

Formatted: Font: Italic

Table 2: Top 5 ASVs for each sample type. Table with ASVs ordered from highest to lowest average proportion in samples of each type dispayed as a percentage of composition within that sample type. Includes Silva 138 taxanomic assignment for each ASV.

	1	2	3	4	5
	ASV1 *Endozoicomonas atrinae*	ASV15 *Synechococcus* CC9902	ASV4 *Endozoicomonas atrinae*	ASV6 *Candidatus Thiobios*	ASV10 SAR11 *Clade III*
Bell	11.585 %	1.272 %	1.105 %	0.832 %	0.709 %
	ASV94 Sandaracinaceae	ASV105 *Actibacter*	ASV95 Desulfocapsaceae	ASV169 *Robiginitalea*	ASV358 *Clostridiisalibacter*
Sub	0.625 %	0.605%	0.566%	0.492%	0.474%
	ASV6 *Candidatus Thiobios*	ASV12 Rhodobacteraceae *HIMB11*	ASV19 Rhodobacteraceae *HIMB11*	ASV10 SAR11 *Clade III*	ASV18 *Marinobacterium*
WTR	7.521 %	5.839%	4.725%	4.497%	2.711%
	ASV1 *Endozoicomonas atrinae*	ASV2 *Mycoplasma*	ASV9 *Mycoplasma*	ASV4 *Endozoicomonas atrinae*	ASV11 *Vibrio coralliilyticus*
GVC	34.133%	20.099%	2.823%	2.327%	2.094%

~~Proteobacteria (68.26%) and Bacteroidetes (14.91%) were the primary phyla of water samples. Primary orders within water samples were Oceanospirillales (15.30%), Rhodobacterales (14.96%), SAR11 (11.92%), Flavobacteriales (10.76%), and Chromatiales (7.41%). Only 1.35% of water sample Oceanospirillales sequences were assigned to Endozoicomonadaceae.~~

~~3.2~~ Diversity

~~Alpha~~ Shannon diversity varied by sample type (Kruskal-Wallis: chi-squared = 48.8, df = 3, p-value =1.412e-10). Shannon diversity had a mean of 2.30 in GVCs, 4.01 in water samples, 5.63 in substrate samples and 6.56 in bell samples. Shannon diversity indices for the gastrovascular samples varied from substrate samples and bell samples (Kruskal-Wallis Dunn test with Holm correction: GVC-Bell, p-val=0.00; GVC-Sub, p-val= 0.00; GVC-Water, p-val=0.07; Bell-Sub, p-val=0.07; Bell-Water, p-val=0.06; Water-Sub, p-val=0.01). Shannon diversity was not explained by location (Kruskal-Wallis: chi-squared = 9.7, df = 7, p-value =0.21)(Fig. 3).

~~diversity was lower in the gastrovascular cavity samples than in external mucus, benthic, or water samples. The Shannon and Inverse Simpson of gastrovascular cavity communities was low (H = 1.73+/-1.81, 1/D = 4.13+/-8.97) when compared to external mucus (H = 3.83+/-1.37, 1/D = 23.46 +/-21.96) and environmental samples (Water: H = 3.19+/-1.24, 1/D = 13.98489+/-15.12037; Benthic: H = 4.21+/-0.33, 1/D = 30.07+/-9.51) (Figure 3) (ANOVA, df = 3, p-val = <2e-16). Family richness was significantly lower within medusae, 155 (+/-158) families were observed in gastrovascular samples, and 270 (+/- 195) were observed in external samples (ANOVA, df = 3, p-val = 6.69e-09).~~

Figure 3. ~~**Order level diversity of samples by body compartment, including the top 20 most common orders by name.**~~**Shannon diversity (a) by sample type and (b + c) location.** GVC, bell, substrate and water Shannon diversity are shown across both the entire dataset or when segmented by sample type across sites with (b) GVC and (c) bell alpha diversity across sites. All p values of holm-adj Dunn comparison of means tests below a = 0.05 are displayed, with statistical test details displayed above each plot.

Formatted: Font: Not Bold

Formatted: Font: Not Bold

~~3.3 Community~~ **Beta diversity**~~identity~~

Formatted: Font: 14 pt, Bold

Euclidean distance showed differences between medusa and non-medusa samples (pairwise.adonis, GVC vs bell: p=0.001, bell vs sub: p=0.049, bell vs water: p=0.022, GVC vs sub: p=0.001, GVC vs water: p=0.001, sub v water p=0.001). Variability along the primary axis was found in both GVC and bell samples, while variability along the secondary axis was found mainly in GVC samples. The location of collection had no clear impact on beta diversity.

Formatted: Not All caps

Figure 4. PCA of Euclidean distance of samples. PCA is colored by sample collection latitude from low (orange) to high (purple). Sample type is designated by shape, circle (Bell), triangle (GVC), square (Substrate) and cross (Water). The plots show the same PCA faceted by sample type.

~~External and internal~~Faith's diversity of communities were distinct from each other (~~AMOVA~~Kruskal-Wallis test, chi-squared = 44.1, df = 3, p-value = 1.4e-09~~, Fs = 20.146, df = 1, p-value: <0.001~~). GVC communities were distinct from ~~benthic~~ bell and ~~water~~ substrate comparison groups, while bells were distinct from water and gastrovascular cavities ~~as well~~ (~~AMOVA, GVC-Benthic Fs = 15.28, df = 1, p-value: <0.001; GVC-Water Fs = 11.87, df = 1, p-value: <0.001~~Dunn test with holm correction) (Fig.~~ure~~ 5~~4~~). ~~External mucus communities were distinct from water (AMOVA, Fs = 8.257, df = 1, p-value: <0.001) but not statistically significant from benthic samples (AMOVA, Fs = 3.214, df = 1, p-value: 0.01). Clustering difference was most distinct between GVC and environmental samples.~~

Figure 5. Violin plot of Faith's diversity. Samples are colored by sample type. Kruskal-Wallis chi-sq, pvalue and pairwise dunn test with holm correction p-values are displayed. Differences between GVC-water and substrate-bell are not significant.

~~**Figure 4. Two-dimension NMDS plot segregated by sample type. Stress = 0.255711, R-squared for configuration: 0.750874**~~

~~Within gut microbial communities, Dirichlet multinomial mixture analysis defined two community profiles, either low Endozoicomonadaceae with higher Vibrionaceae and Rhodobactereaceae (Community Type 1) or high Endozoicomonadaceae with low Vibrionaceae and high Mycoplasmataceae (Community Type 2), with Endozoicomonadaceae being the largest determining component. These profile types were not evenly distributed across sites (to see Dirichlet partitions refer to S7, to see likelihoods of Dirichlet models refer to S8, for final model~~

Formatted: Line spacing: single
Formatted: Font: Bold
Formatted: Not Highlight
Formatted: Not Highlight
Formatted: Not Highlight
Formatted: Not Highlight
Formatted: Not Highlight
Formatted: Not Highlight
Formatted: Not Highlight
Formatted: Highlight
Formatted: Line spacing: single
Formatted: Font: Not Bold
Formatted: Font: Bold
Formatted: Highlight

~~parameters see S9, for comparative value of community members see S10, for Dirichlet types by site refer to S12, to see 16S rRNA gene histograms grouped by site, refer to supplementary figure S11).~~

~~Community Type 1 sites were Lower Matacumbe Key, Bahia Honda Key, and Key Largo. Sites that included a mix of community type 1 and 2 individuals were Tavernier (C2:C1 = 2:1) and Marathon Key (C2:C1 = 5:2). The Lower Keys sites, Key West (C2:C1 = 3:1), Cudjoe Key (C2:C1 = 6:0) and Big Pine Key (C2:C1 = 4:0), were primarily community profile 2. Comparative star plots of community components separated by site can be seen in Figure 5.~~

~~**Figure 5. Star plots of community composition for internal and external mucus swabs at each site separated by order (three most prominent orders and "other"). Each spoke of the star plot ranges from 0% (center) to 100% (edge of shaded diamond). External group spokes (going clockwise): Other, Oceanospirillales, Rhodobacterales, and Desulfobacterales. Internal group: Other, Oceanospirillales, Mycoplasmatales, and Vibrionales. Each site's measured environmental conditions (salinity, water surface temperature, and pH) are presented below star plots. Sites are mapped onto their location within the Florida Keys.**~~

Formatted: Line spacing: Double

~~3.3~~ Core microbiome

Formatted: Font: 16 pt, Bold

Formatted: Indent: First line: 0"

Core microbiome for the purposes of this study was evaluated based on ~~100~~70% occurrence of an ASV ~~family~~ and a relative abundance of > 0.1~~2~~% of reads in all 34 gastrovascular cavity samples or external bell samples. This is ~~a conservative component~~in ~~definition compared~~line with thresholds for ~~to~~ other core microbiome work (53). The most abundant ~~clade~~ASV, *Endozoicomonas* ASV1~~daceae~~, was present in ~~30 of the 34~~90% of GVC samples at a relative abundance of > 0.1% of reads, ~~this level~~ASV2 and ASV4~~,~~ were present in

Formatted: Font: Italic

80% as was Vibrionaceae. Using less stringent criteria (>70% of samples, any abundance), ASV 11, ASV 132 and ASV 6 become part of the core microbiome (Fig 6). These ASVs are across only three genera- *Endozoicommonas*, *Mycoplasma* and *Vibrio* (full taxonomic details of ASVs can be found in table S5).

For Mycoplasmataceae, 26/34 samples met a 0.2% threshold. There were no medusae where all three of these families were absent from the gastrovascular cavity, and three medusae in which two of the three were absent.

Figure 6. Abundance of Cassiopea core GVC microbiome across dataset. The top five ASVs from the gut core microbiome across all sample types (Bell, GVC, Substrate (Sub) and Water (WTR)) organized from furthest south to furthest north sites. Note the difference in y axis maxima between bell and GVC samples (top) and substrate and water samples (bottom).

In bell samples, ASV1, ASV104, and ASV86 have 73%, 70% and 70% relative abundance in samples at a 0.1% threshold. ASV6, ASV95, ASV37, ASV105, and ASV135 are present at any level in >70% of bell samples. These ASVs cover the genera *Endozoicommonas*, *Candidatus Thiobios*, *Methyloceanibacter*, *Ruegeria* and unknown genera from Desulfocapsaceae and Actibacter (Fig. 7).

Figure 7. Core microbiome prevalence in GVC and bell. Heatmap of gastrovascular cavity (top) and bell (bottom) shaded by prevalence (0-1 scale) vs detection threshold (proportions of reads in samples) across the ASVs identified as core in each group.

In terms of abundance, internal core microbiome components are not just present, but highly dominant in *Cassiopea*'s gut. Some of these taxa are highly restricted to GVC samples (*Mycoplasma*), while others are prevalent across body surfaces (*Endozoicomonas*) (Fig. 7).

Formatted: Font: Italic
Formatted: Font: Not Italic
Formatted: Font: Bold
Formatted: Font: Not Bold
Formatted: Indent: First line: 0", Line spacing: single
Formatted: Font: Italic
Formatted: Font: Italic
Formatted: Line spacing: single
Formatted: Font: Not Bold
Formatted: Indent: First line: 0"
Formatted: Font: Italic
Formatted: Font: Italic
Formatted: Font: Italic

The vast majority of ASVs were not differentially abundant between bell and GVC samples. Differential abundance (Aldex2, min effect size absolute value >[±2]) between the gastric and bell surfaces is restricted to the novel *Mycoplasma* group (ASV2), which is completely lacking in external samples (Fig 8). No features were differentially abundant between substrate and bell samples (Sfig 2).

Figure 8. Aldex2 plot of effect size of all ASVs between bell and GVC samples. Only ASV2 has an effect >[± 2].

Dirichlet multinomial mixture (DMM) analysis identified specific proportions of many of these core microbiome as valuable for category differentiation. Within animal-associated samples, model probability value was optimal for two communities, one GVC and one bell without subcommunities. False assignment rate (mismatch between predicted community and true sample type) was present in 5/60 samples. ASV1 amount was a core group differentiator, with fewer meaningful ASVs defining the GVC community than the bell community (Fig. 9).

Figure 9. Most impactful ASVs for Dirichlet multinomial modeling. Top 0.5% most impactful drivers of body cavity prediction in GVC (top) and bell (bottom) Dirichlet multinomial models ordered from largest to smallest effect size.

Despite DMM identifying all gastric samples as belonging to the same community, relative proportions of those community members differed dramatically, varying substantially in abundance of *Endozoicommonas*, *Mycoplasma* and *Vibrio*. Variation within bell samples

Formatted: Font: Italic

Formatted: Font: Bold

Formatted: Font: Bold

Formatted: Indent: First line: 0", Line spacing: single

Formatted: Font: Bold

Formatted: Font: Bold

Formatted: Font: Not Bold

Formatted: Indent: First line: 0", Line spacing: single

Formatted: Font: Not Bold

Formatted: Font: Italic

Formatted: Font: Italic

Formatted: Font: Italic

primarily occurred with proportion of *Endozoicommonas*, but was not reliably distinguishable from substrate samples by DMM (Fig. 10).

Figure 10. Star plots of community composition across sites. Star plots for internal (GVC), external (Bell) mucus swabs, water (WTR) and substrate (Sub) sample averages at each site separated by order (three most prominent orders and "other"). Each spoke of the star plot ranges from 0% (center) to 100%. **Internal** group, starting from the 3' position and going counterclockwise: Pseudomonadales, Mycoplasmatales, Enterobacteriales and "Other". **External** group spokes (going counterclockwise): Pseudomonadales, Rhodobacterales, Chromatiales, and "Other". **Water** group spokes (going counterclockwise): Pseudomonadales, Rhodobacterales, Flavobacteriales, and "Other". **Substrate** group spokes (going counterclockwise): Rhodobacterales, Flavobacteriales, Desulfobacterales, and "Other". Each site's measured environmental conditions (salinity, water surface temperature, and pH) are presented below star plots, colored by intensity (low to high represented by pale to dark) . Sites are mapped onto their location within the Florida Keys.

~~3.5~~ *Vibrio*

ASVs identical to *Vibrio coralliilyticus* were found across *Cassiopea* gastric samples, with abundance of up to 10% of reads within the sample. This far surpasses rates in the surrounding water and substrate samples (ASV11 averages: Bell: 0.05%, WTR: 0.03%, Sub: 0%, GVC: 2.09%) (Fig. 11).

Figure 11. Percent of reads (0%-10%) assigned to *V. coralliilyticus* ASVs in bell, GVC, substrate and water samples averaged by site. Organized by body cavity.

Host medusa species identity

The mitochondrial haplotype of the non-native *Cassiopea*, *Cassiopea andromeda*, a cryptic sister species to *C. xamachana*, was found in four of the 34 medusae sampled. The four

Formatted: Line spacing: Multiple 1.08 li
Formatted: Font: Bold
Formatted: Font: Bold
Formatted: Font: Bold
Formatted: Font: Bold
Formatted: Indent: First line: 0"
Formatted: Font: 16 pt
Formatted: Font: 16 pt, Bold, Italic, Not All caps
Formatted: Font: 16 pt, Bold, Not All caps
Formatted: Font: 16 pt
Formatted: Font: (Default) Times New Roman
Formatted: Font: (Default) Times New Roman, 12 pt
Formatted: Font: (Default) Times New Roman, 12 pt, Italic
Formatted: Font: (Default) Times New Roman, 12 pt
Formatted: Font: (Default) Times New Roman, 12 pt, Italic
Formatted: Font: (Default) Times New Roman, 12 pt
Formatted: Font: (Default) Times New Roman, 12 pt
Formatted: Font: (Default) Times New Roman, Bold
Formatted: Line spacing: single
Formatted: Font: (Default) Times New Roman, Bold
Formatted: Font: (Default) Times New Roman, Bold
Formatted: Font: Bold
Formatted: Font: Bold, Italic
Formatted: Font: Bold
Formatted: Font: Bold
Formatted: Font: Not Bold
Formatted: Font: (Default) Times New Roman, Bold
Formatted: Font: 16 pt, Bold

C. andromeda mitotype individuals included were from the Key Largo (1), Cudjoe Key (2), and Key West (1) sites. ~~The gastrovascular communities of these individuals were not different from *C. xamachana* individuals (AMOVA, Fs = 0.722, df = 1, pval = 0.775).~~While these were excluded from the primary analysis, *Cassiopea andromeda* showed no divergence from the trends of the *C. xamachana* simultaneously collected (Fig. 12).

Figure 12. PCA of Euclidean distance between *Cassiopea* species. *Cassiopea andromeda* (CA: grey) and *Cassiopea xamachana* (CX: black) samples at the sites were they cooccurred (Cudjoe Key, Garrison Bight and Key Largo).

~~4 DISCUSSION~~

Discussion

~~By analyzing the microbiome of 34~~ *Cassiopea* medusae ~~from eight locations within the Florida Keys, we demonstrate clea~~show consistent ~~r~~ differences in the microbial communities present on their ~~external~~ bell and ~~internal~~ gastric surfaces. Gastrovascular cavity samples have lower diversity than the sediment, external surfaces, and ~~the water column surrounding medusae~~host a novel *Mycoplasma* lineage. ~~.~~This low diversity is ~~is~~ consistent with results for multiple other scyphozoans— including *Cotylorhiza tuberculata* (25). ~~*We also record*~~*Cassiopea* also appears to have some variation in community composition between sites, a feature consistent with results from other species, such as *Aurelia*, *Mastigias*, and *Tipedalia* (20,24).

~~4.1 Diversity and consistency~~A bifurcated outer and inner microbiome

Formatted: Font: Italic

Formatted: Font: Italic

Formatted: Font: Bold

Formatted: Line spacing: single

Formatted: Font: Bold

Formatted: Font: Italic

Formatted: Font: Italic

Formatted: Font: Italic

Formatted: Font: 18 pt, Bold

Formatted: Font: Italic

Formatted: Font: Italic

Formatted: Font: 16 pt, Bold

Formatted: Font: 16 pt, Bold

Across all sites, the gastrovascular cavities of *Cassiopea* medusae showed ~~no~~ a core microbiome comprised of taxa previously found in photosymbiotic cnidarians. The external microbial community of *Cassiopea* from the Florida Keys adheres fairly closely to the epibenthic microbial community at each site, with the addition of *Endozoicomonas*~~*daceae* for most individuals~~. ~~Endozoicomonadaceae~~ ASV1 *Endozoicomonas* cf. *atrinae* may be a primary mucosal component and extend to the exterior of the body through mucus exchange, as it is less prevalent on the ~~external~~ bell surface than internal for most individuals. Alternitavely, ASV1 may be a common mucosal resident with no function for the host.

T~~Notably, t~~he abundant ~~*Mycoplasmataceae*~~ *Mycoplasma* and *Vibrio* cf. *coralliilyticus* ~~*naceae*~~ from the gastric cavity of *Cassiopea* were not found in any external mucus samples and found at far lower abundances in external mucosal samples respectively. ~~Oceanospirillales (order of~~

~~*Endozoicomonas*) was the only bacterial order in which external mucus samples were enriched relative to the benthos.~~ The high diversity of the *Cassiopea* bell microbiome ~~Shannon Diversity Index values~~ found in this study ~~are higher than those~~may be unique to *Cassiopea*. Previous work has reported in the low-diversity mucosa, oral arms and umbrella of a previously studied pelagic rhizostome, *Rhizostoma pulmo,* a likely result of the constant contact of *Cassiopea* with the epibenthos (26). The ~~lack~~ limited ~~of~~ overlap between diverse ~~external~~ bell communities and low diversity ~~internal~~ gut communities in *Cassiopea* is notable and consistent with the significant differences in body compartment microbiome of other scyphozoans and anthozoans (21,24,54). While the lack of diversity in scyphozoan microbiomes may seem distinct from many described stony coral microbial communities, these stony coral microbial

Formatted: Font: Italic

Formatted: Font: Italic

Formatted: Font: Italic

Formatted: Font: Italic

Formatted: Font: Italic

Formatted: Font: Italic

Formatted: Font: Italic

Formatted: Font: Italic

profiles are often produced using whole body sampling. Lower diversity gastrovascular cavities with distinct community composition may also be a feature of stony corals (55–57).

Microbial communities across sites within this study were not significantly different from each other, as gastrovascular cavity components remained dominated by three taxa throughout the Keys. ~~This suggests that while lacking a formal "core" microbiome, *Cassiopea* gastric cavities may naturally rely on some combination of several microbial key taxa.~~ This microbiome bears limited resemblance to those sampled in common garden experiments and no resemblance to lab-held lines, demonstrating that "core" here should not be misconstrued as obligate (19,58). The lack of obligate bacterial taxa in the *Cassiopea* gastrovascular cavity is also consistent with other microbial hosts, notably the anemone *Exaiptasia diaphana*, another successful model cnidarian (1). While *Cassiopea* require their algal symbiont, bacterial partners may be transient, and bacteria may have far less significance to host health for *Cassiopea* than for specialized obligate hosts (e.g., leafhoppers) (59–62). Without a better understanding of specific metabolic function of different bacterial associates, microbiome importance cannot be determined. We identified ~~*Endozoicomonadaceae*~~*Endozoicomonas*, ~~*Mycoplasmataceae*~~*Mycoplasma*, and *Vibrio*~~*naceae*~~ as the three most common bacterial ~~families~~ genera found within the gastrovascular cavity of *Cassiopea*. These are all commonly associated with coral microbial communities (8,9,63). Below, we provide information on these common bacterial components of cnidarian holobionts.

~~4.2 *Endozoicomonadaceae*~~ ***Endozoicomonas*** **functions in microbiomes**

The large proportion of *Endozoicomonas* ~~Endozoicomonadaceae~~ in internal and external samples fits within a broader framework of both *Endozoicomonas* ~~Endozoicomonadaceae~~ and

Formatted: Font: Italic

Formatted: Font: Italic

Formatted: Font: Italic

Formatted: Font: Italic

Formatted: Font: 16 pt, Bold, Italic

Formatted: Font: 16 pt, Bold

known cnidarian-associated microbial communities; *Endozoicomonas* strains are present in a variety of invertebrates, including many corals, sponges, ~~and~~ sea anemones, ~~but also~~ scyphozoans and cubozoans (9,63–66). Some hosts house multiple distinct *Endozoicomonas* genotypes, ~~which indicates~~suggesting that different *Endozoicomonas* clades may provide different metabolic services to a host (e.g., Vitamin B12 metabolism) (67). *Endozoicomonas* ~~Endozoicomonadaceae~~ is ~~not restricted to symbiotic taxa but has been~~ more commonly identified in healthy scleractinian corals than bleached and diseased ones (68). For these corals~~taxa~~, it may play a role in a variety of fundamental *Symbiodinium*–host interactions, including nutrient transport and symbiont cell breakdown (67). Notably, *Endozoicomonas* ~~Endozoicomonadaceae alone~~ is not a consistent marker ~~n arbiter~~ of microbiome health, its disappearance and reappearance without apparent host health declines has been identified in octocorals (2,66). In some coral, members of *Endozoicomonas* are found in close proximity to *Symbiodinium*, it is unknown whether this feature is shared with *Cassiopea* (67).

Formatted: Font: Italic

Within scyphozoa, *Endozoicomonas* is abundant in the microbial community of the rhizostome scyphozoan *Mastigias* (20). While *Mastigias*, like *Cassiopea*, host zooxanthellae from the ~~clade~~ family *Symbiodiniaceae*, ~~however~~ not all symbiotic rhizostomes ~~appear to~~ harbor the microbe, and not all taxa with the microbe are symbiotic (20,25). *Endozoicomonas* may be common across medusae, but the difficulty of culturing some strains may have excluded them from the results of past culture-based analyses (66,67). Greater clarity is needed on the function of *Endozoicomonas* for these medusae and other taxa.

Formatted: Font: Not Italic

~~4.3~~ Mycoplasmataceae: a poorly understood cnidarian microbiome resident

Formatted: Font: 16 pt, Bold

Mycoplasma~~taceae~~, ~~as with~~like ~~*Endozoicomonadaceae*~~*Endozoicomonas*, is present in studied coral and scyphozoan microbiomes (9,22,23,25). While intracellular parasitism is common for members of Mycoplasmataceae, those associated with cnidarians may be located extracellularly and exist as opportunistic commensals (2,69). *Mycoplasma* sp. is noted as a potential endosymbiont in lab-raised *Aurelia* polyps, while adults from many jellyfish populations are known to have them (22,24,27). The high divergence between this *Mycoplasma* and others of the genus (closest sequence identity on NCBI at 89%: Accn. No. NR_115937) suggests that this may be a hitherto unknown group. Whether this *Mycoplasma* ~~group~~ represents an endosymbiont, a result of prey capture, or a parasite, its exclusivity to the gastrovascular tract in *Cassiopea* sampled within the Keys is notable, as other studies have not demonstrated gastrovascular cavity localization (24,27).

~~4.4~~ Vibrionaceae

Vibrionaceae is both a core part of coral microbiomes (9,70) and known to facilitate metamorphosis within *Cassiopea* (71,72), however, it is a highly diverse family. While *Vibrio* spp. are found in gut communities of presumed healthy scyphozoans (Daley et al. 2016, Peng et al. 2021), they can also be associated with senescence and disease (Kramar et al., 2019; Tinta et al., 2012). For *Cassiopea* specifically, an increase in *Vibrio* has previously been ~~may be~~ associated with an aposymbiotic or stressed state (58). Given the variation in Vibrionaceae species and strains, family-wide statements on the beneficial or harmful nature of Vibrionaceae within *Cassiopea* gastrovascular cavities should not be made.

Vibrio coralliilyticus

Formatted: Font: Italic

Formatted: Font: Italic

Formatted: Font: Italic

Formatted: English (United States)

Formatted: Font: 16 pt, Bold

Formatted: Font: 14 pt, Bold, Italic

Formatted: Font: 14 pt, Bold

In this dataset we found ample signal of a 16S V3-V4 with 100% sequence match to multiple *Vibrio* strains, including *V. coralliilyticus* and the oyster pathogen, *V. tubiashii*. As *Vibrio* species are highly varied, the sequence match does not mean assured pathogenicity towards any taxa (75–77). The apparent health of the medusae from whom these samples were collected suggest a either a lack of impact of this specific group on the host or a host-microbe facultative interaction between this *Vibrio* and *Cassiopea*. While there is data on *Cassiopea* microbiome antimicrobial isolates and *Cassiopea* mortality when faced with *Serratia marcescens* infection, we lack adequate details on the *Vibrio – Cassiopea* system to determine whether this represents a lowlying widespread infection or a mutualism (78,79).

Drivers of external microbiome

While the external microbiome was not distinct from the sediment around it, it is worth discussing the major groups more common externally than internally. While the core bell microbiome represents a substrate-like community, the bell core microbiome includes representatives from families and genera with a variety of potential metabolic capacities. The most plentiful of these ASVs was *Candidatus Thiobios*, known as a sulfur-oxidizing epibiont of the *Zoothamnium niveum* (80,81). As this bacteria can be found in the Florida Keys, their appearance on *Cassiopea* may be circumstantial, but is consistent across medusae (80). Isolates from *Methyloceanibacter* have been demonstrated to be methane oxidizers from marine environments (82). These may be sediment associated or may recruit to the host to utilize algal products. The potential sulfate reducers within Desulfocapaceae may similarly recruit to the bell and its access to sediment pore waters (83). While *Pseudomonas* cf. *stuzeri* was not as high abundance as the other metabolizers listed above, it may have a role in denitrification or coral health for some species (84–86)

In addition to microbes with known metabolic functions that align with the *Cassiopea* holobiont, the genus *Ruegeria* includes many marine species and has been identified in the microbiomes of photsymbiotic and nonphotosymbiotic cnidarians alike (9,73,87). As Dirichlet modeling for bell microbiomes gave similar weight to many bacteria, the relative impact of all of these bacterial epibionts on the host is unclear.

4.5 Caveats: seasonality and size

Seasonal changes are a feature of some cnidarian-associated microbial communities (26,40). In an outdoor laboratory setting, this is true of *Cassiopea* as well (38). As these samples were taken during the hottest time of the year in the Florida Keys (August), this community may not be representative of other timepoints. Winter communities may harbor different taxa or taxa in different proportions (40). As the internal microenvironment of *Cassiopea* alters over the course of a day with regard to light and dark cycles, it is reasonable to expect that it may become more or less hospitable to some microbiota as temperature and light cycles adjust throughout the year (55).

As *Cassiopea* medusae grow at rates commensurate with their feeding, there is no guarantee that the size of medusae in this study is representative of their age. Developmental changes are known to facilitate changes to microbial communities within other scyphozoans, but size of adults has not been tested (17,23,56). As medusa size and site were not independent (smaller medusae and larger medusae were not usually seen in mixed assemblages), we cannot determine whether size class had an impact.

4.6 Laboratory cultures

Formatted: Indent: First line: 0"

Formatted: Font: Italic

Formatted: Font: Italic

Formatted: Font: 16 pt, Bold

Cassiopea are primarily used for in-lab studies on symbiosis (12,14,88). Some of these use wild-caught individuals, and others use lab-bred cultures, many of which have been held for years. The lab-raised *Cassiopea* used in Rothig et al. 2021 appear to have a completely different microbiome than the wild individuals caught here (19). This potential discrepancy should be considered when using wild-caught or captive-bred individuals, as captive-bred symbiosis and tolerance levels may differ from medusae in the wild. ~~When held in outdoor ponds, the community seems to match wild-type more fully (38).~~ These discrepancies in microbiome are found in other model cnidarians, such as *Exaiptasia* (1). The information on wild *Cassiopea* microbial communities from the Florida Keys presented here may allow researchers working with lab-raised individuals to better contextualize the taxa that they find in cultured medusae.

~~5 CONCLUSION~~

Conclusion

Formatted: Font: 18 pt

The microbiome of *Cassiopea* within the Florida Keys demonstrates strong body compartment discrimination and includes a central group of ~~families~~ ASVs overrepresented in the gut and ~~external~~ bell mucus. All of these families, ~~Endozoicomonadaceae~~Pseudomonadales, Vibrionaceae, and Mycoplasmataceae, are found in other medusae and anthozoans. Despite this overrepresentation of some microbial taxa within the gut, ~~*Cassiopea* lacks a conservatively defined core microbiome~~the *Cassiopea* bell is highly diverse, and overlaps with the communities of the water and substrate in and on which it resides. The consistency of *Cassiopea* microbial communities across the Florida Keys demonstrates that specific taxa are strongly associated with *Cassiopea* and that this can extend across many sites with diverse physical conditions. This work

Formatted: Font: Not Italic

provides a greater understanding of the wild microbiome of the common lab organism *Cassiopea*.

~~6 ACKNOWLEDGEMENTS~~

Acknowledgements

~~This work was funded by Texas Sea Grant and Texas A&M University Galveston.~~

The present work was part of KMM's PhD dissertation. Thank you to Dr. David Retchless for input on figure design. We thank the Florida Fish and Wildlife Conservation Commission for their help and guidance.

~~7 REFERENCES~~

References

1. Brown T, Otero C, Grajales A, Rodriguez E, Rodriguez-Lanetty M. Worldwide exploration of the microbiome harbored by the cnidarian model, Exaiptasia pallida (Agassiz in Verrill, 1864) indicates a lack of bacterial association specificity at a lower taxonomic rank. PeerJ. 2017;2017(5).

2. Van De Water JAJM, Allemand D, Ferrier-Pagès C. Host-microbe interactions in octocoral holobionts - recent advances and perspectives. Microbiome. 2018;6(1):1–28.

3. Meyer JL, Castellanos-Gell J, Aeby GS, Häse CC, Ushijima B, Paul VJ. Microbial Community Shifts Associated With the Ongoing Stony Coral Tissue Loss Disease Outbreak on the Florida Reef Tract. Front Microbiol. 2019;10(September):1–12.

4. Djeghri N, Pondaven P, Stibor H, Dawson MN. Review of the diversity, traits, and

Formatted: Font: 18 pt, Bold

Formatted: Font: 18 pt, Bold

Formatted: Indent: First line: 0.5"

Formatted: Font: 18 pt, Bold

ecology of zooxanthellate jellyfishes. Mar Biol [Internet]. 2019;166(11):1–19. Available from: https://doi.org/10.1007/s00227-019-3581-6

5. Baldassarre L, Ying H, Reitzel AM, Franzenburg S, Fraune S. Microbiota mediated plasticity promotes thermal adaptation in the sea anemone Nematostella vectensis. Nat Commun. 2022;13(1).

6. Ushijima B, Gunasekera SP, Meyer JL, Tittl J, Pitts KA, Thompson S, et al. Chemical and genomic characterization of a potential probiotic treatment for stony coral tissue loss disease. Commun Biol. 2023;6(1).

7. Connelly MT, McRae CJ, Liu PJ, Martin CE, Traylor-Knowles N. Antibiotics Alter Pocillopora Coral-Symbiodiniaceae-Bacteria Interactions and Cause Microbial Dysbiosis During Heat Stress. Front Mar Sci. 2022;8(January):1–18.

8. Pollock FJ, McMinds R, Smith S, Bourne DG, Willis BL, Medina M, et al. Coral-associated bacteria demonstrate phylosymbiosis and cophylogeny. Nat Commun [Internet]. 2018;9(1):1–13. Available from: http://dx.doi.org/10.1038/s41467-018-07275-x

9. Huggett MJ, Apprill A. Coral microbiome database : Integration of sequences reveals high diversity and relatedness of coral- associated microbes. Environ Microbiol Rep. 2019 Jun 1;11(3):372–85.

10. Puntin G, Sweet M, Fraune S, Medina M, Sharp K, Weis VM, et al. Harnessing the Power of Model Organisms To Unravel Microbial Functions in the Coral Holobiont. Microbiol Mol Biol Rev [Internet]. 2022 Oct 26 [cited 2023 Feb 24];86(4). Available from: https://journals.asm.org/doi/10.1128/mmbr.00053-22

11. Ohdera AH, Abrams MJ, Ames CL, Baker DM, Suescún-Bolívar LP, Collins AG, et al. Upside-Down but Headed in the Right Direction: Review of the Highly Versatile Cassiopea xamachana System. Front Ecol Evol. 2018 Apr 9;6:1–15.

12. Mammone M, Ferrier-Pages C, Lavorano S, Rizzo L, Piraino S, Rossi S. High photosynthetic plasticity may reinforce invasiveness of upside-down zooxanthellate jellyfish in Mediterranean coastal waters. PLoS One [Internet]. 2021 Mar 1 [cited 2021 Jun 30];16(3 March):e0248814. Available from: https://doi.org/10.1371/journal.pone.0248814

13. Béziat P, Kunzmann A. Under pressure: Cassiopea andromeda jellyfish exposed to increasing water temperature or lead, cadmium and anthropogenic gadolinium contamination. Mar Biol Res [Internet]. 2022;18(1–2):48–63. Available from: https://doi.org/10.1080/17451000.2022.2066132

14. McGill CJ, Pomory CM. Effects of bleaching and nutrient supplementation on wet weight in the jellyfish Cassiopea xamachana (Bigelow) (Cnidaria: Scyphozoa). Mar Freshw Behav Physiol. 2008;41(3):179–89.

15. Ames CL, Klompen AML, Badhiwala K, Muffett K, Reft AJ, Kumar M, et al. Cassiosomes are stinging-cell structures in the mucus of the upside-down jellyfish Cassiopea xamachana. Commun Biol [Internet]. 2020;3(1):1–15. Available from: http://dx.doi.org/10.1038/s42003-020-0777-8

16. Bakshani CR, Morales-Garcia AL, Althaus M, Wilcox MD, Pearson JP, Bythell JC, et al. Evolutionary conservation of the antimicrobial function of mucus: A first defence against infection. npj Biofilms Microbiomes [Internet]. 2018;4(1):1–12. Available from:

http://dx.doi.org/10.1038/s41522-018-0057-2

17. Klein SG, Pitt KA, Lucas CH, Hung S, Schmidt-roach S, Aranda M, et al. Night-Time Temperature Reprieves Enhance the Thermal Tolerance of a Symbiotic Cnidarian. 2019;6(July):1–16.

18. Lee MD, Kling JD, Araya R, Ceh J. Jellyfish Life Stages Shape Associated Microbial Communities, While a Core Microbiome Is Maintained Across All. Front Microbiol. 2018 Jul 12;0(JUL):1534.

19. Röthig T, Puntin G, Wong JCY, Burian A, McLeod W, Baker DM. Holobiont nitrogen control and its potential for eutrophication resistance in an obligate photosymbiotic jellyfish. Microbiome. 2021;9(1):1–16.

20. Cleary DFR, Becking LE, Polónia ARM, Freitas RM, Gomes NCM. Jellyfish-associated bacterial communities and bacterioplankton in Indonesian Marine lakes. FEMS Microbiol Ecol. 2016;92(5):1–14.

21. Hao W, Gerdts G, Holst S, Wichels A. Bacterial communities associated with scyphomedusae at Helgoland Roads. Mar Biodivers. 2019;49(3):1489–503.

22. Peng S, Hao W, Li Y, Wang L, Sun T, Zhao J, et al. Bacterial Communities Associated With Four Blooming Scyphozoan Jellyfish: Potential Species-Specific Consequences for Marine Organisms and Humans Health. Front Microbiol. 2021 May 5;12(May):1–16.

23. Daley MC, Urban-Rich J, Moisander PH. Bacterial associations with the hydromedusa Nemopsis bachei and scyphomedusa Aurelia aurita from the North Atlantic Ocean. Mar Biol Res. 2016;12(10):1088–100.

24. Weiland-Bräuer N, Neulinger SC, Pinnow N, Künzel S, Baines JF, Schmitz RA. Composition of bacterial communities associated with Aurelia aurita changes with compartment, life stage, and population. Appl Environ Microbiol. 2015;81(17):6038–52.

25. Viver T, Orellana LH, Hatt JK, Urdiain M, Díaz S, Richter M, et al. The low diverse gastric microbiome of the jellyfish Cotylorhiza tuberculata is dominated by four novel taxa. Environ Microbiol. 2017;19(8):3039–58.

26. Basso L, Rizzo L, Marzano M, Intranuovo M, Fosso B, Pesole G, et al. Jellyfish summer outbreaks as bacterial vectors and potential hazards for marine animals and humans health? The case of Rhizostoma pulmo (Scyphozoa, Cnidaria). Sci Total Environ [Internet]. 2019;692:305–18. Available from: https://doi.org/10.1016/j.scitotenv.2019.07.155

27. Stabili L, Rizzo L, Basso L, Marzano M, Fosso B, Pesole G, et al. The microbial community associated with Rhizostoma pulmo: Ecological significance and potential consequences for marine organisms and human health. Mar Drugs. 2020;18(9).

28. Delannoy CMJ, Houghton JDR, Fleming NEC, Ferguson HW. Mauve Stingers (Pelagia noctiluca) as carriers of the bacterial fish pathogen Tenacibaculum maritimum. Aquaculture [Internet]. 2011;311(1–4):255–7. Available from: http://dx.doi.org/10.1016/j.aquaculture.2010.11.033

29. Muffett K, Miglietta MP. Demystifying Cassiopea species identity in the Florida Keys: Cassiopea xamachana and Cassiopea andromeda coexist in shallow waters. PLoS One [Internet]. 2023;18(3):e0283441. Available from: http://dx.doi.org/10.1371/journal.pone.0283441

30. Laramie MB, Pilliod DS, Goldberg CS, Strickler KM. Environmental DNA Sampling Protocol — Filtering Water to Capture DNA from Aquatic Organisms. In: US Geological Survey Techniques and Methods, book 2. Reston, VA: U.S. Geological Survey; 2015. p. 1–15.

31. Pavlovska M, Prekrasna I, Parnikoza I, Dykyi E. Soil sample preservation strategy affects the microbial community structure. Microbes Environ. 2021;36(1):1–7.

32. Callahan BJ, McMurdie PJ, Rosen MJ, Han AW, Johnson AJA, Holmes SP. DADA2: High-resolution sample inference from Illumina amplicon data. Nat Methods. 2016;13(7):581–3.

33. R-Core-Team (Foundation for Statistical Computing). R: A Language and Environment for Statistical Computing [Internet]. 2022. Available from: https://www.r-project.org/

34. McMurdie PJ, Holmes S. Phyloseq: An R Package for Reproducible Interactive Analysis and Graphics of Microbiome Census Data. PLoS One. 2013;8(4).

35. Davis NM, Proctor DiM, Holmes SP, Relman DA, Callahan BJ. Simple statistical identification and removal of contaminant sequences in marker-gene and metagenomics data. Microbiome. 2018;6(1):1–14.

36. Oksanen J, Simpson G, Blanchet F, Kindt R, Legendre P, Minchin P, et al. _vegan: Community Ecology Package_. 2024.

37. Paradis E, Schliep K. ape 5 . 0 : an environment for modern phylogenetics and evolutionary analyses in R. 2019;35(July 2018):526–8.

38. Mikryukov V. metagMisc: Miscellaneous functions for metagenomic analysis [Internet].

Available from: https://github.com/vmikk/metagMisc

39. Taudiere A. MiscMetabar: Miscellaneous Functions for Metabarcoding Analysis [Internet]. 2024. Available from: https://cran.r-project.org/package=MiscMetabar

40. Tsirogiannis C, Sandel B. PhyloMeasures: Fast and Exact Algorithms for Computing Phylogenetic Biodiversity Measures. 2017.

41. Schliep KP. phangorn : phylogenetic analysis in R. 2011;27(4):592–3.

42. Battaglia T. btools: A suite of R function for all types of microbial diversity analyses. 2025.

43. Patil I. Visualizations with statistical details: The "ggstatsplot" approach. J Open Source Softw [Internet]. 2021;6(61)(3167). Available from: doi:10.21105/joss.03167

44. Martinez Arbizu P. pairwiseAdonis: Pairwise Multilevel Comparison using Adonis [Internet]. 2017. Available from: https://github.com/pmartinezarbizu/pairwiseAdonis

45. Kassambara A. rstatix: Pipe-Friendly Framework for Basic Statistical Tests [Internet]. 2023. Available from: https://cran.r-project.org/package=rstatix

46. McLaren M. speedyseq: Faster implementations of phyloseq functions [Internet]. 2025. Available from: https://github.com/mikemc/speedyseq

47. Pebesma E, Bivand R. Spatial Data Science: With applications in R [Internet]. London: Chapman and Hall/CRC; 2023. 352 p. Available from: https://r-spatial.org/book/

48. Macvittie S, Doroodian S, Alberto A, Sogin M, Macvittie S, Doroodian S, et al. Exaiptasia diaphana , following antibiotic exposure. 2024;9(6).

49. Fernandes AD, Reid JNS, Macklaim JM, Mcmurrough TA, Edgell DR, Gloor GB. Unifying the analysis of high-throughput sequencing datasets : characterizing RNA-seq , 16S rRNA gene sequencing and selective growth experiments by compositional data analysis. 2014;1–13.

50. Holmes I, Harris K, Quince C. Dirichlet Multinomial Mixtures : Generative Models for Microbial Metagenomics. 2012;7(2).

51. Morgan M. DirichletMultinomial: Dirichlet-Multinomial Mixture Model Machine Learning for Microbiome Data [Internet]. 2024. Available from: https://doi.org/10.18129/B9.bioc.DirichletMultinomial

52. Lahti L, Shetty S. microbiome R package. 2019.

53. Neu AT. Defining and quantifying the core microbiome : Challenges and prospects. 2021;118(51):1–10.

54. Bonacolta AM, Connelly MT, Rosales SM, del Campo J, Traylor-knowles N. The starlet sea anemone , Nematostella vectensis , possesses body region-specific bacterial associations with spirochetes dominating the capitulum. FEMS Microbiol Lett. 2021;(August 2020):1–9.

55. van Oppen MJH, Blackall LL. Coral microbiome dynamics, functions and design in a changing world. Nat Rev Microbiol [Internet]. 2019;17(9):557–67. Available from: http://dx.doi.org/10.1038/s41579-019-0223-4

56. Agostini S, Yoshimi S, Higuchi T, Casareto BE. Biological and chemical characteristics of the coral gastric cavity. Coral Reefs. 2011;(March).

57. Bollati E, Hughes DJ, Suggett DJ, Raina J-B, Kühl M. Microscale sampling of the coral gastric cavity reveals a gut-like microbial community. bioRxiv [Internet]. 2024;2024.05.20.594925. Available from: https://www.biorxiv.org/content/10.1101/2024.05.20.594925v1%0Ahttps://www.biorxiv.org/content/10.1101/2024.05.20.594925v1.abstract

58. Carabantes N, Cerqueda-García D, García-Maldonado JQ, Thomé PE. Changes in the Bacterial Community Associated With Experimental Symbiont Loss in the Mucus Layer of Cassiopea xamachana Jellyfish. Front Mar Sci. 2022;9(May):1–13.

59. Wu W, Lei J-N, Mao Q, Tian Y-Z, Shan H-W, Chen J-P. Distribution, Vertical Transmission, and Cooperative Mechanisms of Obligate Symbiotic Bacteria in the Leafhopper Maiestas dorsalis (Hemiptera, Cicadellidea). Insects. 2023;1–13.

60. Sharp KH, Pratte ZA, Kerwin AH, Rotjan RD, Stewart FJ. Season, but not symbiont state, drives microbiome structure in the temperate coral Astrangia poculata. Microbiome. 2017;5(1):120.

61. Hammer TJ, Sanders JG, Fierer N. Not all animals need a microbiome. FEMS Microbiol Lett. 2019;366(10):1–11.

62. Riviere M La, Roumagnac M, Garrabou J, Bally M. Transient Shifts in Bacterial Communities Associated with the Temperate Gorgonian Paramuricea clavata in the Northwestern Mediterranean Sea. PLoS One. 2013;8(2).

63. Neave MJ, Apprill A, Ferrier-Pagès C, Voolstra CR. Diversity and function of prevalent symbiotic marine bacteria in the genus Endozoicomonas. Appl Microbiol Biotechnol [Internet]. 2016;100(19):8315–24. Available from: http://dx.doi.org/10.1007/s00253-016-

7777-0

64. Tinta T, Kogovšek T, Klun K, Malej A, Herndl GJ, Turk V. Jellyfish-associated microbiome in the marine environment: Exploring its biotechnological potential. Mar Drugs. 2019;17(2):1–33.

65. Maher RL, Schmeltzer ER, Meiling S, McMinds R, Ezzat L, Shantz AA, et al. Coral Microbiomes Demonstrate Flexibility and Resilience Through a Reduction in Community Diversity Following a Thermal Stress Event. Front Ecol Evol. 2020;8(October):1–15.

66. Pogoreutz C, Oakley CA, Rädecker N, Cárdenas A, Perna G, Xiang N, et al. Coral holobiont cues prime Endozoicomonas for a symbiotic lifestyle. ISME J. 2022;16(8):1883–95.

67. Neave MJ, Michell CT, Apprill A, Voolstra CR. Endozoicomonas genomes reveal functional adaptation and plasticity in bacterial strains symbiotically associated with diverse marine hosts. Sci Rep. 2017;7(December 2016):1–12.

68. McDevitt-Irwin JM, Baum JK, Garren M, Vega Thurber RL. Responses of coral-associated bacterial communities to local and global stressors. Front Mar Sci. 2017;4(AUG):1–16.

69. Neulinger SC, Gärtner A, Järnegren J, Ludvigsen M, Lochte K, Dullo WC. Tissue-associated “Candidatus mycoplasma corallicola” and filamentous bacteria on the cold-water coral Lophelia pertusa (Scleractinia). Appl Environ Microbiol. 2009;75(5):1437–44.

70. Sweet MJ, Bulling MT. On the importance of the microbiome and pathobiome in coral health and disease. Front Mar Sci. 2017;4(January):1–11.

71. Hofmann DK, Neumann R, Henne K. Strobilation, budding and initiation of scyphistoma morphogenesis in the rhizostome Cassiopea andromeda (Cnidaria: Scyphozoa). Mar Biol. 1978;47(2):161–76.

72. Neumann R. Bacterial Induction of Settlement and Metamorphosis in the Planula Larvae of Cassiopea andromeda (Cnidaria: Scyphozoa, Rhizostomeae). Vol. 1. 1979.

73. Kramar MK, Tinta T, Lučić D, Malej A, Turk V. Bacteria associated with moon jellyfish during bloom and post-bloom periods in the Gulf of Trieste (northern Adriatic). PLoS One. 2019;14(1):1–21.

74. Tinta T, Kogovsek T, Malej A, Turk V. Jellyfish Modulate Bacterial Dynamic and Community Structure. PLoS One. 2012;7(4):1–10.

75. Pollock FJ, Wilson B, Johnson WR, Morris PJ, Willis BL, Bourne DG. Phylogeny of the coral pathogen Vibrio coralliilyticus. Environ Microbiol Rep. 2010;2(1):172–8.

76. Ben-Haim Y, Thompson FL, Thompson CC, Cnockaert MC, Hoste B, Swings J, et al. Vibrio coralliilyticus sp. nov., a temperature-dependent pathogen of the coral Pocillopora damicornis. Int J Syst Evol Microbiol. 2003;53(1):309–15.

77. Richards GP, Watson MA, Needleman DS, Church KM, Häse CC. Mortalities of Eastern And Pacific oyster larvae caused by the pathogens Vibrio coralliilyticus and Vibrio tubiashii. Appl Environ Microbiol. 2015;81(1):292–7.

78. Emery MA, Beavers KM, Van Buren EW, Batiste R, Dimos B, Pellegrino MW, et al. Trade-off between photosymbiosis and innate immunity influences cnidarian’s response to pathogenic bacteria. Proc R Soc B Biol Sci. 2024;291(2032):20240428.

79. Thomé PE, Rivera-Ortega J. Specific antibacterial activity against potential pathogens and re-straining of larvae settlement from a pigmented Pseudoalteromonas strain isolated from the jellyfish Cassiopea xamachana. Int Aquat Res. 2022;14(2):95–105.

80. Bright M, Espada-hinojosa S, Lagkouvardos I, Volland J, Hole W. The giant ciliate Zoothamnium niveum and its thiotrophic epibiont Candidatus Thiobios zoothamnicoli : a model system to study interspecies cooperation. 2014;5(April):1–13.

81. Rinke C, Schmitz-esser S, Stoecker K, Nussbaumer AD, Vanura K, Wagner M, et al. " Candidatus Thiobios zoothamnicoli ," an Ectosymbiotic Bacterium Covering the Giant Marine Ciliate Zoothamnium niveum. 2021;72(3):2014–21.

82. Vekeman B. Methanotrophic microbiomes from North Sea sediment.

83. Song J, Hwang J, Kang I, Cho JC. OPEN A sulfate - reducing bacterial genus , Desulfosediminicola gen . nov ., comprising two novel species cultivated from tidal - flat sediments. Sci Rep [Internet]. 2021;1–13. Available from: https://doi.org/10.1038/s41598-021-99469-5

84. Engelen AH, Aires T, Vermeij MJA, Herndl GJ, Serrão EA, Frade PR. Host differentiation and compartmentalization of microbial communities in the azooxanthellate cupcorals Tubastrea coccinea and Rhizopsammia goesi in the Caribbean. Front Mar Sci. 2018;9(NOV):1–9.

85. Meirelles LA, Newman DK. Both toxic and beneficial effects of pyocyanin contribute to the lifecycle of Pseudomonas aeruginosa. Mol Microbiol. 2018;110(6):995–1010.

86. Carlson CA, Ingraham JL. Comparison of Denitrification by Pseudomonas stutzeri ,

Pseudomonas aeruginosa , and Paracoccus denitrificans. 1983;45(4):1247–53.

87. Huo Y, Xu X, Li X, Liu C, Cui H, Wang C, et al. Ruegeria marina sp . nov ., isolated from Marine Sediment. 2011;347–50.

88. Klein SG, Pitt KA, Carroll AR. Reduced salinity increases susceptibility of zooxanthellate jellyfish to herbicide toxicity during a simulated rainfall event. Environ Pollut [Internet]. 2016;209:79–86. Available from: http://dx.doi.org/10.1016/j.envpol.2015.11.012

89. Hyun DW, Shin NR, Kim MS, Oh SJ, Kim PS, Whon TW, et al. Endozoicomonas atrinae sp. nov., isolated from the intestine of a comb pen shell Atrina pectinata. Int J Syst Evol Microbiol. 2014;64(PART 7):2312–8.

90. Sunagawa S, Woodley CM, Medina M. Threatened corals provide underexplored microbial habitats. PLoS One. 2010;5(3):1–7.

Supplementary Information

Formatted: Font: 18 pt, Bold

8 SUPPLEMENTARY INFORMATION

Supplement A1: Full length 16S rRNA gene identities

Three samples were sent to CD Genomics for full length 16S on PacBio Sequel using universal 16S primers (F: AGRGTTTGATYNTGGCTCAG; R:TASGGHTACCTTGTTASGACTT). Samples sent for full length 16S rRNA sequencing included gastrovascular cavity swab extractions from medusae Marathon Key GVC 30 and Key West GVC 96 and GVC 120. As the usable reads returned from this were low (597-12520 per sample), these full-length identities are not discussed in the main text. For further details, please refer to the appendix (A1).

Full length 16S rRNA gene sequences (BioProject: PRJNA1020446) were run on mothur v1.48.0 with deviation from the MiSeq SOP on trim length. ASV cutoff was set to five differences, and ASV representative sequences were compared against the NCBI GenBank (Sept 24, 2023) database using BLAST to find nearest matches by e-value.

In order to identify core microbial groups to a lower taxonomic level, three internal medusa gastrovascular swabs (medusae Kmuffet_96, Kmuffet_120 and Kmuffet_30) from Marathon Key (MK) and Key West (GB) were sequence for full length 16S rRNA gene (see S1 for location of samples). As the primers were not chloroplast exclusionary, the Key West sites produced few useable sequences (Kmuffet_96: 597 and Kmuffet_120: 1097). The Marathon Key medusa (Kmuffet_30), likely bleached at time of collection, returned 12,520 non-chloroplast sequences. The Mycoplasma-like full length 16S rRNA gene sequence (Genbank Acc. No. OR592270) from Marathon Key and Key West has no closely related sequences currently identified (greatest identity 88.43% to sequence HQ393440). The *Endozoicomonas* strain from both Marathon Key and Key West (Genbank Acc. No. OR592271) has 99.93% identity to *Endozoicomonas atrinae* strain WP70(T) collected from the intestine of South Korean *Atrina pectinata* (Accession NR_134024)(89) and 99.80% identity (<5 nucleotide differences) with isolates collected from *Gorgonia ventalina* (GenBank Acc. No. GU118345) in Bocas del Toro, Panama (90). *Endozoicomanas* and *Mycoplasma*-like isolates were the only two groups to have ASVs with over 100 sequences.

S1~~-10~~ Tables: (in Supplementary Excel Workbook)

Supplementary Table S~~0~~1: Roster of collected medusae

Supplementary Table S2: Read processing tracking table

Supplementary Table S3: Representative sequences of all ASVs from the dataset

Supplementary Table S4: ASVs orderd by abundance in each sample type

Supplementary Table S5: Taxonomy of top 1000 ASVs in dataset

Sfig 1: Rarefaction curves of all samples

~~Supplementary Table 02: Ordered MOTHUR commands used for dataset~~

~~Supplementary Table 03: Sequences retained across analysis pipeline by sample~~

~~Supplementary Table 04: Family level taxonomic identity of all phylotypes found in~~

~~Supplementary Table 05: Family level sequence list for each phylotype and sample~~

~~Supplementary Table 06: Family level sequence list for each phylotype and sample organized as proportion of sequences per sample~~

~~Supplementary Table 07: Dirichlet partitions of gastrovascular cavity samples~~

~~Supplementary Table 08: Likelihoods of all Dirichlet models of gastrovascular cavity samples~~

~~Supplementary Table 09: Final Dirichlet model parameters~~

~~Supplementary Table 10: Dirichlet comparitive impact of community members~~

~~S11: Order level bar plots grouped by location~~

~~S12: Mosaic plot of Dirichlet partition identity (community type) by site.~~

935

Response to editor and reviewers

Dear editor and reviewers,

After careful review of your suggestions and criticisms, the methodological and statistical analyses in this manuscript have been revisited, redone, and in the process greatly improved. Many of your broad concerns in these areas are addressed with a significantly expanded methods section.

Sincerely,

KMM

Response to editor:

Thank you for your considered feedback.

In addition to the improved manuscript, please note these specific changes:

1. Inclusion of the funder role statement along with the manuscript
2. Corrected Funding statement in cover letter
3. Fixed formatting in manuscript
4. Added ethics statement

Comments to reviewer 1:

Thank you for your careful feedback. After substantial revision to the backbone of this work, we hope you will find all of your comments duly taken into consideration. Please see specific corrections in addition to the broader overhaul in the response below.

1. *Abstract needs to mention consistent results from across sampling sites.*
   a. Thank you for your feedback. This has been corrected.
2. *Clarify inclusion of C. andromeda in analysis.*
   a. In the text (ln 146) it now clarifies that *C. xamachana* only are used for downstream analyses with the exception explicitly comparing the two mitotypes (ln 298).
3. *Standardize "benthic" sample name*
   a. Thank you for your suggestion. All substrate samples are now referred to as substrate as opposed to benthic.
4. *Figure 2*
   a. Figure two has been replaced with an improved version that includes your suggestions.
5. *Figure 3*
   a. Figure 3 has been replaced and now is a chart of Shannon and observed diversity
6. *Citation for Dirichlet multinomial analysis*
   a. The lack of reference to Holme's original work has been rectified.

7. *Line 227: claim about clustering distinction*
    a. Thank you. This claim has been removed and has been replaced with Faith's D and more appropriate accompanying statistical tests.
8. *Core microbiome information incomplete*
    a. Thank you for the feedback. This section was indeed incomplete and confusing. It has been replaced with analyses from the "microbiome" r package that are more appropriate.
9. *Lack discussion of external microbiome*
    a. Thank you for this comment. There is now a section discussing this within the paper.
10. *Seasonality and size comments unnecessary*
    a. Thank you for your feedback, this section has been removed.

Specific comments:

- *Line 33: "These communities" please specify you are referring to "microbial communities"*
    - This issue has been noted and corrected.
- *Line 38: replace "clade" with "Family"*
    - This issue has been noted and corrected.
- *Line 47: the statement about the tolerance of Cassiopea to intense temperature fluctuations needs a reference*
    - This issue has been noted and corrected.
- *Line 63: I do not understand the purpose of the statement: "Pathogenesis is often a focus…"*
    - This statement has been clarified and corrected.
- *Line 150-151: Why were you not able to use the Siva 138 database?*
    - This work now uses the most up-to-date silva database.
- *Line 165: "benthic OR water" should not be "water AND sediment"?*
    - This issue has been noted and corrected.
- *Line 216: Please revise "Family richness was significantly lower within medusae"*
    - This line has been corrected.
- *Line 227: after referring to your results, it would be good to add a figure number*
    - Figure numbers are now added in correct positions.
- *Lines 280-282: this statement has no support. It could work the other way, going from the exterior but remaining an important component in the internal cavity. Please revise.*
    - This error has been noted and replaced.
- *Lines 299-300: I do not understand why you mention this statement, since in your results no identification of "obligate" taxa was made for the GVC. Please revise.*
    - This statement has been noted and revised.
- *Line 314: a space is needed before the parenthesis*

    - This issue has been corrected.
- *Lines 317-319: is not clear what point are you trying to make.*
    - Thank you for the feedback, this statement has been removed and replaced with one of greater clarity.
- *Line 326: use "Family" rather than "clade"*
    - Corrected.
- *Line 327: orphan phrase? "…appear to harbor the microbiome, …"*
    - *Thank you for the feedback, this statement has been clarified and reworded.*
- *Line 381: I suggest changing "lacks a conservatively defined core microbiome" for "in our samples, no core microbiome could be demonstrated"*
    - *This statement has been removed and replaced after reanalysis.*

Comments to reviewer 2:

The authors thank reviewer 2 for their supportive feedback. The poor explanation of statistical tests and underanalysis of the dataset at hand presented key failings that have been carefully corrected in the updated version. We hope that these revisions make this work more meaningful and improve readability.

Reviewer #2: The authors study the microbiome composition of two upside-down jellyfish species in the Florida Keys using 16S rRNA barcoding. The paper describes the main bacterial taxa in different locations and describes the microbiome composition focusing on an understudied group. They briefly compare these findings with the existing literature for other anthozoan groups.
I found the study interesting, but the results seem overstated, and some important information is missing. For example, the title does not accurately reflect the study's findings. It should better represent the study's scope and avoid claiming general patterns that the data does not appear to support. My major concern is the lack of explanation in the statistical analyses. I think improving this part will help to better understand the analyses and evaluate the content of the manuscript.

Responses to specific comments:

- *Overall, there is a lack of information on sample sizes throughout the study. This omission makes it difficult to assess the balance of the study design or to understand how many samples are being compared in each analysis. Sample size details should be included in the figures, methods, and results sections.*

    - Thank you for your comment. The sample size information is now added to the first table and where relevant in other sections.
- *The authors use two different preservatives—ethanol and DESS—that appear to be combined without distinction. I recommend including a statement in the methods on how this might affect microbiome composition. If the authors tested for potential effects, the results should be included, or they should reference studies clarifying whether these preservatives could influence the results.*
    - Ethanol was not used for any downstream analyses, a statement that has now been added to the methods section to clarify.
- *Table S1: Please provide descriptions for abbreviations used to aid comprehension.*
    - Clarification has now been added to the supplementary information.
- *Table 1: Please add the sample sizes per site.*
    - Corrected
- *L129-135: If PacBio sequences were not used in the study, I recommend removing this from the methods section.*
    - Corrected.
- *L157: Please describe the statistical analyses clearly enough to allow reproducibility. This section lacks essential details.*
    - Corrected.
- *Figure legends need explanations of the content. They are currently limited to single-line titles, which restricts comprehension. I recommend revising the figure design, e.g. Figure 1.*
    - Corrected
- *L226: "significantly different".*
    - This statement has been replaced and clarified.
- *Figure 4: The stress level is high. How balanced is this comparison? Although the figure appears to show a comparison analysis, the text refers to a pairwise comparison. Could the authors clarify the type of analysis used here?*
    - Thank you for your comment. This analysis has been replaced witn a more appropriate MDS of Euclidean distance from the corrected data.
- *The results section presents many analyses and tests that were not introduced in the methods section. Additionally, the results refer to supplementary tables without legends, making it difficult to assess the accuracy of these tests. I recommend a more detailed explanation of the analyses conducted.*
    - This consistent issue has been corrected and all analyses are now listed in the revised version of the manuscript.
- *Figure 2: The y-axis label is missing.*
    - This figure has been replaced with a more appropriate figure.
