# Supplementary material for: Florida Keys Cassiopea host benthos-like external microbiomes and a gut dominated by Vibrio, Endozoicomonas and Mycoplasma
Source: PLoS One. 2025 Aug 12;20(8):e0330180. doi: 10.1371/journal.pone.0330180 (PMC12342295; doi:10.1371/journal.pone.0330180)
Supplement: Attachment — Submitted filename: Specific concerns_PONE_manuscript_04_21_25.docx.pdf [file pone.0330180.s007.pdf]

Response to editor and reviewers:

To all,

Thank you for taking the time to review this manuscript. Your suggestions and critiques have been very helpful. The majority of changes in this round of the manuscript cover refinement of captions, movement of extraneous figures to the supplementary materials, and greater detail in some sections of the methodology section. Hopefully the in-text modifications improve the clarity and readability of the text.

Sincerely,

KMM

Reviewer 1

To Reviewer 1,

Thank you for your specific critiques. As you have suggested, the ethanol preserved samples have been removed from the methods section in order to improve clarity. Table #1 has been separated to provide adequate space for definitions and the term “GVC” is now clarified earlier. Additional analyses of the physical parameters have now been added to the supplements, as is addressed in the specific response below. Additionally, the limited nature of this sampling has now been reiterated in the core microbiome section.

Thank you,

Kaden Muffett

Specific points

1. Briefly, since the samples preserved in alcohol did not give good results, these should be removed from the manuscript.

   The authors accept this suggestion and have modified the text accordingly.

2. Table 1 needs work with headings, column spaces, and some definitions.
3. The GVC term should be introduced in Methods.
   Thank you for bringing up this oversight. The correction has been made.
4. The authors collected information on physical parameters, but such information was not relevant in their analyses, correct?
   Yes, as these were point-in-time measurements, it was deemed imprudent to include them in the primary analysis. Redundancy analysis plots (RDA) including

these features of interest have now been added to the supplementary materials for those interested. They are presented in this table to inform others' interpretation of these data, as factors associated with these have been demonstrated important in other microbiomes.

5. Finally, in the Results section referring to Core Microbiome, the authors need to stress that the sampling was done in one month within a single year.
   Thank you for the suggestion, this has now been specified in the results.

Reviewer #3

To reviewer #3,

Thank you for your review of this manuscript. Your suggestions have improved the precision and clarity of the text. In addition to your specific comments, you also indicated some more general inquiries regarding the nature of these microbiomes. While I am not capable of speaking to the function of the *Cassiopea* microbiome with these data, I do think that these questions are worth pursuing with other study designs.

Please see the responses to all of your specific requested edits below.

Sincerely,

KMM

Specific comments

1. Title: because both internal and external microbiomes were investigated, the words "gastric microbiome" limits the scope of the study, which is broader. My suggestion is to alter it.
   Thank you for your suggestion. The title has been modified to include both external and internal data.
1. Keywords: the word "Cassiopea" is already present in the title. Please, remove it from the keywords section and replace it with another one.
   Corrected.
2. Lines 28-29: not all stony corals, octocorals and sea anemones are symbiotic. I recommend replacing "the well-known" with "some".
   Corrected.
   Line 43: please, replace "hot" with "warm". Also, write the full name of C. andromeda, including authority and year of publication.
   Corrected.
3. Line 45: why is having a "sticky interface" significant? This phrase could be a little more informative.

Corrected. The text now specifies that this is a barrier between host and environment.

4. Line 47: please, add “SOME” stony corals and specify how much heat can Cassiopea tolerate as well as max and min temperature fluctuations.
   Corrected, specific tolerance details added.
5. -Line 98: please, write GVC in full. The same should be applied to caption of figure 1 and 3.
   Thank you, this issue has been corrected.
6. Line 117: my suggestion is to add "amplification" as well.
   Corrected.
7. Line 122: did you mean amplification?
   Corrected.
8. Line 137: great work at providing the codes and describing each command. Congrats!
   Thank you for your praise.
9. Line 139: please, write ASV in full the first time.
   Corrected.
10. Line 143: out of curiosity…Did you face any limitations in the completeness of the Silva database? Do you think that using additional databases would have provided a different outcome?
    It is certainly possible. The Silva database has made substantial strides in the last several years when it comes to marine taxa, so issues I had with ID in the first tests (2021) are largely absent now. RDP certainly was considered, but the comparability of Silva across studies is currently unbeatable. I also considered using the expanded McCauley classifier, but decided that, should someone want perfect taxonomic comparability to another study, they will likely need to reclassify anyway. Such is the reality of amplicon sequencing, and also our constantly in-flux marine microbial genera.
11. Line 151: GVC should be written in full since it's the first time it appears in the text.
    This has now been corrected earlier in the text (methodology section)
12. Line 152: please, rewrite it as “34.808 taxa” to keep numbers standardized.
    Corrected.
13. Line 179: what about the other 21 specimens?
    The other 21 included medusae under 3 cm diameter (9 indv), and samples that did not fit on the final sequencing run with the resources available. This has now been specified in the text.

14. Line 181: please, remove "GVC".
    Corrected.
15. Line 182: please, add authority and year of publication.
    Corrected.
16. Line 183: in total, 55 specimens were sampled, but only 34 were analyzed and out of these 4 were not identified as C. xamachana. Resulting in 30 specimens. Is that correct? If so, what happened to the other 17 specimens. That is not clear. What is the actual number of analyzed specimens?
    Thank you, the description of the dataset has been standardized across the paper. It is now clear that 55 were collected, of these 46 were swabbed, of these 34 samples were sequenced, and of these 30 were *C. xamachana*.
17. Line 196: please, remove "(top)" and "(bottom)".
    Corrected.
18. Line 205: please, remove "(GVC)"
    Corrected.
19. Line 228: please write ASVs in full every time it appears for the first time in a caption.
    Corrected.
20. Line 229: displayed?*
    Corrected.
21. Caption of figures 3 and 6: please, rewrite it explaining what each abbreviation mean (HHP, CK, LW…)
    Thank you for your feedback. The explanations are now available within table 1.
22. Line 243: please, invert to "location (b+c)". Line 245: please, invert to "GVC (b) and bell (c) alpha diversity", Line 256: please, write "PCA" in full. The same to line 352.
    Corrected.
23. Line 272: please, add a single space after "of" (e.g.: of > 0.1%).
    Corrected.
24. Line 280: Cassiopea should be in italics.
    Corrected.
25. Caption of figure 6: please, explain each abbreviation and write GVC in full the first time it appears in the caption. The same should be applied to all captions (tables and figures).
    This has been corrected across captions.
26. Line 288: please, correct the overspacing.
    Corrected.

27. Lines 318 - 322: why some organisms are more abundant than others depending on the area where they were sampled (e.g. bell, gvc)? Could this have anything to do with their functional role?
    It is entirely possible that this is related to functional role, however the assessment of functional role from 16S data, especially from divergent taxa, is difficult and error prone. This question is outside the scope of this paper but certainly an interesting one.
28. Line 353: “were” = where?*
    Corrected.
29. Line 360: Cotylorhiza tuberculata (Macri, 1778). Authority and year of publication should be added the first time a species name is mentioned. Please, revise all species names cited throughout the manuscript.
    Corrected.
30. - Line 436: please, see author guidelines for how studies should be cited. For example, here, a comma is used after “et al.”, but the same is not applied to the citation above.
    The erroneous citations have been modified, thank you for bringing this to my attention.
31. Line 446: remove “a” before “either”.
    Corrected.
32. Line 452: if the Cassiopea’s external microbiome does not differ from the sediment microbiome, does it mean that Cassiopea does not have exclusive external microbiome?
    This is a reasonable interpretation of the data. This community is enriched in *Endozoicommonas* relative to the benthos, as discussed elsewhere, but aside from this, the community is very similar to that of surrounding substrates.

Reviewer #4

Reviewer 4,

Thank you for taking the time to review this manuscript. You have raised two main concerns about the structure of this paper, the first being that we are not fully able to parse the impact of location with the samples collected, and secondly, that this paper has lapses in explanations of methodological purpose.

To the first point, I agree that the sample sizes are not adequate across all sites to justify this being the driver of the paper. In reality, what we have the ability to analyze is only the ways in which gastrovascular cavities and bells show consistencies. In order to bring the introduction into line with this, the introduction has been modified, including

both stressing that this is an exploratory paper and that the focus is to identify the core components of the GVC and bell communities.

To the second point, I have modified the paper such that the impetus for specific tests are clearer, and switched out the less informative DMM figure with a more informative one. Where there were too many figures, they have been moved to the supplementary materials, and where there was inadequate detail in the supplements (eg Shannon diversity), the supplementary materials have been expanded.

In addition to these, in alignment with your suggestion, Bray-Curtis is now used throughout the text.

Thank you for your feedback,

Kaden Muffett

Specific comments:

1. Since microbiome sequencing is a data-driven method, in which ASVs are sequence constructs derived from the original samples, a data-driven pipeline is necessary to ensure that scientific hypotheses are tested without bias.
   Thank you for this criticism. In this case, this is an exploratory study of this community, and while I understand the concern for bias, this study is simply a hypothesis generation mechanism. I accept your criticism as this: The aim of this paper specifically states "compare the microbial communities of Cassiopea across sites of the Florida Keys". I agree that I do not have adequate replicates to fully elucidate differences between sites. As such, I have changed the wording of this aim to reflect the core of what this work has accomplished, "The aim of this work is to identify the core components of the internal and external mucosal microbiomes of *Cassiopea* within the Florida Keys.".
2. This sentence also includes a typo: archaeal (not archael). A similar word choice appears to have been made in the title. Overall, the writing needs improvement.
   The typos throughout the text have been corrected.
3. Another issue is that there are too many figures in the main text. Some of them, such as Figure 1, could be moved to the Supplementary Files. The same applies to Table 1.
   Thank you for your suggestion. While I understand that some of these figures and tables may seem extraneous, this paper is serving a diverse group of scientists, and other reviewers have been and remain interested in the inclusion of this table.
    a. Many of the remaining figures should also be improved in terms of quality and explanation, as they currently appear without adequate context.

Thank you for this criticism. The figure captions have been modified to better explain the contents of each figure.

b. Why were the main phyla and classes depicted?
The main phyla and classes are depicted in this paper because of precedent. For past coral (Dunphy et al 2019 (https://doi.org/10.1038/s41598-019-43268-6), Hernandez-Agreda 2018 (https://doi.org/10.1128/mbio.00812-18)) and scyphozoan microbiome work (Ohdera et al 2024 (https://doi.org/10.1371/journal.pone.0298002) , Peng et al 2021 (https://doi.org/10.3389/fmicb.2021.647089)), this has been a common display mechanism. This figure is displayed for easy communication of the major groups found in these samples within this study.

c. Why use Euclidean distance, Faith's PD, or calculate the core microbiome?
Thank you for your questions. In this case, I did not fully consider the impact of Euclidean diversity. I have changed all beta diversity to Bray-Curtis throughout the text. For Faith's PD, I have now specified the following in the methods section "In order to identify the degree to which diversity spanned primarily shallow or deep phylogenetic distances, Faith's phylogenetic diversity was calculated for all samples and sample types. Faith's phylogenetic diversity was computed.." . For the core microbiome, an explanation of choice is now provided in the methods "In order to identify taxa consistently present across samples of a given type that may be rare, core microbiome was calculated for internal and external communities of *Cassiopea*. Core microbiome was computed.."

d. What is the Dirichlet multinomial mixture (DMM), and why was it used?
Dirichilet multinomial mixture analysis tests an ideal number of populations for a provided diverse population set. The purpose of this has now been clarified in the methods.
"In order to identify whether a gastrovascular/ bell split community partitioning was appropriate for all samples, and identify subcommunity level enterotypes within Cassiopea internal and external microbiomes, Dirichlet Multinomial mixture analysis, as described by Holmes and implemented in the DirichletMultinomial package v 1.48 was used for Cassiopea – derived samples (52,53). "

4. The rationale behind assessing the substrate and water samples is unclear. Grouping eight samples (one per site) and comparing them with thirty GVC and bell samples in Figure 2 seems counterintuitive. Please explain this logic more clearly to the reader. A simple Venn diagram of shared ASVs would more

effectively depict the percentage of ASVs shared among bell, water, substrate, and GVC samples. I could not extract this information from Table 2.
The inclusion of the substrate and water in figure 2 and table 2 serve as comparison tools to the microbiomes discussed. An upset plot of group to group overlap is now included.

5. Still regarding Figure 2, an "unknown" category should be added to group ASVs classified only as "Bacteria." This would create a bar that fills each sample (or group of samples) to 100%, aligning the bars in the plot. It is also helpful to limit the figure to the x most abundant taxa to avoid cluttering the legend with indistinguishable shades of similar colors.
Thank you for your suggestion. The colors and design for this have been altered as suggested to aid comprehensibility.
6. I don't think the in-text Shannon values (L234–L240), or any diversity metric, are informative as currently presented. These should be included in a Supplementary Table with appropriate measures of dispersion and statistical tests, indicating statistically distinct groups with letters.
Thank you for the suggestion. A table of Shannon diversity metrics is now available in the supplementary materials.
7. Why was only the GVC core microbiome analyzed and then searched in other samples (Figure 6)? What do the abbreviations GB, CK, VP, BKP, etc., refer to in this figure? These should be defined in the legend. Also, refer to the sampling table that should include these abbreviations. A map or schematic of these sites would be valuable for readers unfamiliar with the study area. I encourage the authors to consider improved ways to present this result. Figures 7 and 8 could be merged and moved to the Supplementary Material.
The specificity of this gastrovascular cavity is of interest to the field. The explicit separation of internal and external samples is rarely done for cnidarian samples. As such, making it clear that the core of this community is not represented in other sample times is a key goal of this paper.

8. Regarding the DMM, no figure shows the model fit against the number of components (clusters), as stated in L309: “model probability value was optimal for two communities, one GVC and one bell.” This result should be presented to justify grouping the samples by tissue, as previously critiqued.
Additional details on this are now provided in-text and supplementary figs have been added.
9. I cannot read the text in Figure 10. Please explain the star plot in the Results section.

Thank you for your comment. The reasoning behind this figure has been expanded in the results section.

10. The “Host medusa species identity” section should not be the final result, as it is disconnected from the rest of the section. If the intent was to show that there is no microbiome distinction between CA and CX, this could be stated at the beginning of the Results and shown in the Supplementary Material.
    Thank you for your suggestion. This section has been removed and moved to the supplements.
11. What is meant by “Retention metrics” (L144)? Does this refer to the number of reads retained after preprocessing? Please clarify this for the reader.
    Thank you for your comment. This has now been clarified in the text.
12. The provided GitHub repository (https://github.com/kadenmuffett/Cassiopea-Code-Microbiome-Repository) is not publicly accessible. Either correct this or provide the code as a Supplementary File.
    While I have not been able to replicate this issue, a copy of the code is now included in the supplementary material as well.
13. Why are WTR and substrate samples not described in the Sampling section alongside bell and digestive cavity samples (L103–L111)?
    Water and substrate samples are described immediately prior to GVC and bell samples. 1L of water was filtered through a 0.2um filter, then preserved in DESS. Sediment samples were preserved in DESS directly.
14. Abbreviations used throughout the manuscript should be introduced in this section, before they appear in the Results. For example, GVC is mentioned for the first time in the Table 1 legend without prior definition.
    This has been corrected.
15. Why is Bray-Curtis mentioned in the Methods (L161) but not in the Results? Remove it if it was not used. Please consider my suggestion above regarding this metric.
    Thank you for your comment. Euclidean has been replaced with Bray-Curtis throughout the text.
16. Figures 1, 2, and 7 are of poor quality — the text is unclear. Were they uploaded as vector files? In Figure 2a, the text within the box is unreadable.
    Please be assured that the image quality displayed in the reviewer pdf is poor quality and not representative of the quality of the uploads.
17. Also, regarding the Results, current phylum-level nomenclature should be used alongside classical names (L192–L193). For example, Pseudomonadota (formerly Proteobacteria).

Thank you. Bacterial phylum names have been modified to reflect this.

18. While I don’t prefer sections within the Discussion, the text is interesting and connects the findings to the scientific literature. Including more explicit references to the underlying Results would further improve it.

    Thank you, some additional connection to the test has been added to improve congruity across sections.

19. Please cite SILVA database (10.1093/nar/gks1219) (L144)

    This has been corrected.

20. Refraction curves? (L155). Rarefaction

    This typo has been corrected.
