# Supplementary material for: Florida Keys Cassiopea host benthos-like external microbiomes and a gut dominated by Vibrio, Endozoicomonas and Mycoplasma
Source: PLoS One. 2025 Aug 12;20(8):e0330180. doi: 10.1371/journal.pone.0330180 (PMC12342295; doi:10.1371/journal.pone.0330180)
Supplement: Attachment — Submitted filename: PONE-D-24-22287_R2_reviewed.pdf [file pone.0330180.s006.pdf]

PLOS ONE

Florida Keys Cassiopea host benthos-like external microbiomes and a gut dominated by Vibrio, Endozoicommonas and Mycoplasma

--Manuscript Draft--

**Manuscript Number:**	PONE-D-24-22287R2
**Article Type:**	Research Article
**Full Title:**	Florida Keys Cassiopea host benthos-like external microbiomes and a gut dominated by Vibrio, Endozoicommonas and Mycoplasma
**Short Title:**	Microbiome of Cassiopea in the Florida Keys
**Corresponding Author:**	Kaden Muffett<br>University of California Merced<br>Galveston, Texas UNITED STATES OF AMERICA
**Keywords:**	Cnidaria; microbiome; Symbiosis; Symbiodiniaceae; Scyphozoa
**Abstract:**	Interactions with microbial communities fundamentally shape metazoans' physiology, development, and health across marine ecosystems. This is especially true in zooxanthellate (symbiotic algae-containing) cnidarians. In photosymbiotic anthozoans (e.g. shallow water anemones and corals), the key members of the associated microbiota are increasingly well studied, however there is limited data on photosymbiotic scyphozoans (true jellyfish). Using 16S rRNA barcoding, we sampled the internal and external mucus of the zooxanthellate Upside-Down Jellyfish, Cassiopea xamachana throughout eight sites covering the full length of the Florida Keys. We find that across sites, these medusae to have low-diversity internal microbial communities distinct from the communities of their external surfaces and their environment. These internal communities are dominated by only three taxa: Endozoicomonas cf. atrinae, an uncultured novel Mycoplasma, and Vibrio cf. coralliilyticus. Cassiopea bell mucosal samples conform largely to the communities of surrounding sediment with the addition of Endozoicomonas cf. atrinae. The microbial taxa we identify associated with wild Florida Keys Cassiopea bear a strong resemblance to those found within photosymbiotic anthozoans, increasing the known links in ecological position between these groups.
**Order of Authors:**	Kaden Muffett
	Jessica Labonté
	Maria Pia Miglietta
	Corresponding
**Response to Reviewers:**	Response to editor and reviewers:<br><br>To all,<br>Thank you for taking the time to review this manuscript. Your suggestions and critiques have been very helpful. The majority of changes in this round of the manuscript cover refinement of captions, movement of extraneous figures to the supplementary materials, and greater detail in some sections of the methodology section. Hopefully the in-text modifications improve the clarity and readability of the text.<br>Sincerely,<br>KMM<br><br>Reviewer 1<br>To Reviewer 1,<br>Thank you for your specific critiques. As you have suggested, the ethanol preserved samples have been removed from the methods section in order to improve clarity. Table #1 has been separated to provide adequate space for definitions and the term “GVC” is now clarified earlier. Additional analyses of the physical parameters have now been added to the supplements, as is addressed in the specific response below. Additionally, the limited nature of this sampling has now been reiterated in the core microbiome section.<br>Thank you,

Powered by Editorial Manager® and ProduXion Manager® from Aries Systems Corporation

Kaden Muffett

Specific points
Briefly, since the samples preserved in alcohol did not give good results, these should be removed from the manuscript.
The authors accept this suggestion and have modified the text accordingly.
Table 1 needs work with headings, column spaces, and some definitions.
The GVC term should be introduced in Methods.
Thank you for bringing up this oversight. The correction has been made.
The authors collected information on physical parameters, but such information was not relevant in their analyses, correct?
Yes, as these were point-in-time measurements, it was deemed imprudent to include them in the primary analysis. Redundancy analysis plots (RDA) including these features of interest have now been added to the supplementary materials for those interested. They are presented in this table to inform others' interpretation of these data, as factors associated with these have been demonstrated important in other microbiomes.
Finally, in the Results section referring to Core Microbiome, the authors need to stress that the sampling was done in one month within a single year.
Thank you for the suggestion, this has now been specified in the results.

Reviewer #3
To reviewer #3,
Thank you for your review of this manuscript. Your suggestions have improved the precision and clarity of the text. In addition to your specific comments, you also indicated some more general inquiries regarding the nature of these microbiomes. While I am not capable of speaking to the function of the Cassiopea microbiome with these data, I do think that these questions are worth pursuing with other study designs. Please see the responses to all of your specific requested edits below.
Sincerely,
KMM

Specific comments
Title: because both internal and external microbiomes were investigated, the words "gastric microbiome" limits the scope of the study, which is broader. My suggestion is to alter it.
Thank you for your suggestion. The title has been modified to include both external and internal data.
Keywords: the word "Cassiopea" is already present in the title. Please, remove it from the keywords section and replace it with another one.
Corrected.
Lines 28-29: not all stony corals, octocorals and sea anemones are symbiotic. I recommend replacing "the well-known" with "some".
Corrected.
Line 43: please, replace "hot" with "warm". Also, write the full name of C. andromeda, including authority and year of publication.
Corrected.
Line 45: why is having a "sticky interface" significant? This phrase could be a little more informative.
Corrected. The text now specifies that this is a barrier between host and environment.

Line 47: please, add "SOME" stony corals and specify how much heat can Cassiopea tolerate as well as max and min temperature fluctuations.
Corrected, specific tolerance details added.
-Line 98: please, write GVC in full. The same should be applied to caption of figure 1 and 3.
Thank you, this issue has been corrected.
Line 117: my suggestion is to add "amplification" as well.
Corrected.
Line 122: did you mean amplification?
Corrected.
Line 137: great work at providing the codes and describing each command. Congrats!
Thank you for your praise.
Line 139: please, write ASV in full the first time.

Corrected.
Line 143: out of curiosity…Did you face any limitations in the completeness of the Silva database? Do you think that using additional databases would have provided a different outcome?
It is certainly possible. The Silva database has made substantial strides in the last several years when it comes to marine taxa, so issues I had with ID in the first tests (2021) are largely absent now. RDP certainly was considered, but the comparability of Silva across studies is currently unbeatable. I also considered using the expanded McCauley classifier, but decided that, should someone want perfect taxonomic comparability to another study, they will likely need to reclassify anyway. Such is the reality of amplicon sequencing, and also our constantly in-flux marine microbial genera.
Line 151: GVC should be written in full since it's the first time it appears in the text.
This has now been corrected earlier in the text (methodology section)
Line 152: please, rewrite it as “34.808 taxa” to keep numbers standardized.
Corrected.
Line 179: what about the other 21 specimens?
The other 21 included medusae under 3 cm diameter (9 indv), and samples that did not fit on the final sequencing run with the resources available. This has now been specified in the text.
Line 181: please, remove “GVC”.
Corrected.
Line 182: please, add authority and year of publication.
Corrected.
Line 183: in total, 55 specimens were sampled, but only 34 were analyzed and out of these 4 were not identified as C. xamachana. Resulting in 30 specimens. Is that correct? If so, what happened to the other 17 specimens. That is not clear. What is the actual number of analyzed specimens?
Thank you, the description of the dataset has been standardized across the paper. It is now clear that 55 were collected, of these 46 were swabbed, of these 34 samples were sequenced, and of these 30 were C. xamachana.
Line 196: please, remove “(top)” and “(bottom)”.
Corrected.
Line 205: please, remove “(GVC)”
Corrected.
Line 228: please write ASVs in full every time it appears for the first time in a caption.
Corrected.
Line 229: displayed?*
Corrected.
Caption of figures 3 and 6: please, rewrite it explaining what each abbreviation mean (HHP, CK, LW…)
Thank you for your feedback. The explanations are now available within table 1.
Line 243: please, invert to "location (b+c)". Line 245: please, invert to “GVC (b) and bell (c) alpha diversity”, Line 256: please, write “PCA” in full. The same to line 352.
Corrected.
Line 272: please, add a single space after “of” (e.g.: of > 0.1%).
Corrected.
Line 280: Cassiopea should be in italics.
Corrected.
Caption of figure 6: please, explain each abbreviation and write GVC in full the first time it appears in the caption. The same should be applied to all captions (tables and figures).
This has been corrected across captions.
Line 288: please, correct the overspacing.
Corrected.
Lines 318 - 322: why some organisms are more abundant than others depending on the area where they were sampled (e.g. bell, gvc)? Could this have anything to do with their functional role?
It is entirely possible that this is related to functional role, however the assessment of functional role from 16S data, especially from divergent taxa, is difficult and error prone. This question is outside the scope of this paper but certainly an interesting one.
Line 353: “were” = where?*
Corrected.
Line 360: Cotylorhiza tuberculata (Macri, 1778). Authority and year of publication should be added the first time a species name is mentioned. Please, revise all species

Powered by Editorial Manager® and ProduXion Manager® from Aries Systems Corporation

names cited throughout the manuscript.
Corrected.
- Line 436: please, see author guidelines for how studies should be cited. For example, here, a comma is used after “et al.”, but the same is not applied to the citation above.
The erroneous citations have been modified, thank you for bringing this to my attention.
Line 446: remove “a” before “either”.
Corrected.
Line 452: if the Cassiopea’s external microbiome does not differ from the sediment microbiome, does it mean that Cassiopea does not have exclusive external microbiome?
This is a reasonable interpretation of the data. This community is enriched in Endozoicommonas relative to the benthos, as discussed elsewhere, but aside from this, the community is very similar to that of surrounding substrates.

Reviewer #4
Reviewer 4,
Thank you for taking the time to review this manuscript. You have raised two main concerns about the structure of this paper, the first being that we are not fully able to parse the impact of location with the samples collected, and secondly, that this paper has lapses in explanations of methodological purpose.
To the first point, I agree that the sample sizes are not adequate across all sites to justify this being the driver of the paper. In reality, what we have the ability to analyze is only the ways in which gastrovascular cavities and bells show consistencies. In order to bring the introduction into line with this, the introduction has been modified, including both stressing that this is an exploratory paper and that the focus is to identify the core components of the GVC and bell communities.
To the second point, I have modified the paper such that the impetus for specific tests are clearer, and switched out the less informative DMM figure with a more informative one. Where there were too many figures, they have been moved to the supplementary materials, and where there was inadequate detail in the supplements (eg Shannon diversity), the supplementary materials have been expanded.
In addition to these, in alignment with your suggestion, Bray-Curtis is now used throughout the text.
Thank you for your feedback,
Kaden Muffett
Specific comments:
Since microbiome sequencing is a data-driven method, in which ASVs are sequence constructs derived from the original samples, a data-driven pipeline is necessary to ensure that scientific hypotheses are tested without bias.
Thank you for this criticism. In this case, this is an exploratory study of this community, and while I understand the concern for bias, I believe there to be value in the data collected when framed as variability within the Cassiopea microbiome. I accept your criticism as this: The aim of this paper specifically states “compare the microbial communities of Cassiopea across sites of the Florida Keys”. I agree that I do not have adequate replicates to fully elucidate differences between sites. As such, I have changed the wording of this aim to reflect the core of what this work has accomplished, “The aim of this work is to identify the core components of the internal and external mucosal microbiomes of Cassiopea within the Florida Keys.”.
This sentence also includes a typo: archaeal (not archael). A similar word choice appears to have been made in the title. Overall, the writing needs improvement.
The typos throughout the text have been corrected.
Another issue is that there are too many figures in the main text. Some of them, such as Figure 1, could be moved to the Supplementary Files. The same applies to Table 1.
Thank you for your suggestion. While I understand that some of these figures and tables may seem extraneous, this paper is serving a diverse group of scientists, and other reviewers have been and remain interested in the inclusion of this table.
Many of the remaining figures should also be improved in terms of quality and explanation, as they currently appear without adequate context.
Thank you for this criticism. The figure captions have been modified to better explain the contents of each figure.
Why were the main phyla and classes depicted?
The main phyla and classes are depicted in this paper because of precedent. For past coral (Dunphy et al 2019 (https://doi.org/10.1038/s41598-019-43268-6), Hernandez-Agreda 2018 (https://doi.org/10.1128/mbio.00812-18)) and scyphozoan microbiome

Powered by Editorial Manager® and ProduXion Manager® from Aries Systems Corporation

work (Ohdera et al 2024 (https://doi.org/10.1371/journal.pone.0298002) , Peng et al 2021 (https://doi.org/10.3389/fmicb.2021.647089)), this has been a common display mechanism. This figure is displayed for easy communication of the major groups found in these samples within this study.
Why use Euclidean distance, Faith's PD, or calculate the core microbiome?
Thank you for your questions. In this case, I did not fully consider the impact of Euclidean diversity. I have changed all beta diversity to Bray-Curtis throughout the text. For Faith's PD, I have now specified the following in the methods section "In order to identify the degree to which diversity spanned primarily shallow or deep phylogenetic distances, Faith's phylogenetic diversity was calculated for all samples and sample types. Faith's phylogenetic diversity was computed.." . For the core microbiome, an explanation of choice is now provided in the methods "In order to identify taxa consistently present across samples of a given type that may be rare, core microbiome was calculated for internal and external communities of Cassiopea. Core microbiome was computed.."
What is the Dirichlet multinomial mixture (DMM), and why was it used?
Dirichilet multinomial mixture analysis tests an ideal number of populations for a provided diverse population set. The purpose of this has now been clarified in the methods.
"In order to identify whether a gastrovascular/ bell split community partitioning was appropriate for all samples, and identify subcommunity level enterotypes within Cassiopea internal and external microbiomes, Dirichlet Multinomial mixture analysis, as described by Holmes and implemented in the DirichletMultinomial package v 1.48 was used for Cassiopea – derived samples (52,53). "
The rationale behind assessing the substrate and water samples is unclear. Grouping eight samples (one per site) and comparing them with thirty GVC and bell samples in Figure 2 seems counterintuitive. Please explain this logic more clearly to the reader. A simple Venn diagram of shared ASVs would more effectively depict the percentage of ASVs shared among bell, water, substrate, and GVC samples. I could not extract this information from Table 2.
The inclusion of the substrate and water in figure 2 and table 2 serve as comparison tools to the microbiomes discussed. An upset plot of group to group overlap is now included.
Still regarding Figure 2, an "unknown" category should be added to group ASVs classified only as "Bacteria." This would create a bar that fills each sample (or group of samples) to 100%, aligning the bars in the plot. It is also helpful to limit the figure to the x most abundant taxa to avoid cluttering the legend with indistinguishable shades of similar colors.
Thank you for your suggestion. The colors and design for this have been altered as suggested to aid comprehensibility.
I don't think the in-text Shannon values (L234–L240), or any diversity metric, are informative as currently presented. These should be included in a Supplementary Table with appropriate measures of dispersion and statistical tests, indicating statistically distinct groups with letters.
Thank you for the suggestion. A table of Shannon diversity metrics is now available in the supplementary materials.
Why was only the GVC core microbiome analyzed and then searched in other samples (Figure 6)? What do the abbreviations GB, CK, VP, BKP, etc., refer to in this figure? These should be defined in the legend. Also, refer to the sampling table that should include these abbreviations. A map or schematic of these sites would be valuable for readers unfamiliar with the study area. I encourage the authors to consider improved ways to present this result. Figures 7 and 8 could be merged and moved to the Supplementary Material.
The specificity of this gastrovascular cavity is of interest to the field. The explicit separation of internal and external samples is rarely done for cnidarian samples. As such, making it clear that the core of this community is not represented in other sample times is a key goal of this paper.

Regarding the DMM, no figure shows the model fit against the number of components (clusters), as stated in L309: "model probability value was optimal for two communities, one GVC and one bell." This result should be presented to justify grouping the samples by tissue, as previously critiqued.
Additional details on this are now provided in-text and supplementary figs have been added.

	I cannot read the text in Figure 10. Please explain the star plot in the Results section.<br>Thank you for your comment. The reasoning behind this figure has been expanded in the results section.<br>The “Host medusa species identity” section should not be the final result, as it is disconnected from the rest of the section. If the intent was to show that there is no microbiome distinction between CA and CX, this could be stated at the beginning of the Results and shown in the Supplementary Material.<br>Thank you for your suggestion. This section has been removed and moved to the supplements.<br>What is meant by “Retention metrics” (L144)? Does this refer to the number of reads retained after preprocessing? Please clarify this for the reader.<br>Thank you for your comment. This has now been clarified in the text.<br>The provided GitHub repository (https://github.com/kadenmuffett/Cassiopea-Code-Microbiome-Repository) is not publicly accessible. Either correct this or provide the code as a Supplementary File.<br>While I have not been able to replicate this issue, a copy of the code is now included in the supplementary material as well.<br>Why are WTR and substrate samples not described in the Sampling section alongside bell and digestive cavity samples (L103–L111)?<br>Water and substrate samples are described immediately prior to GVC and bell samples. 1L of water was filtered through a 0.2um filter, then preserved in DESS. Sediment samples were preserved in DESS directly.<br>Abbreviations used throughout the manuscript should be introduced in this section, before they appear in the Results. For example, GVC is mentioned for the first time in the Table 1 legend without prior definition.<br>This has been corrected.<br>Why is Bray-Curtis mentioned in the Methods (L161) but not in the Results? Remove it if it was not used. Please consider my suggestion above regarding this metric.<br>Thank you for your comment. Euclidean has been replaced with Bray-Curtis throughout the text.<br>Figures 1, 2, and 7 are of poor quality — the text is unclear. Were they uploaded as vector files? In Figure 2a, the text within the box is unreadable.<br>Please be assured that the image quality displayed in the reviewer pdf is poor quality and not representative of the quality of the uploads.<br>Also, regarding the Results, current phylum-level nomenclature should be used alongside classical names (L192–L193). For example, Pseudomonadota (formerly Proteobacteria).<br>Thank you. Bacterial phylum names have been modified to reflect this.<br>While I don’t prefer sections within the Discussion, the text is interesting and connects the findings to the scientific literature. Including more explicit references to the underlying Results would further improve it.<br>Thank you, some additional connection to the test has been added to improve congruity across sections.<br>Please cite SILVA database (10.1093/nar/gks1219) (L144)<br>This has been corrected.<br>Refraction curves? (L155). Rarefaction<br>This typo has been corrected.
**Additional Information:**	
**Question**	**Response**
**Financial Disclosure**<br><br>Enter a financial disclosure statement that describes the sources of funding for the work included in this submission. Review the submission guidelines for detailed requirements. View published research articles from *PLOS ONE* for specific examples.	Yes

This statement is required for submission and **will appear in the published article** if the submission is accepted. Please make sure it is accurate.<br><br>**Funded studies**<br>Enter a statement with the following details:<br>• Initials of the authors who received each award<br>• Grant numbers awarded to each author<br>• The full name of each funder<br>• URL of each funder website<br>• Did the sponsors or funders play any role in the study design, data collection and analysis, decision to publish, or preparation of the manuscript?<br><br>Did you receive funding for this work?	
Please add funding details.<br>as follow-up to "**Financial Disclosure**<br><br>Enter a financial disclosure statement that describes the sources of funding for the work included in this submission. Review the submission guidelines for detailed requirements. View published research articles from *PLOS ONE* for specific examples.<br><br>This statement is required for submission and **will appear in the published article** if the submission is accepted. Please make sure it is accurate.<br><br>**Funded studies**<br>Enter a statement with the following details:<br>• Initials of the authors who received each award<br>• Grant numbers awarded to each author<br>• The full name of each funder<br>• URL of each funder website<br>• Did the sponsors or funders play any role in the study design, data collection and analysis, decision to publish, or preparation of the manuscript?<br><br>Did you receive funding for this work?"	This work was funded by Texas Sea Grant, NSF EAGER award # 1936565, and Texas A&M University Galveston. The funders had no role in study design, data collection and analysis, decision to publish, or preparation of the manuscript.

Powered by Editorial Manager® and ProduXion Manager® from Aries Systems Corporation

Please select the country of your main research funder (please select carefully as in some cases this is used in fee calculation).<br>as follow-up to "**Financial Disclosure**<br><br>Enter a financial disclosure statement that describes the sources of funding for the work included in this submission. Review the submission guidelines for detailed requirements. View published research articles from *PLOS ONE* for specific examples.<br><br>This statement is required for submission and **will appear in the published article** if the submission is accepted. Please make sure it is accurate.<br><br>**Funded studies**<br>Enter a statement with the following details:<br>• Initials of the authors who received each award<br>• Grant numbers awarded to each author<br>• The full name of each funder<br>• URL of each funder website<br>• Did the sponsors or funders play any role in the study design, data collection and analysis, decision to publish, or preparation of the manuscript?<br><br>Did you receive funding for this work?"	UNITED STATES - US
**Competing Interests**<br><br>Use the instructions below to enter a competing interest statement for this submission. On behalf of all authors, disclose any competing interests that could be perceived to bias this work—acknowledging all financial support and any other relevant financial or non-financial competing interests.<br><br>This statement is required for submission and **will appear in the published article** if the submission is accepted. Please make sure it is accurate and that any funding	The authors have declared that no competing interests exist.

sources listed in your Funding Information later in the submission form are also declared in your Financial Disclosure statement.<br><br>View published research articles from *PLOS ONE* for specific examples.<br><br>**NO authors have competing interests**<br><br>Enter: *The authors have declared that no competing interests exist.*<br><br>**Authors with competing interests**<br><br>Enter competing interest details beginning with this statement:<br><br>*I have read the journal's policy and the authors of this manuscript have the following competing interests: [insert competing interests here]*<br><br>* typeset	
**Ethics Statement**<br><br>Enter an ethics statement for this submission. This statement is required if the study involved:<br><br>• Human participants<br>• Human specimens or tissue<br>• Vertebrate animals or cephalopods<br>• Vertebrate embryos or tissues<br>• Field research<br><br>Write "N/A" if the submission does not require an ethics statement.<br><br>General guidance is provided below. Consult the submission guidelines for detailed instructions. **Make sure that all information entered here is included in the Methods section of the manuscript.**	Special use permitting for these samples was waived by Florida Fish and Wildlife.

**Format for specific study types**<br><br>**Human Subject Research (involving human participants and/or tissue)**<br>• Give the name of the institutional review board or ethics committee that approved the study<br>• Include the approval number and/or a statement indicating approval of this research<br>• Indicate the form of consent obtained (written/oral) or the reason that consent was not obtained (e.g. the data were analyzed anonymously)<br><br>**Animal Research (involving vertebrate animals, embryos or tissues)**<br>• Provide the name of the Institutional Animal Care and Use Committee (IACUC) or other relevant ethics board that reviewed the study protocol, and indicate whether they approved this research or granted a formal waiver of ethical approval<br>• Include an approval number if one was obtained<br>• If the study involved *non-human primates*, add *additional details* about animal welfare and steps taken to ameliorate suffering<br>• If anesthesia, euthanasia, or any kind of animal sacrifice is part of the study, include briefly which substances and/or methods were applied<br><br>**Field Research**<br><br>Include the following details if this study involves the collection of plant, animal, or other materials from a natural setting:<br>• Field permit number<br>• Name of the institution or relevant body that granted permission	
**Data Availability**<br><br>Authors are required to make all data underlying the findings described fully available, without restriction, and from the time of publication. PLOS allows rare exceptions to address legal and ethical concerns. See the PLOS Data Policy and FAQ for detailed information.	Yes - all data are fully available without restriction

Powered by Editorial Manager® and ProduXion Manager® from Aries Systems Corporation

A Data Availability Statement describing where the data can be found is required at submission. Your answers to this question constitute the Data Availability Statement and **will be published in the article**, if accepted.<br><br>**Important:** Stating 'data available on request from the author' is not sufficient. If your data are only available upon request, select 'No' for the first question and explain your exceptional situation in the text box.<br><br>Do the authors confirm that all data underlying the findings described in their manuscript are fully available without restriction?	
**Describe where the data may be found in full sentences. If you are copying our sample text, replace any instances of XXX with the appropriate details.**<br><br>• If the data are **held or will be held in a public repository**, include URLs, accession numbers or DOIs. If this information will only be available after acceptance, indicate this by ticking the box below. For example: *All XXX files are available from the XXX database (accession number(s) XXX, XXX.*).<br>• If the data are all contained **within the manuscript and/or Supporting Information files**, enter the following: *All relevant data are within the manuscript and its Supporting Information files.*<br>• If neither of these applies but you are able to provide **details of access elsewhere**, with or without limitations, please do so. For example:<br><br>*Data cannot be shared publicly because of [XXX]. Data are available from the XXX Institutional Data Access / Ethics Committee (contact via XXX) for researchers who meet the criteria for access to confidential data.*<br><br>*The data underlying the results presented in the study are available from (include the name of the third party*	All raw sequencing files are available from the SRA database (BioProject accession numbers PRJNA1020446, PRJNA1020388.)

and contact information or URL).<br>• This text is appropriate if the data are owned by a third party and authors do not have permission to share the data.<br><br>* typeset	
Additional data availability information:	

Powered by Editorial Manager® and ProduXion Manager® from Aries Systems Corporation

Manuscript

Click here to access/download;Manuscript;Clean_Cassiopea_Microbiome_PL

Florida Keys *Cassiopea* host benthos-like external microbiomes and a gut dominated by *Vibrio*, *Endozoicommonas* and *Mycoplasma*

Kaden M Muffett[1]*, Jessica M Labonté[2], Maria Pia Miglietta[2]

1 University of California Merced, Merced, CA, 95343

2 Texas A&M University at Galveston, Galveston, TX, 77554

*Corresponding email: kmmuffett@gmail.com

Running Page Head: Bacteria of *Cassiopea* in the Florida Keys

Abstract

Interactions with microbial communities fundamentally shape metazoans' physiology, development, and health across marine ecosystems. This is especially true in zooxanthellate (symbiotic algae-containing) cnidarians. In photosymbiotic anthozoans (eg. shallow water anemones and corals), the key members of the associated microbiota are increasingly well studied, however there is limited data on photosymbiotic scyphozoans (true jellyfish). Using 16S rRNA barcoding, we sampled the internal and external mucus of the zooxanthellate Upside-Down Jellyfish, *Cassiopea xamachana* during August throughout eight sites covering the full length of the Florida Keys. We find that across sites, these medusae to have low-diversity internal microbial communities distinct from the communities of their external surfaces and their environment. These internal communities are dominated by only three taxa: *Endozoicomonas* cf. *atrinae*, an uncultured novel *Mycoplasma*, and *Vibrio* cf. *coralliilyticus*. *Cassiopea* bell mucosal samples conform largely to the communities of surrounding sediment with the addition of *Endozoicomonas* cf. *atrinae*. The microbial taxa we identify associated with wild Florida Keys

Cassiopea bear a strong resemblance to those found within photosymbiotic anthozoans, increasing the known links in ecological position between these groups.

Key words: Cnidaria, microbiome, symbiosis, *Symbiodineaceae*, Scyphozoa

Introduction

Cnidarian-photosymbiont interactions are central to ecosystem function in coral reefs and beyond. Algal photosymbionts, or zooxanthellae, are present in some well-known stony corals, octocorals, and anemones, but also play important roles in a variety of lesser-known jellyfishes, chief among them, jellyfish of the genus *Cassiopea* (1–4). For symbiotic anthozoans, microbial communities play a key role in maintaining health of this photosymbiont partnership, conferring disease resistance and heat tolerance on hosts (5–7). These microbial communities are often specialized, with distinct corals demonstrating co-evolution with members of their bacterial communities (8). Several prevalent genera, such as *Endozoicomonas*, are found across the microbiomes of many hosts (9). While anthozoan communities are under active investigation, few analyses have documented the stability and nature of microbiome communities associated with zooxanthellate jellyfishes, a group with divergent life history traits from Anthozoa but with a comparable obligate symbiosis with dinoflagellates (clade Symbiodiniaceae).

The Upside-Down Jellyfish, *Cassiopea* spp., is a model organism for cnidarian-photosymbiont interactions (10,11). These medusae are residents of tropical and subtropical waters globally and can survive in water that is frequently shallow (as little as 8 cm depth) and warm (up to 33˚C for *Cassiopea andromeda* (Forskal, 1775)) (12,13). *Cassiopea* obligatorily hosts algal symbionts, losing mass even when well fed while in aposymbiotic conditions (14). As in corals and other scyphozoans, mucus plays a central role for *Cassiopea*, presenting an interface and barrier between the host tissue and the marine environment (15,16). In contrast to other symbiotic cnidarians, such as photosymbiotic stony corals, *Cassiopea* can tolerate large temperature ranges (18C to 33C) and intense temperature fluctuations (+/- 6C) (17–19).

Understanding the *Cassiopea* natural microbial community could provide new potential insights into the function and diversity of cnidarian holobionts.

Past jellyfish (class Scyphozoa) microbial studies have focused on the microbiome of lab-reared specimens (20,21) or limited sample sizes (22–24). While these types of studies may be able to track state changes across life stages (20) and between condition types (symbiotic/aposymbiotic) (21), there is little guarantee that the microbial communities of jellyfish kept under prolonged laboratory conditions translate bear resemblance to *in situ* microbiomes. Recent *in situ* studies have provided insights into microbial taxa associated with some scyphozoans ("true jellyfish") but have not included species of the genus *Cassiopea*. In the moon jellyfish, *Aurelia* spp*.*, individuals from the Chinese coast were *Vibrionaceae*-dominated, while samples from the northwestern Atlantic were *Mycoplasma*-dominated(24,25). Between the Indonesian marine lakes Kakaban, Haji Buang, and Tanah Bamban, *Mastigias* sp. microbial community composition varied significantly by lake, but consistently included *Oceanospirillales* (primarily *Endozoicomonas*-like) (22). Across hosts, *Mycoplasma*-like bacteria are common components of the identified gut and tissue microbiome of jellyfishes, though their functions remains unknown (24–29). Vertebrate pathogen research is often a focus of scyphozoan microbiome discovery—*Tenacibaculum* has been identified in several scyphozoan species, e.g. *Cotyllorhiza tuberculata, Aurelia aurita,* and *Pelagia noctiluca*, and, as a fish pathogen, which can have important fisheries ramifications (24,25,27,30).

As most studied jellyfish populations occur in coastal waters only sporadically, the relative consistency of *Cassiopea* assemblages provides a system in which body compartment microbiomes can be studied without the confounding impacts of single source sites. *Cassiopea* spp. presents an opportunity to act as a comparison group for the microbial communities

associated with both other scyphozoans and symbiotic anthozoans. The ubiquity of *Cassiopea* assemblages within the Florida Keys allows for the examination of differences in the microbial community of one population (Florida Keys *Cassiopea*) at one time point across a broad geographic area.

The aim of this work is to undertake a first exploratory study of the wild microbiome of *Cassiopea*, and to identify the core components of the internal and external mucosal microbiomes of *Cassiopea* within the Florida Keys.

Materials and Methods

Sampling

A total of 55 *Cassiopea* medusae were collected by wading in eight sites along the length of the Florida Keys in late August 2021 over eight days (see Table 1 for a list of sites). Species identification for each individual is reported in Muffett & Miglietta 2023 and summarized in Supplementary Table S1 (31). At each site, coordinates, temperature, time of day, depth, salinity, and pH were recorded (Table 1). Salinity was measured with a refractometer, and temperature and pH were measured with an Apera A1209. A minimum of two and a maximum of 10 medusae were sampled per site. At each site and in between each medusa, all equipment was cleaned with absolute ethanol and 10% bleach solution. A 1 L water sample was collected from above (>10 cm above the benthos) each medusa aggregation and filtered (0.2 um pore size, Thermo Scientific Cat No.09-740-30G) with a manual sampler as described in the USGS manual water sampling protocol (32), replicate filters were placed in 15 mL of dimethyl sulfoxide ethylenediamine tetraacetic acid saturated salt storage solution (1 L pH 7.5: 93.06 g EDTA, 60

mL 20 % NaOH solution, 20 mL 25 % HCl, 40 mL DMSO, 800 mL water, NaCl to oversaturation; see ref. 30) (commonly referred to as DESS), one filter in each buffer. At each *Cassiopea* medusa aggregation, duplicate scrapings of ~10 g of sediment collections (substrate sample) were placed in 15 mL of DESS storage solution.

Table 1: List of collection sites. Table with Site Name (the collection location), site code (the shorthand used for these locations named for local landmarks, see supplementary table), latitude, longitude, salinity (parts per thousand), pH, surface temperature (in Celsius), average diameter of collected medusae from the site (centimeters), and date and time of collection. Number of paired gastrovascular cavity (GVC) and bell swab samples per collection are for *Cassiopea xamachana* (CX). For collections that included *C. andromeda* mitotyped medusae, the number of *C. andromeda* samples is presented after the dash (CA). These values only include samples collected in DESS. Due to a storm warning, collections from Bahia Honda occurred across two days (TP1, TP2).

**Site Name**	**Site code**	**Latitude**	**Longitude**	**Salinity (ppt)**	**pH**	**Temp (C)**	**Average depth (m)**	**Average col. medusa diam (cm)**	**# of GVC Samps**	**# of Bell Samps**	**Date (AM/PM)**
Key West	GB	24.561526	-81.7881727	36	8.2	30.4	0.56	6.62	CX 3/ CA 1*	CX 3/ CA 1*	15.8.2021 PM
Cudjoe Key	CK	24.6775378	-81.4991819	36	8.4	32.6	0.36	5.91	CX 4/ CA 2*	CX 4/ CA 2*	17.8.2021 AM
Big Pine Key	BPK	24.6978731	-81.3572574	35	7.7	29.1	0.15	4.43	CX 4	CX 4	15.8.2021 PM
Bahia Honda Key TP1	VP	24.6825793	-81.2293673	37	8.3	31.8	0.86	12.41	CX 2	CX 2	13.8.2021 PM
Bahia Honda Key TP2	VP	24.6825793	-81.2293673	36	8	29.4	0.51	10.28	CX 2	CX 2	15.8.2021 AM
Marathon Key	MK	24.69396	-81.0980507	31	8.1	35.2	0.08	6.53	CX 7	CX 7	11.8.2021 PM
Lower Matacumbe Key	LW	24.8582157	-80.7267927	35	8.1	32.9	0.89	11.40	CX 2	CX 2	16.8.2021 PM
Tavernier	HHP	25.0233783	-80.4940316	31	8.1	33.3	0.35	12.26	CX 3	CX 3	16.8.2021 AM
Key Largo	KL	25.0872104	-80.4415797	35	7.8	32.0	0.56	4.30	CX 3/ CA 1*	CX 3/ CA 1*	18.8.2021 AM

Each *Cassiopea* medusa was collected from shallow waters <1.5 m within a single assemblage at each site. Medusae were gently grasped by the oral arms, lifted from the water, swabbed along the apex of the bell, then placed bell-down onto a pre-prepared dissection plate (Figure 1). External bell swabs were done in replicate and placed in 3 mL DESS. Medusa oral arm base was bisected between oral arm groups, and replicate swabs were inserted and circled the digestive cavity (hereafter referred to as gastrovascular cavity or GVC). Medusae < 3 cm in diameter were not sampled for gastrovascular cavity and bell microbiome. A total of 46 medusae were sampled. One internal and one external swab from each medusa was placed in DESS. A section of bell tissue was then collected and placed in ethanol for mitochondrial species confirmation (see 28). The size of each medusa was measured, and individual medusae were photographed. As sampling was done in a nonsterile environment, blanks for buffer with swab and buffer with filter were also taken.

Figure 1: Sampling strategy for medusae collection onsite. Demonstration of the typical orientation of medusa (right upper) and swab locations (apical bell surface and internal to medusa). Gastrovascular cavity (GVC) access strategy is displayed on the left and relevant environmental samples (water filter, substrate sample) are displayed on the right.

DNA Extraction and Sequencing

Samples in DESS were maintained at room temperature for three to ten days and then frozen. DESS can be stored at room temperature for at least thirty days without significantly compromising community integrity (33). DNA from all samples (see Supplementary Table S1) was then extracted using Zymobiomics DNA Kits (PN# D4300T). All samples were pre-tested through the amplification of the 16S rRNA gene (variable region V4). Only samples that

produced visible bands for the V4 region were sent for 16S rRNA V3-V4 preparation and sequencing, with a maximum of 7 specimens per site. In total, the DNA from 34 paired medusa internal and external microbiomes were sent for sequencing. Sequencing was performed by the University of Texas Medical Branch Sequencing Lab. Library was prepared with Zymo Quick 16S V3-V4 kit (PN #D6400) in 20 uL reactions (10 uL Quick-16S qPCR premix, 4 uL Quick-16S V3-V4 primers (341f: (CCTACGGGDGGCWGCAG, CCTAYGGGGYGCWGCAG) and 806r: (GACTACNVGGGTMTCTAATCC)), 4 uL water and 2 uL sample). qPCR was run using Roche LightCycler 480 Instrument II (10min 95C, [30sec 95C, 30sec 55C, 3min 72C]x20). PCRs were enzymatically cleaned according to Zymo Quick-16S kit protocols, and barcodes were added in a secondary reaction at 20 uL (10 min 95˚C, [30 sec 95˚C, 30 sec 55˚C, 3 min 72˚C]x5). Plates included two replicate microbial community standards (Zymobiomics Cat. No. D6300) and two PCR negative controls. Samples were loaded at 10 pM, with 15% 10 pM PhiX sequencing control, and run on Illumina MiSeq with a 600 cycle v3 kit.

Data preprocessing

Data generated was run through the DADA2 v 1.26 pipeline on R v 4.4 with only limited deviations from the standard protocols for amplicon sequence variant (ASV) generation(34,35). Briefly, sequences were filtered and trimmed ("filterAndTrim(truncLen=c(285,230), maxN=0, maxEE=c(4,4), truncQ=2,trimLeft = c(17,21)"), errors were error corrected ("learnErrors", "dada"), and sequences were merged ("mergePairs"). Reads between 380 and 458 bp were retained. Chimeric reads were removed ("removeBimeraDenovo"), then taxonomy was assigned using the Silva v 138.1 database ("assignTaxonomy") (36). Metrics on number of sequences passing quality control along with the full code can be found on the github (https://github.com/kadenmuffett/Cassiopea-Code-Microbiome-Repository). Sequence and

metadata tables were imported into phyloseq v 1.42, where reads assigned to mitochondria and chloroplast were removed. Contaminants were removed using the decontam package v. 1.18, identifying and removing 47 amplicon sequence variants based on differential abundance in swab and buffer controls (37,38).

Based on host genotyping, samples not belonging to *Cassiopea xamachana* (Bigelow, 1892) were removed. The dataset includes 60 paired bell and GVC samples (30 of each), 8 water samples and 8 substrate samples with 6.47 mil sequences across 34,808 taxa. The complete dataset (NCBI Bioproject Accession number PRJNA1020388) contains 12.78 million reads.

Statistical analyses

Rarefaction curves were generated in vegan 2.6.6.1 ("rarecurve") (Sfig. 1)(39). Shannon diversity was computed with vegan and observed diversity was ploted with phyloseq ("estimate_richness", "plot_richness")(37). In order to identify the degree to which diversity spanned primarily shallow or deep phylogenetic distances, Faith's phylogenetic diversity was calculated for all samples and sample types. Faith's phylogenetic diversity was computed using the MiscMetabar v 0.10.1, phanghorn v 2.12.1, metagMisc v 0.5.0 and PhyloseqMeasures v 2.1 packages by first generating an ML tree from all sequences over 300 reads across the dataset, then computing per sample Faith's diversity (40–45). Faith's diversity was displayed with ggstatsplot v 0.13.0 (46). Bray-Curtis distance were computed in phyloseq with "distance" to "ordinate" to "plot_ordination". Between group comparisons were completed with the pairwiseAdonis package v 0.4.1 (" pairwise.adonis2") on Bray-Curtis distance measurements (47). Statistical comparisons were computed with Kruskal-Wallis using the Kruskal.test and

pairwise comparisons using the "kruskal.test" and "dunn.test" commands with holm p adjustment (48).

Data transmutation for starplots and display was assisted by the speedyseq package v 0.5.3.9021 (49). Star plots were made using the stars package v 0.6-7 (50). Differential abundance was calculated with ALDEx2 v 1.38.0 following established pipelines for microbiome data (51,52).

In order to identify whether a gastrovascular/ bell split community partitioning was appropriate for all samples, and identify subcommunity level enterotypes within *Cassiopea* internal and external microbiomes, Dirichlet Multinomial mixture analysis, as described by Holmes and implemented in the DirichletMultinomial package v 1.48 was used for *Cassiopea* – derived samples(53,54). In short, likelihood models were built based on transformed (log10) abundances for ASVs, then an optimal number of distinct community types was selected based on minimum Laplace values ("mcapply(dmn)"). New models were then trained under known variable constraints (GVC or bell sample identity), and crossvalidated ("cvdmngroup()").

In order to identify taxa consistently present across samples of the bell and gastrovascular cavity, core microbiome was calculated for internal and external communities of *Cassiopea*. Core microbiome was computed with the microbiome package v 1.28.0(55). For environmental variable interpretation, a redundancy analysis was conducted on center log ration transformed microbial abundances using the microViz package (56).

Ethics Statement

This work was performed on lower level invertebrates not subject to Animal Use Protocols or within the purview of Texas A&M University IRB approval. Permitting for these specimens was waived by Florida Fish and Wildlife Conservation Commision.

Results

Data description

We analyzed paired microbiome samples associated with 30 medusae from eight sites within the Florida Keys. For each medusa, we analyzed the microbial composition of their gastrovascular cavity ("GVC") and external or bell mucus (herein referred to as "bell"), as well as their substrate ("Sub") or water environment ("WTR"). The four collected paired *C. andromeda* sample sets were excluded from the primary dataset, however they showed no divergence from the trends of the *C. xamachana* simultaneously collected (Supplementary table S1, Sfig2).

After filtering, 10.13 and 9.07 million reads were retained ("filterAndTrim") and merging ("mergePairs") respectively (for all sequence quality statistics, see supplementary table S2) . There were 8.91 million sequences after chimera removal. After primary phyloseq filtration, the dataset included 6.47 million sequences retained in 34,808 unique ASVs across 76 total samples (30 bell, 30 GVC, 8 water, 8 substrate). Reference sequences for top ASVs are also available in the supplementary material (table S3).

Distribution of sequences in full dataset

The dataset generated was predominantly bacterial with few archaeal reads. At the phylum level, 57% of sequences were Pseudomonadota (formerly Proteobacteria), 7% were Bacillota (Firmicutes), 13% were Bacteroidota, and 4% were Cyanobacteriota (Fig 2). No other phylum had greater than 3% relative abundance in the dataset.

Figure 2. Top ten phyla and class relative abundances of sequences in samples aggregated by sample type. Gastrovascular cavity and bell samples are n=30 samples. Water and sediment samples are n=8.

At the class level, Gammaproteobacteria comprised 41% of all sequences. Alphaproteobacteria, the next most abundant, comprised 17%. Bacteriodia (12%), Bacilli (6%), Cyanobacteriia (4%), and Desulfobacteria (3%), were the other classes with a relative abundance above 2%. No Archaean group was prevalent above a relative abundance of 0.25%, with the three most abundant classes being Halobacterota (0.25%), Thermoplasmatota (0.21%) and Nanoarchaeota (0.22%).

Distribution of sequences within gastrovascular cavity samples

Within gastrovascular cavity samples, 75% of all sequences belonged to just three orders, Pseudomonadales (39%), Mycoplasmatales (24%), and Enterobacterales (13%). The majority of Pseudomonadales sequences identified within the GVC were dispersed across very few OTUs, with 66% identified as *Endozoicomonas atrinae* ASV1 and 8% as *E. atrinae* ASV4. This was true of the Mycoplasmatales as well, with 77% of associated gut sequences identified as *Mycoplasma* sp. ASV2 or *Mycoplasma* sp. ASV9 (15%).

Distribution of sequences within bell mucus samples

At the phylum level, within the bell mucosal swabs, 55% of the sequences were Pseudomonadota, 14.5% Bacteroidota, 5.1% Cyanobacteriodota, and 6% Thermodesulfobacteriota (Desulfobacterota).

At the order level, the most abundant groups were Pseudomonadales (23%), Chromatiales (6%), Rhodobacterales (7%), Desulfobacterales (5%), and Flavobacteriales (5%). *Endozoicomonas atrinae* ASV1 was the single most abundant surface mucus ASV at a mean of 12%. The next most abundant were Synechococcus CC9902 ASV15 (1.3%), *Endozoicomonas atrinae* ASV4 (1.1%) and Chromatiales *Candidatus Thiobios* ASV6 (0.8%).

Substrate and water samples

Water samples shared some ASVs with the bell mucus of medusae, the largest contributing ASV was Chromatiales *Candidatus Thiobios* sp. ASV6 (8%). Additional prominent taxa were Rhodobacteraceae *HIMB11* ASV12 (6%), *HIMB11* ASV19 (5%), and SAR11 Clade III ASV 10 (4%). By contrast, substrate samples had no ASVs surpassing a mean of 1% abundance across sites, with the highest two ASVs Sandaracinaceae ASV94 and *Actibacter* ASV105 reaching 0.6% mean abundance across sediment samples (see table 2; extended table is table S4). Overlap was substantial between bell and sediment samples was higher than between any other group sets (Upset plot of overlaps in sfig. 3)

Table 2: Top five amplicon sequence variants for each sample type. Table with ASVs ordered from highest to lowest average proportion in samples of each type displayed as a percentage of composition within that sample type. Includes Silva 138 taxanomic assignment for each ASV.

	1	2	3	4	5
	ASV1 *Endozoicomonas atrinae*	ASV15 *Synechococcus* CC9902	ASV4 *Endozoicomonas atrinae*	ASV6 *Candidatus Thiobios*	ASV10 SAR11 *Clade III*
Bell	11.585 %	1.272 %	1.105 %	0.832 %	0.709 %

	ASV94 Sandaracinaceae	ASV105 *Actibacter*	ASV95 Desulfocapsaceae	ASV169 *Robiginitalea*	ASV358 *Clostridiisalibacter*
Sub	0.625 %	0.605%	0.566%	0.492%	0.474%
	ASV6 *Candidatus Thiobios*	ASV12 Rhodobacteraceae *HIMB11*	ASV19 Rhodobacteraceae *HIMB11*	ASV10 SAR11 *Clade III*	ASV18 *Marinobacterium*
WTR	7.521 %	5.839%	4.725%	4.497%	2.711%
	ASV1 *Endozoicomonas atrinae*	ASV2 *Mycoplasma*	ASV9 *Mycoplasma*	ASV4 *Endozoicomonas atrinae*	ASV11 *Vibrio coralliilyticus*
GVC	34.133%	20.099%	2.823%	2.327%	2.094%

Diversity

Shannon diversity varied by sample type (Kruskal-Wallis: chi-squared = 48.8, df = 3, p-value =1.412e-10). Shannon diversity indices for the gastrovascular samples varied from substrate samples and bell samples (Kruskal-Wallis Dunn test with Holm correction: GVC-Bell, p-val=0.00; GVC-Sub, p-val= 0.00; GVC-Water, p-val=0.07; Bell-Sub, p-val=0.07; Bell-Water, p-val=0.06; Water-Sub, p-val=0.01). Shannon diversity was not explained by location (Kruskal-Wallis: chi-squared = 9.7, df = 7, p-value =0.21)(Fig. 3, full diversity metrics tables in supplement s5 and s6).

Figure 3. Shannon diversity by sample type (a) and location (b+c). Gastrovascular cavity, bell, substrate and water Shannon diversity are shown across both the entire dataset and when segmented by sample type across sites with GVC (b) and bell (c) alpha diversity across sites. All p values of holm-adj Dunn comparison of means tests below a = 0.05 are displayed, with statistical test details displayed above each plot. Means and interquartile ranges are marked with box plots for each sample type.

Faith's phylogenetic diversity of communities were also distinct from each other (Kruskal-Wallis test, chi-squared = 44.1, df = 3, p-value = 1.4e-09). GVC communities were

distinct from bell and substrate comparison groups, while bells were distinct from water and gastrovascular cavities (Dunn test with holm correction) (Fig. 5, s7).

Figure 4. Violin plot of Faith's phylogenetic diversity. Kruskal-Wallis chi-sq (44.12), pvalue (1.4e-9) and pairwise dunn test with holm correction p-values are displayed. Differences between GVC-water and substrate-bell are not significant. Samples are colored by sample type. Means and interquartile ranges are marked with box plots for each sample type.

Beta diversity

Bray-Curtis distance showed differences between medusa and non-medusa samples (pairwise.adonis, GVC vs bell: p=0.001, bell vs sub: p=0.008, bell vs water: p=0.001, GVC vs sub: p=0.001, GVC vs water: p=0.001, sub v water p=0.001). Variability along the primary axis was found in both GVC and bell samples, while variability along the secondary axis was found mainly in GVC samples. Only one pair of gastrovascular cavity sampling sites of Marathon Key (MK) and Key Largo (KL) were significantly different (pairwise Adonis, df = 1, f = 4.63, p= 0.007). The external microbiomes of Marathon Key individuals were significantly different than those of Key West (GB) (pairwise Adonis, df = 1, f =2.28, p= 0.009), Tavernier (HHP) (pairwise Adonis, df = 1, f = 2.29, p= 0.007), and Bahia Honda Key (pairwise Adonis, df = 1, f = 5.14, p= 0.003) (full adonis comparisons in S8).

Figure 5. Principal component analysis of Bray-Curtis distance of samples. PCA is colored by sample collection latitude from lowest (orange) to highest (purple). PCA is presented both together and faceted by sample type. Sample type is designated by shape, circle (Bell), triangle (GVC), square (Substrate) and cross (Water). Bottom plot is presented to demonstrait relative consistency of sunstrate (Sub) and water (WTR) samples on primary axes 1 and 2.

Core microbiome

Core microbiome for the purposes of this study was evaluated based on 70% occurrence of an ASV and a relative abundance of > 0.1% of reads in all 34 gastrovascular cavity samples or external bell samples. This is in line with thresholds for other core microbiome work, however it is important to reiterate that this work was conducted only during one trip, and may be representative of a seasonal community assemblage or one restricted to this specific collection year (57). The most abundant ASV, *Endozoicomonas* ASV1, was present in 90% of GVC samples at a relative abundance of > 0.1% of reads, ASV2 and ASV4 were present in 80% (Fig. 6). Using less stringent criteria (>70% of samples, any abundance), ASV 11, ASV 132 and ASV 6 become part of the core microbiome. These ASVs are across only three genera- *Endozoicommonas*, *Mycoplasma* and *Vibrio* (full taxonomic details of ASVs can be found in table S9).

Figure 6. Abundance of *Cassiopea* core GVC microbiome across dataset. The top five amplicon sequence variants from the gut core microbiome across all sample types (Bell, GVC, Substrate (Sub) and Water (WTR)) organized from furthest south to furthest north sites. Note the difference in y axis maxima between bell and GVC samples (top) and substrate and water samples (bottom). See Table 1 for site details, the sites are Key West (GB), Cudjoe Key (CK), Big Pine Key (BPK), Bahia Honda Key (VP), Marathon (MK), Lower Matacumbe (LW), Tavernier (HHP), and Key Largo (KL).

In bell samples, ASV1, ASV104, and ASV86 have 73%, 70% and 70% relative abundance in samples at a 0.1% threshold. ASV6, ASV95, ASV37, ASV105, and ASV135 are present at any level in >70% of bell samples. These ASVs cover the genera *Endozoicommonas*,

Candidatus Thiobios, *Methyloceanibacter*, *Ruegeria* and unknown genera from Desulfocapsaceae and Actibacter (Fig. S4).

In terms of abundance, internal core microbiome components are not just present, but highly dominant in *Cassiopea*'s gut. Some of these taxa are highly restricted to GVC samples (*Mycoplasma*), while others are prevalent across body surfaces (*Endozoicomonas*) (Fig. S4).

The vast majority of ASVs were not differentially abundant between bell and GVC samples. Differential abundance (Aldex2, min effect size absolute value >[±2]) between the gastric and bell surfaces is restricted to the novel *Mycoplasma* group (ASV2), which is completely absent in external samples (Fig 8). No features were differentially abundant between substrate and bell samples (Sfig 6).

Dirichlet multinomial mixture (DMM) analysis identified specific proportions of many of these core microbiome as valuable for category differentiation. Within animal-associated samples, naïve model probability value was optimal for two communities, one GVC and one bell without subcommunities (k = 2, Laplace = 95583)(Fig. 7a). After training on separated gastrovascular and bell samples, overall model likelihood was similar and a two community structure was retained (Bell model: k=1, Laplace = 63910; GVC model: k=1, Laplace = 33200). ASV1 amount was a core group differentiator, with fewer meaningful ASVs defining the GVC community than the bell community (Fig. 7b, 7c). After crossvalidation, some bell and gastric samples remained grouped (Fig 7d.).

Figure 7. Dirichlet multinomial modeling model fit (a), community assignments (b), and impactful amplicon sequence variants differentiating the two community types (c+d). Dirichilet model choic graph (a) displays number of Dirichlet components (distinct assemblage types) tested vs model fit (Laplace value) in *Cassiopea* derived samples for a tested k value of 1-7. Best fit is at 2 components, or k = 2. Assignment of samples to one of two communities (b) is shown separated by location and sample type with a "0" value indicative of the bell community

type and a “1” value indicative of the GVC community type. Boxplots display means for each sampling group. Points are displayed with a small jitter (0.1) for visibility. ASV list (b) displays the top 0.5% most impactful drivers of body cavity prediction in GVC (c) and bell (d) Dirichlet multinomial models ordered from largest to smallest effect size.

Mean proportions of *Endozoicommonas*, *Mycoplasma* and *Vibrio* varied across locations. Variation of bell samples primarily occurred with proportion of *Endozoicommonas.* The communities across sites for gastrovascular samples varied in degree of community balance, resolving into primarily Mycoplasmatales-dominated and Pseudomonadales-dominated arrangements, with limited relationships between primary ecological vectors (pH, salinity, temp) and Mycoplasmatales, Vibrionales or Pseudomonadales (Fig. 8, Sfig. 7-9).

Figure 8. Star plots of community composition across sites. Star plots for internal (GVC), external (Bell) mucus swabs, water (WTR) and substrate (Sub) sample averages at each site separated by order (three most prominent orders and "other"). Each spoke of the star plot ranges from 0% (center) to 100%. **Internal** group, starting from the 3’ position and going counterclockwise: Pseudomonadales, Mycoplasmatales, Enterobacteriales and “Other”. **External** group spokes (going counterclockwise): Pseudomonadales, Rhodobacterales, Chromatiales, and “Other”. **Water** group spokes (going counterclockwise): Pseudomonadales, Rhodobacterales, Flavobacteriales, and “Other”. **Substrate** group spokes (going counterclockwise): Rhodobacterales, Flavobacteriales, Desulfobacterales, and “Other”. Each site's measured environmental conditions (salinity, water surface temperature, and pH) are presented below star plots, colored by intensity (low to high represented by pale to dark) . Sites are mapped onto their location within the Florida Keys.

Vibrio

ASVs identical to *Vibrio coralliilyticus* were found across *Cassiopea* gastric samples, with abundance of up to 10% of reads within the sample. This far surpasses rates in the

surrounding water and substrate samples (ASV11 averages: Bell: 0.05%, WTR: 0.03%, Sub: 0%, GVC: 2.09%) (Fig. 9).

Figure 9. Percent of reads (0%-10%) assigned to *V. coralliilyticus* amplicon sequence variants in bell, GVC, substrate and water samples averaged by site. Organized by body cavity**.**

Discussion

Cassiopea medusae show consistent differences in the microbial communities present on their bell and gastric surfaces. Gastrovascular cavity samples have lower diversity than the sediment, external surfaces, and host a novel *Mycoplasma* lineage. This low diversity is consistent with results for multiple other scyphozoans— including *Cotylorhiza tuberculata* (Macri, 1778) (27). *Cassiopea* also appears to have some variation in community composition between sites, a feature consistent with results from other species, such as *Aurelia*, *Mastigias*, and *Tipedalia* (22,26).

A bifurcated outer and inner microbiome

Across all sites, the gastrovascular cavities of *Cassiopea* medusae showed a core microbiome comprised of taxa previously found in photosymbiotic cnidarians. The external microbial community of *Cassiopea* from the Florida Keys adheres fairly closely to the epibenthic microbial community at each site, with the addition of *Endozoicomonas*. ASV1 *Endozoicomonas* cf. *atrinae* may be a primary mucosal component and extend to the exterior of the body through mucus exchange, as it is less prevalent on the bell surface than internal for most individuals. Alternitavely, ASV1 may be a common mucosal resident with no function for the host.

The abundant *Mycoplasma* and *Vibrio* cf. *coralliilyticus* from the gastric cavity of *Cassiopea* were not found in any external mucus samples and found at far lower abundances in external mucosal samples respectively.

The high diversity of the *Cassiopea* bell microbiome found in this study may be unique to *Cassiopea*. Previous work has reported in the low-diversity mucosa, oral arms and umbrella of a previously studied pelagic rhizostome, *Rhizostoma pulmo*, a likely result of the constant contact of *Cassiopea* with the epibenthos (28). The limited overlap between diverse bell communities and low diversity gut communities in *Cassiopea* is notable and consistent with the significant differences in body compartment microbiome of other scyphozoans and anthozoans (23,26,58). While the low diversity in scyphozoan microbiomes may seem distinct from many described stony coral microbial communities, these stony coral microbial profiles are often produced using whole body sampling. Lower diversity gastrovascular cavities with distinct community composition may also be a feature of stony corals (59–61).

Few microbial communities across sites within this study were significantly different from each other, as gastrovascular cavity components remained dominated by three taxa throughout the Keys. This microbiome bears limited resemblance to those sampled in common garden experiments and no resemblance to lab-held lines, demonstrating that “core” here should not be misconstrued as obligate (21,62). The lack of obligate bacterial taxa in the *Cassiopea* gastrovascular cavity is also consistent with other microbial hosts, notably the anemone *Exaiptasia diaphana*, another successful model cnidarian (1). While *Cassiopea* require their algal symbiont, bacterial partners may be transient, and bacteria may have far less significance to host health for *Cassiopea* than for specialized obligate hosts (e.g., leafhoppers) (63–66). Without a better understanding of specific metabolic function of different bacterial associates,

microbiome importance cannot be determined. We identified *Endozoicomonas*, *Mycoplasma*, and *Vibrio* as the three most common bacterial genera found within the gastrovascular cavity of *Cassiopea*. These are all commonly associated with coral microbial communities (8,9,67). Below, we provide information on these common bacterial components of cnidarian holobionts.

Endozoicomonas functions in microbiomes

The large proportion of *Endozoicomonas* in internal and external samples fits within a broader framework of both *Endozoicomonas* and known cnidarian-associated microbial communities; *Endozoicomonas* strains are present in a variety of invertebrates, including many corals, sponges, sea anemones, scyphozoans and cubozoans (9,67–70). Some hosts house multiple distinct *Endozoicomonas* genotypes, suggesting that different *Endozoicomonas* clades may provide different metabolic services to a host (e.g., Vitamin B12 metabolism) (71). *Endozoicomonas* is more commonly identified in healthy photosymbiotic scleractinian corals than bleached and diseased ones (72). For these corals, it may play a role in a variety of fundamental *Symbiodinium*–host interactions, including nutrient transport and symbiont cell breakdown (71). Notably, *Endozoicomonas* is not a consistent marker of microbiome health, its disappearance and reappearance without apparent host health declines has been identified in octocorals (2,70). In some coral, members of *Endozoicomonas* are found in close proximity to *Symbiodinium*, it is unknown whether this feature is shared with *Cassiopea* (71). As few cnidarians previously studied have gastric cavities large enough to be readily sampled, the finding of *Endozoicomonas* across cavities suggests that this group is not specialized in surface defense.

Within scyphozoa, *Endozoicomonas* is abundant in the microbial community of the rhizostome scyphozoan *Mastigias* (22). While *Mastigias*, like *Cassiopea*, host zooxanthellae from the family *Symbiodiniaceae*, not all symbiotic rhizostomes harbor the microbe, and not all taxa with the microbe are symbiotic (22,27). *Endozoicomonas* may be common across medusae, but the difficulty of culturing some strains may have excluded them from the results of past culture-based analyses (70,71). Greater clarity is needed on the function of *Endozoicomonas* for these medusae and other taxa.

Mycoplasmataceae: a poorly understood cnidarian microbiome resident

Mycoplasma, like *Endozoicomonas*, is present in studied coral and scyphozoan microbiomes (9,24,25,27). While intracellular parasitism is common for members of Mycoplasmataceae, those associated with cnidarians may be located extracellularly and exist as opportunistic commensals (2,73). *Mycoplasma* sp. is noted as a potential endosymbiont in lab-raised *Aurelia* polyps, while adults from many jellyfish populations are known to have them (24,26,29). The high divergence between this *Mycoplasma* and others of the genus (closest sequence identity on NCBI at 89%: Accn. No. NR_115937) suggests that this may be a hitherto unknown group. Whether this *Mycoplasma* represents an endosymbiont, a result of prey capture, or a parasite, its exclusivity to the gastrovascular tract in *Cassiopea* sampled within the Keys is notable, as other studies have not demonstrated gastrovascular cavity localization (26,29).

Vibrionaceae

Vibrionaceae is both a core part of coral microbiomes (9,74) and known to facilitate metamorphosis within *Cassiopea* (75,76), however, it is a highly diverse family. While *Vibrio* spp. are found in gut communities of presumed healthy scyphozoans (24,25) they can also be associated with senescence and disease(77,78) . For *Cassiopea* specifically, an increase in *Vibrio* has previously been associated with an aposymbiotic or stressed state (62). Given the variation in Vibrionaceae species and strains, family-wide statements on the beneficial or harmful nature of Vibrionaceae within *Cassiopea* gastrovascular cavities should not be made.

Vibrio coralliilyticus

In this dataset we found ample signal of a 16S V3-V4 with 100% sequence match to multiple *Vibrio* strains, including *V. coralliilyticus* and the oyster pathogen, *V. tubiashii*. As *Vibrio* species are highly varied, the sequence match does not mean assured pathogenicity towards any taxa (79–81). The apparent health of the medusae from whom these samples were collected suggest either a lack of impact of this specific group on the host or a host-microbe facultative interaction between this *Vibrio* and *Cassiopea.* While there is data on *Cassiopea* microbiome antimicrobial isolates and *Cassiopea* mortality when faced with *Serratia marcescens* infection, we lack adequate details on the *Vibrio* – *Cassiopea* system to determine whether this represents a lowlying widespread infection or a mutualism (82,83).

Drivers of external microbiome

While the external microbiome was not distinct from the sediment around it, it is worth discussing the major groups more common externally than internally. While the core bell microbiome represents a substrate-like community, the bell core microbiome includes representatives from families and genera with a variety of potential metabolic capacities. The

most plentiful of these ASVs was *Candidatus Thiobios,* known as a sulfur-oxidizing epibiont of the *Zoothamnium niveum* (84,85). As this bacteria can be found in the Florida Keys, their appearance on *Cassiopea* may be circumstantial, but is consistent across medusae (84). Isolates from *Methyloceanibacter* have been demonstrated to be methane oxidizers from marine environments (86). These may be sediment associated or may recruit to the host to utilize algal products. The potential sulfate reducers within Desulfocapaceae may similarly recruit to the bell and its access to sediment pore waters (87). While *Pseudomonas* cf. *stuzeri* was not as high abundance as the other metabolizers listed above, it may have a role in denitrification or coral health for some species (88–90)

In addition to microbes with known metabolic functions that align with the *Cassiopea* holobiont, the genus *Ruegeria* includes many marine species and has been identified in the microbiomes of photsymbiotic and nonphotosymbiotic cnidarians alike (9,77,91). As Dirichlet modeling for bell microbiomes gave similar weight to many bacteria, the relative impact of all of these bacterial epibionts on the host is unclear.

Laboratory cultures

Cassiopea are primarily used for in-lab studies on symbiosis (12,14,92). Some of these use wild-caught individuals, and others use lab-bred cultures, many of which have been held for years. The lab-raised *Cassiopea* used in Rothig et al. 2021 appear to have a completely different microbiome than the wild individuals caught here (21). This potential discrepancy should be considered when using wild-caught or captive-bred individuals, as captive-bred symbiosis and tolerance levels may differ from medusae in the wild. These discrepancies in microbiome are found in other model cnidarians, such as *Exaiptasia* (1). The information on wild *Cassiopea*

microbial communities from the Florida Keys presented here may allow researchers working with lab-raised individuals to better contextualize the taxa that they find in cultured medusae.

Conclusion

The microbiome of *Cassiopea* within the Florida Keys demonstrates strong body compartment discrimination and includes a central group of ASVs overrepresented in the gut and bell mucus. All of these families, Pseudomonadales, Vibrionaceae, and Mycoplasmataceae, are found in other medusae and anthozoans. Despite this overrepresentation of some microbial taxa within the gut, the *Cassiopea* bell is highly diverse, and overlaps with the communities of the water and substrate in and on which it resides. The consistency of *Cassiopea* microbial communities across the Florida Keys demonstrates that specific taxa are strongly associated with *Cassiopea* and that this can extend across many sites with diverse physical conditions. This work provides a greater understanding of the wild microbiome of the common lab organism *Cassiopea*.

Acknowledgements

The present work was part of KMM's PhD dissertation. Thank you to Dr. David Retchless for input on figure design. We thank the Florida Fish and Wildlife Conservation Commission for their help and guidance.

References

1. Brown T, Otero C, Grajales A, Rodriguez E, Rodriguez-Lanetty M. Worldwide exploration of the microbiome harbored by the cnidarian model, Exaiptasia pallida

(Agassiz in Verrill, 1864) indicates a lack of bacterial association specificity at a lower taxonomic rank. PeerJ. 2017;2017(5).

2. Van De Water JAJM, Allemand D, Ferrier-Pagès C. Host-microbe interactions in octocoral holobionts - recent advances and perspectives. Microbiome. 2018;6(1):1–28.

3. Meyer JL, Castellanos-Gell J, Aeby GS, Häse CC, Ushijima B, Paul VJ. Microbial Community Shifts Associated With the Ongoing Stony Coral Tissue Loss Disease Outbreak on the Florida Reef Tract. Front Microbiol. 2019;10(September):1–12.

4. Djeghri N, Pondaven P, Stibor H, Dawson MN. Review of the diversity, traits, and ecology of zooxanthellate jellyfishes. Mar Biol [Internet]. 2019;166(11):1–19. Available from: https://doi.org/10.1007/s00227-019-3581-6

5. Baldassarre L, Ying H, Reitzel AM, Franzenburg S, Fraune S. Microbiota mediated plasticity promotes thermal adaptation in the sea anemone Nematostella vectensis. Nat Commun. 2022;13(1).

6. Ushijima B, Gunasekera SP, Meyer JL, Tittl J, Pitts KA, Thompson S, et al. Chemical and genomic characterization of a potential probiotic treatment for stony coral tissue loss disease. Commun Biol. 2023;6(1).

7. Connelly MT, McRae CJ, Liu PJ, Martin CE, Traylor-Knowles N. Antibiotics Alter Pocillopora Coral-Symbiodiniaceae-Bacteria Interactions and Cause Microbial Dysbiosis During Heat Stress. Front Mar Sci. 2022;8(January):1–18.

8. Pollock FJ, McMinds R, Smith S, Bourne DG, Willis BL, Medina M, et al. Coral-associated bacteria demonstrate phylosymbiosis and cophylogeny. Nat Commun

[Internet]. 2018;9(1):1–13. Available from: http://dx.doi.org/10.1038/s41467-018-07275-x

9. Huggett MJ, Apprill A. Coral microbiome database : Integration of sequences reveals high diversity and relatedness of coral- associated microbes. Environ Microbiol Rep. 2019 Jun 1;11(3):372–85.

10. Puntin G, Sweet M, Fraune S, Medina M, Sharp K, Weis VM, et al. Harnessing the Power of Model Organisms To Unravel Microbial Functions in the Coral Holobiont. Microbiol Mol Biol Rev [Internet]. 2022 Oct 26 [cited 2023 Feb 24];86(4). Available from: https://journals.asm.org/doi/10.1128/mmbr.00053-22

11. Ohdera AH, Abrams MJ, Ames CL, Baker DM, Suescún-Bolívar LP, Collins AG, et al. Upside-Down but Headed in the Right Direction: Review of the Highly Versatile Cassiopea xamachana System. Front Ecol Evol. 2018 Apr 9;6:1–15.

12. Mammone M, Ferrier-Pages C, Lavorano S, Rizzo L, Piraino S, Rossi S. High photosynthetic plasticity may reinforce invasiveness of upside-down zooxanthellate jellyfish in Mediterranean coastal waters. PLoS One [Internet]. 2021 Mar 1 [cited 2021 Jun 30];16(3 March):e0248814. Available from: https://doi.org/10.1371/journal.pone.0248814

13. Béziat P, Kunzmann A. Under pressure: Cassiopea andromeda jellyfish exposed to increasing water temperature or lead, cadmium and anthropogenic gadolinium contamination. Mar Biol Res [Internet]. 2022;18(1–2):48–63. Available from: https://doi.org/10.1080/17451000.2022.2066132

14. McGill CJ, Pomory CM. Effects of bleaching and nutrient supplementation on wet weight in the jellyfish Cassiopea xamachana (Bigelow) (Cnidaria: Scyphozoa). Mar Freshw

Behav Physiol. 2008;41(3):179–89.

15. Ames CL, Klompen AML, Badhiwala K, Muffett K, Reft AJ, Kumar M, et al. Cassiosomes are stinging-cell structures in the mucus of the upside-down jellyfish Cassiopea xamachana. Commun Biol [Internet]. 2020;3(1):1–15. Available from: http://dx.doi.org/10.1038/s42003-020-0777-8

16. Bakshani CR, Morales-Garcia AL, Althaus M, Wilcox MD, Pearson JP, Bythell JC, et al. Evolutionary conservation of the antimicrobial function of mucus: A first defence against infection. npj Biofilms Microbiomes [Internet]. 2018;4(1):1–12. Available from: http://dx.doi.org/10.1038/s41522-018-0057-2

17. Klein SG, Pitt KA, Lucas CH, Hung S, Schmidt-roach S, Aranda M, et al. Night-Time Temperature Reprieves Enhance the Thermal Tolerance of a Symbiotic Cnidarian. 2019;6(July):1–16.

18. Fitt WK, Costley K. The role of temperature in survival of the polyp stage of the tropical rhizostome jellyfish Cassiopea xamachana. 1998;222:79–91.

19. Al-jbour SM, Zimmer M, Kunzmann A. Cellular respiration, oxygen consumption, and trade-offs of the jellyfish Cassiopea sp. in response to temperature change. J Sea Res [Internet]. 2017;128(January):92–7. Available from: https://doi.org/10.1016/j.seares.2017.08.006

20. Lee MD, Kling JD, Araya R, Ceh J. Jellyfish Life Stages Shape Associated Microbial Communities, While a Core Microbiome Is Maintained Across All. Front Microbiol. 2018 Jul 12;0(JUL):1534.

21. Röthig T, Puntin G, Wong JCY, Burian A, McLeod W, Baker DM. Holobiont nitrogen control and its potential for eutrophication resistance in an obligate photosymbiotic jellyfish. Microbiome. 2021;9(1):1–16.

22. Cleary DFR, Becking LE, Polónia ARM, Freitas RM, Gomes NCM. Jellyfish-associated bacterial communities and bacterioplankton in Indonesian Marine lakes. FEMS Microbiol Ecol. 2016;92(5):1–14.

23. Hao W, Gerdts G, Holst S, Wichels A. Bacterial communities associated with scyphomedusae at Helgoland Roads. Mar Biodivers. 2019;49(3):1489–503.

24. Peng S, Hao W, Li Y, Wang L, Sun T, Zhao J, et al. Bacterial Communities Associated With Four Blooming Scyphozoan Jellyfish: Potential Species-Specific Consequences for Marine Organisms and Humans Health. Front Microbiol. 2021 May 5;12(May):1–16.

25. Daley MC, Urban-Rich J, Moisander PH. Bacterial associations with the hydromedusa Nemopsis bachei and scyphomedusa Aurelia aurita from the North Atlantic Ocean. Mar Biol Res. 2016;12(10):1088–100.

26. Weiland-Bräuer N, Neulinger SC, Pinnow N, Künzel S, Baines JF, Schmitz RA. Composition of bacterial communities associated with Aurelia aurita changes with compartment, life stage, and population. Appl Environ Microbiol. 2015;81(17):6038–52.

27. Viver T, Orellana LH, Hatt JK, Urdiain M, Díaz S, Richter M, et al. The low diverse gastric microbiome of the jellyfish Cotylorhiza tuberculata is dominated by four novel taxa. Environ Microbiol. 2017;19(8):3039–58.

28. Basso L, Rizzo L, Marzano M, Intranuovo M, Fosso B, Pesole G, et al. Jellyfish summer

outbreaks as bacterial vectors and potential hazards for marine animals and humans health? The case of Rhizostoma pulmo (Scyphozoa, Cnidaria). Sci Total Environ [Internet]. 2019;692:305–18. Available from: https://doi.org/10.1016/j.scitotenv.2019.07.155

29. Stabili L, Rizzo L, Basso L, Marzano M, Fosso B, Pesole G, et al. The microbial community associated with Rhizostoma pulmo: Ecological significance and potential consequences for marine organisms and human health. Mar Drugs. 2020;18(9).

30. Delannoy CMJ, Houghton JDR, Fleming NEC, Ferguson HW. Mauve Stingers (Pelagia noctiluca) as carriers of the bacterial fish pathogen Tenacibaculum maritimum. Aquaculture [Internet]. 2011;311(1–4):255–7. Available from: http://dx.doi.org/10.1016/j.aquaculture.2010.11.033

31. Muffett K, Miglietta MP. Demystifying Cassiopea species identity in the Florida Keys: Cassiopea xamachana and Cassiopea andromeda coexist in shallow waters. PLoS One [Internet]. 2023;18(3):e0283441. Available from: http://dx.doi.org/10.1371/journal.pone.0283441

32. Laramie MB, Pilliod DS, Goldberg CS, Strickler KM. Environmental DNA Sampling Protocol — Filtering Water to Capture DNA from Aquatic Organisms. In: US Geological Survey Techniques and Methods, book 2. Reston, VA: U.S. Geological Survey; 2015. p. 1–15.

33. Pavlovska M, Prekrasna I, Parnikoza I, Dykyi E. Soil sample preservation strategy affects the microbial community structure. Microbes Environ. 2021;36(1):1–7.

34. Callahan BJ, McMurdie PJ, Rosen MJ, Han AW, Johnson AJA, Holmes SP. DADA2:

High-resolution sample inference from Illumina amplicon data. Nat Methods. 2016;13(7):581–3.

35. R-Core-Team (Foundation for Statistical Computing). R: A Language and Environment for Statistical Computing [Internet]. 2022. Available from: https://www.r-project.org/

36. Quast C, Pruesse E, Yilmaz P, Gerken J, Schweer T, Yarza P, et al. The SILVA ribosomal RNA gene database project: Improved data processing and web-based tools. Nucleic Acids Res. 2013;41(D1):590–6.

37. McMurdie PJ, Holmes S. Phyloseq: An R Package for Reproducible Interactive Analysis and Graphics of Microbiome Census Data. PLoS One. 2013;8(4).

38. Davis NM, Proctor DiM, Holmes SP, Relman DA, Callahan BJ. Simple statistical identification and removal of contaminant sequences in marker-gene and metagenomics data. Microbiome. 2018;6(1):1–14.

39. Oksanen J, Simpson G, Blanchet F, Kindt R, Legendre P, Minchin P, et al. _vegan: Community Ecology Package_. 2024.

40. Paradis E, Schliep K. ape 5 . 0 : an environment for modern phylogenetics and evolutionary analyses in R. 2019;35(July 2018):526–8.

41. Mikryukov V. metagMisc: Miscellaneous functions for metagenomic analysis [Internet]. Available from: https://github.com/vmikk/metagMisc

42. Taudiere A. MiscMetabar: Miscellaneous Functions for Metabarcoding Analysis [Internet]. 2024. Available from: https://cran.r-project.org/package=MiscMetabar

43. Tsirogiannis C, Sandel B. PhyloMeasures: Fast and Exact Algorithms for Computing

Phylogenetic Biodiversity Measures. 2017.

44. Schliep KP. phangorn : phylogenetic analysis in R. 2011;27(4):592–3.

45. Battaglia T. btools: A suite of R function for all types of microbial diversity analyses. 2025.

46. Patil I. Visualizations with statistical details: The “ggstatsplot” approach. J Open Source Softw [Internet]. 2021;6(61)(3167). Available from: doi:10.21105/joss.03167

47. Martinez Arbizu P. pairwiseAdonis: Pairwise Multilevel Comparison using Adonis [Internet]. 2017. Available from: https://github.com/pmartinezarbizu/pairwiseAdonis

48. Kassambara A. rstatix: Pipe-Friendly Framework for Basic Statistical Tests [Internet]. 2023. Available from: https://cran.r-project.org/package=rstatix

49. McLaren M. speedyseq: Faster implementations of phyloseq functions [Internet]. 2025. Available from: https://github.com/mikemc/speedyseq

50. Pebesma E, Bivand R. Spatial Data Science: With applications in R [Internet]. London: Chapman and Hall/CRC; 2023. 352 p. Available from: https://r-spatial.org/book/

51. Macvittie S, Doroodian S, Alberto A, Sogin M, Macvittie S, Doroodian S, et al. Exaiptasia diaphana , following antibiotic exposure. 2024;9(6).

52. Fernandes AD, Reid JNS, Macklaim JM, Mcmurrough TA, Edgell DR, Gloor GB. Unifying the analysis of high-throughput sequencing datasets : characterizing RNA-seq , 16S rRNA gene sequencing and selective growth experiments by compositional data analysis. 2014;1–13.

53. Holmes I, Harris K, Quince C. Dirichlet Multinomial Mixtures : Generative Models for

Microbial Metagenomics. 2012;7(2).

54. Morgan M. DirichletMultinomial: Dirichlet-Multinomial Mixture Model Machine Learning for Microbiome Data [Internet]. 2024. Available from: https://doi.org/10.18129/B9.bioc.DirichletMultinomial

55. Lahti L, Shetty S. microbiome R package. 2019.

56. Barnett D, Arts I, Penders J. microViz: an R package for microbiome data visualization and statistics. J Open Source Softw. 2021;6(63):3201.

57. Neu AT. Defining and quantifying the core microbiome : Challenges and prospects. 2021;118(51):1–10.

58. Bonacolta AM, Connelly MT, Rosales SM, del Campo J, Traylor-knowles N. The starlet sea anemone , Nematostella vectensis , possesses body region-specific bacterial associations with spirochetes dominating the capitulum. FEMS Microbiol Lett. 2021;(August 2020):1–9.

59. van Oppen MJH, Blackall LL. Coral microbiome dynamics, functions and design in a changing world. Nat Rev Microbiol [Internet]. 2019;17(9):557–67. Available from: http://dx.doi.org/10.1038/s41579-019-0223-4

60. Agostini S, Yoshimi S, Higuchi T, Casareto BE. Biological and chemical characteristics of the coral gastric cavity. Coral Reefs. 2011;(March).

61. Bollati E, Hughes DJ, Suggett DJ, Raina J-B, Kühl M. Microscale sampling of the coral gastric cavity reveals a gut-like microbial community. bioRxiv [Internet]. 2024;2024.05.20.594925. Available from:

https://www.biorxiv.org/content/10.1101/2024.05.20.594925v1%0Ahttps://www.biorxiv.org/content/10.1101/2024.05.20.594925v1.abstract

62. Carabantes N, Cerqueda-García D, García-Maldonado JQ, Thomé PE. Changes in the Bacterial Community Associated With Experimental Symbiont Loss in the Mucus Layer of Cassiopea xamachana Jellyfish. Front Mar Sci. 2022;9(May):1–13.

63. Wu W, Lei J-N, Mao Q, Tian Y-Z, Shan H-W, Chen J-P. Distribution, Vertical Transmission, and Cooperative Mechanisms of Obligate Symbiotic Bacteria in the Leafhopper Maiestas dorsalis (Hemiptera, Cicadellidea). Insects. 2023;1–13.

64. Sharp KH, Pratte ZA, Kerwin AH, Rotjan RD, Stewart FJ. Season, but not symbiont state, drives microbiome structure in the temperate coral Astrangia poculata. Microbiome. 2017;5(1):120.

65. Hammer TJ, Sanders JG, Fierer N. Not all animals need a microbiome. FEMS Microbiol Lett. 2019;366(10):1–11.

66. Riviere M La, Roumagnac M, Garrabou J, Bally M. Transient Shifts in Bacterial Communities Associated with the Temperate Gorgonian Paramuricea clavata in the Northwestern Mediterranean Sea. PLoS One. 2013;8(2).

67. Neave MJ, Apprill A, Ferrier-Pagès C, Voolstra CR. Diversity and function of prevalent symbiotic marine bacteria in the genus Endozoicomonas. Appl Microbiol Biotechnol [Internet]. 2016;100(19):8315–24. Available from: http://dx.doi.org/10.1007/s00253-016-7777-0

68. Tinta T, Kogovšek T, Klun K, Malej A, Herndl GJ, Turk V. Jellyfish-associated

microbiome in the marine environment: Exploring its biotechnological potential. Mar Drugs. 2019;17(2):1–33.

69. Maher RL, Schmeltzer ER, Meiling S, McMinds R, Ezzat L, Shantz AA, et al. Coral Microbiomes Demonstrate Flexibility and Resilience Through a Reduction in Community Diversity Following a Thermal Stress Event. Front Ecol Evol. 2020;8(October):1–15.

70. Pogoreutz C, Oakley CA, Rädecker N, Cárdenas A, Perna G, Xiang N, et al. Coral holobiont cues prime Endozoicomonas for a symbiotic lifestyle. ISME J. 2022;16(8):1883–95.

71. Neave MJ, Michell CT, Apprill A, Voolstra CR. Endozoicomonas genomes reveal functional adaptation and plasticity in bacterial strains symbiotically associated with diverse marine hosts. Sci Rep. 2017;7(December 2016):1–12.

72. McDevitt-Irwin JM, Baum JK, Garren M, Vega Thurber RL. Responses of coral-associated bacterial communities to local and global stressors. Front Mar Sci. 2017;4(AUG):1–16.

73. Neulinger SC, Gärtner A, Järnegren J, Ludvigsen M, Lochte K, Dullo WC. Tissue-associated "Candidatus mycoplasma corallicola" and filamentous bacteria on the cold-water coral Lophelia pertusa (Scleractinia). Appl Environ Microbiol. 2009;75(5):1437–44.

74. Sweet MJ, Bulling MT. On the importance of the microbiome and pathobiome in coral health and disease. Front Mar Sci. 2017;4(January):1–11.

75. Hofmann DK, Neumann R, Henne K. Strobilation, budding and initiation of scyphistoma morphogenesis in the rhizostome Cassiopea andromeda (Cnidaria: Scyphozoa). Mar Biol.

1978;47(2):161–76.

76. Neumann R. Bacterial Induction of Settlement and Metamorphosis in the Planula Larvae of Cassiopea andromeda (Cnidaria: Scyphozoa, Rhizostomeae). Vol. 1. 1979.

77. Kramar MK, Tinta T, Lučić D, Malej A, Turk V. Bacteria associated with moon jellyfish during bloom and post-bloom periods in the Gulf of Trieste (northern Adriatic). PLoS One. 2019;14(1):1–21.

78. Tinta T, Kogovsek T, Malej A, Turk V. Jellyfish Modulate Bacterial Dynamic and Community Structure. PLoS One. 2012;7(4):1–10.

79. Pollock FJ, Wilson B, Johnson WR, Morris PJ, Willis BL, Bourne DG. Phylogeny of the coral pathogen Vibrio coralliilyticus. Environ Microbiol Rep. 2010;2(1):172–8.

80. Ben-Haim Y, Thompson FL, Thompson CC, Cnockaert MC, Hoste B, Swings J, et al. Vibrio coralliilyticus sp. nov., a temperature-dependent pathogen of the coral Pocillopora damicornis. Int J Syst Evol Microbiol. 2003;53(1):309–15.

81. Richards GP, Watson MA, Needleman DS, Church KM, Häse CC. Mortalities of Eastern And Pacific oyster larvae caused by the pathogens Vibrio coralliilyticus and Vibrio tubiashii. Appl Environ Microbiol. 2015;81(1):292–7.

82. Emery MA, Beavers KM, Van Buren EW, Batiste R, Dimos B, Pellegrino MW, et al. Trade-off between photosymbiosis and innate immunity influences cnidarian’s response to pathogenic bacteria. Proc R Soc B Biol Sci. 2024;291(2032):20240428.

83. Thomé PE, Rivera-Ortega J. Specific antibacterial activity against potential pathogens and re-straining of larvae settlement from a pigmented Pseudoalteromonas strain isolated from

the jellyfish Cassiopea xamachana. Int Aquat Res. 2022;14(2):95–105.

84. Bright M, Espada-hinojosa S, Lagkouvardos I, Volland J, Hole W. The giant ciliate Zoothamnium niveum and its thiotrophic epibiont Candidatus Thiobios zoothamnicoli : a model system to study interspecies cooperation. 2014;5(April):1–13.

85. Rinke C, Schmitz-esser S, Stoecker K, Nussbaumer AD, Vanura K, Wagner M, et al. “ Candidatus Thiobios zoothamnicoli ,” an Ectosymbiotic Bacterium Covering the Giant Marine Ciliate Zoothamnium niveum. 2021;72(3):2014–21.

86. Vekeman B. Methanotrophic microbiomes from North Sea sediment.

87. Song J, Hwang J, Kang I, Cho JC. OPEN A sulfate - reducing bacterial genus , Desulfosediminicola gen . nov ., comprising two novel species cultivated from tidal - flat sediments. Sci Rep [Internet]. 2021;1–13. Available from: https://doi.org/10.1038/s41598-021-99469-5

88. Engelen AH, Aires T, Vermeij MJA, Herndl GJ, Serrão EA, Frade PR. Host differentiation and compartmentalization of microbial communities in the azooxanthellate cupcorals Tubastrea coccinea and Rhizopsammia goesi in the Caribbean. Front Mar Sci. 2018;9(NOV):1–9.

89. Meirelles LA, Newman DK. Both toxic and beneficial effects of pyocyanin contribute to the lifecycle of Pseudomonas aeruginosa. Mol Microbiol. 2018;110(6):995–1010.

90. Carlson CA, Ingraham JL. Comparison of Denitrification by Pseudomonas stutzeri , Pseudomonas aeruginosa , and Paracoccus denitrificans. 1983;45(4):1247–53.

91. Huo Y, Xu X, Li X, Liu C, Cui H, Wang C, et al. Ruegeria marina sp . nov ., isolated from

Marine Sediment. 2011;347–50.

92. Klein SG, Pitt KA, Carroll AR. Reduced salinity increases susceptibility of zooxanthellate jellyfish to herbicide toxicity during a simulated rainfall event. Environ Pollut [Internet]. 2016;209:79–86. Available from: http://dx.doi.org/10.1016/j.envpol.2015.11.012

93. Hyun DW, Shin NR, Kim MS, Oh SJ, Kim PS, Whon TW, et al. Endozoicomonas atrinae sp. nov., isolated from the intestine of a comb pen shell Atrina pectinata. Int J Syst Evol Microbiol. 2014;64(PART 7):2312–8.

94. Sunagawa S, Woodley CM, Medina M. Threatened corals provide underexplored microbial habitats. PLoS One. 2010;5(3):1–7.

Supplementary Information

Supplement A1: Full length 16S rRNA gene identities

Three samples were sent to CD Genomics for full length 16S on PacBio Sequel using universal 16S primers (F: AGRGTTTGATYNTGGCTCAG; R:TASGGHTACCTTGTTASGACTT). Samples sent for full length 16S rRNA sequencing included gastrovascular cavity swab extractions from medusae Marathon Key GVC 30 and Key West GVC 96 and GVC 120. As the usable reads returned from this were low (597-12520 per sample), these full-length identities are not discussed in the main text. For further details, please refer to the appendix (A1).

Full length 16S rRNA gene sequences (BioProject: PRJNA1020446) were run on mothur v1.48.0 with deviation from the MiSeq SOP on trim length. ASV cutoff was set to five

differences, and ASV representative sequences were compared against the NCBI GenBank (Sept 24, 2023) database using BLAST to find nearest matches by e-value.

In order to identify core microbial groups to a lower taxonomic level, three internal medusa gastrovascular swabs (medusae Kmuffet_96, Kmuffet_120 and Kmuffet_30) from Marathon Key (MK) and Key West (GB) were sequence for full length 16S rRNA gene (see S1 for location of samples). As the primers were not chloroplast exclusionary, the Key West sites produced few useable sequences (Kmuffet_96: 597 and Kmuffet_120: 1097). The Marathon Key medusa (Kmuffet_30), likely bleached at time of collection, returned 12,520 non-chloroplast sequences. The Mycoplasma-like full length 16S rRNA gene sequence (Genbank Acc. No. OR592270) from Marathon Key and Key West has no closely related sequences currently identified (greatest identity 88.43% to sequence HQ393440). The *Endozoicomonas* strain from both Marathon Key and Key West (Genbank Acc. No. OR592271) has 99.93% identity to *Endozoicomonas atrinae* strain WP70(T) collected from the intestine of South Korean *Atrina pectinata* (Accession NR_134024)(93) and 99.80% identity (<5 nucleotide differences) with isolates collected from *Gorgonia ventalina* (GenBank Acc. No. GU118345) in Bocas del Toro, Panama (94). *Endozoicomanas* and *Mycoplasma*-like isolates were the only two groups to have ASVs with over 100 sequences.

Supplementary Tables: (in Supplementary Excel Workbook)

Supplementary Table S1: Roster of collected medusae

Supplementary Table S2: Read processing tracking table

Supplementary Table S3: Representative sequences of all ASVs from the dataset

Supplementary Table S4: ASVs orderd by abundance in each sample type

Supplementary Table S5: Alpha diversity of all samples

Supplementary Table S6: Alpha diversity of all sample types with statistical groupings

Supplementary Table S7: Faiths diversity by sample

Supplementary Table S8: Statistical comparisons for Adonis2 groupings

Supplementary Table S9: Taxonomy of top 1000 ASVs in dataset

Sfig 1: Rarefaction curves of all samples

Sfig 2: Principal component analysis of Bray-Curtis distance between microbiomes of *Cassiopea* species.

Sfig 3: Upset plot of overlap between taxa found in bell, GVC, water and substrate samples.

Sfig 4: Heatmap of core microbiome prevalence in GVC and bell.

Sfig 5: Aldex2 plot of effect size of all ASVs between bell and GVC samples.

Sfig 6: Aldex2 plot of effect size of all ASVs between bell and substrate samples.

Sfig 7: Correlation plot (corrplot) of collected environmental and medusa factors across samples.

Sfig 8: Redundancy analysis plot of GVC

Sfig 9 : Redundancy analysis plot of bell samples.

Figure 1 Click here to access/download;Figure;KMM_CM_2025_Fig1.jpg

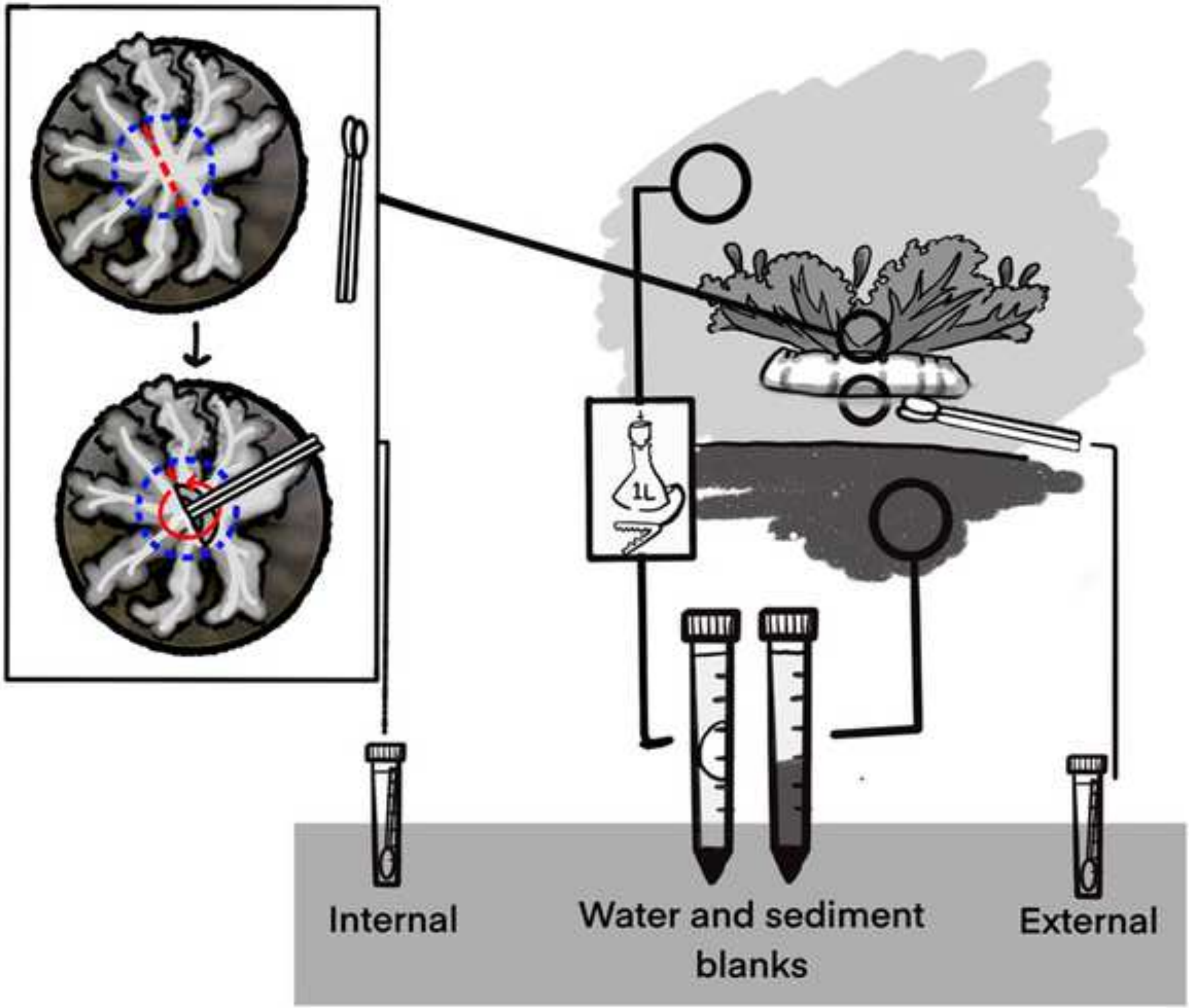

Figure 2

Click here to access/download;Figure;KMM_CM2_2025_Fig2.tif

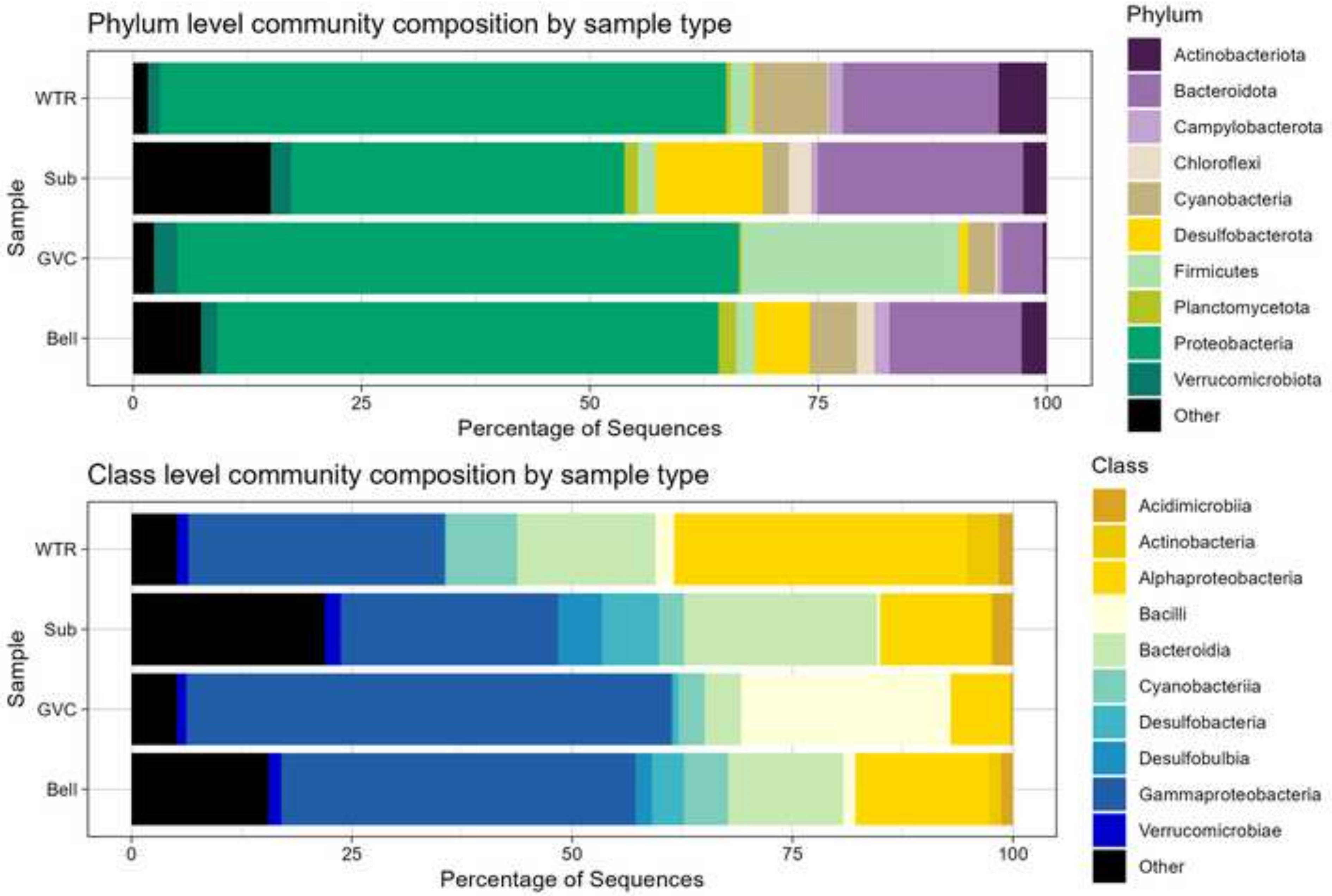

Figure 3

Click here to access/download;Figure;KMM_CM_2025_Fig3.tif

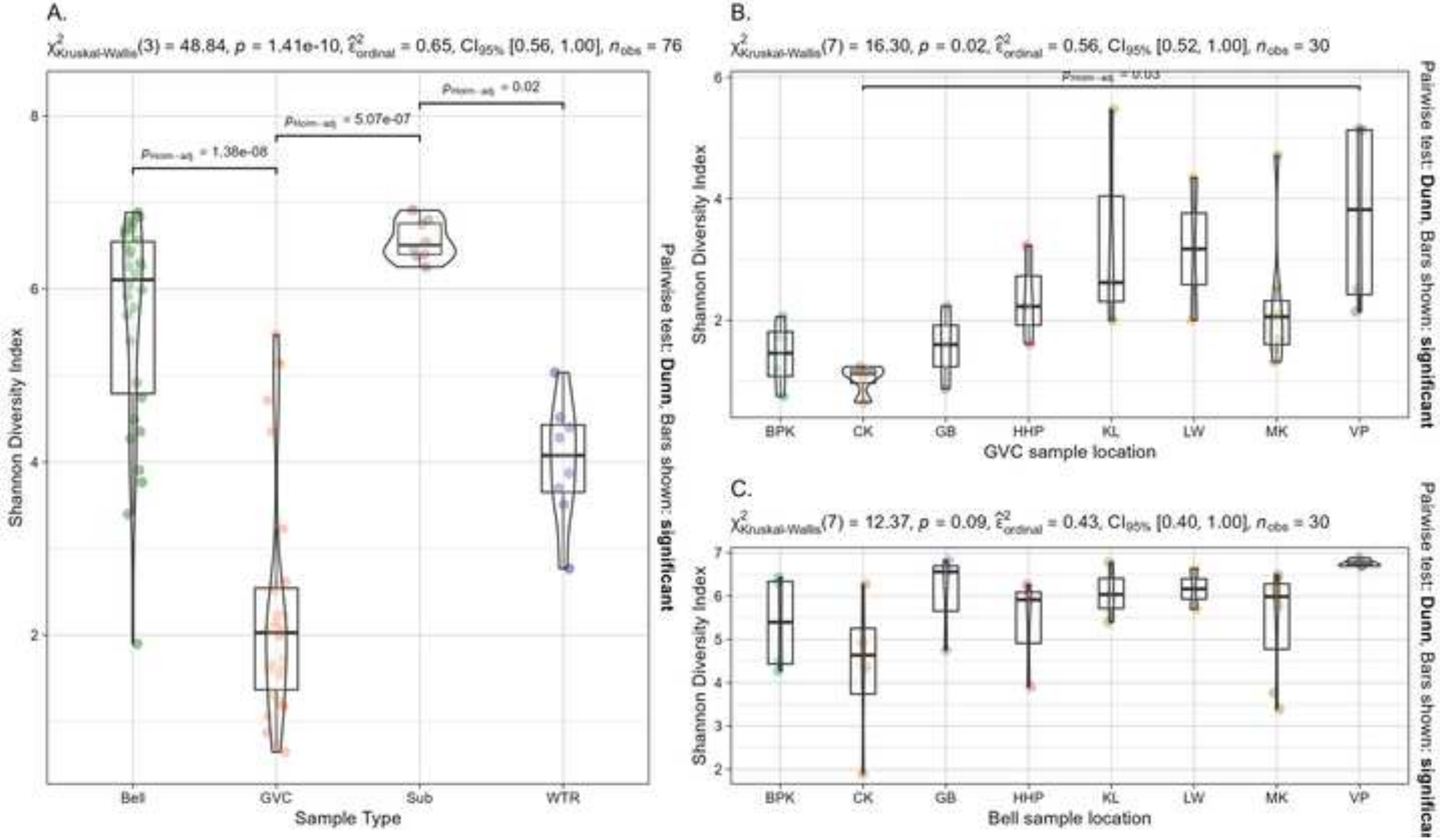

Figure 4

Click here to access/download;Figure;KMM_CM2_2025_Fig4.tif

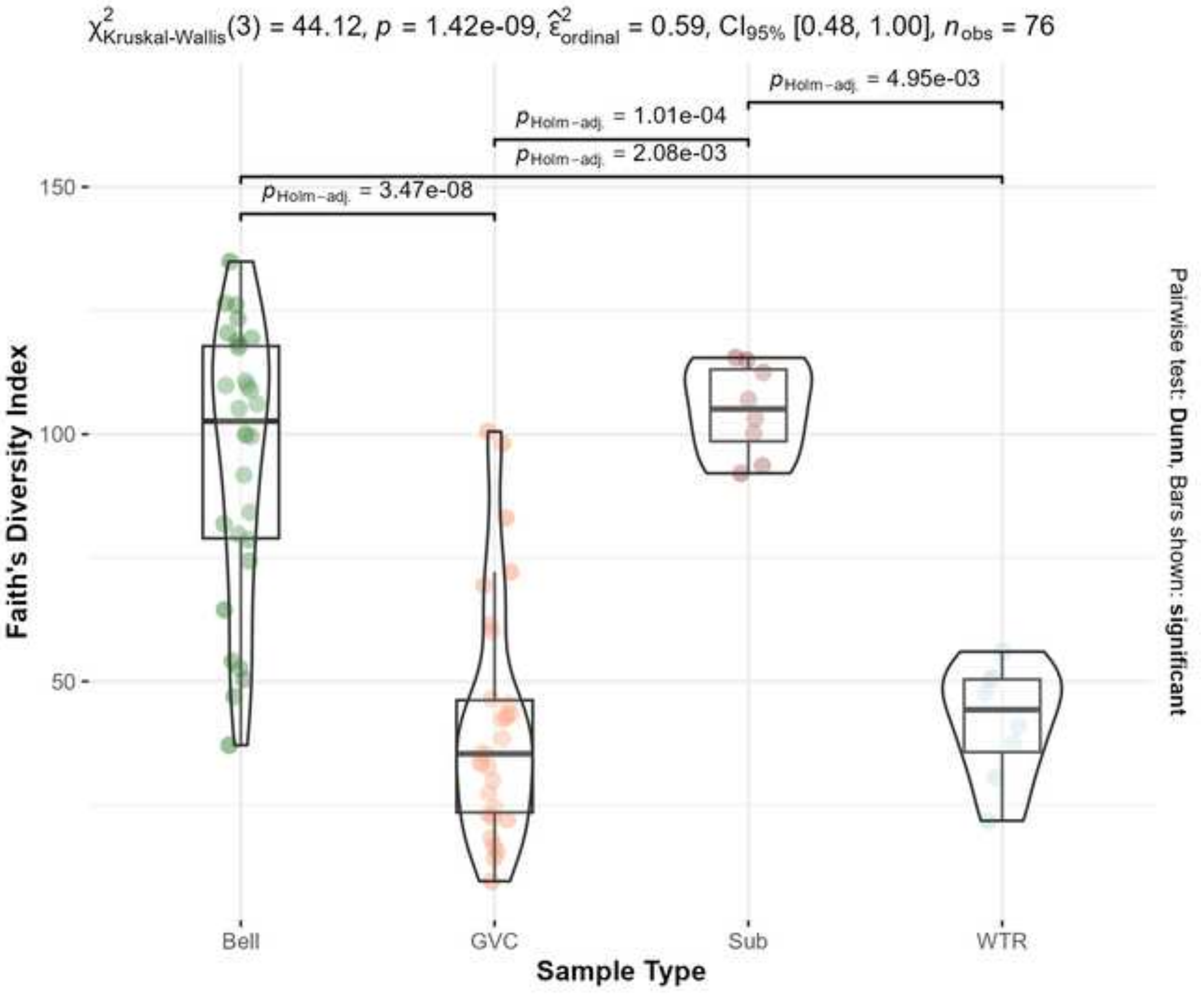

Figure 5

Click here to access/download;Figure;KMM_CM2_2025_Fig5.tif

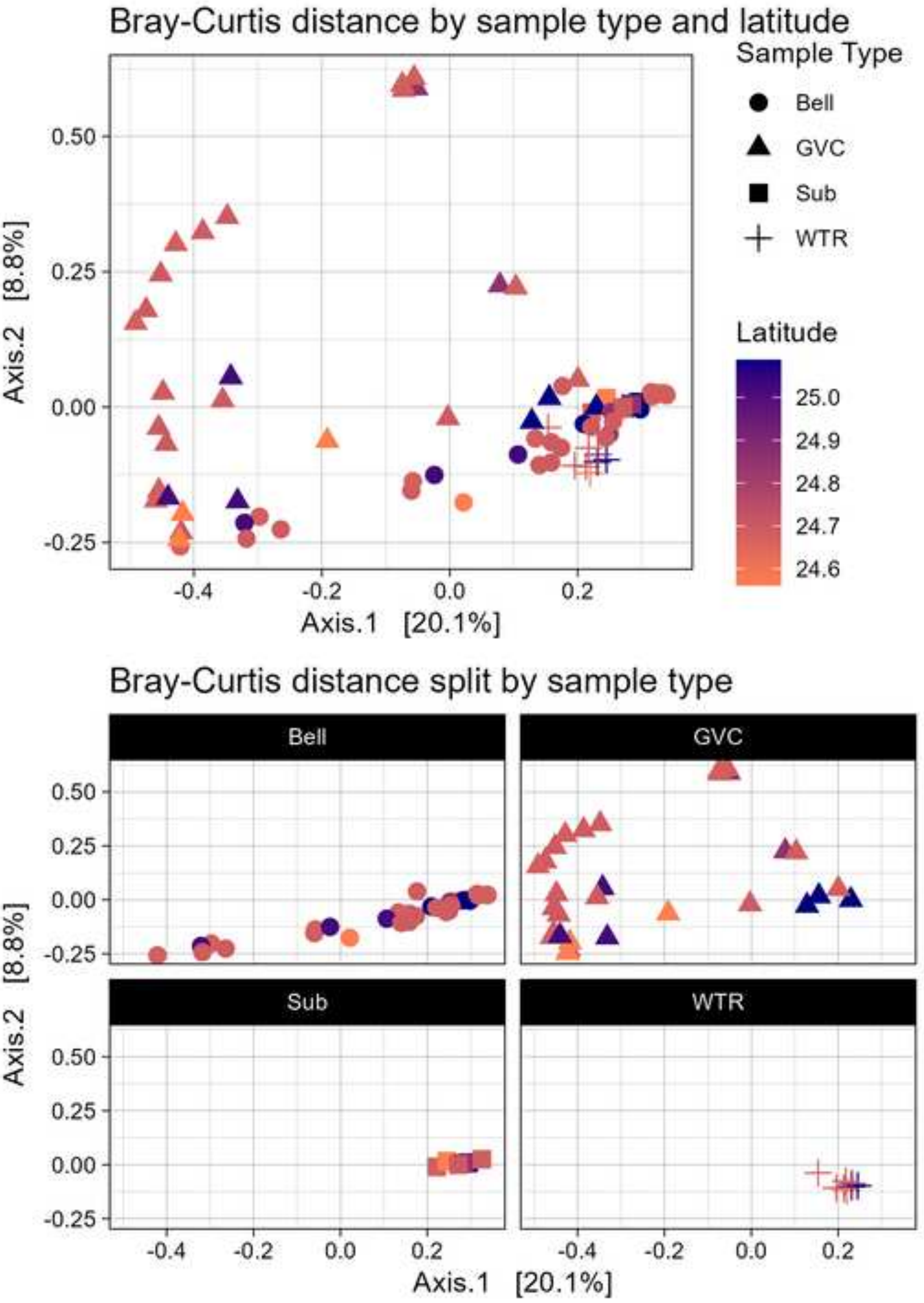

Figure 6

Click here to access/download;Figure;KMM_CM_2025_Fig6.tif

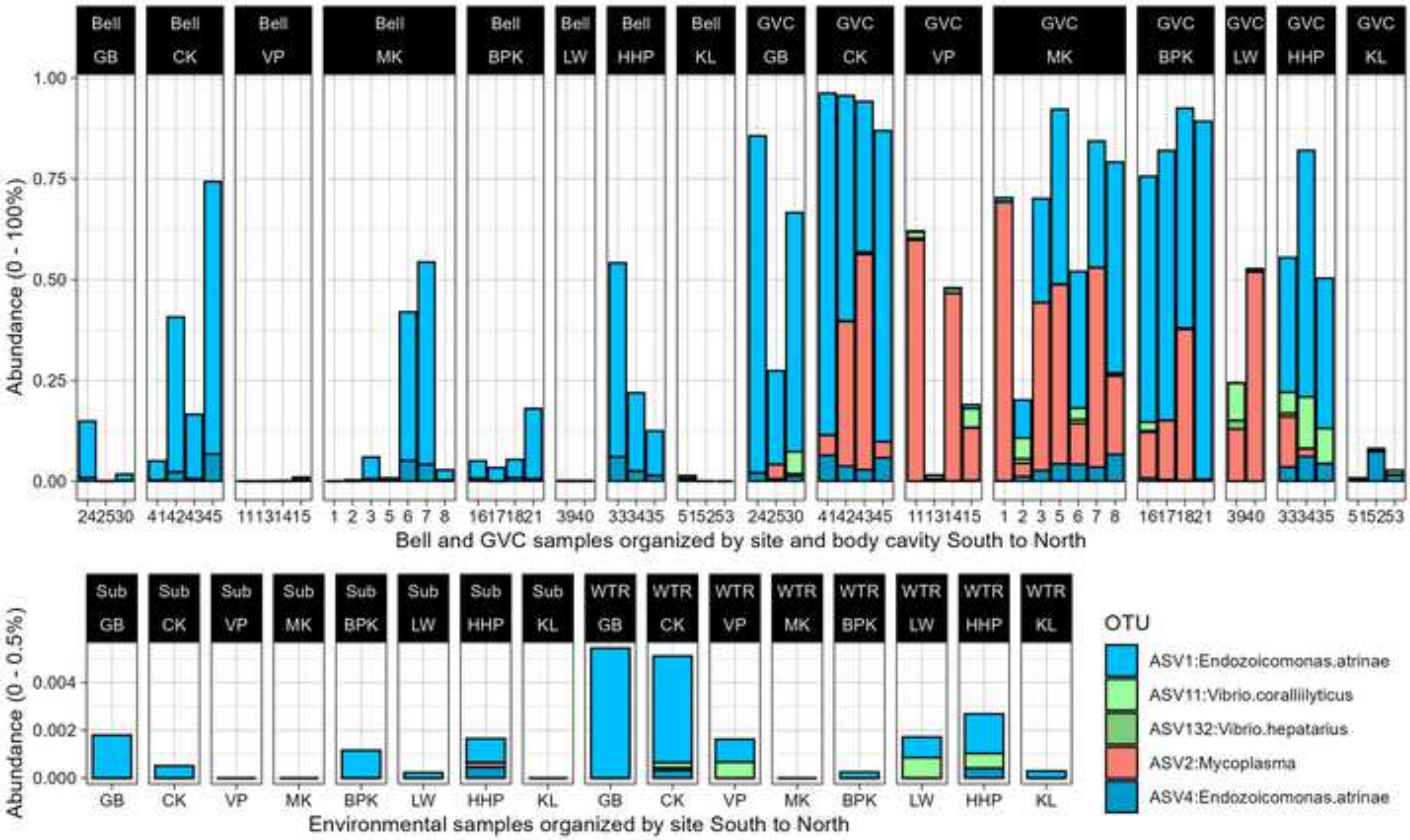

Figure 7

Click here to access/download;Figure;KMM_CM2_2025_Fig7.tif

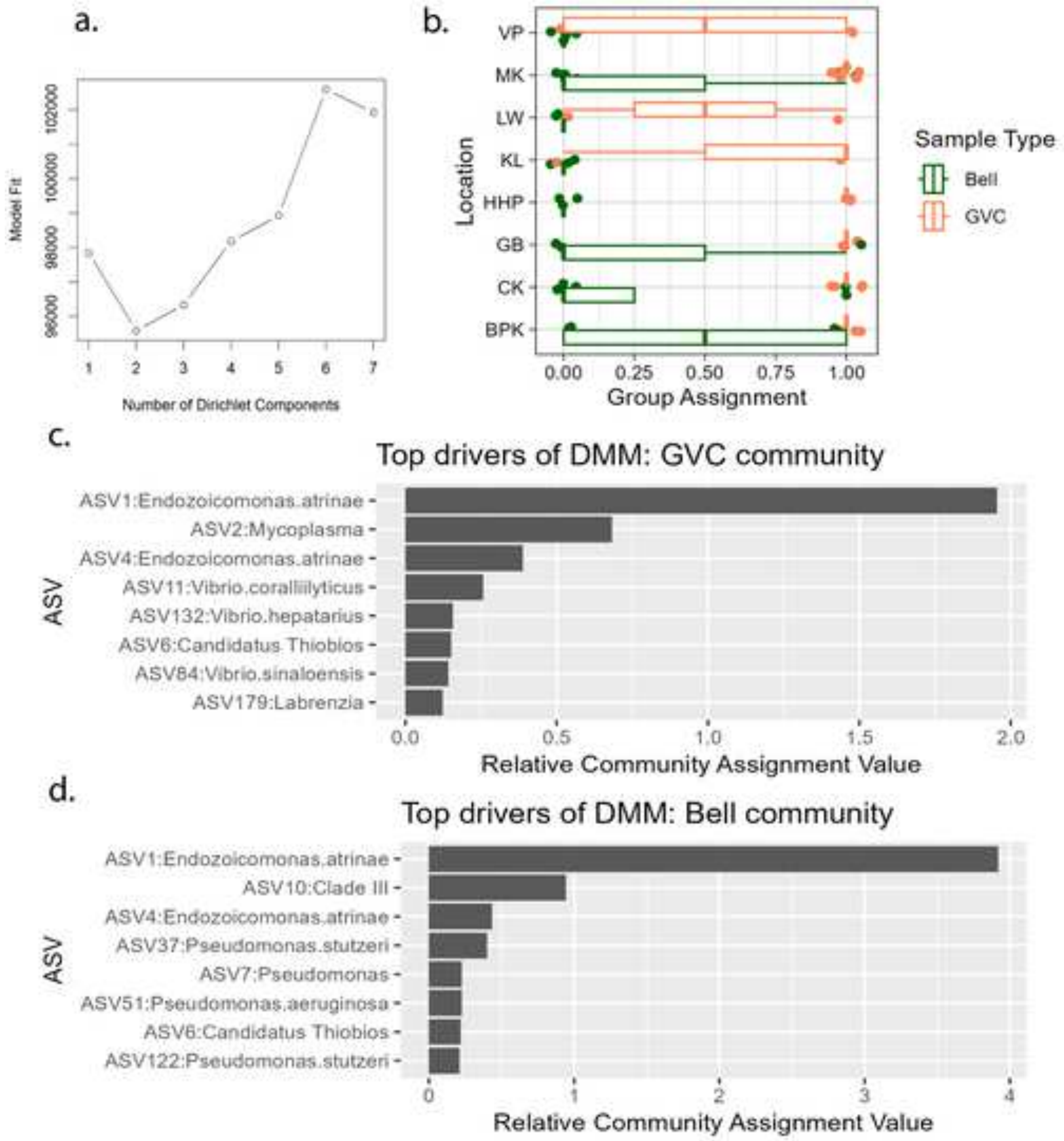

Figure 8

Click here to access/download;Figure;KMM_CM_2025_Fig8.tif

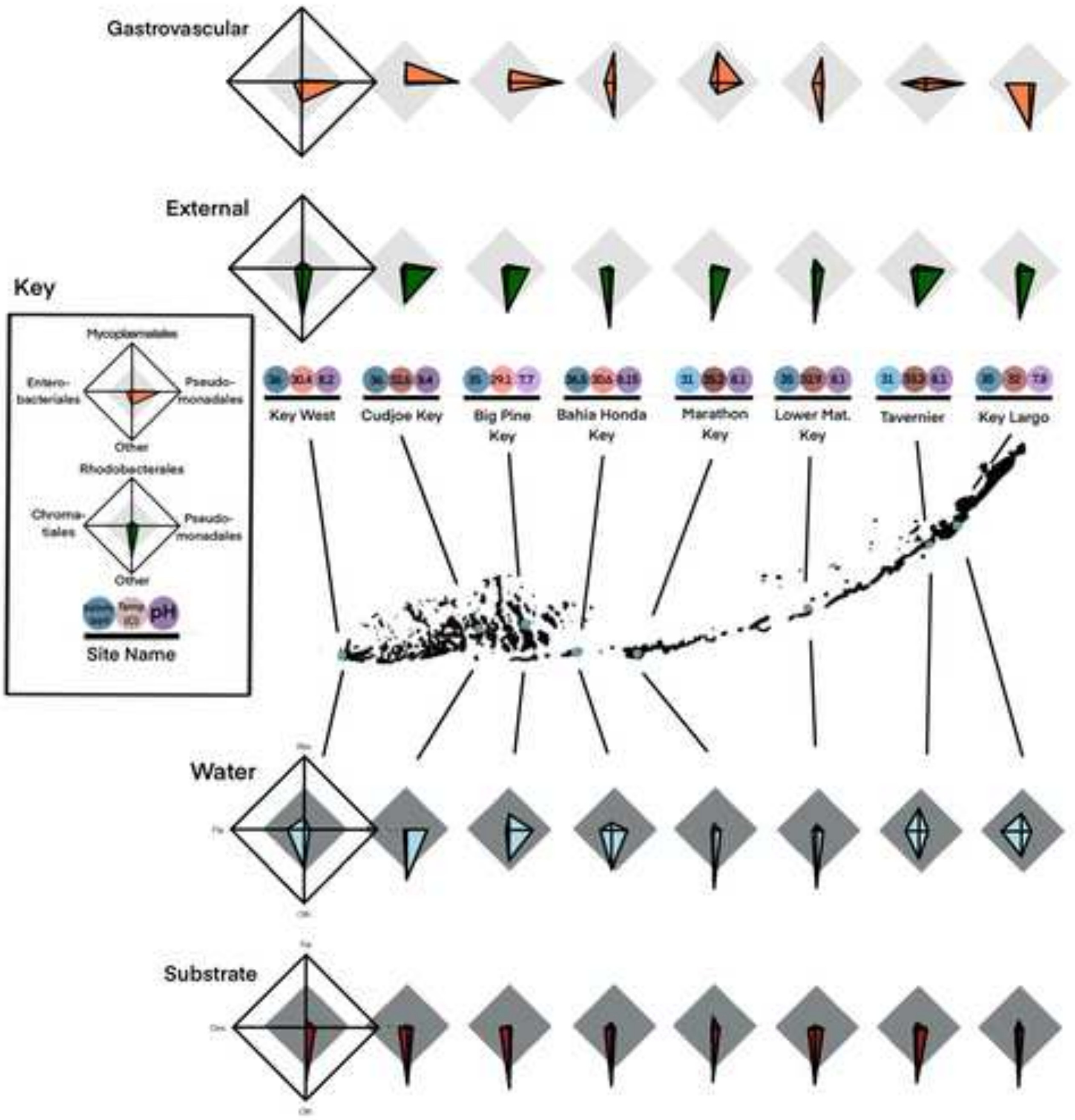

Figure 9

Click here to access/download;Figure;KMM_CM_2025_Fig9.tif

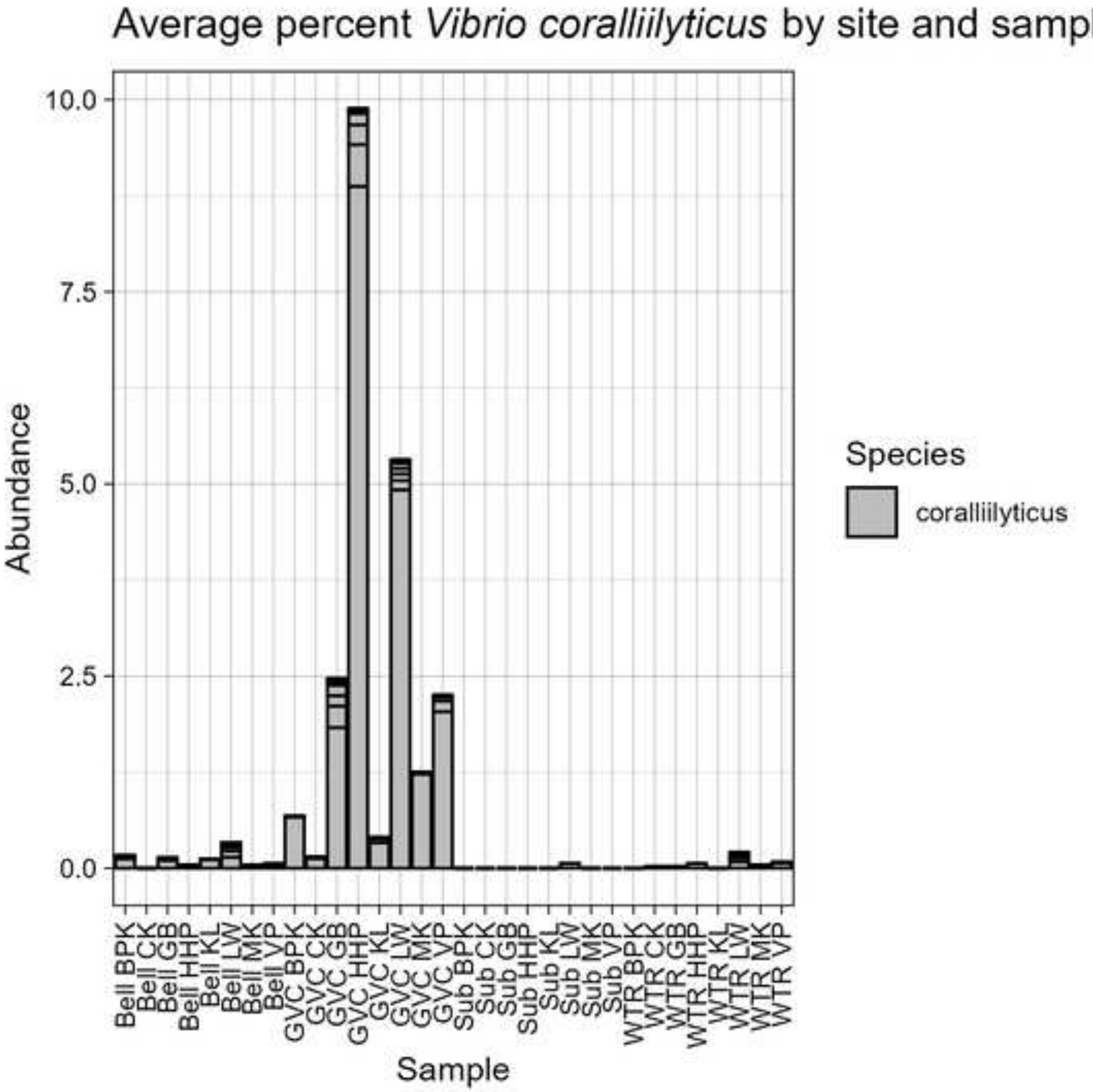

Supporting Information Tables

Click here to access/download

Supporting Information

Supplementary_Material_Microbial_Community_25.04.28.xlsx

Click here to access/download
Supporting Information
Supplementary figures_PONE_04_28_25.docx

Supporting Information Code A

Click here to access/download

Supporting Information

Cassiopea_Keys_Microbiome_workflow_Final_04.29.R

Supporting Information Code B

Click here to access/download
Supporting Information
dirichilet_model_Cass_microbiome_04.29_KMM.R

~~Gastric microbiomes of *Cassiopea* in the Florida Keys are low diversity and *Endozoicomonas*-, *Vibrio*- and *Mycoplasma*-dominated~~ Florida Keys *Cassiopea* host benthos-like external microbiomes and a gut dominated by *Vibrio*, *Endozoicommonas* and *Mycoplasma*

Formatted: Font: Italic

Formatted: Font: Italic

Formatted: Font: Italic

Formatted: Font: Italic

Kaden M Muffett[1]*, Jessica M Labonté[2], Maria Pia Miglietta[2]

1 University of California Merced, Merced, CA, 95343

2 Texas A&M University at Galveston, Galveston, TX, 77554

*Corresponding email: kmmuffett@gmail.com

Running Page Head: Bacteria of *Cassiopea* in the Florida Keys

Abstract

Interactions with microbial communities fundamentally shape metazoans' physiology, development, and health across marine ecosystems. This is especially true in zooxanthellate (symbiotic algae-containing) cnidarians. In photosymbiotic anthozoans (eg. shallow water anemones and corals), the key members of the associated microbiota are increasingly well studied, however there is limited data on photosymbiotic scyphozoans (true jellyfish). Using 16S rRNA barcoding, we sampled the internal and external mucus of the zooxanthellate Upside-Down Jellyfish, *Cassiopea xamachana* during August throughout eight sites covering the full length of the Florida Keys. We find that across sites, these medusae to have low-diversity internal microbial communities distinct from the communities of their external surfaces and their environment. These internal communities are dominated by only three taxa: *Endozoicomonas* cf. *atrinae*, an uncultured novel *Mycoplasma*, and *Vibrio* cf. *coralliilyticus*. *Cassiopea* bell mucosal samples conform largely to the communities of surrounding sediment with the addition of

Endozoicomonas cf. *atrinae*. The microbial taxa we identify associated with wild Florida Keys *Cassiopea* bear a strong resemblance to those found within photosymbiotic anthozoans, increasing the known links in ecological position between these groups.

Key words: Cnidaria, microbiome, symbiosis, ~~*Cassiopea*~~*Symbiodineaceae*, Scyphozoa

Introduction

Cnidarian-photosymbiont interactions are central to ecosystem function in coral reefs and beyond. Algal photosymbionts, or zooxanthellae, are present in ~~the~~ some well-known stony corals, octocorals, and anemones, but also play important roles in a variety of lesser-known jellyfishes, chief among them, jellyfish of the genus *Cassiopea* (1–4). For symbiotic anthozoans, microbial communities play a key role in maintaining health ~~within this~~of this photosymbiont partnership, ~~with community members directly~~ conferring disease resistance and heat tolerance on hosts (5–7). These microbial communities are often specialized, with distinct corals demonstrating co-evolution with members of their bacterial communities (8). ~~, but retain several~~Several prevalent genera, such as *Endozoicomonas*, are found across the microbiomes of many hosts (9). While anthozoan communities are under active investigation, few analyses have documented the stability and nature of microbiome communities associated with zooxanthellate jellyfishes, a group with divergent life history traits from Anthozoa but with a comparable obligate symbiosis with dinoflagellates (clade Symbiodiniaceae).

The Upside-Down Jellyfish, *Cassiopea* spp., is a model organism for cnidarian-photosymbiont interactions (10,11). These medusae are residents of tropical and subtropical waters globally and can survive in water that is frequently shallow (as little as 8 cm depth) and ~~hot~~ warm (up to 33˚C for C*assiopea*~~.~~ ~~a~~*Andromeda* (Forskal, 1775)) (12,13). *Cassiopea* obligatorily hosts algal symbionts, losing mass even when well fed while in aposymbiotic conditions (14). As in corals and other scyphozoans, mucus plays a central role for *Cassiopea*, presenting an interface and barrier ~~sticky interface~~ between the host tissue and the~~ir~~ ~~benthic~~ marine environment (15,16). In contrast to other symbiotic cnidarians, such as photosymbiotic

Formatted: Font: Not Italic

stony corals, *Cassiopea* can tolerate ~~heat~~ large temperature ranges (18C to 33C) and intense temperature fluctuations (+/- 6C) (17–19). Understanding the *Cassiopea* natural microbial community could provide new potential insights into the function and diversity of cnidarian holobionts.

Past jellyfish (class Scyphozoa) microbial studies have focused on the microbiome of lab-reared specimens (20,21) or limited sample sizes (22–24). While these types of studies may be able to track state changes across life stages (20) and between condition types (symbiotic/aposymbiotic) (21), there is little guarantee that the microbial communities of jellyfish kept under prolonged laboratory conditions translate bear resemblance to *in situ* microbiomes. Recent *in situ* studies have provided insights into microbial taxa associated with some scyphozoans ("true jellyfish") but have not included species of the genus *Cassiopea*. In the moon jellyfish, *Aurelia* spp., individuals from the Chinese coast were *Vibrionaceae*-dominated, while samples from the northwestern Atlantic were *Mycoplasma*-dominated(24,25). Between the Indonesian marine lakes Kakaban, Haji Buang, and Tanah Bamban, *Mastigias* sp. microbial community composition varied significantly by lake, but consistently included *Oceanospirillales* (primarily *Endozoicomonas*-like) (22). ~~In samples across taxa~~Across hosts, *Mycoplasma*-like bacteria are- ~~is a~~ common components of the identified gut and tissue microbiome of jellyfishes, though ~~its~~ their functions remains unknown (24–29). Vertebrate pathogen research is often a focus of scyphozoan microbiome discovery—*Tenacibaculum* has been identified in several scyphozoan species, e.g. *Cotyllorhiza tuberculata, Aurelia aurita,* and *Pelagia noctiluca*, and, as a fish pathogen, which can have important fisheries ramifications (24,25,27,30).

As most studied jellyfish populations occur in coastal waters only sporadically, the relative consistency of *Cassiopea* assemblages provides a system in which ~~location effects can~~

~~be analyzed at single time points.~~ body compartment microbiomes can be studied without the confounding impacts of single source sites. *Cassiopea* spp. presents an opportunity to act as a comparison group for the microbial communities associated with both other scyphozoans and symbiotic anthozoans. The ubiquity of *Cassiopea* assemblages within the Florida Keys allows for the examination of differences in the microbial community of one population (Florida Keys *Cassiopea*) at one time point across a broad geographic area.

The aim of this work is to undertake a first exploratory study of the wild microbiome of *Cassiopea*, and to identify the core components of the internal and external mucosal microbiomes of *Cassiopea* ~~and~~ within ~~compare the microbial communities of *Cassiopea* across sites of~~ the Florida Keys.

Formatted: Font: Italic

Materials and Methods

Sampling

A total of 55 *Cassiopea* medusae were collected by wading in eight sites along the length of the Florida Keys in late August 2021 over eight days (see Table 1 for a list of sites). Species identification for each individual is reported in Muffett & Miglietta 2023 and summarized in Supplementary Table S1 (31). At each site, coordinates, temperature, time of day, depth, salinity, and pH were recorded (Table 1). Salinity was measured with a refractometer, and temperature and pH were measured with an Apera A1209. A minimum of two and a maximum of 10 medusae were sampled per site. At each site and in between each medusa, all equipment was cleaned with absolute ethanol and 10% bleach solution. A 1 L water sample was collected from above (>10 cm above the benthos) each medusa aggregation and filtered (0.2 um pore size,

Thermo Scientific Cat No.09-740-30G) with a manual sampler as described in the USGS manual water sampling protocol (32), replicate filters were placed in 15 mL of ~~absolute ethanol or~~ dimethyl sulfoxide ethylenediamine tetraacetic acid saturated salt storage solution (1 L pH 7.5: 93.06 g EDTA, 60 mL 20 % NaOH solution, 20 mL 25 % HCl, 40 mL DMSO, 800 mL water, NaCl to oversaturation; see ref. 30) (commonly referred to as DESS), one filter in each buffer. At each *Cassiopea* medusa aggregation, duplicate scrapings of ~10 g of sediment collections (substrate sample) were placed in ~~either~~ 15 mL of ~~absolute ethanol or~~ DESS storage solution.

Table 1: List of collection sites. Table with Site Name (the collection location), site code (the shorthand used for these locations named for local landmarks, see supplementary table), latitude, longitude, salinity (parts per thousand), pH, surface temperature (in Celsius), average diameter of collected medusae from the site (centimeters), and date and time of collection. Number of paired ~~GVC~~ gastrovascular cavity (GVC) and bell swab samples per collection are for *Cassiopea xamachana* (CX). For collections that included *C. andromeda* mitotyped medusae, the number of *C. andromeda* samples is presented after the dash (CA). These values only include samples collected in DESS. Due to a storm warning, collections from Bahia Honda occurred across two days (TP1, TP2).

Site Name	Site code	Latitude	Longitude	Salinity (ppt)	pH	Temp (C)	Average depth (m)	Average col. medusa diam (cm)	# of GVC Samps	# of Bell Samps	Date (AM/PM)
Key West	GB	24.561526	-81.7881727	36	8.2	30.4	0.56	6.62	CX 3/ CA 1*	CX 3/ CA 1*	15.8.2021 PM
Cudjoe Key	CK	24.6775378	-81.4991819	36	8.4	32.6	0.36	5.91	CX 4/ CA 2*	CX 4/ CA 2*	17.8.2021 AM
Big Pine Key	BPK	24.6978731	-81.3572574	35	7.7	29.1	0.15	4.43	CX 4	CX 4	15.8.2021 PM
Bahia Honda Key TP1	VP	24.6825793	-81.2293673	37	8.3	31.8	0.86	12.41	CX 2	CX 2	13.8.2021 PM
Bahia Honda Key TP2	VP	24.6825793	-81.2293673	36	8	29.4	0.51	10.28	CX 2	CX 2	15.8.2021 AM
Marathon Key	MK	24.69396	-81.0980507	31	8.1	35.2	0.08	6.53	CX 7	CX 7	11.8.2021 PM
Lower Matacumbe Key	LW	24.8582157	-80.7267927	35	8.1	32.9	0.89	11.40	CX 2	CX 2	16.8.2021 PM
Tavernier	HHP	25.0233783	-80.4940316	31	8.1	33.3	0.35	12.26	CX 3	CX 3	16.8.2021 AM
Key Largo	KL	25.0872104	-80.4415797	35	7.8	32.0	0.56	4.30	CX 3/ CA 1*	CX 3/ CA 1*	18.8.2021 AM

Formatted: Width: 11", Height: 8.5"

Formatted: Centered

Formatted Table

Formatted: Centered

Formatted: Centered

Formatted: Centered

Formatted: Centered

Formatted: Centered

Formatted: Centered

Formatted: Centered

Formatted: Centered

Formatted: Centered

Each *Cassiopea* medusa was collected from shallow waters <1.5 m within a single assemblage at each site. Medusae were gently grasped by the oral arms, lifted from the water, swabbed along the apex of the bell, then placed bell-down onto a pre-prepared dissection plate (Figure 1). External bell swabs were done in replicate and placed in 3 mL DESS~~ or absolute ethanol~~. Medusa oral arm base was bisected between oral arm groups, and replicate swabs were inserted and circled the digestive cavity (hereafter referred to as gastrovascular cavity or GVC). Medusae < 3 cm in diameter were not sampled for gastrovascular cavity and bell microbiome. A total of 46 medusae were sampled. One internal and one external swab ~~was~~ from each medusa was placed in DESS~~, the other in ethanol, as with external swabs~~. A section of bell tissue was then collected and placed in ethanol for mitochondrial species confirmation (see 28). The size of each medusa was measured, and individual medusae were photographed. As sampling was done in a nonsterile environment, blanks for buffer with swab and buffer with filter were also taken.

Figure 1: Sampling strategy for medusae collection onsite. Demonstration of the typical orientation of medusa (right upper) and swab locations (apical bell surface and internal to medusa). ~~GVC~~ Gastrovascular cavity (GVC) access strategy is displayed on the left and relevant environmental samples (water filter, substrate sample) are displayed on the right.

DNA Extraction and Sequencing

Samples in DESS ~~and EtOH~~ were maintained at room temperature for three to ten days and then frozen. DESS can be stored at room temperature for at least thirty days without significantly compromising community integrity (33). DNA from all samples (see Supplementary Table S1) was then extracted using Zymobiomics DNA Kits (PN# D4300T). All samples were pre-tested through the ~~ampliciation~~ amplification of the 16S rRNA gene (variable

region V4)~~ amplifications~~. Only samples that produced visible bands for the V4 region were sent for 16S rRNA V3-V4 preparation and sequencing, with a maximum of 7 specimens per site. In total, the DNA from 34 paired medusa internal and external microbiomes were sent for sequencing.~~. ~~ ~~While DESS preserved samples yielded good amplification results, EtOH preserved samples largely failed to produce adequate amplification. As such, only DESS-stored samples were analyzed. ~~Sequencing was performed by the University of Texas Medical Branch Sequencing Lab. Library was prepared with Zymo Quick 16S V3-V4 kit (PN #D6400) in 20 uL reactions (10 uL Quick-16S qPCR premix, 4 uL Quick-16S V3-V4 primers (341f: (CCTACGGGDGGCWGCAG, CCTAYGGGGYGCWGCAG) and 806r: (GACTACNVGGGTMTCTAATCC)), 4 uL water and 2 uL sample). qPCR was run using Roche LightCycler 480 Instrument II (10min 95C, [30sec 95C, 30sec 55C, 3min 72C]x20). PCRs were enzymatically cleaned according to Zymo Quick-16S kit protocols, and barcodes were added in a secondary reaction at 20 uL (10 min 95˚C, [30 sec 95˚C, 30 sec 55˚C, 3 min 72˚C]x5). Plates included two replicate microbial community standards (Zymobiomics Cat. No. D6300) and two PCR negative controls. Samples were loaded at 10 pM, with 15% 10 pM PhiX sequencing control, and run on Illumina MiSeq with a 600 cycle v3 kit.

Data preprocessing

Data generated was run through the DADA2 v 1.26 pipeline on R v 4.4 with only limited deviations from the standard protocols for amplicon sequence variant (ASV) generation(34,35). Briefly, sequences were filtered and trimmed ("filterAndTrim(truncLen=c(285,230), maxN=0, maxEE=c(4,4), truncQ=2,trimLeft = c(17,21)"), errors were error corrected ("learnErrors", "dada"), and sequences were merged ("mergePairs"). Reads between 380 and 458 bp were retained. Chimeric reads were removed ("removeBimeraDenovo"), then taxonomy was assigned

using the Silva v 138.1 database (“assignTaxonomy”) (36). ~~Retention metrics were retained~~Metrics on number of sequences passing quality control~~ and these metrics~~ along with the full code can be found on the github (https://github.com/kadenmuffett/Cassiopea-Code-Microbiome-Repository). Sequence and metadata tables were imported into phyloseq v 1.42, where reads assigned to mitochondria and chloroplast were removed. Contaminants were removed using the decontam package v. 1.18, identifying and removing 47 ~~ASVs~~ amplicon sequence variants based on differential abundance in swab and buffer controls (37,38).

Based on host genotyping, samples not belonging to *Cassiopea xamachana* (Bigelow, 1892) were removed. ~~For all downstream analyses, dataset was limited to those stored in DESS and medusae without a *C. andromeda* mitotype. This~~ The dataset includes 60 paired bell and GVC samples (30 of each), 8 water samples and 8 substrate samples with 6.47 mil sequences across 34,808 taxa. The complete dataset (NCBI Bioproject Accession number PRJNA1020388) contains 12.78 million reads.

Formatted: Font: Italic

Statistical analyses

~~Refraction~~ Rarefaction curves were generated in vegan 2.6.6.1 (“rarecurve”) (Sfig~~FIG~~. 1)(39). Shannon diversity was computed with vegan and observed diversity was ploted with phyloseq (“estimate_richness”, “plot_richness”)(37). In order to identify the degree to which diversity spanned primarily shallow or deep phylogenetic distances, Faith’s phylogenetic diversity was calculated for all samples and sample types. F~~F~~aith’s phylogenetic diversity was computed using the MiscMetabar v 0.10.1, phanghorn v 2.12.1, metagMisc v 0.5.0 and PhyloseqMeasures v 2.1 packages by first generating an ML tree from all sequences over 300 reads across the dataset, then computing per sample Faith’s diversity (40–45). Faith’s diversity

was displayed with ggstatsplot v 0.13.0 (46). Bray-C-curtis and Euclidean distance were computed in phyloseq with “distance” to “ordinate” to “plot_ordination”.– Between group comparisons were completed with the pairwiseAdonis package v 0.4.1 (“ pairwise.adonis2”) on Euclidean Bray-Curtis distance measurements (47). Statistical comparisons were computed with Kruskal-Wallis using the Kruskal.test and pairwise comparisons using the “kruskal.test” and “dunn.test” commands with holm p adjustment (48).

Data transmutation for starplots and display was assisted by the speedyseq package v 0.5.3.9021 (49). Star plots were made using the stars package v 0.6-7 (50).– Differential abundance was calculated with ALDEx2 v 1.38.0 following established pipelines for microbiome data (51,52).

In order to identify whether a gastrovascular/ bell split community partitioning was appropriate for all samples, and identify subcommunity level enterotypes within *Cassiopea* internal and external microbiomes, Dirichlet Multinomial mixture analysis, as described by Holmes and implemented in the DirichletMultinomial package v 1.48 was used for *Cassiopea* – derived samples (53,54). In short, likelihood models were built based on transformed (log10) abundances for ASVs, then an optimal number of distinct community types was selected based on minimum Laplace values (“mcapply(dmn)”). New models were then trained under known variable constraints (GVC or bell sample identity), and crossvalidated (“cvdmngroup()”).

In order to identify taxa consistently present across samples of the bell and gastrovascular cavity, core microbiome was calculated for internal and external communities of *Cassiopea*. Core microbiome was computed with the microbiome package v 1.28.0(55). For environmental variable interpretation, a redundancy analysis was conducted on center log ration transformed microbial abundances using the microViz package (56).

Formatted: Font: Italic

Formatted: Font: Italic

Formatted: Font: Italic

Ethics Statement

This work was performed on lower level invertebrates not subject to Animal Use Protocols or within the purview of Texas A&M University IRB approval. Permitting for these specimens was waived by Florida Fish and Wildlife Conservation Commision.

Results

Data description

We analyzed paired ~~the~~ microbiome samples associated with 30 ~~4~~ medusae from eight sites within the Florida Keys. For each medusa, we analyzed the microbial composition of their gastrovascular cavity (“GVC”) ~~(GVC)~~ and external or bell mucus (herein referred to as “bell”), as well as their substrate (“Sub”) ~~–~~ or water environment (“WTR”). ~~All medusae were identified as *Cassiopea xamachana*, with the exception of four medusae identified as mitochondrial haplotype *C. andromeda* found in Key West, Cudjoe Key, and Key Largo (see Supplementary Table S1).~~ The four collected paired *C. andromeda* sample sets were excluded from the primary dataset, however they showed no divergence from the trends of the *C. xamachana* simultaneously collected (Supplementary table S1, Sfig2).

Formatted: Indent: First line: 0.5"

Formatted: Font: Not Italic

Formatted: Font: Italic

Formatted: Font: Not Italic

After filtering, 10.13 and 9.07 million reads were retained ~~after filtering~~ (“filterAndTrim”) and merging (“mergePairs”) respectively (for all ~~retention~~ sequence quality statistics, see supplementary table S2) . There were 8.91 million sequences after chimera removal. After primary phyloseq filtration, the dataset included 6.47 million sequences retained in 34,808 unique ASVs across 76 total samples (30 bell, 30 GVC, 8 water, 8 substrate). Reference sequences for top ASVs are also available in the supplementary material (table S3).

Distribution of sequences in full dataset

The dataset generated was predominantly bacterial with few archaeal reads. At the phylum level, 57% of sequences were Pseudomonadota (formerly Proteobacteria), 7% were Bacillota (Firmicutes), 13% were Bacteroidota, and 4% were ~~Cyanobacteria~~ Cyanobacteriota (Fig 2). No other phylum had greater than 3% relative abundance in the dataset.

Figure 2. Top ten ~~(top)~~ phyla and ~~(bottom)~~ class relative abundances of sequences in samples aggregated by sample type. Gastrovascular cavity and bell samples are n=30 samples. Water and sediment samples are n=8.

Formatted: Font: (Default) Times New Roman, 12 pt, Font color: Auto, Text Fill

Formatted: Font: Not Bold

At the class level, Gammaproteobacteria comprised 41% of all sequences. Alphaproteobacteria, the next most abundant, comprised 17%. Bacteriodia (12%), Bacilli (6%), Cyanobacteriia (4%), and Desulfobacteria (3%), were the other classes with a relative abundance above 2%. No Archaean group was prevalent above a relative abundance of 0.25%, with the three most abundant classes being Halobacterota (0.25%), Thermoplasmatota (0.21%) and Nanoarchaeota (0.22%).

Distribution of sequences within gastrovascular cavity ~~(GVC)~~ samples

Within gastrovascular cavity samples, 75% of all sequences belonged to just three orders, Pseudomonadales (39%), Mycoplasmatales (24%), and Enterobacterales (13%). The majority of Pseudomonadales sequences identified within the GVC were dispersed across very few OTUs, with 66% identified as *Endozoicomonas atrinae* ASV1 and 8% as *E. atrinae* ASV4. This was

true of the Mycoplasmatales as well, with 77% of associated gut sequences identified as *Mycoplasma* sp. ASV2 or *Mycoplasma* sp. ASV9 (15%).

Distribution of sequences within bell mucus samples

At the phylum level, within the bell mucosal swabs, 55% of the sequences were ~~Proteobacteria~~Pseudomonadota, 14.5% Bacteroidota, 5.1% Cyanobacteriodota~~a~~, and 6% Thermodesulfobacteriota (Desulfobacterota).

At the order level, the most abundant groups were Pseudomonadales (23%), Chromatiales (6%), Rhodobacterales (7%), Desulfobacterales (5%), and Flavobacteriales (5%). *Endozoicomonas atrinae* ASV1 was the single most abundant surface mucus ASV at a mean of 12%. The next most abundant were Synechococcus CC9902 ASV15 (1.3%), *Endozoicomonas atrinae* ASV4 (1.1%) and Chromatiales *Candidatus Thiobios* ASV6 (0.8%).

Substrate and water samples

Water samples shared some ASVs with the bell mucus of medusae, the largest contributing ASV was Chromatiales *Candidatus Thiobios* sp. ASV6 (8%). Additional prominent taxa were Rhodobacteraceae *HIMB11* ASV12 (6%), *HIMB11* ASV19 (5%), and SAR11 Clade III ASV 10 (4%). By contrast, substrate samples had no ASVs surpassing a mean of 1% abundance across sites, with the highest two ASVs Sandaracinaceae ASV94 and *Actibacter* ASV105 reaching 0.6% mean abundance across sediment samples (see table 2; extended table is table S4). Overlap was substantial between bell and sediment samples was higher than between any other group sets (Upset plot of overlaps in sfig. 3)

Formatted: Indent: First line: 0.5"

Formatted: Font: Bold

Formatted: Indent: First line: 0", Line spacing: Multiple 1.08 li

Table 2: Top five~~5~~ ~~ASVs~~ amplicon sequence variants for each sample type. Table with ASVs ordered from highest to lowest average proportion in samples of each type displayed as a percentage of composition within that sample type. Includes Silva 138 taxanomic assignment for each ASV.

Formatted: Indent: First line: 0"

	1	2	3	4	5
	ASV1 *Endozoicomonas atrinae*	ASV15 *Synechococcus* CC9902	ASV4 *Endozoicomonas atrinae*	ASV6 *Candidatus Thiobios*	ASV10 SAR11 *Clade III*
Bell	11.585 %	1.272 %	1.105 %	0.832 %	0.709 %
	ASV94 Sandaracinaceae	ASV105 *Actibacter*	ASV95 Desulfocapsaceae	ASV169 *Robiginitalea*	ASV358 *Clostridiisalibacter*
Sub	0.625 %	0.605%	0.566%	0.492%	0.474%
	ASV6 *Candidatus Thiobios*	ASV12 Rhodobacteraceae *HIMB11*	ASV19 Rhodobacteraceae *HIMB11*	ASV10 SAR11 *Clade III*	ASV18 *Marinobacterium*
WTR	7.521 %	5.839%	4.725%	4.497%	2.711%
	ASV1 *Endozoicomonas atrinae*	ASV2 *Mycoplasma*	ASV9 *Mycoplasma*	ASV4 *Endozoicomonas atrinae*	ASV11 *Vibrio coralliilyticus*
GVC	34.133%	20.099%	2.823%	2.327%	2.094%

Diversity

Shannon diversity varied by sample type (Kruskal-Wallis: chi-squared = 48.8, df = 3, p-value =1.412e-10). ~~Shannon diversity had a mean of 2.30 in GVCs, 4.01 in water samples, 5.63 in substrate samples and 6.56 in bell samples.~~ Shannon diversity indices for the gastrovascular samples varied from substrate samples and bell samples (Kruskal-Wallis Dunn test with Holm correction: GVC-Bell, p-val=0.00; GVC-Sub, p-val= 0.00; GVC-Water, p-val=0.07; Bell-Sub, p-val=0.07; Bell-Water, p-val=0.06; Water-Sub, p-val=0.01). Shannon diversity was not explained by location (Kruskal-Wallis: chi-squared = 9.7, df = 7, p-value =0.21)(Fig. 3, full diversity metrics tables in supplement s5 and s6).

Figure 3. Shannon diversity (a) by sample type (a) and (b + c) location (b+c). GVCGastrovascular cavity, bell, substrate and water Shannon diversity are shown across both the entire dataset or and when segmented by sample type across sites with (b) GVC (b) and (c) bell (c) alpha diversity across sites. All p values of holm-adj Dunn comparison of means tests below a = 0.05 are displayed, with statistical test details displayed above each plot. Means and interquartile ranges are marked with box plots for each sample type.

Faith's phylogenetic diversity of communities were also distinct from each other (Kruskal-Wallis test, chi-squared = 44.1, df = 3, p-value = 1.4e-09). GVC communities were distinct from bell and substrate comparison groups, while bells were distinct from water and gastrovascular cavities (Dunn test with holm correction) (Fig. 5, s7).

Figure 4. Violin plot of Faith's phylogenetic diversity. Kruskal-Wallis chi-sq (44.12), pvalue (1.4e-9) and pairwise dunn test with holm correction p-values are displayed. Differences between GVC-water and substrate-bell are not significant. Samples are colored by sample type. Means and interquartile ranges are marked with box plots for each sample type.

Formatted: Font: Bold, Not All caps

Beta diversity

Euclidean Bray-Curtis distance showed differences between medusa and non-medusa samples (pairwise.adonis, GVC vs bell: p=0.001, bell vs sub: p=0.00849, bell vs water: p=0.00122, GVC vs sub: p=0.001, GVC vs water: p=0.001, sub v water p=0.001). Variability along the primary axis was found in both GVC and bell samples, while variability along the secondary axis was found mainly in GVC samples. The location of collection had no clear impact on beta diversity.Only one pair of gastrovascular cavity sampling sites of Marathon Key (MK) and Key Largo (KL) were significantly different (pairwise Adonis, df = 1, f = 4.63, p= 0.007). The external microbiomes of Marathon Key individuals were significantly different than

those of Key West (GB) (pairwise Adonis, df = 1, f =2.28, p= 0.009), Tavernier (HHP) (pairwise Adonis, df = 1, f = 2.29, p= 0.007), and Bahia Honda Key (pairwise Adonis, df = 1, f = 5.14, p= 0.003) (full adonis comparisons in S8).

Figure 5~~4~~. ~~PCA~~ Principal component analysis of ~~Euclidean~~ Bray-Curtis distance of samples. PCA is colored by sample collection latitude from lowest (orange) to highest (purple). PCA is presented both together and faceted by sample type. Sample type is designated by shape, circle (Bell),~~-~~ triangle (GVC), square (Substrate) and cross (Water). ~~The plots show the same PCA faceted by sample type.~~Bottom plot is presented to demonstrait relative consistency of sunstrate (Sub) and water (WTR) samples on primary axes 1 and 2.

~~Faith's diversity of communities were distinct from each other (Kruskal-Wallis test, chi-squared = 44.1, df = 3, p-value = 1.4e-09). GVC communities were distinct from bell and substrate comparison groups, while bells were distinct from water and gastrovascular cavities (Dunn test with holm correction) (Fig. 5).~~

~~**Figure 5. Violin plot of Faith's diversity.** Samples are colored by sample type. Kruskal-Wallis chi-sq, pvalue and pairwise dunn test with holm correction p-values are displayed. Differences between GVC-water and substrate-bell are not significant.~~

Core microbiome

Core microbiome for the purposes of this study was evaluated based on 70% occurrence of an ASV and a relative abundance of > 0.1% of reads in all 34 gastrovascular cavity samples or external bell samples. This is in line with thresholds for other core microbiome work, however it is important to reiterate that this work was conducted only during one trip, and may be

representative of a seasonal community assemblage or one restricted to this specific collection year (57). The most abundant ASV, *Endozoicomonas* ASV1, was present in 90% of GVC samples at a relative abundance of > 0.1% of reads, ASV2 and ASV4 were present in 80% (Fig. 6). Using less stringent criteria (>70% of samples, any abundance), ASV 11, ASV 132 and ASV 6 become part of the core microbiome~~ (Fig 6)~~. These ASVs are across only three genera- *Endozoicommonas*, *Mycoplasma* and *Vibrio* (full taxonomic details of ASVs can be found in table S9~~5~~).

Figure 6. Abundance of~~–~~ *Cassiopea* core GVC microbiome across dataset. The top five ~~ASVs~~ amplicon sequence variants from the gut core microbiome across all sample types (Bell, GVC, Substrate (Sub) and Water (WTR)) organized from furthest south to furthest north sites. Note the difference in y axis maxima between bell and GVC samples (top) and substrate and water samples (bottom). See Table 1 for site details, the sites are Key West (GB), Cudjoe Key (CK), Big Pine Key (BPK), Bahia Honda Key (VP), Marathon (MK), Lower Matacumbe (LW), Tavernier (HHP), and Key Largo (KL).

In bell samples, ASV1, ASV104, and ASV86 have 73%, 70% and 70% relative abundance in samples at a 0.1% threshold. ASV6, ASV95, ASV37, ASV105, and ASV135 are present at any level in >70% of bell samples. These ASVs cover the genera *Endozoicommonas*, *Candidatus Thiobios*, *Methyloceanibacter*, *Ruegeria* and unknown genera from Desulfocapsaceae and Actibacter (Fig. S4~~7~~).

~~**Figure 7. Core microbiome prevalence in GVC and bell.** Heatmap of gastrovascular cavity (top) and bell (bottom) shaded by prevalence (0-1 scale) vs detection threshold (proportions of reads in samples) across the ASVs identified as core in each group.~~

Formatted: Font: Italic

Formatted: Indent: First line: 0.5"

In terms of abundance, internal core microbiome components are not just present, but highly dominant in *Cassiopea*'s gut. Some of these taxa are highly restricted to GVC samples (*Mycoplasma*), while others are prevalent across body surfaces (*Endozoicomonas*) (Fig. S4~~7~~).

The vast majority of ASVs were not differentially abundant between bell and GVC samples. Differential abundance (Aldex2, min effect size absolute value >[±2]) between the gastric and bell surfaces is restricted to the novel *Mycoplasma* group (ASV2), which is completely ~~lacking~~ absent in external samples (Fig 8). No features were differentially abundant between substrate and bell samples (Sfig 6~~2~~).

~~**Figure 8. Aldex2 plot of effect size of all ASVs between bell and GVC samples.** Only ASV2 has an effect >[± 2].~~

Dirichlet multinomial mixture (DMM) analysis identified specific proportions of many of these core microbiome as valuable for category differentiation. Within animal-associated samples, naïve model probability value was optimal for two communities, one GVC and one bell without subcommunities (k = 2, Laplace = 95583)(Fig. 7a). After training on separated gastrovascular and bell samples, overall model likelihood was similar and a two community structure was retained (Bell model: k=1, Laplace = 63910; GVC model: k=1, Laplace = 33200)~~False assignment rate (mismatch between predicted community and true sample type) was present in 5/60 samples~~. ASV1 amount was a core group differentiator, with fewer meaningful ASVs defining the GVC community than the bell community (Fig. 7~~9~~b, 7c). After crossvalidation, some bell and gastric samples remained grouped (Fig 7d.).

Figure 7. Dirichlet multinomial modeling model fit (a), community assignments (b), and impactful amplicon sequence variants differentiating the two community types (c+d). Dirichilet model choic graph (a) displays number of Dirichlet components (distinct assemblage types) tested vs model fit (Laplace value) in *Cassiopea* derived samples for a tested *k* value of 1-7. Best fit is at 2 components, or *k* = 2. Assignment of samples to one of two communities (b) is shown separated by location and sample type with a “0” value indicative of the bell community type and a “1” value indicative of the GVC community type. Boxplots display means for each sampling group. Points are displayed with a small jitter (0.1) for visibility. ASV list (b) displays the top 0.5% most impactful drivers of body cavity prediction in GVC (c) and bell (d) Dirichlet multinomial models ordered from largest to smallest effect size.

~~**Figure 9. Most impactful ASVs for Dirichlet multinomial modeling.** Top 0.5% most impactful drivers of body cavity prediction in GVC (top) and bell (bottom) Dirichlet multinomial models ordered from largest to smallest effect size.~~

~~Despite DMM identifying all gastric samples as belonging to the same community, relative~~Mean proportions ~~of those community members differed dramatically, varying substantially in abundance of~~of *Endozoicommonas*, *Mycoplasma* and *Vibrio* varied across locations. Variation ~~within~~ of bell samples primarily occurred with proportion of *Endozoicommonas*. The communities across sites for gastrovascular samples varied in degree of community balance, resolving into primarily Mycoplasmatales-dominated and Pseudomonadales-dominated arrangements, with limited relationships between primary ecological vectors (pH, salinity, temp) and Mycoplasmatales, Vibrionales or Pseudomonadales (Fig. 8, Sfig. 7-9). ~~, but was not reliably distinguishable from substrate samples by DMM (Fig. 10).~~

Figure ~~8~~10. Star plots of community composition across sites. Star plots for internal (GVC), external (Bell) mucus swabs, water (WTR) and substrate (Sub) sample averages at each site

Formatted: Font: Not Bold

Formatted: Font: Not Bold

Formatted: Font: Not Bold

Formatted: Font: Not Bold

Formatted: Indent: First line: 0"

Formatted: Font: Not Italic

Formatted: Font: Not Italic

separated by order (three most prominent orders and "other"). Each spoke of the star plot ranges from 0% (center) to 100%. **Internal** group, starting from the 3' position and going counterclockwise: Pseudomonadales, Mycoplasmatales, Enterobacteriales and "Other". **External** group spokes (going counterclockwise): Pseudomonadales, Rhodobacterales, Chromatiales, and "Other". **Water** group spokes (going counterclockwise): Pseudomonadales, Rhodobacterales, Flavobacteriales, and "Other". **Substrate** group spokes (going counterclockwise): Rhodobacterales, Flavobacteriales, Desulfobacterales, and "Other". Each site's measured environmental conditions (salinity, water surface temperature, and pH) are presented below star plots, colored by intensity (low to high represented by pale to dark) . Sites are mapped onto their location within the Florida Keys.

Vibrio

ASVs identical to *Vibrio coralliilyticus* were found across *Cassiopea* gastric samples, with abundance of up to 10% of reads within the sample. This far surpasses rates in the surrounding water and substrate samples (ASV11 averages: Bell: 0.05%, WTR: 0.03%, Sub: 0%, GVC: 2.09%) (Fig. ~~11~~9).

Figure ~~11~~9. Percent of reads (0%-10%) assigned to *V. coralliilyticus* ~~ASVs~~ amplicon sequence variants in bell, GVC, substrate and water samples averaged by site. Organized by body cavity**.**

~~Host medusa species identity~~

~~The mitochondrial haplotype of the non-native *Cassiopea*, *Cassiopea andromeda*, a cryptic sister species to *C. xamachana*, was found in four of the 34 medusae sampled. The four *C. andromeda* mitotype individuals included were from the Key Largo (1), Cudjoe Key (2), and Key West (1) sites. While these were excluded from the primary analysis, *Cassiopea andromeda* showed no divergence from the trends of the *C. xamachana* simultaneously collected (Fig. 12).~~

Figure 12. PCA of Euclidean distance between *Cassiopea* species. *Cassiopea andromeda* (CA: grey) and *Cassiopea xamachana* (CX: black) samples at the sites were they cooccurred (Cudjoe Key, Garrison Bight and Key Largo).

Discussion

Cassiopea medusae show consistent differences in the microbial communities present on their bell and gastric surfaces. Gastrovascular cavity samples have lower diversity than the sediment, external surfaces, and host a novel *Mycoplasma* lineage. This low diversity is consistent with results for multiple other scyphozoans— including *Cotylorhiza tuberculata* (Macri, 1778) (27). *Cassiopea* also appears to have some variation in community composition between sites, a feature consistent with results from other species, such as *Aurelia*, *Mastigias*, and *Tipedalia* (22,26).

Formatted: Font: Not Italic

A bifurcated outer and inner microbiome

Across all sites, the gastrovascular cavities of *Cassiopea* medusae showed a core microbiome comprised of taxa previously found in photosymbiotic cnidarians. The external microbial community of *Cassiopea* from the Florida Keys adheres fairly closely to the epibenthic microbial community at each site, with the addition of *Endozoicomonas*. ASV1 *Endozoicomonas* cf. *atrinae* may be a primary mucosal component and extend to the exterior of the body through mucus exchange, as it is less prevalent on the bell surface than internal for most individuals. Alternitavely, ASV1 may be a common mucosal resident with no function for the host.

The abundant *Mycoplasma* and *Vibrio* cf. *coralliilyticus* from the gastric cavity of *Cassiopea* were not found in any external mucus samples and found at far lower abundances in external mucosal samples respectively.

The high diversity of the *Cassiopea* bell microbiome found in this study may be unique to *Cassiopea*. Previous work has reported in the low-diversity mucosa, oral arms and umbrella of a previously studied pelagic rhizostome, *Rhizostoma pulmo,* a likely result of the constant contact of *Cassiopea* with the epibenthos (28). The limited overlap between diverse bell communities and low diversity gut communities in *Cassiopea* is notable and consistent with the significant differences in body compartment microbiome of other scyphozoans and anthozoans (23,26,58). While the low~~lack of~~ diversity in scyphozoan microbiomes may seem distinct from many described stony coral microbial communities, these stony coral microbial profiles are often produced using whole body sampling. Lower diversity gastrovascular cavities with distinct community composition may also be a feature of stony corals (59–61).

Few m~~M~~icrobial communities across sites within this study were ~~not~~ significantly different from each other, as gastrovascular cavity components remained dominated by three taxa throughout the Keys.~~–~~ This microbiome bears limited resemblance to those sampled in common garden experiments and no resemblance to lab-held lines, demonstrating that “core” here should not be misconstrued as obligate (21,62). The lack of obligate bacterial taxa in the *Cassiopea* gastrovascular cavity is also consistent with other microbial hosts, notably the anemone *Exaiptasia diaphana,* another successful model cnidarian (1). While *Cassiopea* require their algal symbiont, bacterial partners may be transient, and bacteria may have far less significance to host health for *Cassiopea* than for specialized obligate hosts (e.g., leafhoppers) (63–66). Without a better understanding of specific metabolic function of different bacterial

associates, microbiome importance cannot be determined. We identified *Endozoicomonas*, *Mycoplasma*, and *Vibrio* as the three most common bacterial genera found within the gastrovascular cavity of *Cassiopea*. These are all commonly associated with coral microbial communities (8,9,67). Below, we provide information on these common bacterial components of cnidarian holobionts.

Endozoicomonas functions in microbiomes

The large proportion of *Endozoicomonas* in internal and external samples fits within a broader framework of both *Endozoicomonas* and known cnidarian-associated microbial communities; *Endozoicomonas* strains are present in a variety of invertebrates, including many corals, sponges, sea anemones, scyphozoans and cubozoans (9,67–70). Some hosts house multiple distinct *Endozoicomonas* genotypes, suggesting that different *Endozoicomonas* clades may provide different metabolic services to a host (e.g., Vitamin B12 metabolism) (71). *Endozoicomonas* is more commonly identified in healthy photosymbiotic scleractinian corals than bleached and diseased ones (72). For these corals, it may play a role in a variety of fundamental *Symbiodinium*–host interactions, including nutrient transport and symbiont cell breakdown (71). Notably, *Endozoicomonas* is not a consistent marker of microbiome health, its disappearance and reappearance without apparent host health declines has been identified in octocorals (2,70). In some coral, members of *Endozoicomonas* are found in close proximity to *Symbiodinium*, it is unknown whether this feature is shared with *Cassiopea* (71). As few cnidarians previously studied have gastric cavities large enough to be readily sampled, the finding of *Endozoicomonas* across cavities suggests that this group is not specialized in surface defense.

Formatted: Font: Italic

Within scyphozoa, *Endozoicomonas* is abundant in the microbial community of the rhizostome scyphozoan *Mastigias* (22). While *Mastigias*, like *Cassiopea*, host zooxanthellae from the family *Symbiodiniaceae*,– not all symbiotic rhizostomes harbor the microbe, and not all taxa with the microbe are symbiotic (22,27). *Endozoicomonas* may be common across medusae, but the difficulty of culturing some strains may have excluded them from the results of past culture-based analyses (70,71). Greater clarity is needed on the function of *Endozoicomonas* for these medusae and other taxa.

Mycoplasmataceae: a poorly understood cnidarian microbiome resident

Mycoplasma, like *Endozoicomonas*, is present in studied coral and scyphozoan microbiomes (9,24,25,27). While intracellular parasitism is common for members of Mycoplasmataceae, those associated with cnidarians may be located extracellularly and exist as opportunistic commensals (2,73). *Mycoplasma* sp. is noted as a potential endosymbiont in lab-raised *Aurelia* polyps, while adults from many jellyfish populations are known to have them (24,26,29). The high divergence between this *Mycoplasma* and others of the genus (closest sequence identity on NCBI at 89%: Accn. No. NR_115937) suggests that this may be a hitherto unknown group. Whether this *Mycoplasma* represents an endosymbiont, a result of prey capture, or a parasite, its exclusivity to the gastrovascular tract in *Cassiopea* sampled within the Keys is notable, as other studies have not demonstrated gastrovascular cavity localization (26,29).

Vibrionaceae

Vibrionaceae is both a core part of coral microbiomes (9,74) and known to facilitate metamorphosis within *Cassiopea* (75,76), however, it is a highly diverse family. While *Vibrio* spp. are found in gut communities of presumed healthy scyphozoans (24,25) (Daley et al. 2016, Peng et al. 2021), they can also be associated with senescence and disease(77,78) (Kramar et al., 2019; Tinta et al., 2012). For *Cassiopea* specifically, an increase in *Vibrio* has previously been associated with an aposymbiotic or stressed state (62). Given the variation in Vibrionaceae species and strains, family-wide statements on the beneficial or harmful nature of Vibrionaceae within *Cassiopea* gastrovascular cavities should not be made.

Vibrio coralliilyticus

In this dataset we found ample signal of a 16S V3-V4 with 100% sequence match to multiple *Vibrio* strains, including *V. coralliilyticus* and the oyster pathogen, *V. tubiashii*. As *Vibrio* species are highly varied, the sequence match does not mean assured pathogenicity towards any taxa (79–81). The apparent health of the medusae from whom these samples were collected suggest a either a lack of impact of this specific group on the host or a host-microbe facultative interaction between this *Vibrio* and *Cassiopea.* While there is data on *Cassiopea* microbiome antimicrobial isolates and *Cassiopea* mortality when faced with *Serratia marcescens* infection, we lack adequate details on the *Vibrio* – *Cassiopea* system to determine whether this represents a lowlying widespread infection or a mutualism (82,83).

Drivers of external microbiome

While the external microbiome was not distinct from the sediment around it, it is worth discussing the major groups more common externally than internally. While the core bell microbiome represents a substrate-like community, the bell core microbiome includes

representatives from families and genera with a variety of potential metabolic capacities. The most plentiful of these ASVs was *Candidatus Thiobios*, known as a sulfur-oxidizing epibiont of the *Zoothamnium niveum* (84,85). As this bacteria can be found in the Florida Keys, their appearance on *Cassiopea* may be circumstantial, but is consistent across medusae (84). Isolates from *Methyloceanibacter* have been demonstrated to be methane oxidizers from marine environments (86). These may be sediment associated or may recruit to the host to utilize algal products. The potential sulfate reducers within Desulfocapaceae may similarly recruit to the bell and its access to sediment pore waters (87). While *Pseudomonas* cf. *stuzeri* was not as high abundance as the other metabolizers listed above, it may have a role in denitrification or coral health for some species (88–90)

In addition to microbes with known metabolic functions that align with the *Cassiopea* holobiont, the genus *Ruegeria* includes many marine species and has been identified in the microbiomes of photsymbiotic and nonphotosymbiotic cnidarians alike (9,77,91). As Dirichlet modeling for bell microbiomes gave similar weight to many bacteria, the relative impact of all of these bacterial epibionts on the host is unclear.

Laboratory cultures

Cassiopea are primarily used for in-lab studies on symbiosis (12,14,92). Some of these use wild-caught individuals, and others use lab-bred cultures, many of which have been held for years. The lab-raised *Cassiopea* used in Rothig et al. 2021 appear to have a completely different microbiome than the wild individuals caught here (21). This potential discrepancy should be considered when using wild-caught or captive-bred individuals, as captive-bred symbiosis and tolerance levels may differ from medusae in the wild. These discrepancies in microbiome are

found in other model cnidarians, such as *Exaiptasia* (1). The information on wild *Cassiopea* microbial communities from the Florida Keys presented here may allow researchers working with lab-raised individuals to better contextualize the taxa that they find in cultured medusae.

Conclusion

The microbiome of *Cassiopea* within the Florida Keys demonstrates strong body compartment discrimination and includes a central group of ASVs overrepresented in the gut and bell mucus. All of these families, Pseudomonadales, Vibrionaceae, and Mycoplasmataceae, are found in other medusae and anthozoans. Despite this overrepresentation of some microbial taxa within the gut, the *Cassiopea* bell is highly diverse, and overlaps with the communities of the water and substrate in and on which it resides. The consistency of *Cassiopea* microbial communities across the Florida Keys demonstrates that specific taxa are strongly associated with *Cassiopea* and that this can extend across many sites with diverse physical conditions. This work provides a greater understanding of the wild microbiome of the common lab organism *Cassiopea*.

Acknowledgements

The present work was part of KMM's PhD dissertation. Thank you to Dr. David Retchless for input on figure design. We thank the Florida Fish and Wildlife Conservation Commission for their help and guidance.

References

1. Brown T, Otero C, Grajales A, Rodriguez E, Rodriguez-Lanetty M. Worldwide

exploration of the microbiome harbored by the cnidarian model, Exaiptasia pallida (Agassiz in Verrill, 1864) indicates a lack of bacterial association specificity at a lower taxonomic rank. PeerJ. 2017;2017(5).

2. Van De Water JAJM, Allemand D, Ferrier-Pagès C. Host-microbe interactions in octocoral holobionts - recent advances and perspectives. Microbiome. 2018;6(1):1–28.

3. Meyer JL, Castellanos-Gell J, Aeby GS, Häse CC, Ushijima B, Paul VJ. Microbial Community Shifts Associated With the Ongoing Stony Coral Tissue Loss Disease Outbreak on the Florida Reef Tract. Front Microbiol. 2019;10(September):1–12.

4. Djeghri N, Pondaven P, Stibor H, Dawson MN. Review of the diversity, traits, and ecology of zooxanthellate jellyfishes. Mar Biol [Internet]. 2019;166(11):1–19. Available from: https://doi.org/10.1007/s00227-019-3581-6

5. Baldassarre L, Ying H, Reitzel AM, Franzenburg S, Fraune S. Microbiota mediated plasticity promotes thermal adaptation in the sea anemone Nematostella vectensis. Nat Commun. 2022;13(1).

6. Ushijima B, Gunasekera SP, Meyer JL, Tittl J, Pitts KA, Thompson S, et al. Chemical and genomic characterization of a potential probiotic treatment for stony coral tissue loss disease. Commun Biol. 2023;6(1).

7. Connelly MT, McRae CJ, Liu PJ, Martin CE, Traylor-Knowles N. Antibiotics Alter Pocillopora Coral-Symbiodiniaceae-Bacteria Interactions and Cause Microbial Dysbiosis During Heat Stress. Front Mar Sci. 2022;8(January):1–18.

8. Pollock FJ, McMinds R, Smith S, Bourne DG, Willis BL, Medina M, et al. Coral-

associated bacteria demonstrate phylosymbiosis and cophylogeny. Nat Commun [Internet]. 2018;9(1):1–13. Available from: http://dx.doi.org/10.1038/s41467-018-07275-x

9. Huggett MJ, Apprill A. Coral microbiome database : Integration of sequences reveals high diversity and relatedness of coral- associated microbes. Environ Microbiol Rep. 2019 Jun 1;11(3):372–85.

10. Puntin G, Sweet M, Fraune S, Medina M, Sharp K, Weis VM, et al. Harnessing the Power of Model Organisms To Unravel Microbial Functions in the Coral Holobiont. Microbiol Mol Biol Rev [Internet]. 2022 Oct 26 [cited 2023 Feb 24];86(4). Available from: https://journals.asm.org/doi/10.1128/mmbr.00053-22

11. Ohdera AH, Abrams MJ, Ames CL, Baker DM, Suescún-Bolívar LP, Collins AG, et al. Upside-Down but Headed in the Right Direction: Review of the Highly Versatile Cassiopea xamachana System. Front Ecol Evol. 2018 Apr 9;6:1–15.

12. Mammone M, Ferrier-Pages C, Lavorano S, Rizzo L, Piraino S, Rossi S. High photosynthetic plasticity may reinforce invasiveness of upside-down zooxanthellate jellyfish in Mediterranean coastal waters. PLoS One [Internet]. 2021 Mar 1 [cited 2021 Jun 30];16(3 March):e0248814. Available from: https://doi.org/10.1371/journal.pone.0248814

13. Béziat P, Kunzmann A. Under pressure: Cassiopea andromeda jellyfish exposed to increasing water temperature or lead, cadmium and anthropogenic gadolinium contamination. Mar Biol Res [Internet]. 2022;18(1–2):48–63. Available from: https://doi.org/10.1080/17451000.2022.2066132

14. McGill CJ, Pomory CM. Effects of bleaching and nutrient supplementation on wet weight

in the jellyfish Cassiopea xamachana (Bigelow) (Cnidaria: Scyphozoa). Mar Freshw Behav Physiol. 2008;41(3):179–89.

15. Ames CL, Klompen AML, Badhiwala K, Muffett K, Reft AJ, Kumar M, et al. Cassiosomes are stinging-cell structures in the mucus of the upside-down jellyfish Cassiopea xamachana. Commun Biol [Internet]. 2020;3(1):1–15. Available from: http://dx.doi.org/10.1038/s42003-020-0777-8

16. Bakshani CR, Morales-Garcia AL, Althaus M, Wilcox MD, Pearson JP, Bythell JC, et al. Evolutionary conservation of the antimicrobial function of mucus: A first defence against infection. npj Biofilms Microbiomes [Internet]. 2018;4(1):1–12. Available from: http://dx.doi.org/10.1038/s41522-018-0057-2

17. Klein SG, Pitt KA, Lucas CH, Hung S, Schmidt-roach S, Aranda M, et al. Night-Time Temperature Reprieves Enhance the Thermal Tolerance of a Symbiotic Cnidarian. 2019;6(July):1–16.

18. Fitt WK, Costley K. The role of temperature in survival of the polyp stage of the tropical rhizostome jellyfish Cassiopea xamachana. 1998;222:79–91.

19. Al-jbour SM, Zimmer M, Kunzmann A. Cellular respiration, oxygen consumption, and trade-offs of the jellyfish Cassiopea sp. in response to temperature change. J Sea Res [Internet]. 2017;128(January):92–7. Available from: https://doi.org/10.1016/j.seares.2017.08.006

20. Lee MD, Kling JD, Araya R, Ceh J. Jellyfish Life Stages Shape Associated Microbial Communities, While a Core Microbiome Is Maintained Across All. Front Microbiol. 2018 Jul 12;0(JUL):1534.

21. Röthig T, Puntin G, Wong JCY, Burian A, McLeod W, Baker DM. Holobiont nitrogen control and its potential for eutrophication resistance in an obligate photosymbiotic jellyfish. Microbiome. 2021;9(1):1–16.

22. Cleary DFR, Becking LE, Polónia ARM, Freitas RM, Gomes NCM. Jellyfish-associated bacterial communities and bacterioplankton in Indonesian Marine lakes. FEMS Microbiol Ecol. 2016;92(5):1–14.

23. Hao W, Gerdts G, Holst S, Wichels A. Bacterial communities associated with scyphomedusae at Helgoland Roads. Mar Biodivers. 2019;49(3):1489–503.

24. Peng S, Hao W, Li Y, Wang L, Sun T, Zhao J, et al. Bacterial Communities Associated With Four Blooming Scyphozoan Jellyfish: Potential Species-Specific Consequences for Marine Organisms and Humans Health. Front Microbiol. 2021 May 5;12(May):1–16.

25. Daley MC, Urban-Rich J, Moisander PH. Bacterial associations with the hydromedusa Nemopsis bachei and scyphomedusa Aurelia aurita from the North Atlantic Ocean. Mar Biol Res. 2016;12(10):1088–100.

26. Weiland-Bräuer N, Neulinger SC, Pinnow N, Künzel S, Baines JF, Schmitz RA. Composition of bacterial communities associated with Aurelia aurita changes with compartment, life stage, and population. Appl Environ Microbiol. 2015;81(17):6038–52.

27. Viver T, Orellana LH, Hatt JK, Urdiain M, Díaz S, Richter M, et al. The low diverse gastric microbiome of the jellyfish Cotylorhiza tuberculata is dominated by four novel taxa. Environ Microbiol. 2017;19(8):3039–58.

28. Basso L, Rizzo L, Marzano M, Intranuovo M, Fosso B, Pesole G, et al. Jellyfish summer

outbreaks as bacterial vectors and potential hazards for marine animals and humans health? The case of Rhizostoma pulmo (Scyphozoa, Cnidaria). Sci Total Environ [Internet]. 2019;692:305–18. Available from: https://doi.org/10.1016/j.scitotenv.2019.07.155

29. Stabili L, Rizzo L, Basso L, Marzano M, Fosso B, Pesole G, et al. The microbial community associated with Rhizostoma pulmo: Ecological significance and potential consequences for marine organisms and human health. Mar Drugs. 2020;18(9).

30. Delannoy CMJ, Houghton JDR, Fleming NEC, Ferguson HW. Mauve Stingers (Pelagia noctiluca) as carriers of the bacterial fish pathogen Tenacibaculum maritimum. Aquaculture [Internet]. 2011;311(1–4):255–7. Available from: http://dx.doi.org/10.1016/j.aquaculture.2010.11.033

31. Muffett K, Miglietta MP. Demystifying Cassiopea species identity in the Florida Keys: Cassiopea xamachana and Cassiopea andromeda coexist in shallow waters. PLoS One [Internet]. 2023;18(3):e0283441. Available from: http://dx.doi.org/10.1371/journal.pone.0283441

32. Laramie MB, Pilliod DS, Goldberg CS, Strickler KM. Environmental DNA Sampling Protocol — Filtering Water to Capture DNA from Aquatic Organisms. In: US Geological Survey Techniques and Methods, book 2. Reston, VA: U.S. Geological Survey; 2015. p. 1–15.

33. Pavlovska M, Prekrasna I, Parnikoza I, Dykyi E. Soil sample preservation strategy affects the microbial community structure. Microbes Environ. 2021;36(1):1–7.

34. Callahan BJ, McMurdie PJ, Rosen MJ, Han AW, Johnson AJA, Holmes SP. DADA2:

High-resolution sample inference from Illumina amplicon data. Nat Methods. 2016;13(7):581–3.

35. R-Core-Team (Foundation for Statistical Computing). R: A Language and Environment for Statistical Computing [Internet]. 2022. Available from: https://www.r-project.org/

36. Quast C, Pruesse E, Yilmaz P, Gerken J, Schweer T, Yarza P, et al. The SILVA ribosomal RNA gene database project: Improved data processing and web-based tools. Nucleic Acids Res. 2013;41(D1):590–6.

37. McMurdie PJ, Holmes S. Phyloseq: An R Package for Reproducible Interactive Analysis and Graphics of Microbiome Census Data. PLoS One. 2013;8(4).

38. Davis NM, Proctor DiM, Holmes SP, Relman DA, Callahan BJ. Simple statistical identification and removal of contaminant sequences in marker-gene and metagenomics data. Microbiome. 2018;6(1):1–14.

39. Oksanen J, Simpson G, Blanchet F, Kindt R, Legendre P, Minchin P, et al. _vegan: Community Ecology Package_. 2024.

40. Paradis E, Schliep K. ape 5 . 0 : an environment for modern phylogenetics and evolutionary analyses in R. 2019;35(July 2018):526–8.

41. Mikryukov V. metagMisc: Miscellaneous functions for metagenomic analysis [Internet]. Available from: https://github.com/vmikk/metagMisc

42. Taudiere A. MiscMetabar: Miscellaneous Functions for Metabarcoding Analysis [Internet]. 2024. Available from: https://cran.r-project.org/package=MiscMetabar

43. Tsirogiannis C, Sandel B. PhyloMeasures: Fast and Exact Algorithms for Computing

Phylogenetic Biodiversity Measures. 2017.

44. Schliep KP. phangorn : phylogenetic analysis in R. 2011;27(4):592–3.

45. Battaglia T. btools: A suite of R function for all types of microbial diversity analyses. 2025.

46. Patil I. Visualizations with statistical details: The “ggstatsplot” approach. J Open Source Softw [Internet]. 2021;6(61)(3167). Available from: doi:10.21105/joss.03167

47. Martinez Arbizu P. pairwiseAdonis: Pairwise Multilevel Comparison using Adonis [Internet]. 2017. Available from: https://github.com/pmartinezarbizu/pairwiseAdonis

48. Kassambara A. rstatix: Pipe-Friendly Framework for Basic Statistical Tests [Internet]. 2023. Available from: https://cran.r-project.org/package=rstatix

49. McLaren M. speedyseq: Faster implementations of phyloseq functions [Internet]. 2025. Available from: https://github.com/mikemc/speedyseq

50. Pebesma E, Bivand R. Spatial Data Science: With applications in R [Internet]. London: Chapman and Hall/CRC; 2023. 352 p. Available from: https://r-spatial.org/book/

51. Macvittie S, Doroodian S, Alberto A, Sogin M, Macvittie S, Doroodian S, et al. Exaiptasia diaphana , following antibiotic exposure. 2024;9(6).

52. Fernandes AD, Reid JNS, Macklaim JM, Mcmurrough TA, Edgell DR, Gloor GB. Unifying the analysis of high-throughput sequencing datasets : characterizing RNA-seq , 16S rRNA gene sequencing and selective growth experiments by compositional data analysis. 2014;1–13.

53. Holmes I, Harris K, Quince C. Dirichlet Multinomial Mixtures : Generative Models for

Microbial Metagenomics. 2012;7(2).

54. Morgan M. DirichletMultinomial: Dirichlet-Multinomial Mixture Model Machine Learning for Microbiome Data [Internet]. 2024. Available from: https://doi.org/10.18129/B9.bioc.DirichletMultinomial

55. Lahti L, Shetty S. microbiome R package. 2019.

56. Barnett D, Arts I, Penders J. microViz: an R package for microbiome data visualization and statistics. J Open Source Softw. 2021;6(63):3201.

57. Neu AT. Defining and quantifying the core microbiome : Challenges and prospects. 2021;118(51):1–10.

58. Bonacolta AM, Connelly MT, Rosales SM, del Campo J, Traylor-knowles N. The starlet sea anemone , Nematostella vectensis , possesses body region-specific bacterial associations with spirochetes dominating the capitulum. FEMS Microbiol Lett. 2021;(August 2020):1–9.

59. van Oppen MJH, Blackall LL. Coral microbiome dynamics, functions and design in a changing world. Nat Rev Microbiol [Internet]. 2019;17(9):557–67. Available from: http://dx.doi.org/10.1038/s41579-019-0223-4

60. Agostini S, Yoshimi S, Higuchi T, Casareto BE. Biological and chemical characteristics of the coral gastric cavity. Coral Reefs. 2011;(March).

61. Bollati E, Hughes DJ, Suggett DJ, Raina J-B, Kühl M. Microscale sampling of the coral gastric cavity reveals a gut-like microbial community. bioRxiv [Internet]. 2024;2024.05.20.594925. Available from:

https://www.biorxiv.org/content/10.1101/2024.05.20.594925v1%0Ahttps://www.biorxiv.org/content/10.1101/2024.05.20.594925v1.abstract

62. Carabantes N, Cerqueda-García D, García-Maldonado JQ, Thomé PE. Changes in the Bacterial Community Associated With Experimental Symbiont Loss in the Mucus Layer of Cassiopea xamachana Jellyfish. Front Mar Sci. 2022;9(May):1–13.

63. Wu W, Lei J-N, Mao Q, Tian Y-Z, Shan H-W, Chen J-P. Distribution, Vertical Transmission, and Cooperative Mechanisms of Obligate Symbiotic Bacteria in the Leafhopper Maiestas dorsalis (Hemiptera, Cicadellidea). Insects. 2023;1–13.

64. Sharp KH, Pratte ZA, Kerwin AH, Rotjan RD, Stewart FJ. Season, but not symbiont state, drives microbiome structure in the temperate coral Astrangia poculata. Microbiome. 2017;5(1):120.

65. Hammer TJ, Sanders JG, Fierer N. Not all animals need a microbiome. FEMS Microbiol Lett. 2019;366(10):1–11.

66. Riviere M La, Roumagnac M, Garrabou J, Bally M. Transient Shifts in Bacterial Communities Associated with the Temperate Gorgonian Paramuricea clavata in the Northwestern Mediterranean Sea. PLoS One. 2013;8(2).

67. Neave MJ, Apprill A, Ferrier-Pagès C, Voolstra CR. Diversity and function of prevalent symbiotic marine bacteria in the genus Endozoicomonas. Appl Microbiol Biotechnol [Internet]. 2016;100(19):8315–24. Available from: http://dx.doi.org/10.1007/s00253-016-7777-0

68. Tinta T, Kogovšek T, Klun K, Malej A, Herndl GJ, Turk V. Jellyfish-associated

microbiome in the marine environment: Exploring its biotechnological potential. Mar Drugs. 2019;17(2):1–33.

69. Maher RL, Schmeltzer ER, Meiling S, McMinds R, Ezzat L, Shantz AA, et al. Coral Microbiomes Demonstrate Flexibility and Resilience Through a Reduction in Community Diversity Following a Thermal Stress Event. Front Ecol Evol. 2020;8(October):1–15.

70. Pogoreutz C, Oakley CA, Rädecker N, Cárdenas A, Perna G, Xiang N, et al. Coral holobiont cues prime Endozoicomonas for a symbiotic lifestyle. ISME J. 2022;16(8):1883–95.

71. Neave MJ, Michell CT, Apprill A, Voolstra CR. Endozoicomonas genomes reveal functional adaptation and plasticity in bacterial strains symbiotically associated with diverse marine hosts. Sci Rep. 2017;7(December 2016):1–12.

72. McDevitt-Irwin JM, Baum JK, Garren M, Vega Thurber RL. Responses of coral-associated bacterial communities to local and global stressors. Front Mar Sci. 2017;4(AUG):1–16.

73. Neulinger SC, Gärtner A, Järnegren J, Ludvigsen M, Lochte K, Dullo WC. Tissue-associated “Candidatus mycoplasma corallicola” and filamentous bacteria on the cold-water coral Lophelia pertusa (Scleractinia). Appl Environ Microbiol. 2009;75(5):1437–44.

74. Sweet MJ, Bulling MT. On the importance of the microbiome and pathobiome in coral health and disease. Front Mar Sci. 2017;4(January):1–11.

75. Hofmann DK, Neumann R, Henne K. Strobilation, budding and initiation of scyphistoma morphogenesis in the rhizostome Cassiopea andromeda (Cnidaria: Scyphozoa). Mar Biol.

1978;47(2):161–76.

76. Neumann R. Bacterial Induction of Settlement and Metamorphosis in the Planula Larvae of Cassiopea andromeda (Cnidaria: Scyphozoa, Rhizostomeae). Vol. 1. 1979.

77. Kramar MK, Tinta T, Lučić D, Malej A, Turk V. Bacteria associated with moon jellyfish during bloom and post-bloom periods in the Gulf of Trieste (northern Adriatic). PLoS One. 2019;14(1):1–21.

78. Tinta T, Kogovsek T, Malej A, Turk V. Jellyfish Modulate Bacterial Dynamic and Community Structure. PLoS One. 2012;7(4):1–10.

79. Pollock FJ, Wilson B, Johnson WR, Morris PJ, Willis BL, Bourne DG. Phylogeny of the coral pathogen Vibrio coralliilyticus. Environ Microbiol Rep. 2010;2(1):172–8.

80. Ben-Haim Y, Thompson FL, Thompson CC, Cnockaert MC, Hoste B, Swings J, et al. Vibrio coralliilyticus sp. nov., a temperature-dependent pathogen of the coral Pocillopora damicornis. Int J Syst Evol Microbiol. 2003;53(1):309–15.

81. Richards GP, Watson MA, Needleman DS, Church KM, Häse CC. Mortalities of Eastern And Pacific oyster larvae caused by the pathogens Vibrio coralliilyticus and Vibrio tubiashii. Appl Environ Microbiol. 2015;81(1):292–7.

82. Emery MA, Beavers KM, Van Buren EW, Batiste R, Dimos B, Pellegrino MW, et al. Trade-off between photosymbiosis and innate immunity influences cnidarian’s response to pathogenic bacteria. Proc R Soc B Biol Sci. 2024;291(2032):20240428.

83. Thomé PE, Rivera-Ortega J. Specific antibacterial activity against potential pathogens and re-straining of larvae settlement from a pigmented Pseudoalteromonas strain isolated from

the jellyfish Cassiopea xamachana. Int Aquat Res. 2022;14(2):95–105.

84. Bright M, Espada-hinojosa S, Lagkouvardos I, Volland J, Hole W. The giant ciliate Zoothamnium niveum and its thiotrophic epibiont Candidatus Thiobios zoothamnicoli : a model system to study interspecies cooperation. 2014;5(April):1–13.

85. Rinke C, Schmitz-esser S, Stoecker K, Nussbaumer AD, Vanura K, Wagner M, et al. “ Candidatus Thiobios zoothamnicoli ,” an Ectosymbiotic Bacterium Covering the Giant Marine Ciliate Zoothamnium niveum. 2021;72(3):2014–21.

86. Vekeman B. Methanotrophic microbiomes from North Sea sediment.

87. Song J, Hwang J, Kang I, Cho JC. OPEN A sulfate - reducing bacterial genus , Desulfosediminicola gen . nov ., comprising two novel species cultivated from tidal - flat sediments. Sci Rep [Internet]. 2021;1–13. Available from: https://doi.org/10.1038/s41598-021-99469-5

88. Engelen AH, Aires T, Vermeij MJA, Herndl GJ, Serrão EA, Frade PR. Host differentiation and compartmentalization of microbial communities in the azooxanthellate cupcorals Tubastrea coccinea and Rhizopsammia goesi in the Caribbean. Front Mar Sci. 2018;9(NOV):1–9.

89. Meirelles LA, Newman DK. Both toxic and beneficial effects of pyocyanin contribute to the lifecycle of Pseudomonas aeruginosa. Mol Microbiol. 2018;110(6):995–1010.

90. Carlson CA, Ingraham JL. Comparison of Denitrification by Pseudomonas stutzeri , Pseudomonas aeruginosa , and Paracoccus denitrificans. 1983;45(4):1247–53.

91. Huo Y, Xu X, Li X, Liu C, Cui H, Wang C, et al. Ruegeria marina sp . nov ., isolated from

Marine Sediment. 2011;347–50.

92. Klein SG, Pitt KA, Carroll AR. Reduced salinity increases susceptibility of zooxanthellate jellyfish to herbicide toxicity during a simulated rainfall event. Environ Pollut [Internet]. 2016;209:79–86. Available from: http://dx.doi.org/10.1016/j.envpol.2015.11.012

93. Hyun DW, Shin NR, Kim MS, Oh SJ, Kim PS, Whon TW, et al. Endozoicomonas atrinae sp. nov., isolated from the intestine of a comb pen shell Atrina pectinata. Int J Syst Evol Microbiol. 2014;64(PART 7):2312–8.

94. Sunagawa S, Woodley CM, Medina M. Threatened corals provide underexplored microbial habitats. PLoS One. 2010;5(3):1–7.

Supplementary Information

Supplement A1: Full length 16S rRNA gene identities

Three samples were sent to CD Genomics for full length 16S on PacBio Sequel using universal 16S primers (F: AGRGTTTGATYNTGGCTCAG; R:TASGGHTACCTTGTTASGACTT). Samples sent for full length 16S rRNA sequencing included gastrovascular cavity swab extractions from medusae Marathon Key GVC 30 and Key West GVC 96 and GVC 120. As the usable reads returned from this were low (597-12520 per sample), these full-length identities are not discussed in the main text. For further details, please refer to the appendix (A1).

Full length 16S rRNA gene sequences (BioProject: PRJNA1020446) were run on mothur v1.48.0 with deviation from the MiSeq SOP on trim length. ASV cutoff was set to five

differences, and ASV representative sequences were compared against the NCBI GenBank (Sept 24, 2023) database using BLAST to find nearest matches by e-value.

In order to identify core microbial groups to a lower taxonomic level, three internal medusa gastrovascular swabs (medusae Kmuffet_96, Kmuffet_120 and Kmuffet_30) from Marathon Key (MK) and Key West (GB) were sequence for full length 16S rRNA gene (see S1 for location of samples). As the primers were not chloroplast exclusionary, the Key West sites produced few useable sequences (Kmuffet_96: 597 and Kmuffet_120: 1097). The Marathon Key medusa (Kmuffet_30), likely bleached at time of collection, returned 12,520 non-chloroplast sequences. The Mycoplasma-like full length 16S rRNA gene sequence (Genbank Acc. No. OR592270) from Marathon Key and Key West has no closely related sequences currently identified (greatest identity 88.43% to sequence HQ393440). The *Endozoicomonas* strain from both Marathon Key and Key West (Genbank Acc. No. OR592271) has 99.93% identity to *Endozoicomonas atrinae* strain WP70(T) collected from the intestine of South Korean *Atrina pectinata* (Accession NR_134024)(93) and 99.80% identity (<5 nucleotide differences) with isolates collected from *Gorgonia ventalina* (GenBank Acc. No. GU118345) in Bocas del Toro, Panama (94). *Endozoicomanas* and *Mycoplasma*-like isolates were the only two groups to have ASVs with over 100 sequences.

Supplementary ~~1~~ Tables: (in Supplementary Excel Workbook)

Supplementary Table S1: Roster of collected medusae

Supplementary Table S2: Read processing tracking table

Supplementary Table S3: Representative sequences of all ASVs from the dataset

Supplementary Table S4: ASVs orderd by abundance in each sample type

Supplementary Table S5: Alpha diversity of all samples

Supplementary Table S6: Alpha diversity of all sample types with statistical groupings

Supplementary Table S7: Faiths diversity by sample

Supplementary Table S8: Statistical comparisons for Adonis2 groupings

Supplementary Table S9: Taxonomy of top 1000 ASVs in dataset

Sfig 1: Rarefaction curves of all samples

Sfig 2: Principal component analysis of Bray-Curtis distance between microbiomes of *Cassiopea* species.

Sfig 3: Upset plot of overlap between taxa found in bell, GVC, water and substrate samples.

Sfig 4: Heatmap of core microbiome prevalence in GVC and bell.

Sfig 5: Aldex2 plot of effect size of all ASVs between bell and GVC samples.

Sfig 6: Aldex2 plot of effect size of all ASVs between bell and substrate samples.

Sfig 7: Correlation plot (corrplot) of collected environmental and medusa factors across samples.

Sfig 8: Redundancy analysis plot of GVC

Sfig 9 : Redundancy analysis plot of bell samples.

Formatted: Font: Not Bold

Formatted: Font: Not Bold

Formatted: Font: Not Bold

Formatted: Font: Not Bold

Formatted: Font: Not Bold

Formatted: Font: Not Bold

Response to editor and reviewers:

To all,

Thank you for taking the time to review this manuscript. Your suggestions and critiques have been very helpful. The majority of changes in this round of the manuscript cover refinement of captions, movement of extraneous figures to the supplementary materials, and greater detail in some sections of the methodology section. Hopefully the in-text modifications improve the clarity and readability of the text.

Sincerely,

KMM

Reviewer 1

To Reviewer 1,

Thank you for your specific critiques. As you have suggested, the ethanol preserved samples have been removed from the methods section in order to improve clarity. Table #1 has been separated to provide adequate space for definitions and the term “GVC” is now clarified earlier. Additional analyses of the physical parameters have now been added to the supplements, as is addressed in the specific response below. Additionally, the limited nature of this sampling has now been reiterated in the core microbiome section.

Thank you,

Kaden Muffett

Specific points

1. Briefly, since the samples preserved in alcohol did not give good results, these should be removed from the manuscript.

   The authors accept this suggestion and have modified the text accordingly.

2. Table 1 needs work with headings, column spaces, and some definitions.
3. The GVC term should be introduced in Methods.
   Thank you for bringing up this oversight. The correction has been made.
4. The authors collected information on physical parameters, but such information was not relevant in their analyses, correct?
   Yes, as these were point-in-time measurements, it was deemed imprudent to include them in the primary analysis. Redundancy analysis plots (RDA) including

these features of interest have now been added to the supplementary materials for those interested. They are presented in this table to inform others' interpretation of these data, as factors associated with these have been demonstrated important in other microbiomes.

5. Finally, in the Results section referring to Core Microbiome, the authors need to stress that the sampling was done in one month within a single year.
   Thank you for the suggestion, this has now been specified in the results.

Reviewer #3

To reviewer #3,

Thank you for your review of this manuscript. Your suggestions have improved the precision and clarity of the text. In addition to your specific comments, you also indicated some more general inquiries regarding the nature of these microbiomes. While I am not capable of speaking to the function of the *Cassiopea* microbiome with these data, I do think that these questions are worth pursuing with other study designs.

Please see the responses to all of your specific requested edits below.

Sincerely,

KMM

Specific comments

1. Title: because both internal and external microbiomes were investigated, the words "gastric microbiome" limits the scope of the study, which is broader. My suggestion is to alter it.
   Thank you for your suggestion. The title has been modified to include both external and internal data.
1. Keywords: the word "Cassiopea" is already present in the title. Please, remove it from the keywords section and replace it with another one.
   Corrected.
2. Lines 28-29: not all stony corals, octocorals and sea anemones are symbiotic. I recommend replacing "the well-known" with "some".
   Corrected.
   Line 43: please, replace "hot" with "warm". Also, write the full name of C. andromeda, including authority and year of publication.
   Corrected.
3. Line 45: why is having a "sticky interface" significant? This phrase could be a little more informative.

Corrected. The text now specifies that this is a barrier between host and environment.

4. Line 47: please, add “SOME” stony corals and specify how much heat can Cassiopea tolerate as well as max and min temperature fluctuations.
   Corrected, specific tolerance details added.
5. -Line 98: please, write GVC in full. The same should be applied to caption of figure 1 and 3.
   Thank you, this issue has been corrected.
6. Line 117: my suggestion is to add "amplification" as well.
   Corrected**.**
7. Line 122: did you mean amplification?
   Corrected.
8. Line 137: great work at providing the codes and describing each command. Congrats!
   Thank you for your praise.
9. Line 139: please, write ASV in full the first time.
   Corrected.
10. Line 143: out of curiosity…Did you face any limitations in the completeness of the Silva database? Do you think that using additional databases would have provided a different outcome?
    It is certainly possible. The Silva database has made substantial strides in the last several years when it comes to marine taxa, so issues I had with ID in the first tests (2021) are largely absent now. RDP certainly was considered, but the comparability of Silva across studies is currently unbeatable. I also considered using the expanded McCauley classifier, but decided that, should someone want perfect taxonomic comparability to another study, they will likely need to reclassify anyway. Such is the reality of amplicon sequencing, and also our constantly in-flux marine microbial genera.
11. Line 151: GVC should be written in full since it's the first time it appears in the text.
    This has now been corrected earlier in the text (methodology section)
12. Line 152: please, rewrite it as “34.808 taxa” to keep numbers standardized.
    Corrected.
13. Line 179: what about the other 21 specimens?
    The other 21 included medusae under 3 cm diameter (9 indv), and samples that did not fit on the final sequencing run with the resources available. This has now been specified in the text.

14. Line 181: please, remove "GVC".
    Corrected.
15. Line 182: please, add authority and year of publication.
    Corrected.
16. Line 183: in total, 55 specimens were sampled, but only 34 were analyzed and out of these 4 were not identified as C. xamachana. Resulting in 30 specimens. Is that correct? If so, what happened to the other 17 specimens. That is not clear. What is the actual number of analyzed specimens?
    Thank you, the description of the dataset has been standardized across the paper. It is now clear that 55 were collected, of these 46 were swabbed, of these 34 samples were sequenced, and of these 30 were *C. xamachana*.
17. Line 196: please, remove "(top)" and "(bottom)".
    Corrected.
18. Line 205: please, remove "(GVC)"
    Corrected.
19. Line 228: please write ASVs in full every time it appears for the first time in a caption.
    Corrected.
20. Line 229: displayed?*
    Corrected.
21. Caption of figures 3 and 6: please, rewrite it explaining what each abbreviation mean (HHP, CK, LW…)
    Thank you for your feedback. The explanations are now available within table 1.
22. Line 243: please, invert to "location (b+c)". Line 245: please, invert to "GVC (b) and bell (c) alpha diversity", Line 256: please, write "PCA" in full. The same to line 352.
    Corrected.
23. Line 272: please, add a single space after "of" (e.g.: of > 0.1%).
    Corrected.
24. Line 280: Cassiopea should be in italics.
    Corrected.
25. Caption of figure 6: please, explain each abbreviation and write GVC in full the first time it appears in the caption. The same should be applied to all captions (tables and figures).
    This has been corrected across captions.
26. Line 288: please, correct the overspacing.
    Corrected.

27. Lines 318 - 322: why some organisms are more abundant than others depending on the area where they were sampled (e.g. bell, gvc)? Could this have anything to do with their functional role?
    It is entirely possible that this is related to functional role, however the assessment of functional role from 16S data, especially from divergent taxa, is difficult and error prone. This question is outside the scope of this paper but certainly an interesting one.
28. Line 353: “were” = where?*
    Corrected.
29. Line 360: Cotylorhiza tuberculata (Macri, 1778). Authority and year of publication should be added the first time a species name is mentioned. Please, revise all species names cited throughout the manuscript.
    Corrected.
30. - Line 436: please, see author guidelines for how studies should be cited. For example, here, a comma is used after “et al.”, but the same is not applied to the citation above.
    The erroneous citations have been modified, thank you for bringing this to my attention.
31. Line 446: remove “a” before “either”.
    Corrected.
32. Line 452: if the Cassiopea’s external microbiome does not differ from the sediment microbiome, does it mean that Cassiopea does not have exclusive external microbiome?
    This is a reasonable interpretation of the data. This community is enriched in *Endozoicommonas* relative to the benthos, as discussed elsewhere, but aside from this, the community is very similar to that of surrounding substrates.

Reviewer #4

Reviewer 4,

Thank you for taking the time to review this manuscript. You have raised two main concerns about the structure of this paper, the first being that we are not fully able to parse the impact of location with the samples collected, and secondly, that this paper has lapses in explanations of methodological purpose.

To the first point, I agree that the sample sizes are not adequate across all sites to justify this being the driver of the paper. In reality, what we have the ability to analyze is only the ways in which gastrovascular cavities and bells show consistencies. In order to bring the introduction into line with this, the introduction has been modified, including

both stressing that this is an exploratory paper and that the focus is to identify the core components of the GVC and bell communities.

To the second point, I have modified the paper such that the impetus for specific tests are clearer, and switched out the less informative DMM figure with a more informative one. Where there were too many figures, they have been moved to the supplementary materials, and where there was inadequate detail in the supplements (eg Shannon diversity), the supplementary materials have been expanded.

In addition to these, in alignment with your suggestion, Bray-Curtis is now used throughout the text.

Thank you for your feedback,

Kaden Muffett

Specific comments:

1. Since microbiome sequencing is a data-driven method, in which ASVs are sequence constructs derived from the original samples, a data-driven pipeline is necessary to ensure that scientific hypotheses are tested without bias.
   Thank you for this criticism. In this case, this is an exploratory study of this community, and while I understand the concern for bias, this study is simply a hypothesis generation mechanism. I accept your criticism as this: The aim of this paper specifically states "compare the microbial communities of Cassiopea across sites of the Florida Keys". I agree that I do not have adequate replicates to fully elucidate differences between sites. As such, I have changed the wording of this aim to reflect the core of what this work has accomplished, "The aim of this work is to identify the core components of the internal and external mucosal microbiomes of *Cassiopea* within the Florida Keys.".
2. This sentence also includes a typo: archaeal (not archael). A similar word choice appears to have been made in the title. Overall, the writing needs improvement.
   The typos throughout the text have been corrected.
3. Another issue is that there are too many figures in the main text. Some of them, such as Figure 1, could be moved to the Supplementary Files. The same applies to Table 1.
   Thank you for your suggestion. While I understand that some of these figures and tables may seem extraneous, this paper is serving a diverse group of scientists, and other reviewers have been and remain interested in the inclusion of this table.
    a. Many of the remaining figures should also be improved in terms of quality and explanation, as they currently appear without adequate context.

Thank you for this criticism. The figure captions have been modified to better explain the contents of each figure.

b. Why were the main phyla and classes depicted?
The main phyla and classes are depicted in this paper because of precedent. For past coral (Dunphy et al 2019 (https://doi.org/10.1038/s41598-019-43268-6), Hernandez-Agreda 2018 (https://doi.org/10.1128/mbio.00812-18)) and scyphozoan microbiome work (Ohdera et al 2024 (https://doi.org/10.1371/journal.pone.0298002) , Peng et al 2021 (https://doi.org/10.3389/fmicb.2021.647089)), this has been a common display mechanism. This figure is displayed for easy communication of the major groups found in these samples within this study.

c. Why use Euclidean distance, Faith's PD, or calculate the core microbiome?
Thank you for your questions. In this case, I did not fully consider the impact of Euclidean diversity. I have changed all beta diversity to Bray-Curtis throughout the text. For Faith's PD, I have now specified the following in the methods section "In order to identify the degree to which diversity spanned primarily shallow or deep phylogenetic distances, Faith's phylogenetic diversity was calculated for all samples and sample types. Faith's phylogenetic diversity was computed.." . For the core microbiome, an explanation of choice is now provided in the methods "In order to identify taxa consistently present across samples of a given type that may be rare, core microbiome was calculated for internal and external communities of *Cassiopea*. Core microbiome was computed.."

d. What is the Dirichlet multinomial mixture (DMM), and why was it used?
Dirichilet multinomial mixture analysis tests an ideal number of populations for a provided diverse population set. The purpose of this has now been clarified in the methods.
"In order to identify whether a gastrovascular/ bell split community partitioning was appropriate for all samples, and identify subcommunity level enterotypes within Cassiopea internal and external microbiomes, Dirichlet Multinomial mixture analysis, as described by Holmes and implemented in the DirichletMultinomial package v 1.48 was used for Cassiopea – derived samples (52,53). "

4. The rationale behind assessing the substrate and water samples is unclear. Grouping eight samples (one per site) and comparing them with thirty GVC and bell samples in Figure 2 seems counterintuitive. Please explain this logic more clearly to the reader. A simple Venn diagram of shared ASVs would more

effectively depict the percentage of ASVs shared among bell, water, substrate, and GVC samples. I could not extract this information from Table 2.

The inclusion of the substrate and water in figure 2 and table 2 serve as comparison tools to the microbiomes discussed. An upset plot of group to group overlap is now included.

5. Still regarding Figure 2, an "unknown" category should be added to group ASVs classified only as "Bacteria." This would create a bar that fills each sample (or group of samples) to 100%, aligning the bars in the plot. It is also helpful to limit the figure to the x most abundant taxa to avoid cluttering the legend with indistinguishable shades of similar colors.

   Thank you for your suggestion. The colors and design for this have been altered as suggested to aid comprehensibility.

6. I don't think the in-text Shannon values (L234–L240), or any diversity metric, are informative as currently presented. These should be included in a Supplementary Table with appropriate measures of dispersion and statistical tests, indicating statistically distinct groups with letters.

   Thank you for the suggestion. A table of Shannon diversity metrics is now available in the supplementary materials.

7. Why was only the GVC core microbiome analyzed and then searched in other samples (Figure 6)? What do the abbreviations GB, CK, VP, BKP, etc., refer to in this figure? These should be defined in the legend. Also, refer to the sampling table that should include these abbreviations. A map or schematic of these sites would be valuable for readers unfamiliar with the study area. I encourage the authors to consider improved ways to present this result. Figures 7 and 8 could be merged and moved to the Supplementary Material.

   The specificity of this gastrovascular cavity is of interest to the field. The explicit separation of internal and external samples is rarely done for cnidarian samples. As such, making it clear that the core of this community is not represented in other sample times is a key goal of this paper.

8. Regarding the DMM, no figure shows the model fit against the number of components (clusters), as stated in L309: “model probability value was optimal for two communities, one GVC and one bell.” This result should be presented to justify grouping the samples by tissue, as previously critiqued.

   Additional details on this are now provided in-text and supplementary figs have been added.

9. I cannot read the text in Figure 10. Please explain the star plot in the Results section.

Thank you for your comment. The reasoning behind this figure has been expanded in the results section.

10. The "Host medusa species identity" section should not be the final result, as it is disconnected from the rest of the section. If the intent was to show that there is no microbiome distinction between CA and CX, this could be stated at the beginning of the Results and shown in the Supplementary Material.
    Thank you for your suggestion. This section has been removed and moved to the supplements.
11. What is meant by "Retention metrics" (L144)? Does this refer to the number of reads retained after preprocessing? Please clarify this for the reader.
    Thank you for your comment. This has now been clarified in the text.
12. The provided GitHub repository (https://github.com/kadenmuffett/Cassiopea-Code-Microbiome-Repository) is not publicly accessible. Either correct this or provide the code as a Supplementary File.
    While I have not been able to replicate this issue, a copy of the code is now included in the supplementary material as well.
13. Why are WTR and substrate samples not described in the Sampling section alongside bell and digestive cavity samples (L103–L111)?
    Water and substrate samples are described immediately prior to GVC and bell samples. 1L of water was filtered through a 0.2um filter, then preserved in DESS. Sediment samples were preserved in DESS directly.
14. Abbreviations used throughout the manuscript should be introduced in this section, before they appear in the Results. For example, GVC is mentioned for the first time in the Table 1 legend without prior definition.
    This has been corrected.
15. Why is Bray-Curtis mentioned in the Methods (L161) but not in the Results? Remove it if it was not used. Please consider my suggestion above regarding this metric.
    Thank you for your comment. Euclidean has been replaced with Bray-Curtis throughout the text.
16. Figures 1, 2, and 7 are of poor quality — the text is unclear. Were they uploaded as vector files? In Figure 2a, the text within the box is unreadable.
    Please be assured that the image quality displayed in the reviewer pdf is poor quality and not representative of the quality of the uploads.
17. Also, regarding the Results, current phylum-level nomenclature should be used alongside classical names (L192–L193). For example, Pseudomonadota (formerly Proteobacteria).

Thank you. Bacterial phylum names have been modified to reflect this.

18. While I don’t prefer sections within the Discussion, the text is interesting and connects the findings to the scientific literature. Including more explicit references to the underlying Results would further improve it.

    Thank you, some additional connection to the test has been added to improve congruity across sections.

19. Please cite SILVA database (10.1093/nar/gks1219) (L144)

    This has been corrected.

20. Refraction curves? (L155). Rarefaction

    This typo has been corrected.
