# Supplementary material for: Florida Keys Cassiopea host benthos-like external microbiomes and a gut dominated by Vibrio, Endozoicomonas and Mycoplasma
Source: PLoS One. 2025 Aug 12;20(8):e0330180. doi: 10.1371/journal.pone.0330180 (PMC12342295; doi:10.1371/journal.pone.0330180)
Supplement: Attachment — Submitted filename: 01.13.25_Response to editor and reviewers.docx [file pone.0330180.s005.docx]

**Response to editor and reviewers**

**Dear editor and reviewers,**

**After careful review of your suggestions and criticisms, the methodological and statistical analyses in this manuscript have been revisited, redone, and in the process greatly improved. Many of your broad concerns in these areas are addressed with a significantly expanded methods section.**

**Sincerely,**

**KMM**

**Response to editor:**

Thank you for your considered feedback.

In addition to the improved manuscript, please note these specific changes:

1. Inclusion of the funder role statement along with the manuscript
2. Corrected Funding statement in cover letter
3. Fixed formatting in manuscript
4. Added ethics statement

**Comments to reviewer 1:**

Thank you for your careful feedback. After substantial revision to the backbone of this work, we hope you will find all of your comments duly taken into consideration. Please see specific corrections in addition to the broader overhaul in the response below.

1. *Abstract needs to mention consistent results from across sampling sites.*
   a. Thank you for your feedback. This has been corrected.
2. *Clarify inclusion of C. andromeda in analysis.*
   a. In the text (ln 146) it now clarifies that *C. xamachana* only are used for downstream analyses with the exception explicitly comparing the two mitotypes (ln 298).
3. *Standardize “benthic” sample name*
   a. Thank you for your suggestion. All substrate samples are now referred to as substrate as opposed to benthic.
4. *Figure 2*
   a. Figure two has been replaced with an improved version that includes your suggestions.
5. *Figure 3*
   a. Figure 3 has been replaced and now is a chart of Shannon and observed diversity
6. *Citation for Dirichlet multinomial analysis*
   a. The lack of reference to Holme’s original work has been rectified.
7. *Line 227: claim about clustering distinction*
   a. Thank you. This claim has been removed and has been replaced with Faith’s D and more appropriate accompanying statistical tests.
8. *Core microbiome information incomplete*
   a. Thank you for the feedback. This section was indeed incomplete and confusing. It has been replaced with analyses from the “microbiome” r package that are more appropriate.
9. *Lack discussion of external microbiome*
   a. Thank you for this comment. There is now a section discussing this within the paper.
10. *Seasonality and size comments unnecessary*
    a. Thank you for your feedback, this section has been removed.

Specific comments:

- *Line 33: “These communities” please specify you are referring to “microbial communities”*
  - This issue has been noted and corrected.
- *Line 38: replace “clade” with “Family”*
  - This issue has been noted and corrected.
- *Line 47: the statement about the tolerance of Cassiopea to intense temperature fluctuations needs a reference*
  - This issue has been noted and corrected.
- *Line 63: I do not understand the purpose of the statement: “Pathogenesis is often a focus…”*
  - This statement has been clarified and corrected.
- *Line 150-151: Why were you not able to use the Siva 138 database?*
  - This work now uses the most up-to-date silva database.
- *Line 165: “benthic OR water” should not be “water AND sediment”?*
  - This issue has been noted and corrected.
- *Line 216: Please revise “Family richness was significantly lower within medusae”*
  - This line has been corrected.
- *Line 227: after referring to your results, it would be good to add a figure number*
  - Figure numbers are now added in correct positions.
- *Lines 280-282: this statement has no support. It could work the other way, going from the exterior but remaining an important component in the internal cavity. Please revise.*
  - This error has been noted and replaced.
- *Lines 299-300: I do not understand why you mention this statement, since in your results no identification of “obligate” taxa was made for the GVC. Please revise.*
  - This statement has been noted and revised.
- *Line 314: a space is needed before the parenthesis*
  - This issue has been corrected.
- *Lines 317-319: is not clear what point are you trying to make.*
  - Thank you for the feedback, this statement has been removed and replaced with one of greater clarity.
- *Line 326: use “Family” rather than “clade”*
  - Corrected.
- *Line 327: orphan phrase? “…appear to harbor the microbiome, …”*
  - *Thank you for the feedback, this statement has been clarified and reworded.*
- *Line 381: I suggest changing “lacks a conservatively defined core microbiome” for “in our samples, no core microbiome could be demonstrated”*
  - *This statement has been removed and replaced after reanalysis.*

**Comments to reviewer 2:**

The authors thank reviewer 2 for their supportive feedback. The poor explanation of statistical tests and underanalysis of the dataset at hand presented key failings that have been carefully corrected in the updated version. We hope that these revisions make this work more meaningful and improve readability.

*Reviewer #2: The authors study the microbiome composition of two upside-down jellyfish species in the Florida Keys using 16S rRNA barcoding. The paper describes the main bacterial taxa in different locations and describes the microbiome composition focusing on an understudied group. They briefly compare these findings with the existing literature for other anthozoan groups.
I found the study interesting, but the results seem overstated, and some important information is missing. For example, the title does not accurately reflect the study's findings. It should better represent the study’s scope and avoid claiming general patterns that the data does not appear to support. My major concern is the lack of explanation in the statistical analyses. I think improving this part will help to better understand the analyses and evaluate the content of the manuscript.*

Responses to specific comments:

- *Overall, there is a lack of information on sample sizes throughout the study. This omission makes it difficult to assess the balance of the study design or to understand how many samples are being compared in each analysis. Sample size details should be included in the figures, methods, and results sections.*
  - Thank you for your comment. The sample size information is now added to the first table and where relevant in other sections.
- *The authors use two different preservatives—ethanol and DESS—that appear to be combined without distinction. I recommend including a statement in the methods on how this might affect microbiome composition. If the authors tested for potential effects, the results should be included, or they should reference studies clarifying whether these preservatives could influence the results.*
  - Ethanol was not used for any downstream analyses, a statement that has now been added to the methods section to clarify.
- *Table S1: Please provide descriptions for abbreviations used to aid comprehension.*
  - Clarification has now been added to the supplementary information.
- *Table 1: Please add the sample sizes per site.*
  - Corrected
- *L129-135: If PacBio sequences were not used in the study, I recommend removing this from the methods section.*
  - Corrected.
- *L157: Please describe the statistical analyses clearly enough to allow reproducibility. This section lacks essential details.*
  - Corrected.
- *Figure legends need explanations of the content. They are currently limited to single-line titles, which restricts comprehension. I recommend revising the figure design, e.g. Figure 1.*
  - Corrected
- *L226: "significantly different".*
  - This statement has been replaced and clarified.
- *Figure 4: The stress level is high. How balanced is this comparison? Although the figure appears to show a comparison analysis, the text refers to a pairwise comparison. Could the authors clarify the type of analysis used here?*
  - Thank you for your comment. This analysis has been replaced witn a more appropriate MDS of Euclidean distance from the corrected data.
- *The results section presents many analyses and tests that were not introduced in the methods section. Additionally, the results refer to supplementary tables without legends, making it difficult to assess the accuracy of these tests. I recommend a more detailed explanation of the analyses conducted.*
  - This consistent issue has been corrected and all analyses are now listed in the revised version of the manuscript.
- *Figure 2: The y-axis label is missing.*
  - This figure has been replaced with a more appropriate figure.
