# Supplementary material for: Florida Keys Cassiopea host benthos-like external microbiomes and a gut dominated by Vibrio, Endozoicomonas and Mycoplasma
Source: PLoS One. 2025 Aug 12;20(8):e0330180. doi: 10.1371/journal.pone.0330180 (PMC12342295; doi:10.1371/journal.pone.0330180)
Supplement: Attachment — Submitted filename: Reviewer response_PLOS_07_2025.docx [file pone.0330180.s008.docx]

Reviewer response

**Dear editor and reviewers,**

**Thank you for your thoughtful commentary and criticism. In response to these reviews, figures 2 and 9 have been corrected, and a variety of clarity and spelling issues have been fixed throughout the text. I hope that these changes are satisfactory.**

**Sincerely,**

**KMM**

To the editor;

The following changes have been made in line with suggestions:

1) Statements in abstract: The high diversity of bell-associated microbes should be mentioned.

We have accepted this note and modified the abstract.

2) Figure 2: The correct naming convention for Actinobacteria should be double checked (Actinobacteria vs. Actinomycetota).

The outdated name has been replaced with the correct “Actinomycetota” within the text.

3) Figure 9 requires some more clarification regarding its description, in particular the term ‘identical’, axis labeling, and description of specific sections in the figure. This should be clarified.

For figure 9, the y-axis, title and figure label have been modified to impart greater clarity. “Identical” has been removed as it is uninformative in this context. A supplementary table has been added with best matches for the top six *Vibrio* cf. *coralliilyticus* sequences and referenced within the text.

4) Please note that one of the reviewers has uploaded an annotated PDF highlighting a few remaining points.

The typo fixes and clarifications provided by reviewer two have been accepted into the body of the text.

5) Editor’s note: Please also double check the correct spelling for microbial taxa. ‘Endozoicomonas’ should be spelled with only 1 ‘m’. There are 14 instances of misspelling of this genus name (with 2 ‘m’, i.e., ‘Endozoicommonas’), including in the title.

Corrected.

**Comments to the Author**
Reviewer #1

To reviewer #1,

Thank you for reviewing. Your suggestion and minor recommendations have been accepted into the text.

Sincerely,

Kaden Muffett

1) In the abstract, I find the high diversity of the bell-associated microbes, with no clear dominance of any group, more relevant than the written phrase “Cassiopea bell mucosal samples conform largely to the communities of surrounding sediment with the addition of Endozoicomonas cf. atrinae.” Perhaps the authors can include both?

Corrected.

2) Minor recomendations:
   Lines 24 and 449: Symbiodiniaceae should NOT be in italics
   Line 38: in the parenthesis, Symbiodiniaceae is not a clade, is a family

Corrected.

**Reviewer #3**

To reviewer #3,

Thank you for reviewing, as well as for your praise of the manuscript. Your edits identifying typos and grammatical errors has significantly improved the readability of the text.

Sincerely,

Kaden Muffett

**Reviewer #4**

To reviewer #4,

Thank you for reviewing. Figures 9 and 2 have been revised to correct identities and improve clarity. The word “Spoke” has been replaced with “point” as readers are likely more familiar with this in star plots. Details of the ASV blast search are now included in the text.

Sincerely,

Kaden Muffett

1) Figure 9 and its accompanying description remain somewhat unclear. I found the use of the term "identical" (L379) confusing, as its meaning is only clarified later in the Discussion section (L531). I recommend that the BLAST search, in which an ASV is used as a query, be described explicitly in the Results section. Regarding Figure 9, the y-axis label is "Abundance," yet the authors mention that the abundance reached "up to 10% within the sample" (L424). In this context, the y-axis label should be revised to "Relative abundance." Additionally, if only Vibrio coralliilyticus reads were plotted, the figure’s appearance as a stacked bar plot is misleading. What do the sections within each bar represent—values from samples of the same type or location? The bars are also visually cluttered; replacing the inner background grid lines with area shading or patterned fills could improve readability.

The blast search is now described in the text and present in a supplementary table with closest blast identities from the curated NCBI rRNA database. The y-axis has been changed to relative abundance, the title has been clarified, as has the description. The uninformative legend was removed and the x-axis was modified to improve readability.

2) Figure 2 – Actinobacteriota. Does it refer to Actinobacteria? If it does, the accepted nomenclature is Actinomycetota. Please consult the LPSN website to use the up-to-date nomenclature (https://lpsn.dsmz.de/).

Thank you for the feedback. This has now been corrected.

3) Minor corrections:
   "Spoke" (L411) appears out of context—please clarify or revise.

“Spoke” has been replaced with the more common “point” for the starplot.
